# Catalyst-free late-stage functionalization to assemble α-acyloxyenamide electrophiles for selectively profiling conserved lysine residues

**DOI:** 10.1038/s42004-024-01107-4

**Published:** 2024-02-14

**Authors:** Yuanyuan Zhao, Kang Duan, Youlong Fan, Shengrong Li, Liyan Huang, Zhengchao Tu, Hongyan Sun, Gregory M. Cook, Jing Yang, Pinghua Sun, Yi Tan, Ke Ding, Zhengqiu Li

**Affiliations:** 1 State Key Laboratory of Bioactive Molecules and Druggability Assessment, Jinan University, 601 Huangpu Avenue West, Guangzhou, 510632 China; 2 International Cooperative Laboratory of Traditional Chinese Medicine Modernization and Innovative Drug Development (MOE), School of Pharmacy, Jinan University, 601 Huangpu Avenue West, Guangzhou, 510632 China; 3 Guangdong Second Provincial General Hospital, Postdoctoral Station of Traditional Chinese Medicine, Jinan University, Guangzhou, 510632 China; 4 Department of Chemistry and COSDAF (Centre of Super-Diamond and Advanced Films), City University of Hong Kong, 83 TatChee Avenue, Kowloon, Hong Kong, 999077 China; 5 Department of Microbiology and Immunology, University of Otago, Dunedin, 9054 New Zealand; 6 Guangzhou National Laboratory, Guangzhou International Bio Island, Guangzhou, 510005 China; 7 MOE Key Laboratory of Tumor Molecular Biology, Jinan University, 601 Huangpu Avenue West, Guangzhou, 510632 China

**Keywords:** Proteomics, Target identification, Chemical tools, Chemical modification, Mass spectrometry

## Abstract

Covalent probes coupled with chemical proteomics represent a powerful method for investigating small molecule and protein interactions. However, the creation of a reactive warhead within various ligands to form covalent probes has been a major obstacle. Herein, we report a convenient and robust process to assemble a unique electrophile, an *α-*acyloxyenamide, through a one-step late-stage coupling reaction. This procedure demonstrates remarkable tolerance towards other functional groups and facilitates ligand-directed labeling in proteins of interest. The reactive group has been successfully incorporated into a clinical drug targeting the EGFR L858R mutant, erlotinib, and a pan-kinase inhibitor. The resulting probes have been shown to be able to covalently engage a lysine residue proximal to the ATP-binding pocket of the EGFR L858R mutant. A series of active sites, and Mg^2+^, ATP-binding sites of kinases, such as K33 of CDK1, CDK2, CDK5 were detected. This is the first report of engaging these conserved catalytic lysine residues in kinases with covalent inhibition. Further application of this methodology to natural products has demonstrated its success in profiling ligandable conserved lysine residues in whole proteome. These findings offer insights for the development of new targeted covalent inhibitors (TCIs).

## Abstract (web summary)

Covalent probes are a powerful tool for investigating small molecule and protein interactions, however, the development of reactive warheads to form covalent probes remains underexplored. Here, the authors develop *α*-acyloxyenamide electrophiles to covalently bind to lysine residues, and selectively profile the conserved lysine residues of kinase in live cells.

## Introduction

In recent years, targeted covalent inhibitors (TCIs) such as pyrotinib, osimertinib, ibrutinib, and sotorasib have received considerable attention due to their remarkable pharmaceutical properties^1,2^. The significant advantages of this kind of inhibitors over non-covalent inhibitors include greater potency, excellent selectivity, and prolonged duration of action^3,4^. However, the reactive warhead of these inhibitors is usually limited to *α*,*β*-unsaturated amides and the target is only the cysteine residue^5^. Due to the high reactivity of cysteine residue and its mutation, acquired drug resistance usually occur in the treatment of patients. Moreover, many protein binding pockets lack a targetable cysteine^6^, it is thus necessary to develop novel electrophiles that can target less nucleophilic residues such as conserved lysine and glutamic or aspartic acid and identify ligandable conserved residues. For example, Taunton et al. developed a class of sulfonyl fluoride probes that can covalently label lysine residues in various protein kinases^7^, and they recently reported reversible covalent kinase probes based on a salicylaldehyde for in vivo studies^8^. Campos et al. reported an active ester probe that can produce selective and irreversible inhibition of PI3Kδ by targeting a catalytic lysine in the protein kinase^9^. Yao et al. reported the use of *o*-boronic acid benzaldehyde, SuFEx, and salicylaldehyde to afford BCR-ABL kinase inhibitors, which target the conserved lysine residues^10,11^. Our group recently developed a 3-phenyl-2H-azirine and a coupling reagent, ynamide, as tool probes for the selective profiling of carboxylic acid residues in live cells^12,13^, and found that the ynamide-based kinase probes can covalently target a conserved glutamic acid in the ATP binding pocket of EGFR L858R mutant^13^. In this study, we further developed a new approach for exploration of covalently targetable lysine residues of kinases from live cells, and applied this method in natural products for profiling conserved lysine residues. These might be useful information for the development of new types of targeted covalent inhibitors.

Covalent chemistry integrated with chemical proteomics has been a powerful approach to the investigation of small molecule-protein interactions under in situ conditions^14^. It can not only identify ligandable binding sites in the vicinity of a binding pocket, but can also facilitate an understanding of natural protein functions and the biological properties of small molecules^15^. To create a covalent probe, a highly activated electrophile such as an *N*-hydroxy-succinimide (NHS)^16^, sulfotetrafluorophenyl (STP) ester^17^, alkyloxyacyl imidazole^18^, tosylate^19^ or *N*-acyl-*N*-alkyl sulfonamide^20^, is usually required to embed into a pharmacophore, enabling the surrounding amino acid residues are selectively labeled after ligand binding. However, difficulties usually encountered during the course of incorporation of an electrophilic warhead into various non-covalent ligands, especially the complex natural products^21–23^. It is thus highly desirable to develop a convenient and efficient approach for introduction of a latent electrophile into a ligand at a late-stage in the process.

**Fig. 1:**
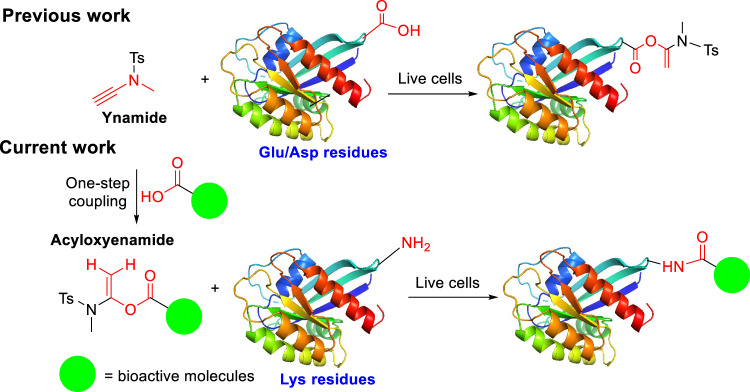
Proposed mechanism of protein labeling by α-acyloxyenamide intermediate at lysine residues. α-acyloxyenamide electrophiles covalently bind to lysine residues and selectively label conserved lysine residues in live cells.

It has been observed that many bioactive molecules, including natural products either have a carboxylic acid group, or can be easily embedded with a carboxylic acid group. This carboxylic acid can readily react with a commercially available coupling reagent, ynamide, to achieve a stable latent electrophilic *α*-acyloxyenamide without any catalyst (Fig. 1 and Supplementary methods in supporting information)^24^. Importantly, this process can tolerate other functional groups such as hydroxyl groups present in a pharmacophore^24,25^. We envisioned that this could be a general approach for various bioactive molecules in creation of covalent probes, and we further applied this method in proteome-wide profiling of ligandable conserved lysine residues by using probes derived from kinase inhibitors and natural products.

## Results and discussion

### Development of α-acyloxyenamide-based probes and applications in protein labeling

**Fig. 2:**
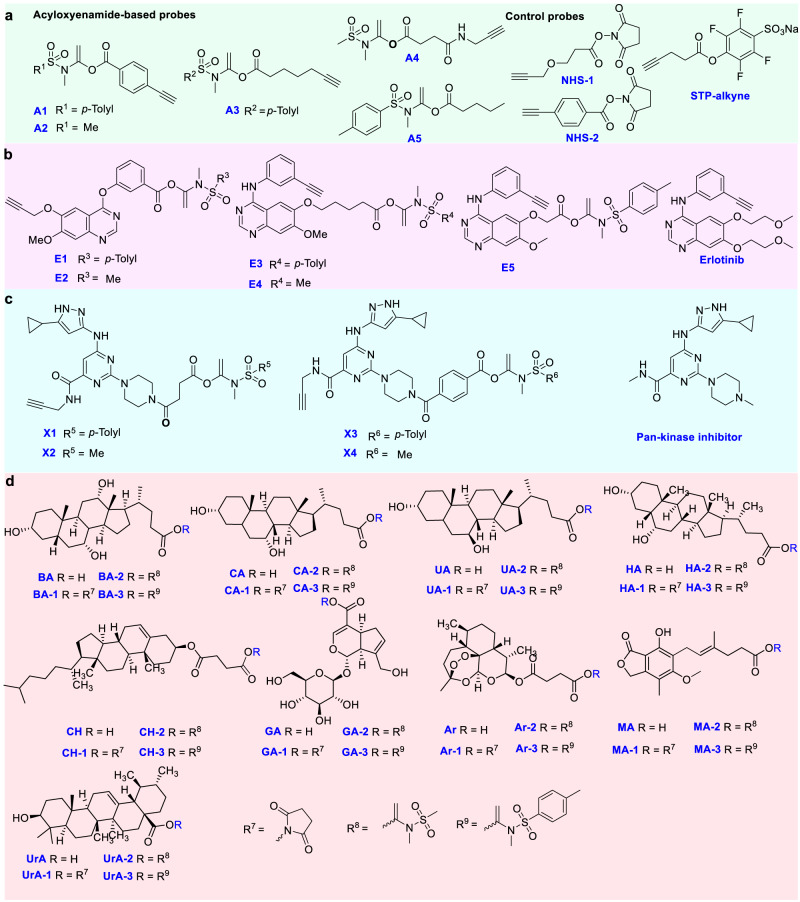
The chemical structures of probes. **a** Aromatic and aliphatic probes **A1**, **A2**, **A3**, **A4**, and **A5** with an *α*-acyloxyenamide reactive group and the chemical structures of control probes, aliphatic or aromatic probes NHS-1/2, STP-alkyne. **b** Erlotinib and Erlotinib-based probes **E1**, **E2**, **E3**, **E4**, and **E5** with an *α*-acyloxyenamide embedded into different positions. **c** *α*-acyloxyenamide was embedded into a pan-kinase inhibitor to form the probes **X1**, **X2**, **X3**, and **X4** and the parent inhibitor. **d** Structure of natural products and probes by incorporation with NHS and *α*-acyloxyenamide for comparison. BA cholic acid, CA chenodeoxycholic acid, UA ursodeoxycholic acid, HA hyodeoxycholic acid, CH cholesteryl hemisuccinate, GA geniposidic acid, Ar artesunate, MA mycophenolic acid, UrA ursolic acid. The synthesis was presented in supplementary scheme S1–S41 and the NMR spectra of the probes were in Supplementary data 2.

To test this idea, four *α*-acyloxyenamide-based probes, **A1**, **A2**, **A3** and **A4**, were created by the coupling reaction between a benzoic or an aliphatic acid with commercially available ynamides, *N*-methylynemethylsulfonamide (MYMsA) and *N*-methylyne-toluenesulfonamide (MYTsA) (Fig. 2a)^26,27^. A clickable alkyne was embedded into the probes for subsequent bioconjugation with reporter tags. Probes **A1**, **A2**, **A3**, and **A4** were synthesized smoothly in good to excellent yield by a similar route (Schemes S1-S4, supporting information). An alkyne free analogue (**A5**) was also prepared to eliminate background labeling in target profiling of bioactive molecules (Scheme S5, supporting information). The well-known lysine-targeting probes NHS ester (NHS-1/-2) and tetrafluorophenyl esters (**STP-alkyne**) were used for comparison. With these probes in hand, we first tested the stability of **A1** and **A4** in PBS and cell culture medium by HPLC or NMR analysis with high or low probe concentrations, which showed that A1 remains unchanged after 24 h and **A4** is unstable in PBS solution (Supplementary Fig. 1). This demonstrated good stability of aromatic *α*-acyloxyenamide under physiological conditions. To test if an *α*-acyloxyenamide electrophile can react with an amino group, model reactions between probe **A1**/**A2**/**A3**/**A4** and benzylamine or ethylamine were carried out (Scheme S42 in Supporting Information). HPLC analysis revealed that probes **A1**/**A2**/**A3** can react with amino group smoothly and **A2** displayed the highest reaction velocity, while aliphatic probe **A4** failed to form the amination product (Supplementary Fig. 2). The instability of **A4** in PBS might be the reason of failure amination. The reaction between probe **A1** and different amino acids also confirmed that the probe can selectively react with lysine residues (Supplementary Fig. 3), demonstrating good amino acid selectivity. To investigate if this electrophile could be used as a warhead in kinase inhibitors for ligandable site mapping, a series of probes **E1**, **E2**, **E3**, **E4**, and **E5** based on the non-covalent drug erlotinib^28,29^, were synthesized by installation of an aliphatic acid or aromatic acid group into the pharmacophore, and a subsequent coupling reaction with *N*-methylynemethylsulfonamide or *N*-methylynetoluenesulfonamide (Fig. 2b, Schemes S6–S10 in supporting information). In order to profile the conserved lysine residues in the active site of kinases from whole proteome^7,8^, the reactive group was embedded into a promiscuous kinase inhibitor to produce probes **X1**, **X2**, **X3**, and **X4** (Fig. 2c, Table 1 in Supporting Information). The probes can be readily synthesized using the same protocol as was used for the erlotinib-based probes (Schemes S11–S14). The electrophile was positioned optimally in the hydrophilic moiety of the parent molecules, **E3**, **E4**, **E5**, **X1**, **X2**, **X3** and **X4**, to avoid affecting the binding of the probe with target protein^30,31^. For comparison, two probes, **E1** and **E2**, were prepared by incorporation of the electrophile at the recognition unit. To evaluate the performance of different electrophilic warheads in bioactive molecules, *N*-hydroxy-succinimide and *α*-acyloxyenamide were incorporated into a series of natural products including cholic acid, chenodeoxycholic acid, ursodeoxycholic acid, hyodeoxycholic acid, cholesteryl hemisuccinate, geniposidic acid, artesunate, mycophenolic acid, ursolic acid to afford the probes **BA-1/2/3,** **CA-1/2/3,** **UA-1/2/3,** **HA-1/2/3,** **CH-1/2/3,** **GA-1/2/3,** **Ar-1/2/3,** **MA-1/2/3,** **UrA-1/2/3** for comparison (Fig. 2d, Table 1 in Supporting Information). Mycophenolic acid was previously identified as an IMPDH2 inhibitor^32^. The results showed that the acid group in different natural products can be smoothly converted to an *α*-acyloxyenamide in excellent yield at a late stage (Fig. 2d, Schemes S15–S41). The NHS derived probes were produced by using DCC as a catalyst.

**Fig. 3:**
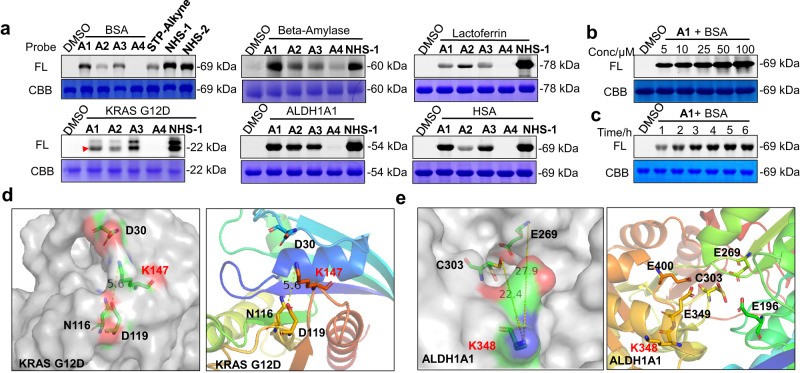
In vitro proteome labeling and modification sites analysis. **a** Labeling profiles of **A1**, **A2**, **A3**, **A4**, **STP-alkyne**, **NHS-1**, **NHS-2** (50 μM) with BSA. And labeling profiles of **A1**, **A2**, **A3**, **A4**, **NHS-1/-2** (50 μM) with β-Amylase, Lactoferrin, KRAS G12D, ALDH1A1, HSA. FL = in-gel fluorescence scanning. CBB = Coomassie gel. **b** Concentration- and **c** Time-dependent labeling profiles of **A1** (50 μM) with BSA. **d** Crystal structure of the K147 site in Kras G12D (PDB code: 4LPK). **e** K348 in ALDH1A1(PDB code: 5L2M).

The labeling capability of the probes with recombinant proteins was first examined. Commercially available or purified proteins, including bovine serum albumin (BSA), HSA, *β*-amylase, lactoferrin, KRAS G12D and ALDH1A1 were selected as model proteins. Commercially available lysine-targeting probes, **STP-alkyne** and **NHS-1/2** (Fig. 2a), were used for comparison^33,34^. After incubation of the probes with pure protein in PBS for 4 h, the resulting samples were conjugated to TAMRA-N_3_ under click chemistry conditions. The labeled proteins were separated by SDS-PAGE and analyzed by fluorescence scanning. As shown in Fig. 3a, the probe **A1**, containing two aryl groups, displayed the highest labeling capability under the same conditions. Negligible labeling was observed with the aliphatic probe **A4**, and probes **A2** and **A3** containing one aryl group which produced fluorescence with moderate intensity. This demonstrated that the aromatic group is beneficial for probe binding to proteins, and the instability of probe **A4** might be the reason for the failure of labeling profile. The trend is consistent with the amination reaction by HPLC analysis in PBS buffer (Supplementary Fig. 2). Similarly, **A1,** **A2** and **A3** can successfully label HSA, *β*-amylase, lactoferrin, KRAS G12D and ALDH1A1. The fluorescence intensity of **A1** is comparable to that of STP-alkyne and NHS-1/2 (Fig. 3a, Supplementary Fig. 5), demonstrating that the *α*-acyloxyenamide electrophile is able to efficiently crosslink with the amino acid residues of different proteins. Concentration- and time-dependent labeling experiments showed that a probe concentration as low as 5 µM and an incubation time as little as 1 h is sufficient to produce visible bands, and saturated fluorescence signals were observed at 10 µM concentration after a 2 h incubation time, signifying excellent labeling efficiency (Fig. 3b, c). To characterize the binding site of the probes with these proteins, BSA, ALDH1A1 and KRAS G12D labeled with probes **A1** were digested with trypsin and then tested by LC-MS/MS. As shown in Fig. 3d and Tables S1–S3 (Supplementary Data 1 and Supplementary Figs. 26, 27), a total of 33, 3 and 10 lysine residues were detected by **A1** from BSA, KRAS G12D and ALDH1A1, respectively. Searches of the Uniprot database showed that K147 of Kras G12D and K348 of ALDH1A1 are located close to the GTP binding site and the NAD^+^ binding site, respectively (Fig. 3d, e), indicating that this probe is capable of labeling essential amino acid residues and potentially modulating the protein function.

### The performance of *α*-acyloxyenamide warhead in live mammalian cells

**Fig. 4:**
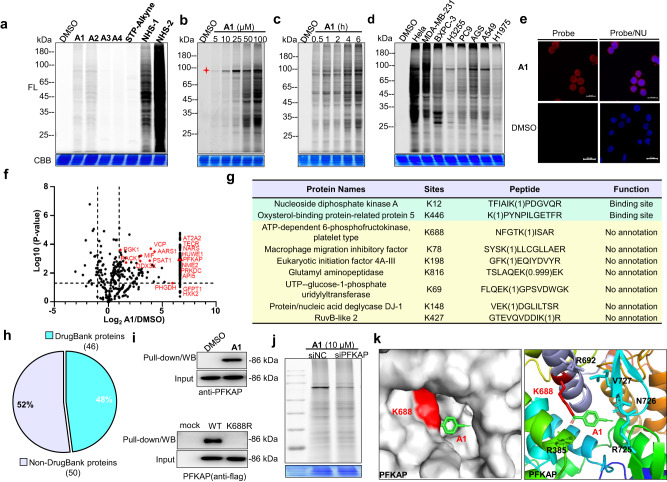
In situ protein labeling and modification site analysis. **a** Proteome reactivity profiles of **A1**, **A2**, **A3**, **A4**, **STP-alkyne** and **NHS-1/**2 (100 μM) with MDA-MB-231 live cells. FL = in-gel fluorescence scanning. CBB = Coomassie gel. **b** Concentration- (4 h incubation) and **c** Time-dependent labeling profiles of A1 (100 μM) with MDA-MB-231 live cells. **d** Proteome reactivity profiles of **A1** (100 μM) with different cancer cells. **e** Cellular imaging of A1 (50 μM) in MDA-MB-231 live cells. NU = nucleus, scale bar = 20 μm, DMSO-treated samples were used as controls. **f** Protein hits identified by pull-down/LC−MS with A1 (10 μM), DMSO-treated sample was used as a negative control. **g** Subset of residues identified by **A1** (100 μM) with MDA-MB-231 live cells. **h** Among the proteins with lysine modification by **A1**, the fraction found in DrugBank. **i** Target validation by pull-down/WB with **A1** (10 μM), the western blots used PFKAP antibodies (upper gel). Pull-down/WB of wild type and K688R PFKAP with **A1** (10 μM), the western blots were confirmed by using flag antibodies (lower gel). **j** Labeling of target protein PFKAP by probe **A1** after gene knockdown. **k** Docking experiments to predict the binding mode of **A1** with PFKAP at K688 (PDB code: 4WL0).

First, antiproliferation assay and cell apoptosis assay showed that weak anticancer activity was observed from **A1/A2/A3/A4** probes (Supplementary Figs. 4 and 14d), indicating low toxicity of this electrophilic warhead. Next, the proteome reactivity profiles of the probes with a triple-negative breast cancer cell line, MDA-MB-231, was examined. After incubation of **A1, A2, A3** or **A4** and the control probes STP-alkyne and **NHS-1/2** with live cells for 4 h, the cells were lysed and conjugated with TAMRA-N_3_. The resulting proteomes were analyzed by in-gel fluorescence scanning after SDS-PAGE gel separation. Consistent with the trend of the labeling with pure proteins, strong fluorescence bands throughout the whole lane were observed from the aromatic probes **A1** and **A2**, but not from the aliphatic probe **A4** (Fig. 4a), displaying excellent reactivity of the probes in cells. The labeling profile of **A1, A2** was sustained for at least 8 h of washing out, demonstrating a robust covalent protein modification (Supplementary Fig. 8). Different fluorescence bands between samples treated with **A1** or **A2** and those treated with **NHS-1/-2** probes were observed, indicating that *α*-acyloxyenamide-containing probes have unique protein hits. The **STP-alkyne** probe, however, failed to produce any labeling bands in live cells, likely due to the poor cell permeability. Concentration- and time-dependent labeling experiments showed that a probe concentration as low as 5 µM and an incubation time as little as 0.5 h is sufficient to produce clearly observed labeling profiles (Fig. 4b, c). Interestingly, a specific labeling band at ~80 kDa was observed at low probe concentrations (* marked band, Fig. 4b). When applied in different cancer cell lines, including HeLa, BXPC-3, H3255, PC9, AGS, A549, and H1975, stronger labeling profiles were observed from HeLa and MDA-MB-231 cells than from the other cancer cell lines, demonstrating distinct labeling profiles of the probe under different cellular conditions (Fig. 4d, Supplementary Fig. 6). The ~80 kDa band can be observed from various cancer cell lines with the **A1** probe, underscoring the excellent selectivity toward this particular protein. Notably, different labeling profiles between live cells and cell lysates can be observed (Supplementary Fig. 7), indicating varied labeling capability of the probes in various biological system.

To track the subcellular locations of the probes, cellular imaging experiments with **A1**, **A2** or **A3** probes were carried out. After incubation of each of the probes with MDA-MB-231 cells for 4 h, the cells were fixed and permeabilized, subjected to click chemistry with TAMRA-N_3_, and then imaged by microscopy. As shown in Fig. 4e and Supplementary Fig. 9, the fluorescence signal of **A1**, **A2** or **A3** spreads to the whole cell including the nucleus, and this supported the excellent labeling capability. The fluorescence intensity generated from the **A1** or **A2** probe is higher than the intensity from **A3** (Supplementary Fig. 9), which agrees with the results of the labeling profiles. Taken together, these proteome labeling and imaging experiments demonstrated that the *α*-acyloxyenamide is an efficient electrophilic warhead for protein labeling in live cells.

To characterize the labeled protein hits, a pull-down/LC-MS experiment was carried out. MDA-MB-231 cells were incubated with **A1** or DMSO for 4 h, and the cells were then lysated and conjugated with biotin-N_3_. The probe-labeled proteomes were affinity-enriched with streptavidin beads, and the resulting samples were characterized by LC-MS/MS after trypsin digestion. As shown in Fig. 4f and Table S4 in Supplementary Data 1, a total of 560 protein hits were positively identified. Of these, 394 showed a label-free quantification ratio greater than 2 (Probe versus DMSO) and were designated as “high-confidence” proteins. The molecular weight of ATP-dependent 6-phosphofructokinase, platelet type (PFKAP) corresponds to the specific ~80 kDa band in labeling profiles (Fig. 4b, d). This protein is overexpressed in a variety of cancer cells and is closely related to cell proliferation and differentiation^35^. Its functions include catalysis of the phosphorylation of D-fructose 6-phosphate to fructose 1,6-biphosphate by ATP, which is the first step of glycolysis^36^. This protein was further validated by pull-down/western blotting using corresponding antibodies (Fig. 4i, upper gel). Two other PFK proteins, ATP-dependent 6-phosphofructokinase muscle type (PFKAM) and liver type (PFKAL), were also positively detected, indicating that this probe can successfully label all PFK proteins in live cells^37^. Other protein hits that were detected include protein kinase, DNA-activated, catalytic subunit (PRKDC), hydroxysteroid 17-beta dehydrogenase 12 (DHB12), NAD(P)H quinone dehydrogenase 1 (NQO1) and nucleoside diphosphate kinase B (NME2) and proteins that are related to various diseases^38–41^.

To assess the amino acid reactivity and selectivity of the probes in complex proteomes, binding site analysis was carried out using pChem search engine, a computational tool for unbiased assessment of chemoproteomic probes^42^. After incubation of the probes with MDA-MB-231 cell lysates, the proteome were digested and subjected to click-reaction with Heavy or Light azido-UV-biotin, respectively, and the samples were then combined and analyzed by LC-MS/MS and pChem. The results showed that *α*-acyloxyenamide warhead (A1/A2/A3) predominantly targets lysine residues (Supplementary Fig. 10), demonstrating excellent amino acid selectivity. We then proceeded to identify the labeled lysine residues in live cells. MDA-MB-231 cells were incubated with probe A1 followed by cell lysis. The resulting samples were conjugated with UV-cleavable PC-biotin-N_3_ (Supplementary Table S1) followed by affinity purification, and the probe-labeled peptides were then released by UV irradiation and tested by LC−MS/MS. A total of 122 lysine residues were detected with a molecular weight increase of A1 (Fig. 4g, h and Supplementary Fig. 29, Table S5 in Supplementary Data 1). These reveal a series of functional residues, such as K12 of nucleoside diphosphate kinase A (NDKA), K446 of oxysterol-binding protein-related protein 5 (OSBL5), which are annotated binding sites or active sites by searching uniprot database. Mutation of PFKAP K688 in the protein binding pocket to K688R severely affects the probe labeling (Fig. 4i, lower gel), indicating that the protein labeling indeed occurs at K688. Knockdown of PFKAP decreases the labeling bands, demonstrating that the major labeling band is PFKAP protein (Fig. 4j and Supplementary Fig. 11). Docking experiments showed that the probe **A1** is accommodated well in the binding pocket of PFKAP near the K688 site (Fig. 4k). These results demonstrated that the probe can covalently modify a series of significant proteins and could modulate the protein function, and **A1** can serve as a useful tool for specific detection of the expression and activity of PFK protein in live cells at low probe concentrations.

### The performance of the reactive group in kinase inhibitors

**Fig. 5:**
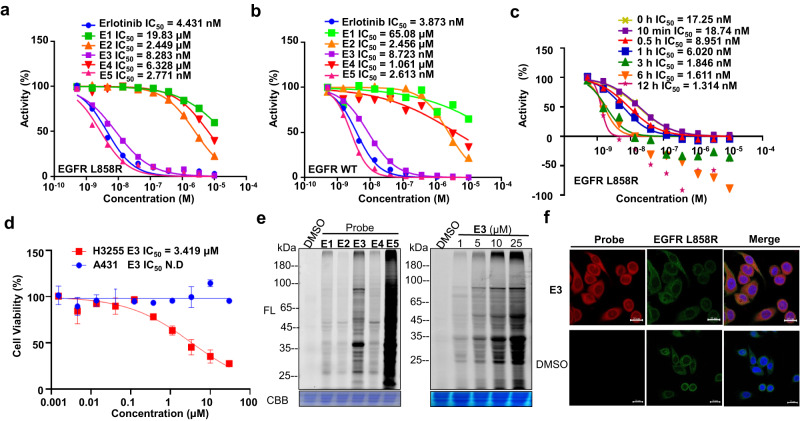
The activity of erlotinib-based probes. **a** Inhibitory activity of Erlotinib, **E1**, **E2**, **E3**, **E4** and **E5** against EGFR L858R mutants. **b** Inhibitory activity of Erlotinib, **E1**, **E2**, **E3**, **E4** and **E5** against EGFR WT. **c** Time-dependent inhibition assay of **E3** against EGFR L858R. **d** Cellular inhibition assay of **E3** against H3255 cancer cells and A431 cancer cells. **e** Proteome reactivity profiles of **E1**, **E2**, **E3**, **E4**, and **E5** (10 μM) with H3255 cells (left gel). Concentration-dependent labeling profiles of **E3** (2 h incubation) with H3255 cells (right gel). FL = in-gel fluorescence scanning. CBB = Coomassie gel. **f** Cellular imaging of **E3** (10 μM) and immunofluorescence (IF) in H3255 live cells. scale bar = 20 μm, DMSO-treated samples were used as controls.

The inhibitory activity of **E1**, **E2**, **E3**, **E4** or **E5** against kinases was first examined using erlotinib as a positive control. As shown in Fig. 5a, the probes **E3** and **E5** with an electrophile at the hydrophilic moiety, showed comparable to that of erlotinib against EGFR L858R mutant. A similar trend was observed in the inhibition against EGFR WT (Fig. 5b). In contrast, the probes produced by coupling with the aliphatic ynamide at the same position (**E4**), showed 1000-fold reduced potency against both EGFR L858R mutant and EGFR WT, demonstrating that the *α*-acyloxyenamide produced by aromatic ynamide supports retention of the inhibitory activity. This could be attributed to the stability of different *α*-acyloxyenamide warheads (Supplementary Fig. 1). The IC_50_ values of **E3** in inhibition of EGFR L858R activity improved from 18.7 nM (*t* = 10 min) to 1.3 nM (*t* = 12 h) as the drug-incubation time increased (Fig. 5c and Supplementary Fig. 13), supporting the covalent inactivation in this assay^10,11^. Weak inhibitory activity was observed from the probes modified at the recognition moiety, **E1** and **E2**, which could be accounted for by the steric hindrance of the electrophile on the probe binding. Consistently, **E3** and **E5** displayed the most potent antiproliferative activity against H3255 cells harboring EGFR L858R mutant, but not against the A431 cells harboring EGFR WT, which is consistent with the trend of positive control Erlotinib (Fig. 5d and Supplementary Fig. 14). These data demonstrated that the probes **E3** and **E5** possess potent inhibitory activity against EGFR L858R under both in vitro and in situ conditions. After confirmation of the inhibitory activity, proteome labeling experiments were carried out under live-cell conditions. Using procedures similar to those described above, **E3** and **E5** were found to display higher labeling capability in H3255 cells toward endogenous proteins than other probes (Fig. 5e, left gel), which is consistent with the trend of the inhibition assays. Multiple fluorescence bands were detected throughout the whole lane, indicating that off-targets probably exist in addition to the EGFR L858R. Concentration-dependent labeling experiments showed that **E3** probe at a 1 µM probe concentration can produce notable labeling bands (Fig. 5e, right gel, Supplementary Fig. 12), and immunofluorescence experiments showed that the fluorescence signal of **E3** can cover the location of target protein EGFR L858R in H3255 cancer cells (Fig. 5f and Supplementary Fig. 15). These data demonstrate that the probes **E3** and **E5** preserved the bioactivity of the parent compound and possess labeling capability.

**Fig. 6:**
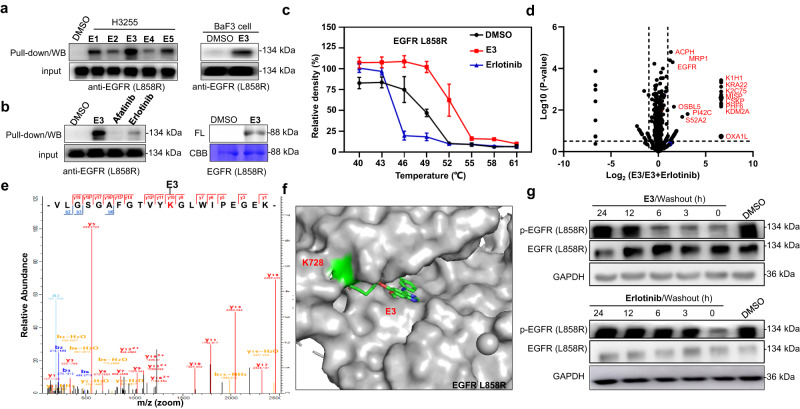
E3 probe covalently binds to EGFR L858R. **a** Pull-down/WB validation of EGFR L858R in H3255 cells with **E1**, **E2**, **E3**, **E4** and **E5** (10 μM) (left); Pull-down/WB validation of EGFR L858R in BaF3 cells with **E3** (10 μM) (right). EGFR antibodies were used in western blots. **b** Competitive labeling of endogenous EGFR (L858R) with H3255 live cells in the presence of afatinib or erlotinib (5×) as a competitor (left). Labeling of EGFR L858R protein (0.1 µg/µL) with **E3** (10 μM) (right). FL = in-gel fluorescence scanning. CBB = Coomassie gel. **c** Cellular thermal shift assay performed in H3255 cells with **E3** (10 μM), and EGFR L858R kinase is the target protein. **d** Mass spectrometry-based profiling of **E3**/(**E3**+1×Erlotinib) binding proteins (10 μM probe concentration) from H3255 cells. **e** Mass spectrum of binding site of EGFR L858R at K728 modified by **E3**, identified modification site by MS2 spectra was highlighted (red). **f** Docking experiment to predict the binding mode of **E3** with EGFR L858R (PDB code: 4LQM). **g** H3255 cells were treated with **E3** or Erlotinib (10 μM) for 2 h, followed by compound wash-out for the indicated time. Effect on the phosphorylation level of EGFR was tested.

To assess the target engagement capability of the probes, validation experiments were carried out. Pull-down/WB experiments showed that the probes **E1**, **E2**, **E3**, **E4**, and **E5** can successfully label EGFR L858R in H3255 cells, and the potent probes **E3**, **E5** exerted higher labeling capability than the others (Fig. 6a, left gel, and Supplementary Fig. 16). **E3** successfully labeled EGFR L858R from BaF3 cancer cells by pull-down/WB experiments using corresponding antibodies (Fig. 6a, right gel, and Supplementary Fig. 17). Pretreatment of 5 equivalents of afatinib or erlotinib significantly reduced or eliminated the labeling of EGFR L858R, demonstrating that the probe **E3** shares the same binding model as the parent molecules in live cells (Fig. 6b, left gel). Labeling with recombinant protein displayed that **E3** probe can successfully label the EGFR L858R protein (Fig. 6b, right gel). A thermal shift assay demonstrated **E3** can stabilize EGFR L858R protein when the temperature increases, which is a reverse trend from the behavior of the non-covalent erlotinib (Fig. 6c and Supplementary Fig. 20). Competitive pull-down/LC-MS/MS with H3255 cancer cells in the presence of Erlotinib revealed a series of off-targets including ACPH, MRP1 and CSKP in addition to EGFR L858R (Fig. 6d, Tables S6, S7 in Supplementary Data 1), implying that the drug activity of erlotinib might be attributed to the combination of these protein hits (Supplementary Fig. 18). Further evidence of this covalent interaction was obtained by binding site mapping of the whole cell lysate, which revealed that **E3** can covalently bind to K728 of EGFR L858R proximal to the ATP binding pocket (Fig. 6e and Table S9 in Supplementary Data 1). Docking experiments showed that probe **E3** adopted a conformation identical to that of erlotinib, and occupied the ATP binding pocket of EGFR L858R by covalent conjugation at K728 (Fig. 6f). Signaling pathway validation experiments proved that the **E3** probe downregulated EGFR phosphorylation (Fig. 6g/Supplementary Fig. 19), and this activity was not recovered in up to 6 h wash out experiments. In contrast, the activity of non-covalent Erlotinib can not be retained under washout conditions (Fig. 6g). Together, these data demonstrate that probe **E3** covalently engages the EGFR L858R mutant under in situ conditions, and K728 is a conserved lysine residue for the development of new targeted covalent inhibitors.

**Fig. 7:**
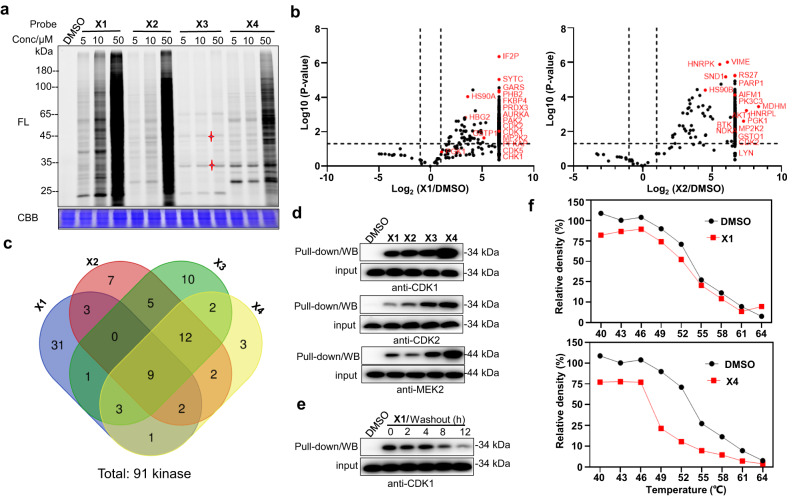
Proteome reactivity profiles of α-acyloxyenamide-based pan-kinase inhibitor. **a** Concentration-dependent labeling profiles of **X1**, **X2**, **X3**, and **X4** (10 μM) with K562 cells. FL = in-gel fluorescence scanning. CBB = Coomassie gel. **b** MS-spectrometry-based profiling of **X1** and **X2** binding proteins (10 μM), DMSO-treated sample was used as a negative control, the data of **X3** and **X4** were presented in Fig. S22 (Supporting information). **c** Venn diagram explains the coincidence and difference of **X1**, **X2**, **X3**, and **X4** kinase targets. **d** Target validation by pull-down, WB with **X1**, **X2**, **X3**, and **X4** and the corresponding antibodies. **e** K562 cells were treated with **X1** (10 μM, 2 h), followed by compound washout for the indicated times. Pull-down/WB validation with a CDK1 antibody. **f** Cellular thermal shift assay performed in K562 cells with **X1** and **X4** (10 μM), and CDK1 kinase is the target protein.

To quantify the kinase engagement of probes **X1**, **X2**, **X3**, and **X4** based on a pan-kinase inhibitor, proteome reactivity profiles were carried out. After treatment of K562 cells with the probes for 2 h, the cells were lysed and subjected to TAMRA-N_3_. Similar to the trend of erlotinib-based probes, **X1** generated from aromatic ynamide produced higher fluorescence intensity than the other probes (Fig. 7a and Supplementary Fig. 21) and the labeling profiles of aliphatic *α*-acyloxyenamide containing probe, **X3**, were severely influenced. Remarkably, distinct labeling bands were observed with **X3** and **X4** probes at lower probe concentrations (* marked bands), showing excellent selectivity. To identify the probe-labeled proteins, pull-down/LC-MS was carried out. K562 cells were incubated with probes for 2 h, and this was followed by the procedures described above. A total of 91 different kinases were detected by these probes together at a low probe concentration (10 µM), and 50, 40, 42, and 34 protein kinases were labeled by probe **X1**, **X2**, **X3** and **X4**, respectively, demonstrating high labeling capability toward kinases (Fig. 7b and Supplementary Fig. 22, Tables S10, S11, S12 and S13 in Supplementary Data 1). Most kinases were simultaneously detected by at least two probes and ∼20% of the kinases were identified by four probes, which could be attributed to the same recognition scaffold and high reproducibility (Fig. 7c). Analysis of these protein hits showed that cyclin-dependent kinase 1 (CDK1), dual specificity mitogen-activated protein kinase kinase 2 (MEK2) and glutathione S-transferase P (GSTP1) correspond to the ~35 kDa, ~45 kDa and ~23 kDa labeling bands, respectively. Other CDK family kinases, CDK2, CDK4, CDK5, CDK6, CDK7, and CDK9, were also detected, indicating that these probes preferentially label CDK kinases. CDK1 promotes G2-M transition and regulates G1 progress and G1-S transition by association with multiple interphase cyclins^43^. MEK2 can activate the extracellular signal-regulated kinase (ERK) and mitogen-activated protein (MAP) kinase upon binding of an agonist to a receptor^44^, these are critical targets for drug development and cancer therapy. Other identified kinases include BTK, AURKA/B, PAK2, VRK1, YES, PKN1, GSK3A. Further validation of CDK1, CDK2, and MEK2 was established by pull-down/WB with **X1**, **X2**, **X3** or **X4** using the corresponding antibodies (Fig. 7d and Supplementary Fig. 23), which substantially persisted after 4 h washing time (Fig. 7e). Probe binding can destabilize the CDK1 protein upon temperature increase, as revealed by a thermal shift assay (Fig. 7f and Supplementary Fig. 25). These results demonstrated that the probes can covalently label a series of essential kinases in live cells.

**Fig. 8:**
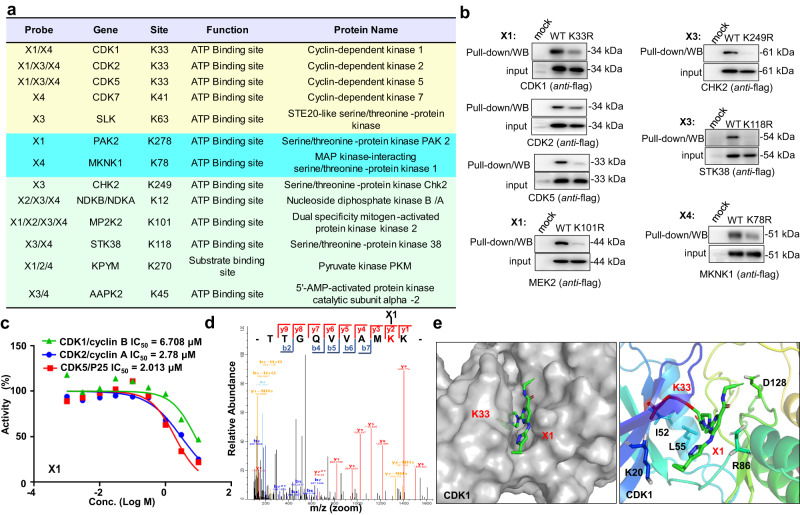
Modification site analysis of X1, X2, X3, and X4 in whole proteome. **a** Subset of residues identified by **X1**, **X2**, **X3**, or **X4** (100 μM) with K562 cell lysates. **b** Pull-down/WB of wild type and mutant proteins with **X1**, **X3**, and **X4** (10 μM); The western blots were confirmed by using flag antibodies. **c** Inhibitory activity of **X1** against CDK1, CDK2, CDK5. **d** Mass spectrum of modification site of CDK1 at K33 by **X1**, identified modification site by MS2 spectra was highlighted (red). **e** Docking experiment to predict the binding mode of **X1** with CDK1 (PDB code: 4Y72).

To evaluate if the probes could be used in profiling the conserved residues of kinase in whole proteome, binding site analysis was carried out in cell lysates using the aforementioned procedures. A total of 64 lysine residues from different kinases were detected, and 28, 25, 17, 35 sites were detected by **X1**, **X2**, **X3**, and **X4**, respectively (Tables S14/S15/S16/S17 in Supplementary Data 1). Amongst the sites identified, K33 of CDK1, CDK2, CDK5; K278 of PAK2; K78 of MKNK1; K97, K101 of MP2K2, MP2K1; K12 of NDKB; K45 and K56 of AAPK1, AAPK2 and K68, K69 of CSK21, CSK22, CSK23 are annotated ATP binding sites. K270 of KPYM are the annotated substrate binding site (Fig. 8a and Supplementary Fig. 28, Tables S14/S15/S16/S17 in Supplementary Data 1). A small portion of identified sites, for example K216 of PGK1, or K20 of CDK1 were found to be located in the ATP binding pocket with no annotated functions, indicating that these might be covalently modifiable sites and affect the kinase function. Mutation of the catalytic K33 of CDK1, CDK2, CDK5; K101 of MEK2; K249 of CHK2; K118 of STK38; K78 of MKNK1 to arginine leads to a decreased labeling profile of the probe with the corresponding kinases (Fig. 8b), demonstrating that the modification occurs mainly at the catalytic residues. A kinase inhibition assay proved that probe **X1** exhibited moderate inhibitory activity against CDK1, CDK2, and CDK5 with IC_50_ values of 2–6 µM (Fig. 8c). Mass spectrometry showed that the modification of **X1** with CDK1 indeed occurs at K33 (Fig. 8d), and docking experiments proved that the probe can bind to the ATP pocket proximal to the K33 site (Fig. 8e and Supplementary Fig. 24). These results revealed the covalent engagement of these conserved residues with annotated or unannotated functions, and this could be a useful clue for the development of new types of covalent inhibitors. Taken together, these results demonstrate that the *α*-acyloxyenamide-based probes are suitable for profiling ligandable conserved residues of kinases in the whole proteome.

### The performance of α-acyloxyenamide reactive group in natural products

**Fig. 9:**
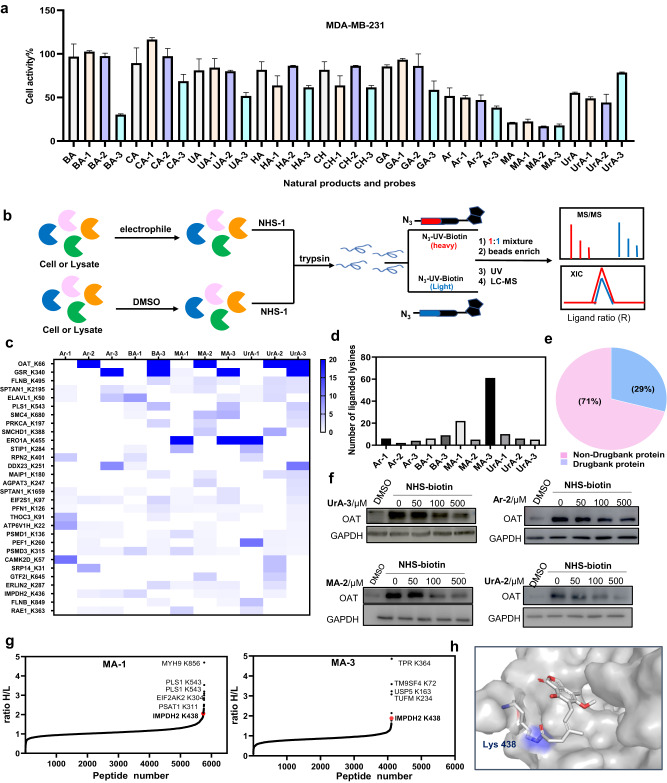
Evaluation of the performance of α-acyloxyenamide- and NHS-based probes derived from natural products. **a** Antiproliferation activity was tested against MDA-MB-231 cells with natural products and electrophilic probes. **b** General process to identify the binding sites of natural product-based probes in live cells or cell lysates by using light and heavy reagents (iso-TOP) and LC-MS/MS, DMSO- (heavy, or red) versus fragment-treated (light, or bule) samples. *R* value ≥4 was used to define a fragment binding toward a quantified lysine. **c** Heat map presents the liganded targets by natural product-based probes bearing various electrophilic warheads, and R_H/L_ ≥4 sites are considered to be targets of natural product ligands (Data processing is to take the mean value of two or more groups of R, and CV ≤ 40 was used as a cutoff value). **d** Plot comparison of the number of lysine residues for each probe. **e** Drugbank database analysis of protein hits identified by the different electrophilic probes (29% are druggable proteins and 71% are undruggable proteins). **f** Competitive labeling of OAT target by pull-down/WB experiment with NHS-biotin probe (100 µM) in the presence of Ar-2, MA-2, UrA-2, UrA-3. **g** Protein hits and binding sites of **MA-1/3** identified from MDA-MB-231 breast cancer cells by iso-TOP experiments, red-colored IMPDH2 is a known target of mycophenolic acid, K438 is the binding site by both covalent probes MA-1 and MA-3. MYH9 K856, TPR K364 are identified unknown targets. **h** Docking experiments to predict the binding mode of MA-3 with IMPDH2 at K438 (PDB code: 6U8E). All error bars represent standard deviation (*n* = 3), there is no statistical significance between the samples (*p* > 0.1).

To evaluate the performance of the *α*-acyloxyenamide electrophile in natural products, further validation experiments were carried out. Firstly, antiproliferation activity of these probes against MDA-MB-231 cells was evaluated with a CCK8 assay and the original natural products were tested concurrently. As shown in Fig. 9a, the results displayed that *α*-acyloxyenamide containing probes exerted higher potency than NHS-containing probes, suggesting that the *α*-acyloxyenamide moiety could improve the anticancer effects of the probes. Subsequently, quantitative proteomics analysis was carried out with the probes containing NHS and *α*-acyloxyenamide electrophiles to identify conserved lysine residues (Figs. 2d, 9b)^45^. The compounds, **Ar-1/2/3**, **BA-1/3**, **MA-1/2/3**, **UrA-1/2/3**, with potent anticancer effects were selected for binding site mapping. The NHS- and *α*-acyloxyenamide-containing probes or DMSO were incubated with MDA-MB-231 cell lysates, followed by incubation with alkyne-containing tool probe **NHS-1**. The samples were digested with trypsin, and then clicked with light or heavy UV cleavable biotin-azide under click chemistry conditions^16,42^. The probe labeled peptides were enriched by streptavidin agarose beads and released by UV irradiation and identified by LC-MS/MS. As shown in Fig. 9c and Table S18 in Supplementary Data 1, heat map presents the target engagement of various electrophilic warheads, and the protein hits with *R* value ≥4 were defined as a fragment engagement of identified lysine residues. The top two labeled sites include K66 of ornithine aminotransferase (OAT) and K340 of the glutathione reductase (GSR), and the *α*-acyloxyenamide based probes (**Ar-2/3**, **BA-3**, **MA-2/3**, **UrA-2/3**) produced higher isotopic ratios than NHS-based probes (**Ar-1**, **BA-1**, **MA-1**, **UrA-1**), showing excellent labeling capability toward these conserved lysine residues. Different subsets of liganded lysine residues were detected from these probes, and **MA-3** exerted highest number of modification (Fig. 9d). Up to 71% of protein hits are undruggable targets based on drugbank database analysis (Fig. 9e). Observed that K66 of ornithine aminotransferase (OAT) is located in a binding pocket and this protein is related to autosomal recessive disease^46^, further validation experiments were carried out. Competitive labeling profiles with NHS-biotin showed that **Ar-2,** **MA-2,** **UrA-2,** **UrA-3** dose-dependently block the labeling profiles, demonstrating that these probes indeed bind to ornithine aminotransferase protein (Fig. 9f). Binding site mapping with **MA-1/3** in MDA-MB-231 live cells revealed that the known target IMPDH2 can be successfully identified by both probes (Fig. 9g)^32^, and the labeled site K438 was found to be an adjacent residue in the binding pocket (Fig. 9h). Notably, a suite of different protein hits and lysine residues such as K856 of MYH9, K364 of TPR were detected by these two probes (Fig. 9g, Tables S19, 20 in Supplementary Data 1), demonstrating complementary utility of these electrophilic warheads in profiling of ligandable lysine residues. These results again demonstrated that the *α*-acyloxyenamide electrophiles can be applied in various bioactive molecule for profiling known or unknown targets and the corresponding ligandable conserved lysine residues, thus facilitating the development of new targeted covalent inhibitors.

## Conclusion

In conclusion, our study introduces a straightforward one-step coupling reaction that transforms a carboxylic acid group in a bioactive molecule into a highly reactive electrophile, an *α*-acyloxyenamide, at a late-stage of synthesis. This process is compatible with various functional groups in a pharmacophore, allowing for broad applications. The resulting electrophile exhibits remarkable reactivity toward lysine residues in proteins. By incorporation of this potent electrophile into a specific kinase inhibitor, erlotinib, and a promiscuous kinase inhibitor, we successfully developed probes capable of covalently engaging proteins of interest. A series of essential conserved lysine residues of different kinases were identified, providing valuable clues for the development of new targeted covalent inhibitors. Moreover, the utility of this method extends to natural products, where it enabled target profiling and binding pattern characterization of unknown targets, and can be complementary to NHS warhead. The ability to study the interactions of α-acyloxyenamide-containing probes with various protein targets opens new avenues for understanding their biological activities. These findings demonstrate the versatility and utility of α-acyloxyenamide electrophiles in bioactive molecules for studying target engagement and profiling conserved lysine residues.

## Methods

### General procedures

All reactions were carried out under an atmosphere of argon in oven-dried glassware with a magnetic stirring bar. All reactions were monitored by thin layer chromatography (TLC), which were visualized with ultraviolet light and then developed with basic potassium permanganate solution. Flash column chromatography was carried out using silica gel (HAIYANG, 200–300 mesh). All NMR spectra (^1^H-NMR, ^13^C-NMR) were recorded on BrukerAvance ARX- 400. Chemical shifts were reported in parts per million (ppm) referenced with respect to appropriate internal standards or residual solvent peaks (CDCl_3_ = 7.26 ppm, DMSO-d6 = 2.50 ppm). The following abbreviations were used in reporting spectra, br s (broad singlet), s (singlet), d (doublet), t (triplet), q (quartet), m (multiplet), dd (doublet of doublets). Mass spectra were obtained on Agilent LC-ESI-MS system.

### Cell culture and western blot

Cell lines were obtained from the National Cancer Institute Developmental Therapeutics Program (NCI-60). Cells were cultured in Dulbeccoʼs modified Eagle medium (DMEM; Invitrogen, Carlsbad, CA) or RPMI 1640 Medium (Invitrogen, Carlsbad, CA) containing 10% heat-inactivated fetal bovine serum (FBS; Invitrogen), 100 units/mL penicillin, and 100 μg/mL streptomycin (Thermo Scientific) and maintained in a humidified 37 °C incubator with 5% CO_2_. To generate protein lysates, cells were washed twice with cold phosphate-buffered saline (PBS), harvested with 1× trypsin or by use of a cell scraper, and collected by centrifugation. Cell pellets were then washed with PBS and lysed with RIPA (Thermo ScientificTM, #89900) lysis and extraction buffer (with PierceTM Protease Inhibitor Tablets, Thermo ScientificTM, #A32955). Protein concentration was determined by PierceTM BCA Protein Assay Kit (Thermo Scientific TM, #23252) and Synergy H1 Hybrid Multi-Mode Reader (BioTek). PFKAP antibodies (ab119796), CDK1 antibodies (ab133327), CDK2 antibodies (ab32147), MEK2 antibodies (ab32517) were purchased from abcam company, GAPDH antibodies (AG019-1) was purchased from Beyotime, anti-flag (14793S) and EGFR antibodies (2232S) were purchased from Cell Signaling Technology. For Western blotting experiments, samples were resolved by SDS−polyacrylamide gels and transferred to poly membranes. Membranes were then blocked with 5% bovine serum albumin (BSA) in TBST (0.1% Tween in Tris-buffered saline) for 1 h at room temperature. After blocking, membranes were incubated with the corresponding primary antibody overnight at 4 °C. After incubation, membranes were washed with TBST (4 × 10 min) and then incubated with an appropriate secondary antibody. Finally, blots were washed again with TBST before being developed with SuperSignal West Dura Kit (Thermo Scientific), and finally imaged with Amersham Imager 600 (GE Healthcare). Cell Counting Kit-8 (CCK-8, DOJINDO, #CK04) was used for cell proliferation assay.

### In situ and in vitro proteome labeling

For labeling of pure protein, 1 mg/mL protein in PBS buffer was treated with probe (A1/A2/A3/A4/NHS-1/2). The mixture was incubated for 3 h at 37 °C with gentle shaking, and then clicked with TAMRA-N_3_. The labeled protein was separated by SDS−PAGE and visualized by in-gel fluorescence scanning (Typhoon FLA 9500). Time- and concentration-dependent experiments were performed with different concentrations of the probes and different incubation time.

For in situ proteome labeling with live cells, cells were grown to 80−90% confluency in six-well plates under conditions as described above. The medium was removed and washed twice with PBS and then treated with 2 mL probe-containing fresh medium in the presence or absence of competitors (diluted from DMSO stocks whereby DMSO never exceeded 1% in the final solution). After incubation for 2–4 h, the medium was aspirated and cells were washed twice with PBS to remove excessive probes. The cells were treated with RIPA lysis buffer containing 2.5% chaps and phosphatase inhibitor (Thermo ScientificTM #88669), the suspended cells were lysed by sonication (14 × 3 s with 5 s breaks, 60% power) on ice. A soluble protein solution was obtained by centrifugation for 20 min (14,000 rpm, 4 °C). Eventually, the protein concentrations were determined by using the BCA protein assay (PierceTM BCA protein assay kit) and diluted to 1 mg/mL with PBS. A freshly pre-mixed click chemistry reaction cocktail was added (100 μM TAMRA-N_3_ from 10 mM stock solution in DMSO, 100 μM TBTA from 10 mM freshly prepared stock solution in DMSO, 1 mM TCEP from 100 mM freshly prepared stock solution in deionized water, and 1 mM CuSO_4_ from 100 mM freshly prepared stock solution in deionized water). The reaction was further incubated for 2 h prior to the addition of pre-chilled methanol (−20 °C). The precipitated proteins were subsequently collected by centrifugation (14,000 rpm, 10 min at 4 °C), and washed twice with 1 mL of prechilled methanol. The samples were dissolved in 1 × SDS loading buffer and heated for 15 min at 95 °C. 50 μg proteins for each lane were loaded on SDS−PAGE (10% or 8% gel) and then visualized by in-gel fluorescence scanning (Typhoon FLA 9500).

For labeling of MDA-MB-231 cell lysates with **A1**–**A4**, probes were incubated with 1 mg/mL protein in PBS buffer, the mixture was incubated for 4 h, clicked with TAMRA-N_3_ under standard click chemistry conditions (100 μM TAMRA-N_3_ from 10 mM stock solution in DMSO, 100 μM TBTA from 10 mM freshly prepared stock solution in DMSO, 1 mM TCEP from 100 mM freshly prepared stock solution in deionized water, and 1 mM CuSO_4_ from 100 mM freshly prepared stock solution in deionized water). After 2 h of click reaction, 1 mL of pre-cooled methanol was added. The precipitated proteins were subsequently collected by centrifugation (14,000 rpm, 10 min at 4 °C), and washed twice with 1 mL of prechilled methanol. The samples were dissolved in 1 × SDS loading buffer and heated for 15 min at 95 °C. The resulting proteins were resolved by SDS-PAGE. In-gel fluorescence scanning was used to visualize the labeled protein bands. Both in-gel fluorescence scanning (FL) and coomassie staining (CBB) were always carried out on the gels upon SDS-PAGE separation of labeled samples.

### Cellular imaging and immunofluorescence

To demonstrate the utility of the cell-permeable probes for imaging of cellular targets, we performed fluorescence microscopy. The general procedures were similar to what was previously reported^12,13^. For fixed cells, MDA-MB-231 cells or H3255 cells were seeded in glass bottom dishes (Mattek) and grown until 70−80% confluency. Cells were treated with 0.2 mL of 1640 with a probe (**A1**/**A2**/**A3** or **E3**) or DMSO at different indicated concentrations. After incubation for 2–4 h, the medium was removed and cells were gently washed twice with PBS. The cells were subsequently fixed for 2 h at room temperature with 3.7% formaldehyde in PBS, washed twice with cold PBS, and permeabilized with 0.1% Triton X-100 in PBS for 1h. Cells were then treated with a freshly premixed click chemistry reaction (50 μM TAMRA-N_3_ from 2.5 mM stock solution in DMSO, 0.1 mM TBTA from 2.5 mM freshly prepared stock solution in deionized water, 1 mM TCEP from 25 mM freshly prepared stock solution in deionized water, and 1 mM CuSO_4_ from 25 mM freshly prepared stock solution in deionized water) for 2 h at room temperature with vigorous shaking. Cells were washed with PBS three times and 0.1% Tween 20 in PBS once. Finally, the cells were stained with DAPI for 10 min at room temperature prior to image. DMSO-treated samples were used as controls concurrently.

For co-localization experiments, cells were further incubated with anti-EGFR (Cell Signaling Technology TM, 2232S) (1:500) for 1 h at room temperature (or overnight at 4°C), washed twice with 0.1% Tween 20 in PBS, and then incubated with Goat Anti-Rabbit IgG H&L (Alexa Fluor® 488) (1:200) for 1 h, followed by washing again. Imaging was done with the Zeiss LSM800 confocal microscope system equipped with Pln Apo 40 ×/1.3 Oil DICII, 405 nm diode laser, white laser (470−670 nm, with 1 nm increments, with eight channels AOTF for simultaneous control of eight laser lines, each excitation wavelength provides 1.5 mV), and a photomultipliertube (PMT) detector ranging from 410 to 700 nm for steady state fluorescence.

### Kinase inhibition assay

The probes were evaluated with the EGFR kinase inhibition using Z’-LYTE™ fluorescence resonance energy transfer (FRET) method, parent inhibitors were used as the reference compounds. The Z’-LYTE™ biochemical assay employs a FRET-based, coupled-enzyme format and is based on the differential sensitivity of phosphorylated and non-phosphorylated peptides to proteolytic cleavage. The peptide substrate is labeled with two fluorophores-one at each end-that make up a FRET pair. The compounds were diluted three-fold from 5.1 × 10^−9^ M to 1 × 10^−4^ M in DMSO. Plate was measured on EnVision Multilabel Reader (Perkin Elmer). Curve fitting and data presentations were performed using Graph Pad Prism version 4.0.

Probe X1 was evaluated with the kinase inhibition using Enzymatic Radiometric Assay by Europhin company. CDK1/cyclinB (h)/CDK2/cyclinA (h)/CDK5/p25 (h) was incubated with 8 mM MOPS pH 7.0, 0.2 mM EDTA, 0.1 mg/mL histone H1, 10 mM Mg(OAc)_2_ and [gamma-33P]-ATP (specific activity and concentration as required). The reaction is initiated by the addition of the Mg/ATP mix. After incubation for 40 min at room temperature, the reaction is stopped by the addition of phosphoric acid to a concentration of 0.5%. An aliquot of the reaction is then spotted onto a filter and washed four times for 4 min in 0.425% phosphoric acid and once in methanol prior to drying and scintillation counting.

### IC_50_ values of the probes derived from kinase inhibitors and natural products against different cancer cells

Cell growth inhibition assay was carried out using MDA-MB-231, Ba/F3, H3255 and A431 cancer cells and cell viability was determined by CCK8 assay. 4000 cells per well were seeded in a 96-well plate and incubated for 24 h in a humidified incubator for adherence. A1/A2/A3/A4 (0 μM to 100 μM) or E1/2/3/4/5 and Erlotinib (0–30 μM) were dissolved in DMSO and added to each well while maintaining a final DMSO concentration of 0.1%. After incubation for 72 h, 15 μL of CCK8 reagent was added to each well and incubated for 2 h. After that, the absorbance was measured at 450 nm and 650 nm on a plate reader (Synergy HI, BioTek Instruments, Inc. Vermont, US). Cell viability rate was determined as VR = (A – A0)/(As–A0) × 100%, where A is the absorbance of the experimental group, As is the absorbance of the control group (DMSO was used as the control) and A0 is the absorbance of the blank group (no cells). IC_50_ values were calculated using Graphpad Prism. For antiproliferation activity of natural products, cell growth inhibition assay was carried out using MDA-MB-231 cells. Natural products and probes (50 μM) were dissolved in DMSO and added to each well while maintaining a final DMSO concentration of 0.1%. After incubation for 72 h, 15 μL of CCK8 reagent was added to each well and incubated for 2 h and tested.

### Target identification by pull-down-LC-MS/MS

The cells were treated with probes for 2–4 h, DMSO-treated samples were used as negative controls. Upon cell lysis, the protein concentrations were determined by using the BCA protein assay (PierceTM BCA protein assay kit) and diluted to 1 mg/mL with PBS. A freshly pre-mixed click chemistry reaction cocktail was added (100 μM biotin-N_3_ from 10 mM stock solution in DMSO, 100 μM TBTA from 10 mM freshly prepared stock solution in DMSO, 2 mM sodium ascorbate from 200 mM freshly prepared stock solution in deionized water, and 1 mM CuSO_4_ from 100 mM freshly prepared stock solution in deionized water). The reaction was further incubated for 2 h prior to the addition of pre-chilled MeOH at −20 °C. Precipitated proteins were subsequently collected by centrifugation (14,000 rpm × 10 min at 4 °C) and dissolved in PBS containing 1% SDS. The probe labeled proteins were enriched by PierceTM Streptavidin Plus UltraLinkTM Resin and digested for 4 h at r.t., the beads were washed with PBS containing 1% SDS (thrice), PBS containing 0.1% SDS (once) and PBS (thrice). The beads were suspended in 500 μL 6 M urea in PBS, 25 μL of 200 mM DTT in 25 mM NH_4_HCO_3_ buffer was added and the reaction was incubated for 37 °C for 30 min. For alkylation, 25 μL of 400 mM IAA in 25 mM NH_4_HCO_3_ buffer was added followed by incubation for 1h at r.t. in dark. The supernatant was then removed and the beads were washed with 1 mL PBS (once). For the digestion, 150 μL 2 M urea in PBS,1 mM CaCl_2_ in 50 mM NH_4_HCO_3_ and 5 μL trypsin (1 μg/μL) were added at a ratio of 1:200, and the reaction was incubated at 37 °C overnight. The supernatants containing the digested peptides were collected, desalted with Waters C18 Tips and dried by vacuum centrifugation.

For target protein identification of E3, the H3255 cells were treated with DMSO, A5 (10 µM) and Erlotinib (10 µM) at 37 °C for 1 h and then treated with E3 (10 µM) at 37 °C for 2 h. DMSO-treated samples were used as negative controls. Upon cell lysis, the protein concentrations were determined by using the BCA protein assay (PierceTM BCA protein assay kit) and diluted to 1 mg/mL with PBS.

### Modification site analysis of A1/A2/A3 with model proteins

A1/A2/A3 (100 μM) was incubated with 150 μL (1 mg/mL) of three model proteins in PBS for 4 h. The excessive probe was removed through centrifugal filter (12,500 × *g* × 5 min at 4 °C) and washed with TEAB buffer for 6 times. The mixture was resuspended in 150 μL TEAB buffer. 1.5 μL of 1 M DTT in PBS was added and the reaction was incubated at 37 °C for 45 min. For alkylation, 3.0 μL of IAA (1 M) in PBS was added followed by incubation for 1 h at r.t. in dark. The excess IAA was neutralized by adding 1.5 μL of DTT (1 M) in PBS and removed by centrifugal filter (12,500 × *g* × 5 min at 4 °C), followed by washing with water for 3 times. For the digestion, 150 μL TEAB buffer and 3 μL of trypsin (1.0 μg/μL) were added. The resulting mixture was incubated at 37 °C overnight. Peptides were collected by washing with 200 μL water for 3 times, and then desalted by C18 column. After evaporation in speedvac, the samples were analyzed by LC-MS/MS.

### Modification site analysis of probes with cell lysates

E3 and X1/2/3/4 (100 μM) was incubated with 1 mL (1 mg/mL) of BaF3-EGFR-L858R and K562 cell lysates, respectively, in PBS for 2 h. The excessive probes were removed through centrifugal filter (12,500 × *g* × 5 min at 4 °C) and washed with water for 6 times. The mixture was resuspended in 1 mL water. A freshly pre-mixed click chemistry reaction cocktail was added (100 μM PC-biotin-N_3_ from 10 mM stock solution in DMSO, 100 μM TBTA from 10 mM freshly prepared stock solution in DMSO, 2 mM sodium ascorbate from 200 mM freshly prepared stock solution in deionized water, and 1 mM CuSO_4_ from 100 mM freshly prepared stock solution in deionized water). The reaction was further incubated for 2 h, followed by the addition of 1 mL of pre-cooled methanol and stored at −20 °C overnight. Precipitated proteins were subsequently collected by centrifugation (14,000 rpm × 10 min at 4 °C) and dissolved in PBS containing 1% SDS. Upon incubation with streptavidin beads for 4 h at r.t., the beads were washed with PBS containing 1% SDS (thrice), PBS containing 0.1% SDS (once) and PBS (thrice). The beads were suspended in 500 μL 6 M urea in PBS, 25 μL of 200 mM DTT in 25 mM NH_4_HCO_3_ buffer was added and the reaction was incubated for 37 °C for 30 min. For alkylation, 25 μL of 400 mM IAA in 25 mM NH_4_HCO_3_ buffer was added followed by incubation for 1 h at r.t. in dark. The supernatant was then removed and the beads were washed with 1 mL PBS (once). For the digestion, 150 μL 2 M urea in PBS, 1 mM CaCl_2_ in 50 mM NH_4_HCO_3_ and 5 μL trypsin (1 μg/μL) were added at a ratio of 1:200, and the reaction was incubated at 37 °C overnight. The beads were washed with water for 6 times to remove the unmodified peptides. The beads were then incubated with 500 μL 0.1% formic acid under 365 nm UV light for 1 h to release the probe modified peptides. After spin down at 1000 rpm for 5 min, the resulting probe modified peptides were collected. The beads were washed with 60% ACN, the washing buffer were combined and then desalted by C18 column. After evaporation in speedvac, the samples were analyzed by LC-MS/MS.

### Modification site analysis of A1 with live cells

MDA-MB-231 cells were grown to 80−90% confluency in 10 mL dish under conditions described above. The medium was removed and washed twice with PBS and then treated with 10 mL probe-containing fresh medium (diluted from DMSO stocks whereby DMSO never exceeded 1% in the final solution). After 4 h of incubation, the medium was aspirated and cells were washed twice with PBS to remove excessive probes. The cells were treated with PBS containing 2.5% chaps and phosphatase inhibitor (Thermo ScientificTM #88669), the suspended cells were lysed with sonication (14 × 3 s with 5 s breaks, 15% power) on ice. A soluble protein solution was obtained by centrifugation for 10 min (14,000 rpm, 4 °C). Eventually, the protein concentrations were determined by using the BCA protein assay (PierceTM BCA protein assay kit) and diluted to 1 mg/mL with PBS and 1 mL proteome was used for the following experiments. A freshly pre-mixed click chemistry reaction cocktail was added (100 μM PC-biotin-N_3_ from 10 mM stock solution in DMSO, 100 μM TBTA from 10 mM freshly prepared stock solution in DMSO, 2 mM sodium ascorbate from 200 mM freshly prepared stock solution in deionized water, and 1 mM CuSO_4_ from 100 mM freshly prepared stock solution in deionized water). The reaction was further incubated for 2 h followed by addition of 10 mL pre-cooled methanol and incubated at −20 °C overnight. Precipitated proteins were subsequently collected by centrifugation (14,000 rpm × 10 min at 4 °C) and dissolved in PBS containing 1% SDS. Upon incubation with streptavidin beads for 4 h at r.t., the beads were washed with PBS containing 1% SDS (thrice), PBS containing 0.1% SDS (once) and PBS (thrice). The beads were suspended in 500 μL 6 M urea in PBS, 25 μL of 200 mM DTT in 25 mM NH_4_HCO_3_ buffer was added and the reaction was incubated at 37 °C for 30 min. For alkylation, 25 μL of 400 mM IAA in 25 mM NH_4_HCO_3_ buffer was added followed by incubation for 1 h at r.t. in dark. The supernatant was then removed and the beads were washed with 1 mL PBS (once). For the digestion, 150 μL 2 M urea in PBS, 1mM CaCl_2_ in 50 mM NH_4_HCO_3_ and 5 μL trypsin (1 μg/μL) were added at a ratio of 1:200, and the reaction was incubated at 37 °C overnight. The beads were washed with water for 6 times to remove the unmodified peptides. The beads were then incubated with 500 μL 0.1% formic acid under 365 nm UV light for 1 h to release the probe modified peptides. After spin down at 1000 rmp for 5 min, the resulting probe modified peptides were collected. The beads were washed with 60% ACN, the washing buffer were combined and then desalted by C18 column. After evaporation in speedvac, the samples were analyzed by LC-MS/MS.

### LC-MS/MS analysis

The raw data were processed by using MaxQuant software (1.5.8.3) and processed as per default workflow. MS tolerance is 4.5 ppm, and MS/MS tolerance is 20 ppm. Searches were performed against the UniProtKB human database (taxonomy 9606, version 20180929). Reversed database searches were used to evaluate false discovery rate (FDR) of site, peptide and protein identifications. Two missed cleavage sites of trypsin were allowed. Carbamidomethylation (C) was set as a fixed modification, and Acetyl (Protein N-term), Oxidation (M), deamidation (NQ), modified probes were set as variable modifications. The FDR of both peptide identification and protein identification is set to be 1%. The options of “Second peptides”, “Match between runs” and “Dependent peptides” were used. Label-free quantification was used to quantify the difference between different samples.

As such there is no fixed cut-off score threshold but instead spectra were accepted until the 1% false discovery rate (FDR) is reached. Only peptides with a minimum length of 7 amino acids were considered for identification and detected in at least one or more of the replicates. All probes modified peptide spectra were manually validated by applying stringent acceptance criteria: only modification event on K with PEP ≤ 0.01 were used for further analysis. Assignments were screened for peptides uniquely labeled on a single amino acid residue in two out of three biological replicates.

### Pull-down/western blot experiments

For target validation, cells were treated with probes for 2–4 h, DMSO-treated samples were used as negative controls. Upon cell lysis, the protein concentrations were determined by using the BCA protein assay (PierceTM BCA protein assay kit) and diluted to 1 mg/mL with PBS. A freshly pre-mixed click chemistry reaction cocktail was added (100 μM biotin-N_3_ from 10 mM stock solution in DMSO, 100 μM TBTA from 10 mM freshly prepared stock solution in DMSO, 2 mM sodium ascorbate from 200 mM freshly prepared stock solution in deionized water, and 1 mM CuSO_4_ from 100 mM freshly prepared stock solution in deionized water). The reaction was further incubated for 2 h prior to the addition of pre-chilled MeOH at -20°C and dissolved in PBS containing 1% SDS. Upon incubation with streptavidin beads for 4 hours at r.t., the beads were washed with PBS containing 1% SDS (thrice), PBS containing 0.1% SDS (once) and PBS (thrice). 50 µL (2×) SDS loading buffer was added and was heated at 95 °C for 30 min. The samples were separated by SDS-PAGE (10% or 8% gel) and the target protein was validated by western blot. Western blot experiments were carried out as previously described using the corresponding antibodies.

### Cell signaling and phosphorylation assay

H3255 cells were grown to 80–90% confluence in six-well plates under conditions described above. The H3255 cells were treated with 0.05–10 μM E3 or Erlotinib for 2 h, then washed two times with PBS to remove excessive probes. The cells were treated with PBS containing 2.5% chaps and phosphatase inhibitor (Thermo ScientificTM #88669), the suspended cells were lysed with sonication (14 × 3 s with 5 s breaks, 12% power) on ice. A soluble protein solution was obtained by centrifugation for 10 min (14,000 rpm, 4 °C). Eventually, the protein concentrations were determined by using the BCA protein assay (PierceTM BCA protein assay kit) and diluted to 1 mg/mL with PBS, and 80 μL of lysate was used by the addition of 20 μL of 5×SDS loading buffer and heating at 95 °C for 10 min. The phosphorylation level of EGFR L858R and its downstream proteins ERK/AKT inhibition was detected by Western blot.

### Wash-out experiments

The cells were grown to 80–90% confluence in six-well plate under conditions described above. The cells were treated with probes for 2 h at 37 °C, then washed two times with PBS and cultured with fresh medium. The cells were harvested and lysed at different time points between 0 and 24 and were detected by Western blot.

### Crystal structure analysis of the modification sites in proteins

To examine the locations of the identified sites, the protein crystal structures were analyzed with PDB code. Representative sites that locate in the active sites or their distance from the known active-site ligands within 10 Å. For proteins without PDB structures, BLAST was used instead to analyze the structural homologues. All protein structures were analyzed by using PyMol.

### Transfection of plasmids

The full-length of PFKAP, MEK2, CDK2, CDK5, STK38, MKNK1, CHK2, and CDK1 were cloned into a pReceiver-M14 vector with a C-terminal 3 × Flag tag. Plasmids of above protein and empty vector were transiently transfected in Opti-MEM using Lipofectamine 2000 according to the manufacturer’s protocol, respectively. Each well of HEK 293T cells was transfected in Opti-MEM with 3 μg of plasmids/10 μL of Lipofectamine 2000. After incubation for 8 h, the cells were changed with fresh medium and incubated for an additional 40 h. Western blotting analysis and labeling of probes were then performed.

### siRNA transfection

In order to validate the identified target, a small interfering RNA (siRNA) duplex targeting PFKAP purchased from Cohesion Biosciences company (catalog# CRH1995) was used to knock down gene expression in MDA-MB-231 cells. In order to optimize the transfection conditions, different concentrations of siRNA and transfection reagent were used. As a negative control, a scrambled oligonucleotide (NControl) was used. MDA-MB-231 cells were seeded into a six-well plate, and Lipofectamine RNAi MAX (Invitro, USA) was used according to the manufacturer’s recommendations, and siRNA was dissolved in 125 μL Opti-MEM solution, then dissolved Lipofectamine RNAi MAX in 125 μL opti-MEM medium. After standing for 5 min, siRNA was added to Lipofectamine RNAi MAX and mixed together for 15 min, and finally the siRNA compound was added to 2 mL of culture medium. After incubating for 8 h, the medium was changed to a medium containing serum and incubated for another 40 h, the cells were treated with the indicated concentrations of A1 and then washed twice with PBS and lysed. Finally, transfection efficiency was determined by using Western blotting and in-gel labeling.

### Docking experiments

Molecular docking between A1 and PFKAP, E3 and EGFR, X1 and CDK1 were performed in Maestro 11.5 (Schrödinger LLC). The crystal structure of PFKAP, EGFR, CDK1, CDK2, and CDK5 was downloaded from the Protein Data Bank (PDB code: 4WL0, 4LQM, 4Y72, 1B38, and 1H4L). Optimization of the protein structure including hydrogen atoms addition, missing side chains addition, water deletion, protonation state adjustment, and restrained minimization were performed using the “Protein Preparation Wizard” workflow in Maestro 11.5, with default parameters for further docking study. The 3D structure of X was generated using LigPrep module with OPLS3e force field. Covalent docking of X and PFKAP/EGFR L858R/CDK1/CDK2/CDK5 protein was performed using “Covalent Docking” panel using custom reaction type in Maestro 11.5, choosing K688/K728/K33/K33/K33 as reactive residues and the center of docking box. The figures were generated using PyMol. Sequence number of residues were verified using data from UniProt.

### Modification site selectivity analysis of A1/A2/A3/NHS-1 by quantitative proteomics in cell lysates^45^

In order to screen the above modification sites, we used H/L Azido-UV-biotin for data analysis by LC-MS/MS. MDA-MB-231 cell lysates were incubated with 100 μM of probe A1/A2/A3/NHS-1 at 37 °C for 4 h. The probe-labeled protein samples were incubated with 10 mM DTT and 40 mM IAA at 37 °C for 30 min. To remove all the excess small molecules, proteins were then precipitated with a methanol-chloroform system (aqueous phase/methanol/chloroform, 4:4:1 (v/v/v)). In brief, proteins were collected at the phase interface as a solid white disk after centrifugation at 2000 × *g* for 15 min at 4 °C. Liquid layers were discarded, and the protein was washed twice in prechilled methanol/chloroform (1:1, v/v), followed by centrifugation at 12,000 × *g* for 5 min at 4 °C. The precipitated proteins were resuspended with 50 mM ammonium bicarbonate with sonication (10 s with 20% output, repeat three times). Protein concentrations of lysate samples were then determined with the BCA assay (Pierce Thermo Fisher), 1 mg protein was used and digested with trypsin at a 1:50 (enzyme/substrate) ratio for 16 h at 37 °C. The tryptic digests were desalted with HLB extraction cartridges (Waters) as described previously. The dried peptides were resuspended in a water solution 30 µL including 30% acetonitrile (ACN) buffer. CuAAC reaction was then performed by subsequently adding 1 mM either light or heavy azido-UV-biotin (1 μL of a 40 mM stock, KeraFast, cat. no. EVU102 and KeraFast, cat. no. EVU151), 10 mM sodium ascorbate (4 μL of a 100 mM stock, sigma-Aldrich, cat. no. A7631), 1 mM TBTA (1 μL of a 50 mM stock, Sigma-Aldrich, cat. no. 678937, and 10 mM CuSO_4_ (4 μL of a 100 mM stock, Sigma-Aldrich, cat.no. 678937). After 2 h incubation at rt. in dark, the light and heavy isotopic tagged samples then mixed immediately following click reaction. The peptides were diluted with 120 µL 50 mM sodium acetate buffer (NaAc, pH 4.5) and then subject to the enrichment with streptavidin agarose beads (GE cat. no. 17-5113-01). After 2 h of incubation at r.t., streptavidin beads were washed 2–3 times with 50 mM NaAc (pH 4.5), 50 mM NaAc containing 2 M NaCl (pH 4.5), and deionized water twice each with vortexing and/or rotation to remove non-specific binding substances, then resuspended in 25 mM ammonium bicarbonate. The resulting mixture was transferred to glass tubes (VWR), and irradiated with 365 nm UV light (UVP, cat. no. UVL-28 EL series) for 2 h at r.t. with magnetic stirring. The supernatant was collected, concentrated under vacuum, and desalted with HLB extraction cartridges. The supernatant was collected, dried under vacuum, and stored at −20 °C until LC-MS/MS analysis.

The MS data was directly imported into pChem (http://pfind.ict.ac.cn/software/pChem/index.html) that could automatically invoke an efficient open-search engine 4 to generate all possible mass shifts within the given range (±1000 Da by default) at the PSM (peptide spectrum match)-level. Then, all mass shifts were calibrated with the calculated system error and unified to generate modification candidates. The latter were automatically paired according to the isotopic mass difference between a pair of light and heavy PDMs (12C6/13C6, 6.020132Da). Furthermore, all non-PDMs and unrealistic modifications were filtered out by (1) neglecting a measured mass difference out of the range of [6.020132 − 0.001, 6.020132 + 0.001] Da, corresponding to 166 ppm mass tolerance for the theoretical value; (2) discarding less abundant modification candidates with the PSM counting number lower than 5% of total; (3) removing the modifications with masses lower than 200 Da.

### Quantitative proteomics to identify the binding sites of probes derived from natural products^47^

Iso-TOP ABPP experiments. For in vitro profiling, MDA-MB-231 cells were lysed in pre-chilled NETN buffer (50 mM HEPES, pH 7.6, 150 mM NaCl, and 1% IGEPAL) and was added with 1 × protease and phosphatase inhibitors (Thermo Scientific, A32961). The cell lysates were then incubated with NHS-containing natural products at 100 μM concentration or DMSO at rt for 1h with rotation (other natural product fragments or DMSO at rt. for 4 h based on various reactive warhead). This was followed by incubation with NHS-1 (100 μM) for 1 h at rt. For in situ labeling, MDA-MB-231 cells were incubated with 20 μM of natural product fragments at 37 °C for 4 h; MDA-MB-231 cells were lysed as described above and were then incubated with NHS-1 (100 μM) at r.t. for 1 h. The probe-labeled protein samples were processed using a protocol as described above. The probe-labeled samples were then incubated with 10 mM DTT at room temperature for 30 min and then was added with 40 mM IAA for 30 min incubation in dark at room temperature. After that, the proteins were then precipitated with a methanol-chloroform system (aqueous phase/methanol/chloroform, 4:4:1 (v/v/v)). After standing on ice for 15 min, and centrifuging at 2000 rpm for 15 min at 4 °C. The liquid was discarded and the protein was washed twice in prechilled methanol/chloroform (1:1, v/v), followed by centrifugation at 12,000 × *g* for 5 min at 4 °C. The precipitated proteins were resuspended with 50 mM ammonium bicarbonate with sonication (10 s with 20% output, repeat three times). Protein concentration of lysate samples was then determined with the BCA assay (Pierce Thermo Fisher), 1 mg protein was used and digested with trypsin at a 1:50 ratio for 16 h at 37 °C. The tryptic digests were desalted with HLB extraction cartridges (Waters) as described above, the dried peptides were resuspended in a 30 μL solution buffer containing 30% acetonitrile. Click reaction was then performed by adding 1 mM either light or heavy azido-UV-biotin (1 μL of a 40 mM stock, KeraFast, Cat. No. EVU102 and KeraFast, Cat. No. EVU151), 10 mM sodium ascorbate (4 μL of a 100 mM stock, sigma-Aldrich, Cat. No. A7631), 1 mM TBTA (1 μL of a 50 mM stock, Sigma-Aldrich, Cat. No. 678937), and 10 mM CuSO_4_ (4 μL of a 100 mM stock, Sigma-Aldrich, Cat. No. 678937). After 2 h incubation at rt. in dark, the light and heavy isotopic tagged samples then mixed immediately. The peptides were diluted with 120 μL 10 × 50 mM sodium acetate buffer (NaAc, pH 4.5) and then subjected to streptavidin agarose beads (GE Cat. No. 17-5113-01). After 2 h of incubation at rt., streptavidin beads were washed with 2–3 times of 50 mM NaAc (pH 4.5), 50 mM NaAc containing 2 M NaCl (pH 4.5), and deionized water twice, and then resuspended in 25 mM ammonium bicarbonate. The samples were then transferred to glass tubes (VWR), and irradiated with 365 nm UV light (UVP, Cat. No. UVL-28 EL series) for 2 h at r.t. with magnetic stirring. The supernatant was collected, concentrated under vacuum, and desalted with HLB extraction cartridges. The supernatant was collected, dried under vacuum, and stored at −20 °C until LC-MS/MS analysis. LC-MS/MS analysis was performed on a Q Extractive TM HF-X (Thermo Fisher Scientific) equipped with a UltiMateTM 3000 Rusnano system (Thermo Fisher Scientific) for online sample handling and peptide separations. Then 6 µL of sample was loaded onto a fused-silica nano-ESI column (360 µm OD × 150 µm ID) with a needle tip (3~5 µM) packed to a length of 15 cm with a C18 reverse phase resin (AQ 1.9 μm, 120 Å, ReproSil-Pur). The peptides were separated using an 88 min linear gradient from 6 to 95% buffer B (80% ACN) equilibrated with buffer A (0.1% formic acid) at a flow rate of 600 nL/min across the column. The scan sequence for the Orbitrap began with an MS1 spectrum (Orbitrap analysis, resolution 120,000, scan range of 350–1550 *m/z*, AGC target 3×106, maximum injection time 20 ms, dynamic exclusion of 15 s). The “Top25” precursors was selected for MS2 analysis, in which precursors were fragmented by HCD prior to Orbitrap analysis ((N) CE 27, AGC target 2×104, maximum injection time 30 ms, resolution 15,000, and isolation window: 1.6 Da.

### Data analysis of the quantitative proteomics

Raw data files were searched against the Homo sapiens UniProt canonical database (20210516) using pFind studio (v3.1.5, http://pfind.ict.ac.cn/software/pFind/index^47^. Precursor ion mass and fragmentation tolerance were set as 10 ppm and 20 ppm, respectively. The maximum number of modifications and missed cleavages allowed per peptide were both set as three. For all analyses, mass shifts of common modifications (i.e., methionine oxidation + 15.9949 Da; and cysteine carbamidomethylation + 57.0214 Da) and mass shifts of 267.121894 (12C(6)13C (6)H(17)N(3)O(4)) for NHS-1 modification. An isotopic mass difference of 6.020132 Da (12C6/13C6) on probe-derived modifications was used for quantification. The FDRs were estimated by the program from the number and quality of spectral matches to the decoy database. The FDRs at spectrum, peptide, and protein level were <1%. Quantitative analyses were performed using pQuant, which calculates heavy to light ratios based on each identified MS scan with a 15 ppm-level *m/z* tolerance window and assigns an interference score (Int. Score) to each value from zero to one. The median values of probe-modified peptide ratios with σ less than or equal 0.5 were considered to calculate site-level ratios. For peptides exhibiting a ≥90% reduction in MS1 peak intensity, a maximal ratio of 10 was assigned. Overall, quantification results were obtained from at least two biological replicates.

**The supplementary methods** including synthetic procedures of probes, identification of conjugation product between A2 and ethylamine, stability analysis of A1, the reactivity analysis of A1/A2/A3/A4 with amino compounds, HPLC analysis of the reactivity between A1 and different amino acids, database search, Annexin V-FITC/PI dual staining analysis, cellular thermal shift assay (CETSA) and pull-down experiment to validate the targets of natural ligand were shown in Supporting Information.

### Reporting summary

Further information on research design is available in the Nature Portfolio Reporting Summary linked to this article.

### Supplementary information

Supporting information

Description of Additional Supplementary Files

Supplementary Data 1

Supplementary Data 2

Reporting Summary

## Data Availability

Any relevant data are available from the authors upon reasonable request.
