# Supplementary material for: Catalyst-free late-stage functionalization to assemble α-acyloxyenamide electrophiles for selectively profiling conserved lysine residues
Source: Commun Chem. 2024 Feb 14;7:31. doi: 10.1038/s42004-024-01107-4 (PMC10866925; doi:10.1038/s42004-024-01107-4)
Supplement: Supplementary file 1 — Supporting information [file 42004_2024_1107_MOESM1_ESM.pdf]

Supporting Information

Catalyst-Free Late-Stage Functionalization to Assemble α-acyloxyenamide Electrophiles for Selectively Profiling Conserved Lysine Residues

Yuanyuan Zhao,[1,2,8] Kang Duan,[1,2,8] Youlong Fan,[1,2,8] Shengrong Li,[3,8] Liyan Huang,[1,2] Zhengchao Tu,[1,2] Hongyan Sun,[4] Gregory M. Cook,[5] Jing Yang,[6] Pinghua Sun,[1,2] Yi Tan,[*1,2] Ke Ding,[*1,2] Zhengqiu Li[*1,2,7]

[1]State Key Laboratory of Bioactive Molecules and Druggability Assessment, Jinan University, 601 Huangpu Avenue West, Guangzhou, 510632 China

[2]International Cooperative Laboratory of Traditional Chinese Medicine Modernization and Innovative Drug Development (MOE), School of Pharmacy, Jinan University, 601 Huangpu Avenue West, Guangzhou, 510632 China

[3]Guangdong Second Provincial General Hospital, Postdoctoral Station of Traditional Chinese Medicine, Jinan University, Guangzhou 510632, China

[4]Department of Chemistry and COSDAF (Centre of Super-Diamond and Advanced Films), City University of Hong Kong, 83 TatChee Avenue, Kowloon, Hong Kong, China 999077

[5]Department of Microbiology and Immunology, University of Otago, Dunedin 9054, New Zealand

[6] Guangzhou National Laboratory, Guangzhou International Bio Island, Guangzhou 510005 China

[7]MOE Key Laboratory of Tumor Molecular Biology, Jinan University, 601 Huangpu Avenue West, Guangzhou, 510632 China

[8]These authors contributed equally to this work.

Email: pharmlzq@jnu.edu.cn (Z. Li), dingke@jnu.edu.cn (K. Ding), tanyi@jnu.edu.cn (Y. Tan)

Supplementary Notes

1. Structures of probes and reporters

2. Supplementary Methods

3. Supplementary Figure S1-S29

Table S1. Structures of probes and reporters

Tool probes and kinase inhibitor derived probes

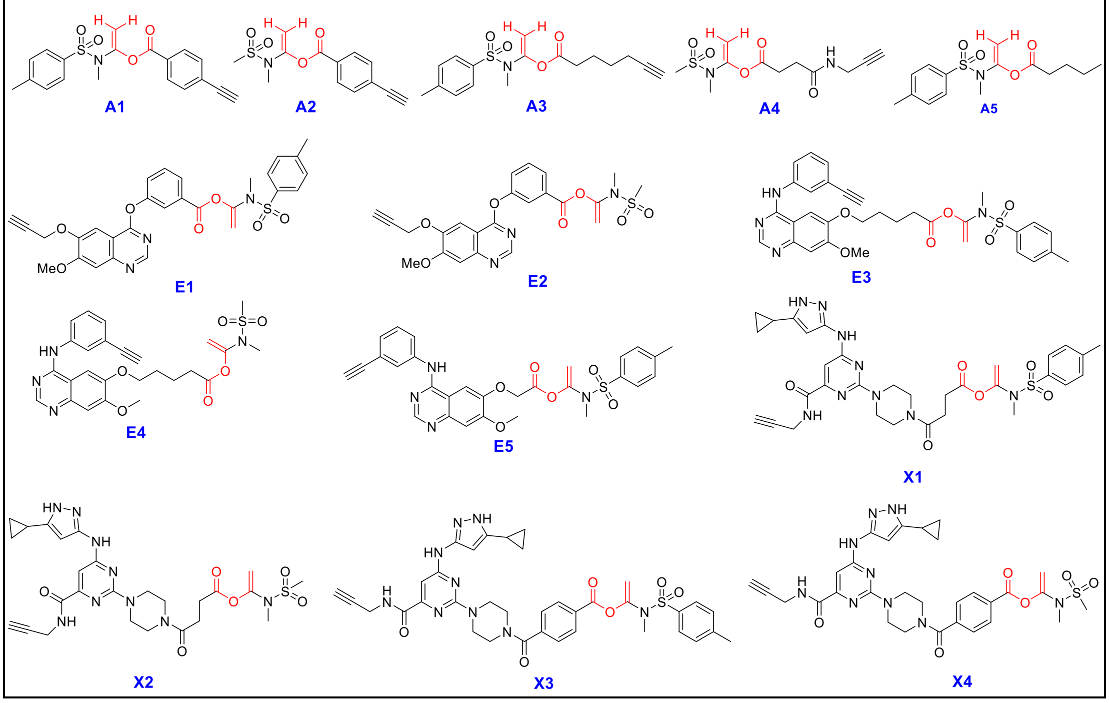

Natural product based probes

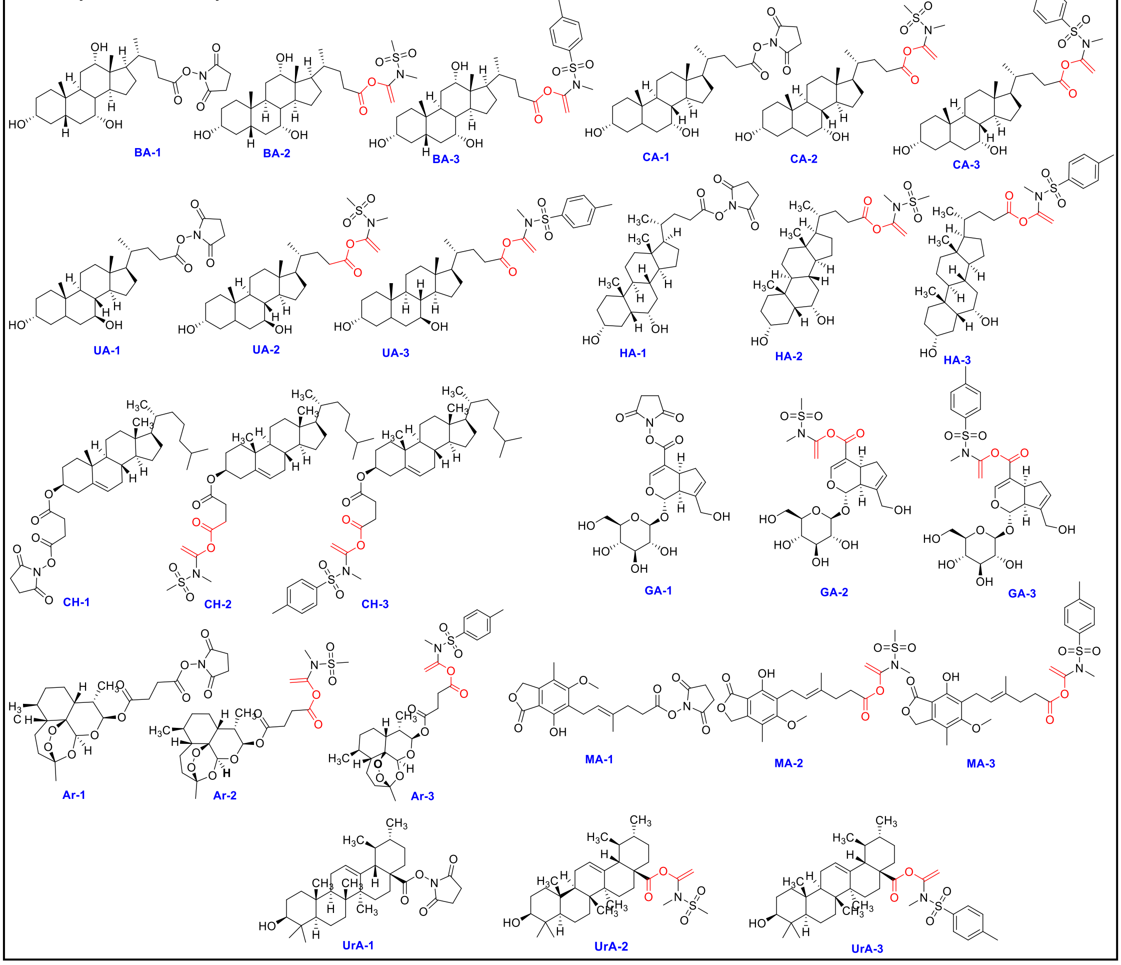

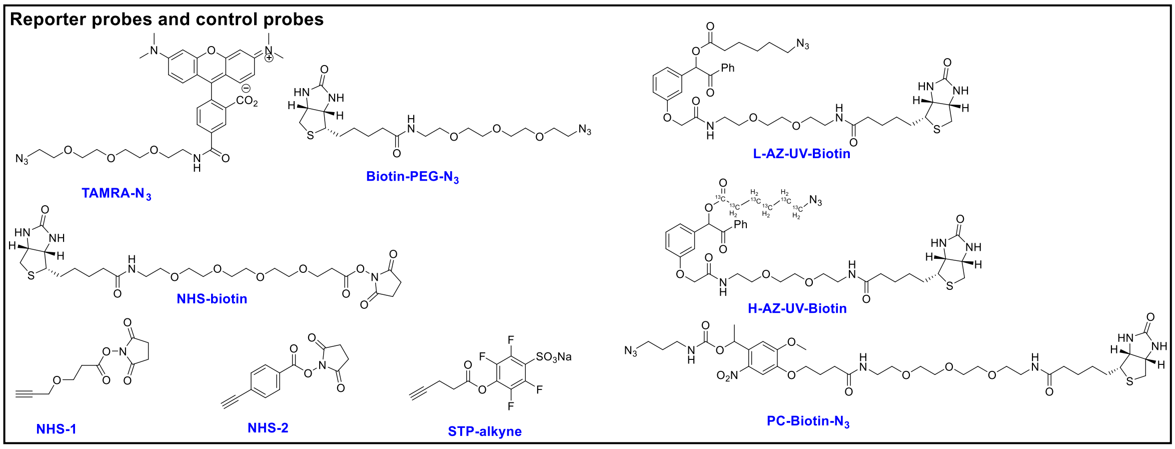

Supplementary Methods

1. Synthetic procedures of probes

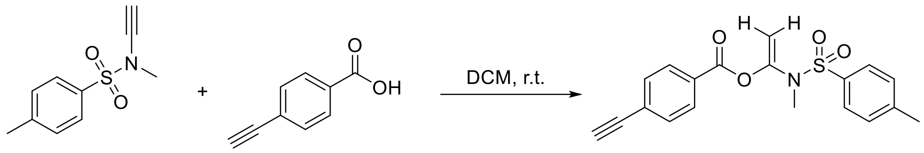

S1 **S2** **A1**

Scheme S1

1-((*N*,4-dimethylphenyl)sulfonamido)vinyl 4-ethynylbenzoate (**A1**). To a solution of *N*-methylynetoluenesulfonamide (MYTsA) **S1** (50 mg, 0.24 mmol) in 5 mL DCM was added 4-ethynylbenzoic acid **S2** (70 mg, 0.48 mmol), the mixture was stirred for 8 h at r.t. and the solvent was removed under vacuum. The crude reaction mixture was purified by flash chromatography eluting with petroleum ether/acetone (3:1) to give the desired product **A1** as a white solid (68 mg, 80%). ^{1}H NMR (400 MHz, $CDCl_3$) δ 7.90 – 7.81 (m, 2H), 7.79 – 7.71 (m, 2H), 7.57 – 7.48 (m, 2H), 7.28 (d, J = 7.9 Hz, 2H), 5.04 (d, J = 2.5 Hz, 1H), 4.87 (d, J = 2.6 Hz, 1H), 3.29 (s, 1H), 3.12 (s, 3H), 2.41 (s, 3H). ^{13}C NMR (151 MHz, $CDCl_3$) δ 163.4, 146.9, 144.1, 134.2, 132.1, 130.0, 129.6, 128.6, 127.9, 127.9, 127.7, 101.7, 82.6, 80.8, 37.3, 21.5.ESI-MS calcd for $C_{19}H_{17}NO_4S$ $[M+Na]^+$ m/z = 378.0770; Found 378.0773.

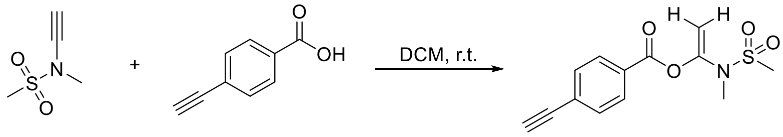

S3 **S2** **A2**

Scheme S2

1-(*N*-methylmethylsulfonamido)vinyl 4-ethynylbenzoate (**A2**). The synthesis protocol is same as **A1**, product **A2** was afforded as a white solid (46 mg, 69% yield). ^{1}H NMR (400 MHz, $CDCl_3$) δ 8.04 (d, J = 8.5 Hz, 2H), 7.59 (d, J = 8.5 Hz, 2H), 5.13 (d, J = 2.7 Hz, 1H), 4.99 (d, J = 2.7 Hz, 1H), 3.28 (s, 1H), 3.18 (s, 3H), 3.02 (s, 3H). ^{13}C NMR (151 MHz, $CDCl_3$) δ 163.6, 146.3, 132.4, 130.1, 128.3, 128.1, 100.3, 82.5, 81.0, 37.7, 36.2. ESI-MS calcd for $C_{13}H_{13}NO_4S$ $[M+Na]^+$ m/z = 302.0457; Found 302.0443.

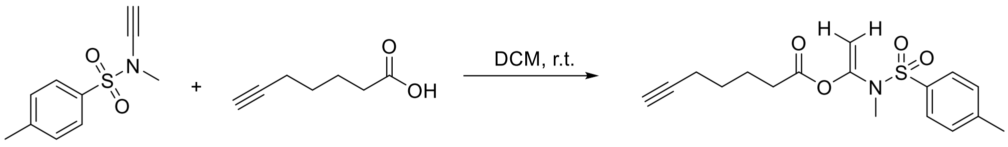

S1 **S4** **A3**

Scheme S3

1-((*N*,4-dimethylphenyl)sulfonamido)vinyl hept-6-ynoate (**A3**). The synthesis protocol is same as **A1**, product **A3** was afforded as a white oil (48 mg, 60% yield). ^{1}H NMR (400 MHz, $CDCl_3$) δ 7.78 – 7.65 (m, 2H), 7.33 (d, *J* = 8.1 Hz, 2H), 4.81 (d, *J* = 2.6 Hz, 1H), 4.62 (d, *J* = 2.6 Hz, 1H), 3.01 (s, 3H), 2.44 (s, 3H), 2.40 – 2.29 (m, 2H), 2.20 (m, 2H), 1.96 (t, *J* = 2.6 Hz, 1H), 1.78 – 1.63 (m, 2H), 1.63 – 1.47 (m, 2H). ^{13}C NMR (151 MHz, $CDCl_3$) δ 170.8, 147.0, 144.1, 133.8, 130.0, 129.6, 129.6, 129.5, 128.0, 128.0, 128.0, 127.3, 100.4, 83.8, 68.8, 37.3, 33.4, 27.6, 23.5, 21.6, 18.1. ESI-MS calcd for $C_{17}H_{21}NO_4S$ $[M+Na]^+$ m/z = 358.1084; Found 358.1074.

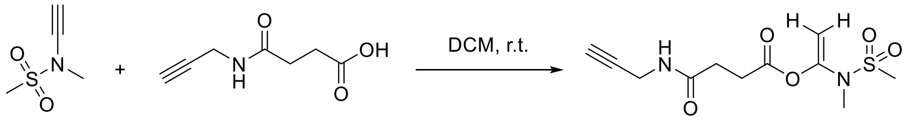

S3 **S5** **A4**

Scheme S4

1-(*N*-methylmethylsulfonamido)vinyl 4-oxo-4-(prop-2-yn-1-ylamino)butanoate (**A4**). The synthesis protocol is same as **A1**, product **A4** was afforded as a white oil (24 mg, 35% yield). ^{1}H NMR (400 MHz, DMSO) δ 8.38 (t, *J* = 5.6 Hz, 1H), 5.00 (d, *J* = 2.4 Hz, 1H), 4.82 (d, *J* = 2.4 Hz, 1H), 3.85 (dd, *J* = 5.5, 2.6 Hz, 2H), 3.10 (t, *J* = 2.5 Hz, 1H), 3.05 (s, 3H), 3.00 (s, 3H), 2.66 (t, *J* = 6.7 Hz, 2H), 2.44 (t, *J* = 6.8 Hz, 2H).^{13}C NMR (151 MHz, DMSO-d_6) δ 174.2, 171.2, 170.9, 170.8, 100.2, 81.6, 73.5, 37.5, 36.4, 29.8, 29.2, 28.3. ESI-MS calcd for $C_{11}H_{16}N_2O_5S$ $[M+Na]^+$ m/z = 311.0672; Found 311.0672.

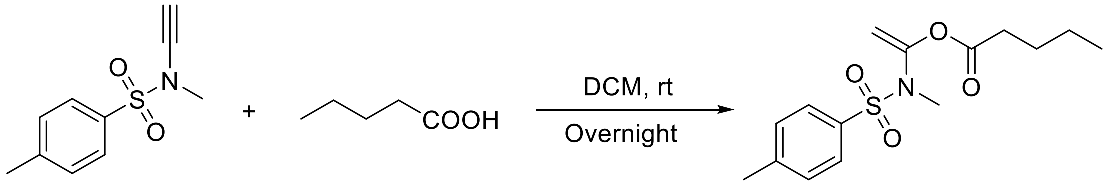

Scheme S5

1-((N,4-dimethylphenyl)sulfonamido)vinyl pentanoate (**A5**). The synthesis protocol is same as A1, product **A5** was afforded as a white solid (50 mg, 67% yield). ^{1}H NMR (400 MHz, $CDCl_3$) δ 7.72 (d, *J* = 8.3 Hz, 2H), 7.35 – 7.29 (m, 2H), 4.81 (d, *J* = 2.5 Hz, 1H), 4.63 (d, *J* = 2.5 Hz, 1H), 3.01 (s, 3H), 2.43 (s, 3H), 2.31 (t, *J* = 7.5 Hz, 2H), 1.60 – 1.49 (m, 2H), 1.33 (m, 3H), 0.90 (t, *J* = 7.4 Hz, 3H). ^{13}C NMR (151 MHz, $CDCl_3$) δ 171.3, 147.0, 144.0, 134.0, 130.0, 129.5, 128.0, 127.3, 100.6, 37.3, 33.7, 26.5, 22.1, 21.6, 13.7. ESI-MS calcd for $C_{15}H_{22}NO_4S$ $[M+Na]^+$ m/z = 312.1270; Found 312.1268.

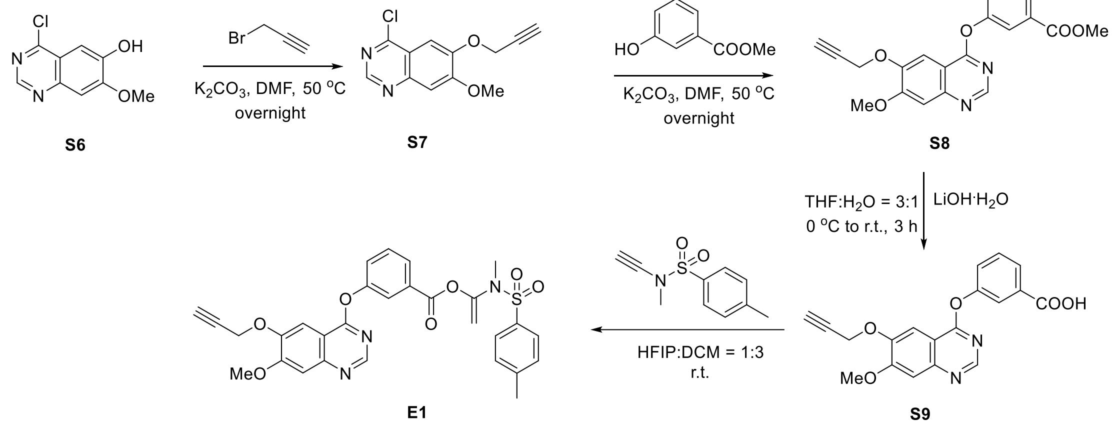

Scheme S6

4-chloro-7-methoxy-6-(prop-2-yn-1-yloxy)quinazoline (**S7**). **S6** (210 mg, 1.0 mmol), 3-bromopropyne (178 mg, 1.5 mmol), potassium carbonate (276 mg, 2.0 mmol) and DMF (5 mL) were added to a round bottom flask. After stirring at 50°C for 2 h, the mixture was added with water (20 mL) and extracted with ethyl acetate (3 ×15 mL). All organic layers were combined and washed with water (3×10 mL). Finally, the solvent was dried with Na_2SO_4 and evaporated under reduced pressure. The crude product was purified by flash chromatography eluting with ethyl acetate/petroleum ether (1:3) to produce **S7** as a white solid (156 mg, 63% yield). ^{1}H NMR (400 MHz, $CDCl_3$) δ 8.88 (s, 1H), 7.61 (s, 1H), 7.36 (s, 1H), 4.96 (d, J = 2.4 Hz, 2H), 4.07 (s, 3H), 2.63 (t, J = 2.4 Hz, 1H).

methyl 3-((7-methoxy-6-(prop-2-yn-1-yloxy)quinazolin-4-yl)oxy)benzoate (**S8**). **S7** (150 mg, 0.6 mmol), methyl 3-hydroxybenzoate (137 mg, 0.9 mmol), potassium carbonate (166 mg, 1.2 mmol) and DMF (3 mL) was added to a round bottom flask. After stirring at 50°C overnight, the mixture was added water (15 mL) and extracted with ethyl acetate (3×15 mL). The organic layers were combined and washed with water (3×10 mL). Finally, the solvent was dried with Na_2SO_4 and evaporated under reduced pressure. The crude product was purified by flash chromatography eluting with ethyl acetate/DCM (10:1) to produce **S8** as a white solid (206 mg, 94%). ^{1}H NMR (400 MHz, $CDCl_3$) δ 8.65 (s, 1H), 8.02 (m, 1H), 7.96 (t, J = 2.0 Hz, 1H), 7.74 (s, 1H), 7.59 (t, J = 7.9 Hz, 1H), 7.51 (m, 1H), 7.37 (s, 1H), 4.98 (d, J = 2.4 Hz, 2H), 4.09 (s, 3H), 3.95 (s, 3H), 2.63 (t, J = 2.4 Hz, 1H). ^{13}C NMR (151 MHz, $CDCl_3$) δ 166.2, 165.3, 156.2, 153.1, 152.5, 149.8, 148.0, 132.0, 129.7, 127.1, 126.7, 123.3, 110.4, 107.2, 103.2, 56.9, 56.4, 52.4.

3-((7-methoxy-6-(prop-2-yn-1-yloxy)quinazolin-4-yl)oxy)benzoic acid (**S9**). To a round-bottom flask (25 mL) was added **S8** (146 mg, 0.4 mmol) and THF/H_2O (3:1, 4 mL) followed by addition of LiOH·H_2O (34 mg, 0.8 mmol) at 0°C. The resulting mixture was stirred at r.t. for 3 h, and then concentrated. The residue was acidified to pH 4-5 with 1M HCl, and then extracted with DCM. The organic layers were combined and dried over Na_2SO_4. Upon evaporation of the solvent under reduced pressure, the product **S9** was obtained as a white solid (110 mg, 79% yield). ^{1}H NMR (400 MHz, DMSO-d_6) δ 13.21 (s, 1H), 8.58 (s, 1H), 7.90 (dt, J = 6.7, 1.8 Hz, 1H), 7.81 (d, J = 2.3

Hz, 1H), 7.74 (s, 1H), 7.63 (dd, J = 4.9, 2.0 Hz, 2H), 7.43 (s, 1H), 5.06 (d, J = 2.4 Hz, 2H), 4.01 (s, 3H), 3.69 (t, J = 2.4 Hz, 1H). ^{13}C NMR (151 MHz, DMSO-d_6) δ 167.0, 165.2, 156.4, 152.9, 152.8, 149.7, 148.3, 133.0, 130.6, 127.3, 127.0, 123.2, 109.9, 107.5, 103.3, 79.6, 79.0, 56.9, 56.7.

1-((*N*,4-dimethylphenyl)sulfonamido)vinyl-3-((7-methoxy-6-(prop-2-yn-1-yloxy)quinazolin-4-yl)oxy) benzoate (**E1**). **S9** (35 mg, 0.1 mmol) was dissolved in hexafluoroisopropanol (HFIP) (0.5 mL) and DCM (1.5 mL), followed by addition of *N*-methylynetoluenesulfonamide (MYTsA) (21 mg, 0.1 mmol). After stirring at r.t. for 5 minutes, the solvent was removed under vacuum, and the crude reaction mixture was purified by flash chromatography eluting with petroleum ether/ acetone (3:1) to give the desired product **E1** as a white solid (30 mg, 54% yield). ^{1}H NMR (400 MHz, $CDCl_3$) δ 8.65 (s, 1H), 7.87 (dd, J = 4.7, 2.4 Hz, 2H), 7.81 – 7.70 (m, 3H), 7.59 – 7.52 (m, 2H), 7.39 (s, 1H), 7.30 – 7.23 (m, 3H), 5.04 (d, J = 2.6 Hz, 1H), 5.00 (d, J = 2.4 Hz, 2H), 4.86 (d, J = 2.6 Hz, 1H), 4.10 (s, 3H), 3.12 (s, 3H), 2.65 (t, J = 2.4 Hz, 1H), 2.35 (s, 3H). ^{13}C NMR (151 MHz, $CDCl_3$) δ 165.2, 163.3, 156.3, 153.0, 152.5, 149.9, 148.1, 147.0, 144.1, 134.1, 130.5, 129.8, 129.6, 128.0, 127.7, 127.6, 123.8, 110.3, 107.3, 103.2, 101.8, 56.9, 56.5, 37.3, 21.5. ESI-MS calcd for $C_{29}H_{26}N_3O_7S$ $[M+H]^+$ m/z = 560.1491; Found 560.1486.

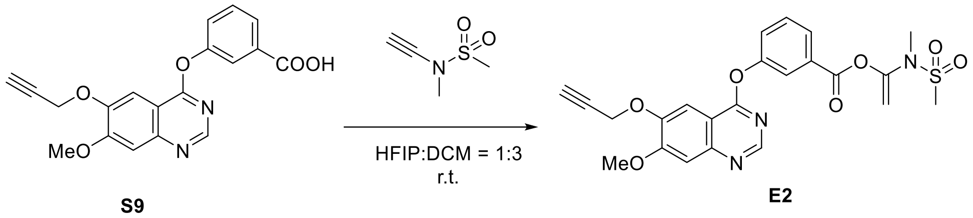

Scheme S7

1-(*N*-methylmethylsulfonamido)vinyl-3-((7-methoxy-6-(prop-2-yn-1-yloxy)quinazolin-4-yl)oxy)benzoate (**E2**). **S9** (35 mg, 0.1 mmol) was dissolved in HFIP (0.5 mL) and DCM (1.5 mL), followed by addition of *N*-methylynemethylsulfonamide (MYMsA) (14 mg, 0.1 mmol). After stirring at r.t. for 5 minutes, the solvent was removed under vacuum, and the crude reaction mixture was purified by flash chromatography eluting with petroleum ether/acetone (3:1) to give the desired product **E2** as a white solid (31 mg, 65% yield). ^{1}H NMR (400 MHz, $CDCl_3$) δ 8.67 (s, 1H), 8.08 (m, 1H), 8.02 (t, J = 1.9 Hz, 1H), 7.75 (s, 1H), 7.65 (t, J = 7.9 Hz, 1H), 7.59 (m, 1H), 7.46 (s, 1H), 5.16 (d, J = 2.7 Hz, 1H), 5.03 (d, J = 2.7 Hz, 1H), 4.99 (d, J = 2.4 Hz, 2H), 4.10 (s, 3H), 3.21 (s, 3H), 3.05 (s, 3H), 2.65 (t, J = 2.4 Hz, 1H). ^{13}C NMR (151 MHz, $CDCl_3$) δ 165.1, 163.4, 156.3, 152.9, 152.7, 149.9, 148.1, 146.2, 130.2, 130.1, 128.1, 127.6, 123.8, 110.3, 107.2, 103.1, 100.4, 56.9, 56.4, 37.7, 36.2. ESI-MS calcd for $C_{23}H_{22}N_3O_7S$ $[M+H]^+$ m/z = 484.1178; Found 484.1173.

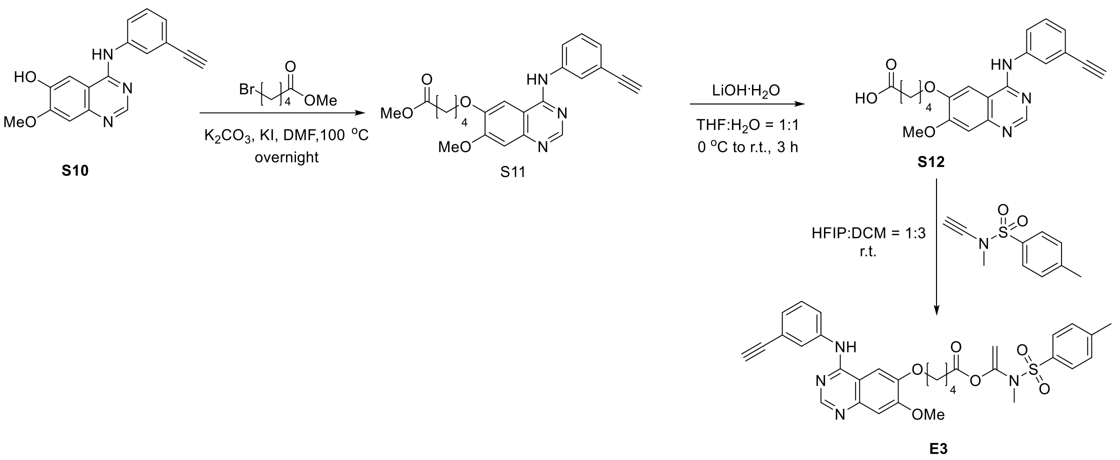

Scheme S8

S10 was synthesized according to the published procedures.[3]

Methyl 5-((4-((3-ethynylphenyl)amino)-7-methoxyquinazolin-6-yl)oxy)pentanoate (**S11**). **S10** (291 mg, 1.0 mmol), methyl 5-bromovalerate (293 mg, 1.5 mmol), potassium carbonate (166 mg, 1.2 mmol), potassium iodide (50 mg, 0.3 mmol) and DMF (5 mL) were added to a round bottom flask. After stirring at 100°C overnight, the mixture was added with water (20 mL) and extracted with ethyl acetate (3×20 mL). The organic layers were combined and washed with water (3×15 mL). Finally, the solvent was dried with Na_2SO_4 and evaporated under reduced pressure. The crude product was purified by flash chromatography eluting with ethyl acetate/petroleum ether (1:2) to produce **S11** as a white solid (237 mg, 59 % yield). ^{1}H NMR (400 MHz, $CDCl_3$) δ 8.68 (s, 1H), 8.06 (s, 1H), 7.93 (t, *J* = 1.8 Hz, 1H), 7.87 (m, 1H), 7.40 – 7.30 (m, 2H), 7.30 – 7.22 (m, 2H), 4.15 (t, *J* = 7.0 Hz, 2H), 3.98 (s, 3H), 3.68 (s, 3H), 3.09 (s, 1H), 2.47 (t, *J* = 6.9 Hz, 2H), 1.98 – 1.88 (m, 2H), 1.88 – 1.78 (m, 2H). ^{13}C NMR (151 MHz, $CDCl_3$) δ 169.8, 151.8, 150.4, 148.8, 143.7, 142.6, 134.4, 124.1, 122.9, 120.4, 117.9, 117.7, 117.7, 102.9, 96.9, 78.7, 63.9, 51.4, 47.2, 28.6, 22.6, 16.4.

5-((4-((3-ethynylphenyl)amino)-7-methoxyquinazolin-6-yl)oxy)pentanoic acid (**S12**). To a round-bottom flask (25 mL) was added **S11** (217 mg, 0.5 mmol) and THF/H_2O (1:1, 6 mL) followed by addition of LiOH·H_2O (42 mg, 1.0 mmol) at 0°C. The resulting mixture was stirred at r.t. for 2 h and then concentrated. The residue was acidified to pH 4-5 with 1M HCl, and was extracted with DCM. The organic layers were combined and dried over Na_2SO_4. Upon evaporation of the solvent under reduced pressure, the product **S12** was obtained as a white solid (147 mg, 71% yield). ^{1}H NMR (400 MHz, DMSO-d_6) δ 12.05 (s, 1H), 9.65 (s, 1H), 8.53 (s, 1H), 7.99 (s, 1H), 7.90 (d, *J* = 8.0 Hz, 2H), 7.42 (t, *J* = 7.9 Hz, 1H), 7.23 (d, *J* = 10.0 Hz, 2H), 4.18 (dd, *J* = 13.1, 6.8 Hz, 3H), 3.95 (s, 3H), 2.35 (t, *J* = 7.3 Hz, 2H), 1.90 – 1.81 (m, 2H), 1.73 (m, 2H). ^{13}C NMR (151 MHz, DMSO-d_6) δ 174.9, 156.6, 154.9, 153.2, 148.8, 147.5, 140.3, 129.4, 126.8, 125.3, 123.1, 122.2, 109.4, 107.8, 103.0, 81.1, 69.0, 56.4, 56.4, 33.8, 28.5, 21.8.

1-((*N*,4-dimethylphenyl)sulfonamido)vinyl-5-((4-((3-ethynylphenyl)amino)-7-methoxy-quinazolin-6-yl)oxy)pentanoate (**E3**). The synthesis protocol is same as **E1**, product **E3** was afforded as a white solid in 49 % yield (29 mg). ^{1}H NMR (400 MHz, $CDCl_3$) δ 8.67 (s, 1H), 8.10

(s, 1H), 7.88 (t, *J* = 1.8 Hz, 1H), 7.81 – 7.75 (m, 1H), 7.70 – 7.63 (m, 2H), 7.36 – 7.20 (m, 7H), 4.79 (d, *J* = 2.8 Hz, 1H), 4.43 (d, *J* = 2.8 Hz, 1H), 4.11 (t, *J* = 6.5 Hz, 2H), 3.96 (s, 3H), 3.07 (s, 1H), 2.96 (s, 3H), 2.49 (t, *J* = 6.9 Hz, 2H), 2.42 (s, 3H), 1.94 (m, 3H), 1.86 (m, 2H). ^{13}C NMR (151 MHz, $CDCl_3$) δ 171.3, 156.5, 155.2, 153.5, 148.8, 147.5, 147.4, 144.4, 139.0, 132.9, 129.7, 129.6, 128.8, 128.0, 127.7, 125.5, 125.5, 122.9, 122.8, 122.6, 109.3, 107.7, 107.6, 107.6, 101.6, 101.5, 100.1, 83.5, 68.5, 56.2, 37.9, 37.9, 33.4, 27.7, 21.6, 20.8. ESI-MS calcd for $C_{32}H_{32}N_4O_6SNa$ $[M+Na]^+$ m/z = 623.1940; Found 623.1935.

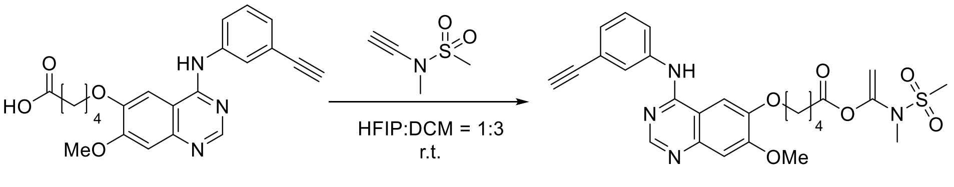

Scheme S9

1-(*N*-methylmethylsulfonamido)vinyl-5-((4-((3-ethynylphenyl)amino)-7-methoxyquinazolin-6-yl)oxy)pentanoate (**E4**). The synthesis protocol is same as **E2**, product **E4** was afforded as a white solid in 68 % yield (35 mg). ^{1}H NMR (400 MHz, $CDCl_3$) δ 8.68 (s, 1H), 7.90 – 7.78 (m, 3H), 7.36 (m, 1H), 7.30 – 7.26 (m, 2H), 7.25 (d, *J* = 2.1 Hz, 2H), 4.90 (d, *J* = 2.8 Hz, 1H), 4.84 (d, *J* = 2.8 Hz, 1H), 4.15 (t, *J* = 6.4 Hz, 2H), 3.99 (s, 3H), 3.10 (m, 4H), 2.97 (s, 3H), 2.58 (t, *J* = 6.8 Hz, 2H), 2.04 – 1.95 (m, 2H), 1.95 – 1.85 (m, 2H). ^{13}C NMR (151 MHz, $CDCl_3$) δ 166.5, 151.6, 150.4, 148.8, 148.8, 144.0, 142.7, 141.8, 134.3, 124.3, 124.3, 124.2, 123.0, 123.0, 123.0, 120.5, 120.4, 118.0, 118.0, 117.9, 117.8, 104.5, 103.1, 96.7, 94.4, 94.3, 78.7, 63.8, 51.4, 32.3, 32.2, 32.0, 28.7, 28.6, 22.9, 16.2, 16.1. ESI-MS calcd for $C_{26}H_{29}N_4O_6S$ $[M+H]^+$ m/z = 525.1808; Found 525.1802.

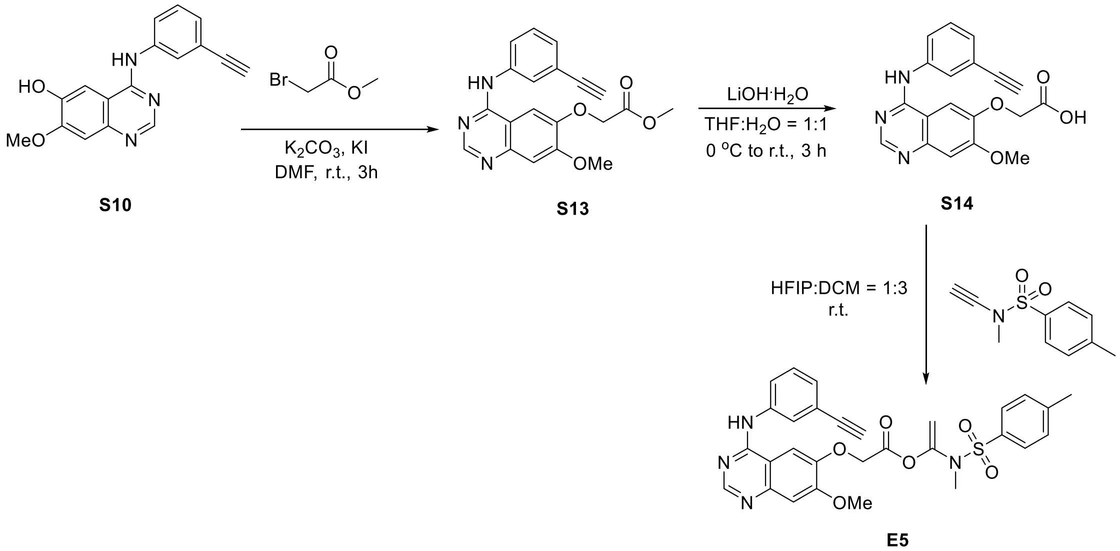

Scheme S10

Methyl 2-((4-((3-ethynylphenyl)amino)-7-methoxyquinazolin-6-yl)oxy)acetate (**S13**). **S10** (1 g, 3.4 mmol), methyl bromoalkanoate (788 mg, 5.1 mmol), potassium carbonate (948 mg, 6.8 mmol),

potassium iodide (170 mg, 1.0 mmol) and DMF (15 mL) were added to a round bottom flask. After stirring at r.t. for 3 h, the reaction mixture was added water (20 mL), and then filtered. Ethyl acetate was added to the liquid to form precipitate. The precipitate was filtered and dried to give compound **S13** as a grey powder (447 mg, 73%). ^{1}H NMR (400 MHz, DMSO-d_6) δ 9.47 (s, 1H), 8.51 (s, 1H), 7.95 (t, *J* = 1.9 Hz, 1H), 7.89 – 7.81 (m, 2H), 7.42 (t, *J* = 7.9 Hz, 1H), 7.27 – 7.20 (m, 2H), 4.98 (s, 2H), 4.21 (s, 1H), 3.97 (s, 3H), 3.75 (s, 3H). ^{13}C NMR (151 MHz, DMSO-d_6) δ 169.0, 156.7, 154.8, 153.6, 147.8, 147.5, 140.1, 129.4, 127.0, 125.4, 123.3, 122.2, 109.0, 108.1, 103.6, 83.9, 81.1, 65.7, 56.4, 52.4.

2-((4-((3-ethynylphenyl)amino)-7-methoxyquinazolin-6-yl)oxy)acetic acid (**S14**). The synthesis protocol is same as **S12**, product **S14** was afforded as a light-yellow solid (474 mg, 80% yield). ^{1}H NMR (400 MHz, DMSO-d_6) δ 9.51 (s, 1H), 8.51 (s, 1H), 7.95 (t, *J* = 1.8 Hz, 1H), 7.91 – 7.80 (m, 2H), 7.41 (t, *J* = 7.9 Hz, 1H), 7.32 – 7.17 (m, 2H), 4.88 (s, 2H), 4.21 (s, 1H), 3.96 (s, 3H).

1-((*N*,4-dimethylphenyl)sulfonamido)vinyl-2-((4-((3-ethynylphenyl)amino)-7-methoxy-quinazolin-6-yl)oxy)acetate (**E5**). The synthesis protocol is same as **E1**, product **E5** was afforded as a white solid (68% yield, 38 mg). ^{1}H NMR (400 MHz, $CDCl_3$) δ 8.58 (s, 1H), 8.09 (s, 1H), 7.68 – 7.62 (m, 2H), 7.60 (m, 1H), 7.44 (d, *J* = 1.3 Hz, 2H), 7.37 (s, 1H), 7.22 (d, *J* = 8.1 Hz, 2H), 7.15 – 7.10 (m, 2H), 5.11 (s, 2H), 4.82 (d, *J* = 3.4 Hz, 1H), 4.42 (d, *J* = 3.5 Hz, 1H), 4.06 (s, 3H), 3.01 (s, 1H), 2.99 (s, 3H), 2.38 (s, 3H). ^{13}C NMR (151 MHz, $CDCl_3$) δ 160.9, 151.9, 149.6, 149.3, 143.2, 142.5, 141.6, 140.3, 133.6, 126.6, 125.0, 123.7, 123.3, 122.8, 120.9, 118.1, 117.5, 104.4, 103.5, 97.3, 93.5, 78.9, 60.8, 51.6, 33.3, 16.9, 16.8. ESI-MS calcd for $C_{29}H_{27}N_4O_6S$ $[M+H]^+$ m/z = 559.1646; Found 559.1645.

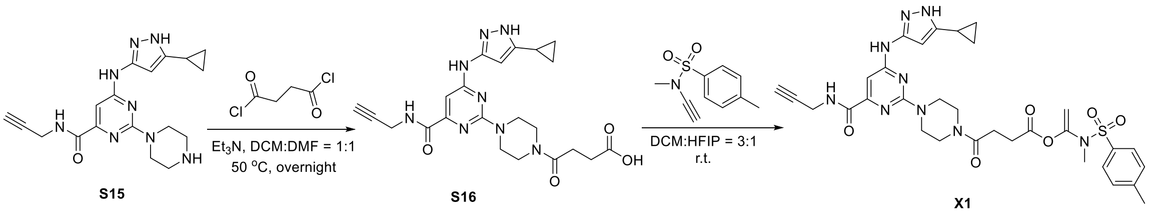

Scheme S11

S15 was synthesized according to reported procedures.[4]

4-(4-(4-((5-cyclopropyl-1*H*-pyrazol-3-yl)amino)-6-(prop-2-yn-1-ylcarbamoyl)pyrimidin-2-yl)piperazin-1-yl)-4-oxobutanoic acid (**S16**). A mixture of **S15** (102 mg, 0.28 mmol), succinoyl chloride (44 mg, 0.28 mmol) and Et_3N (28 mg, 0.28 mmol) were dissolved in 5 mL DCM and 5 mL DMF and then was stirred at 50°C overnight. The mixture was added with acetic acid (0.2 mL) and the solvent was removed under vacuum. DCM was added to the residue to form precipitate. The precipitate was filtered and dried to give compound **S16** as a white powder (81 mg, 62%). ^{1}H NMR (400 MHz, DMSO-d_6) δ 12.11 – 12.00 (m, 2H), 9.81 (br, 1H), 8.95 (t, *J* = 6.1 Hz, 1H), 6.85 (br, 1H),6.16 (br, 1H), 4.02 (m, 2H), 3.81 (d, *J* = 23.7 Hz, 4H), 3.55 (m, 4H), 3.10 (p, *J* = 2.6 Hz, 1H),

2.62 (dd, J = 7.4, 5.6 Hz, 2H), 2.47 (dd, J = 7.3, 5.6 Hz, 2H), 1.90 (tt, J = 8.4, 5.1 Hz, 1H), 1.01 – 0.90 (m, 2H), 0.76 – 0.61 (m, 2H).

1-((*N*,4-dimethylphenyl)sulfonamido)vinyl-4-(4-(4-((5-cyclopropyl-1*H*-pyrazol-3-yl)amino)-6-(prop-2-yn-1-ylcarbamoyl)pyrimidin-2-yl)piperazin-1-yl)-4-oxobutanoate (**X1**). The synthesis protocol is same as **E1**. The reaction mixture was purified by flash chromatography eluting with DCM: MeOH = 30:1 to give the desired product **X1** as a white solid (36 mg, 53% yield). ^{1}H NMR (400 MHz, $CDCl_3$) δ 8.37 (s, 1H), 7.98 (t, J = 5.7 Hz, 1H), 7.75 (d, J = 8.2 Hz, 2H), 7.35 (d, J = 8.0 Hz, 2H), 6.96 (s, 1H), 6.13 (s, 1H), 4.87 (d, J = 2.7 Hz, 1H), 4.59 (d, J = 2.6 Hz, 1H), 4.25 (m, 2.5 Hz, 2H), 3.85 (m, 4H), 3.74 (m, 2H), 3.61 (d, J = 5.4 Hz, 2H), 3.03 (s, 3H), 2.76 (m, 4H), 2.46 (s, 3H), 2.30 (t, J = 2.5 Hz, 1H), 1.90 (m, 1H), 1.05 – 0.92 (m, 2H), 0.76 (m, 2H).^{13}C NMR (151 MHz, $CDCl_3$) δ 170.7, 169.6, 164.1, 160.9, 147.0, 144.2, 133.6, 130.0, 129.6, 129.6, 128.0, 127.3, 100.0, 79.3, 71.7, 45.1, 43.9, 43.8, 41.6, 37.3, 29.3, 29.1, 27.9, 21.6, 7.9. ESI-MS calcd for $C_{32}H_{38}N_9O_6S$ $[M+H]^+$ m/z = 676.2666; Found 676.2670.

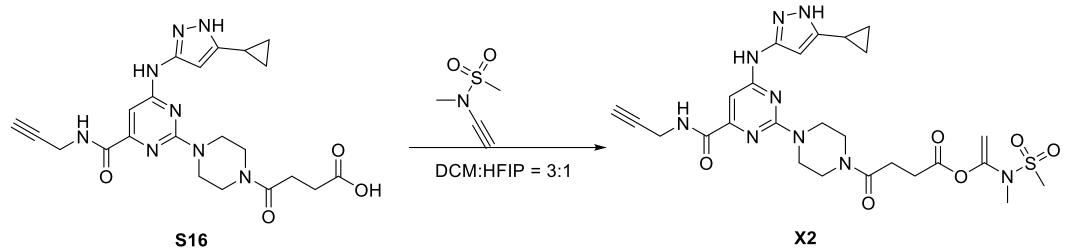

Scheme S12

1-(*N*-methylmethylsulfonamido)vinyl-4-(4-(4-((5-cyclopropyl-1*H*-pyrazol-3-yl)amino)-6-(prop-2-yn-1-ylcarbamoyl)pyrimidin-2-yl)piperazin-1-yl)-4-oxobutanoate (**X2**). The synthesis protocol is same as **E2**. The reaction mixture was purified by flash chromatography eluting with DCM: MeOH = 30:1 to give the desired product **X2** as a white solid (46 mg, 77% yield). ^{1}H NMR (400 MHz, DMSO-d_6) δ 12.04 (s, 1H), 9.82 (br, 1H), 8.95 (t, J = 6.2 Hz, 1H), 6.74 (br, 1H), 6.22 (br, 1H), 5.02 (d, J = 2.4 Hz, 1H), 4.82 (d, J = 2.4 Hz, 1H), 4.02 (d, J = 6.0 Hz, 2H), 3.82 (m, 4H), 3.56 (m, 4H), 3.10 (d, J = 2.5 Hz, 1H), 3.07 (d, J = 2.0 Hz, 3H), 3.01 (d, J = 2.0 Hz, 3H), 2.83 – 2.63 (m, 4H), 1.89 (m, 1H), 0.93 (m, 2H), 0.69 (m, 2H). ^{13}C NMR (151 MHz, DMSO-d_6) δ 171.5, 171.1, 170.4, 170.3, 169.9, 169.5, 164.1, 163.8, 161.2, 161.0, 152.4, 146.4, 100.3, 93.2, 93.2, 81.7, 73.1, 55.4, 41.0, 40.7, 37.6, 36.4, 33.0, 29.4, 28.6, 28.0, 25.0, 8.3. ESI-MS calcd for $C_{26}H_{34}N_9O_6S$ $[M+H]^+$ m/z =600.2347; Found 600.2349.

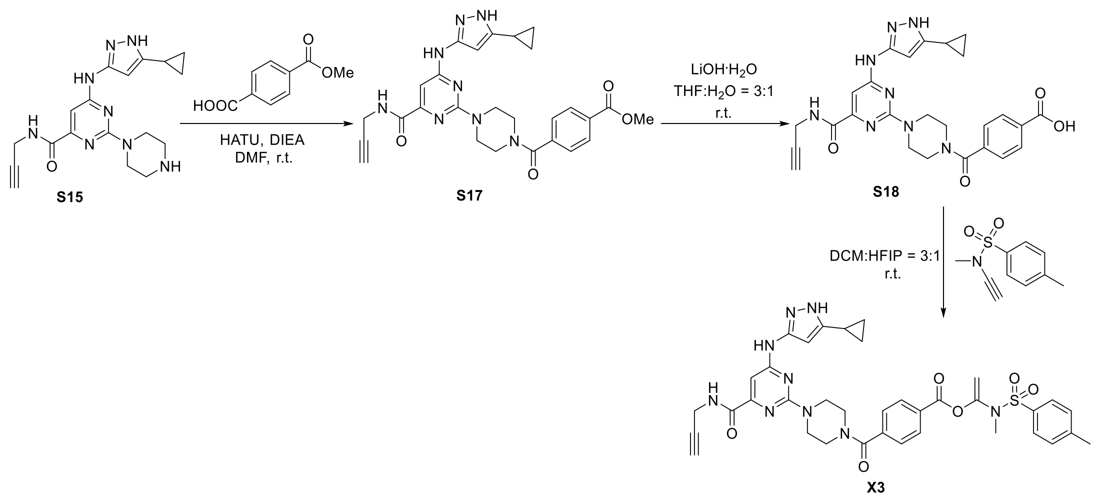

Scheme S13

Methyl 4-(4-(4-((5-cyclopropyl-1*H*-pyrazol-3-yl)amino)-6-(prop-2-yn-1-ylcarbamoyl) pyrimidin-2-yl)piperazine-1-carbonyl)benzoate (**S17**). Mono-methyl terephthalate (180 mg, 1.0 mmol), HATU (380 mg, 1.0 mmol), DIEA (323 mg, 2.5 mmol) and DMF (6 mL) were added to a round bottom flask (25 mL). The resulting mixture was stirred for 15 minutes, **S15** (366 mg, 1.0 mmol) was added to the solution and stirred for another 3 h. The mixture was added with water (20 mL) and extracted with ethyl acetate (3×10 mL). The organic layers were combined and washed with water (3×10 mL). Finally, the solvent was dried with Na_2SO_4 and evaporated under reduced pressure. The crude product was purified by flash chromatography eluting with DCM: MeOH = 30:1 to give the product **S17** as a white solid (119 mg, 23% yield). ^{1}H NMR (400 MHz, DMSO-d_6) δ 12.09 (s, 1H), 9.83 (s, 1H), 8.95 (t, *J* = 6.2 Hz, 1H), 8.05 (d, *J* = 8.2 Hz, 2H), 7.61 (d, *J* = 7.9 Hz, 2H), 6.83 (br, 1H), 6.17 (br, 1H), 4.01 (d, *J* = 6.0 Hz, 2H), 3.82 (m, 9H), 3.38 (s, 2H), 3.09 (s, 1H), 1.88 (m, 1H), 0.90 (m, 2H), 0.67 (m, 2H).

4-(4-(4-((5-cyclopropyl-1*H*-pyrazol-3-yl)amino)-6-(prop-2-yn-1-ylcarbamoyl)pyrimidin-2-yl)piperazine-1-carbonyl)benzoic acid (**S18**). The synthesis protocol is same as **S9,** product **S18** was afforded as a white solid (150 mg, 73% yield).

1-((*N*,4-dimethylphenyl)sulfonamido)vinyl-4-(4-(4-((5-cyclopropyl-1*H*-pyrazol-3-yl)amino)-6-(prop-2-yn-1-ylcarbamoyl)pyrimidin-2-yl)piperazine-1-carbonyl)benzoate (**X3**). The synthesis protocol is same as **E1**. The reaction mixture was purified by flash chromatography eluting with DCM/MeOH 30:1 to give the desired product **X3** as a white solid (28 mg, 38% yield). ^{1}H NMR (400 MHz, DMSO-d_6) δ 12.02 (s, 1H), 9.84 (s, 1H), 8.94 (s, 1H), 8.00 – 7.82 (m, 2H), 7.79 – 7.68 (m, 2H), 7.66 – 7.54 (m, 2H), 7.41 (d, *J* = 8.1 Hz, 2H), 6.81 (s, 1H), 6.20 (s, 1H), 5.07 (d, *J* = 2.9 Hz, 1H), 4.90 (d, *J* = 2.9 Hz, 1H), 4.28 – 3.58 (m, 8H), 3.40 (s, 2H), 3.07 (d, *J* = 18.0 Hz, 4H), 2.38 (s, 3H), 1.89 (m, 1H), 0.90 (d, *J* = 8.1 Hz, 2H), 0.67 (h, *J* = 4.5, 4.1 Hz, 2H).^{13}C NMR (151 MHz, DMSO-d_6) δ 168.5, 164.0, 163.3, 161.2, 146.8, 144.7, 141.7, 133.8, 130.4, 130.3, 130.3, 129.3, 128.0, 127.9, 101.4, 93.4, 81.7, 73.1, 37.8, 32.0, 31.6, 30.8, 28.6, 22.6, 21.5, 14.4, 8.3. ESI-MS calcd for $C_{36}H_{38}N_9O_6S$ $[M+H]^+$ m/z = 724.2660; Found 724.2657.

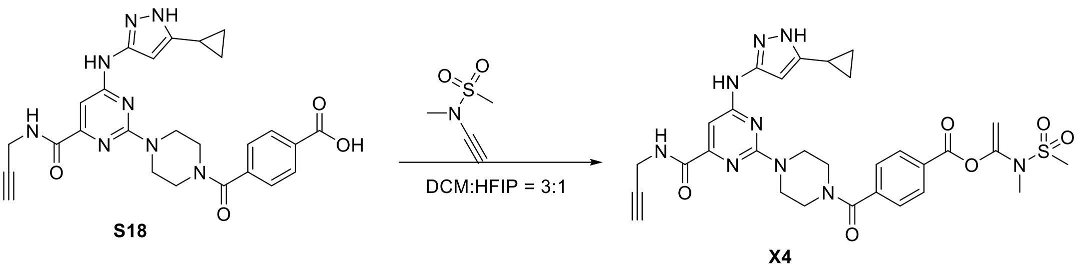

Scheme S14

1-(*N*-methylmethylsulfonamido)vinyl-4-(4-(4-((5-cyclopropyl-1*H*-pyrazol-3-yl)amino)-6-(prop-2-yn-1-ylcarbamoyl)pyrimidin-2-yl)piperazine-1-carbonyl)benzoate (**X4**). The synthesis protocol is same as **E2**. The reaction mixture was purified by flash chromatography eluting with DCM: MeOH = 30:1 to give the desired product **X4** as a white solid (47 mg, 49% yield). ^{1}H NMR (400 MHz, DMSO-d_6) δ 12.02 (s, 1H), 9.85 (s, 1H), 8.94 (s, 1H), 8.12 (d, *J* = 7.9 Hz, 2H), 7.67 (d, *J* = 7.9 Hz, 2H), 6.89 (s, 1H), 6.16 (s, 1H), 5.20 (t, *J* = 2.2 Hz, 1H), 5.09 (d, *J* = 2.5 Hz, 1H), 4.12 – 3.59 (m, 8H), 3.3 (s,2H),3.20 – 2.96 (m, 7H), 1.89 (s, 1H), 0.89 (t, *J* = 12.2 Hz, 2H), 0.67 (d, *J* = 5.2 Hz, 2H).^{13}C NMR (151 MHz, DMSO-d_6) δ 168.5, 164.0, 163.6, 161.2, 147.0, 141.8, 130.6, 129.5, 128.0, 100.6, 81.7, 73.1, 49.1, 44.2, 43.7, 42.0, 37.2, 37.0, 28.6, 25.0, 22.6, 14.4, 8.3. ESI-MS calcd for $C_{30}H_{34}N_9O_6S$ $[M+H]^+$ m/z = 648.2347; Found 648.2345.

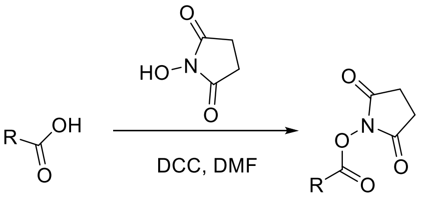

General Procedure A. Commercially available natural products were dissolved in 5 mL of anhydrous DMF. 1.5 equivalents of NHS and 1.1 equivalents of DCC were added and was stirred overnight. The material was consumed up to 90% by TLC analysis after 14 h. The mixture was filtered, concentrated and purified by flash column chromatography eluting with DCM/ MeOH, and the rection product was obtained as a white solid.

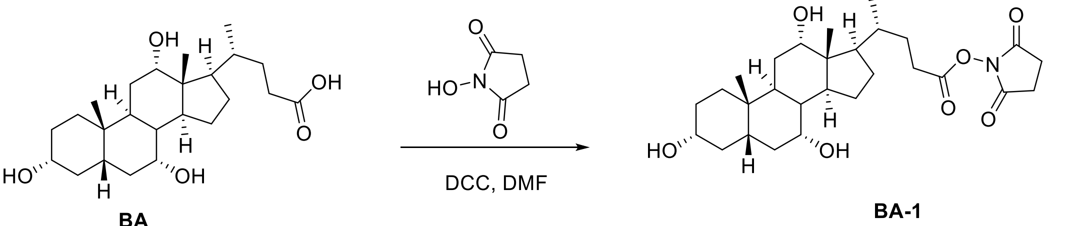

Scheme S15

(**BA-1**) This compound was synthesized according to General Procedure A**,** product **BA-1** was afforded as a white solid (493 mg, 85% yield). ^{1}H NMR (400 MHz, DMSO-d_6) δ 4.68 (s, 1H), 4.55 (s, 1H), 2.97 (s, 2H), 2.50 (s, 3H), 2.27 (s, 1H), 2.12 (d, *J* = 11.8 Hz, 1H), 1.80 (d, *J* = 6.7 Hz, 2H), 1.64 (s, 3H), 1.37 (m, 14H), 0.92 (s, 3H), 0.87 (s, 6H), 0.76 (s, 3H), 0.65 (s, 3H). ^{13}C NMR (151 MHz, DMSO-d_6) δ 170.64, 169.76, 71.24 (d, *J* = 87.4 Hz), 70.95, 66.75, 55.31, 46.43, 46.33 , 41.94,

41.86, 35.80, 35.27, 35.27, 34.84, 31.04, 30.89, 28.95, 28.08, 27.68, 26.68, 25.90, 23.16, 23.06, 17.19, 12.74. HRMS (ESI) calcd for $C_{28}H_{43}NO_7$ $[M+H]^+$ = 506.3112; Found 506.3132.

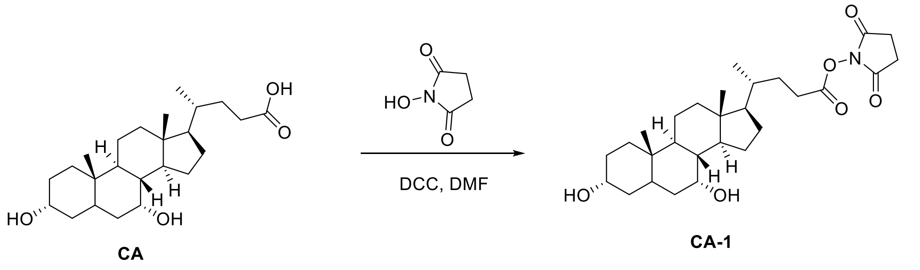

Scheme S16

(**CA-1**) This compound was synthesized according to General Procedure A, product **CA-1** was afforded as a white solid (479 mg, 85% yield). ^{1}H NMR (400 MHz, DMSO-d_6) δ 4.31 (d, J = 4.7 Hz, 1H), 4.10 (d, J = 3.2 Hz, 1H), 3.63 (s, 1H), 3.19 (d, J = 4.5 Hz, 1H), 2.81 (s, 4H), 2.55 (d, J = 36.2 Hz, 2H), 2.20 (d, J = 12.2 Hz, 1H), 1.79 (m, 8H), 1.51 – 1.09 (m, 17H), 0.93 (d, J = 6.4 Hz, 3H), 0.84 (s, 3H), 0.62 (s, 3H).^{13}C NMR (151 MHz, DMSO-d_6) δ 170.67, 169.76, 70.82, 66.64, 55.89, 50.46, 42.47, 41.93, 35.80, 35.30, 35.20, 35.14, 32.76, 31.04, 30.97, 28.16, 28.01, 25.91, 23.63, 23.18, 20.74, 18.43, 12.09. HRMS (ESI) calcd for $C_{28}H_{43}NO_6$ $[M+Na]^+$ = 512.2983; Found 512.2996.

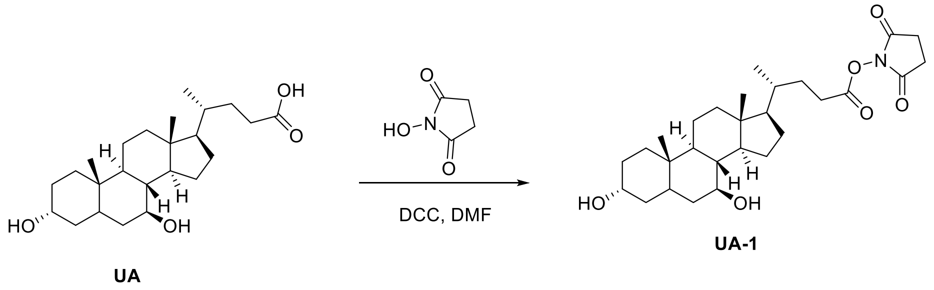

Scheme S17

(**UA-1**) This compound was synthesized according to General Procedure A, product **UA-1** was afforded as a white solid (479 mg, 85% yield). ^{1}H NMR (400 MHz, DMSO-d_6) δ 4.44 (d, J = 4.6 Hz, 1H), 3.87 (d, J = 6.8 Hz, 1H), 3.29 (d, J = 9.2 Hz, 2H), 2.81 (s, 4H), 2.51 (s, 2H), 2.01 – 1.61 (m, 6H), 1.52 – 0.99 (m, 18H), 0.93 (d, J = 6.4 Hz, 3H), 0.88 (s, 3H), 0.63 (s, 3H). ^{13}C NMR (151 MHz, DMSO-d_6) δ 170.68, 169.76, 70.20, 69.92, 56.29, 55.02, 43.63, 43.50, 42.66, 39.19, 38.19, 37.75, 35.18, 35.04, 34.23, 30.88, 30.73, 28.53, 28.01, 27.17, 25.91, 23.79, 21.33, 18.58. HRMS (ESI) calcd for $C_{28}H_{43}NO_6$ $[M+Na]^+$ = 512.2983; Found 512.2996.

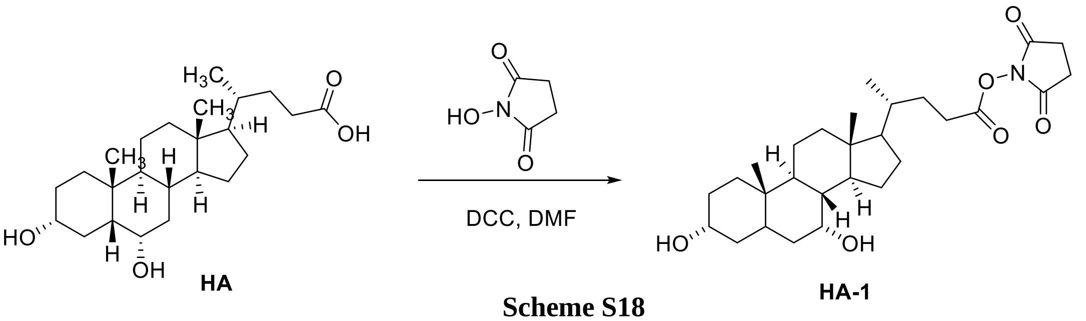

Scheme S18

(**HA-1**) This compound was synthesized according to General Procedure A, product **HA-1** was afforded as a white solid (479 mg, 85% yield). ^{1}H NMR (400 MHz, DMSO) δ 4.41 (s, 1H), 4.23 (s, 1H), 3.83 (s, 1H), 3.31 – 3.24 (m, 1H), 2.80 (s, 4H), 2.72 – 2.44 (m, 2H), 2.00 – 1.59 (m, 6H), 1.56 – 0.97 (m,18H), 0.91 (d, *J* = 5.1 Hz, 3H), 0.84 (s, 3H), 0.62 (s, 3H). ^{13}C NMR (151 MHz, DMSO) δ 170.67, 169.74, 70.49, 66.37, 56.33, 55.83, 48.74, 42.91, 36.02, 36.02, 35.87, 35.35, 35.01, 34.81, 30.88, 30.84, 29.73, 28.06, 27.97, 25.90, 24.35, 24.02, 20.89, 18.39, 12.30. HRMS (ESI) calcd for $C_{28}H_{43}NO_6$ 490.3163 [M+H]$^+$, found 490.3177.

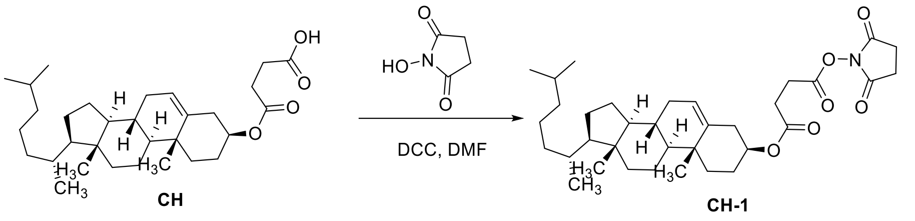

Scheme S19

(**CH-1**) This compound was synthesized according to General Procedure A, product **CH-1** was afforded as a white solid (428 mg, 65% yield). ^{1}H NMR (400 MHz, $CDCl_3$) δ 5.39 (d, *J* = 4.2 Hz, 1H), 4.75 – 4.60 (m, 1H), 2.97 (t, *J* = 7.0 Hz, 2H), 2.86 (s, 4H), 2.74 (t, *J* = 7.0 Hz, 2H), 2.35 (d, *J* = 7.7 Hz, 2H), 2.09 – 1.92 (m, 2H), 1.93 – 1.79 (m, 2H), 1.68 – 1.47 (m, 8H), 1.46 – 1.31 (m, 4H), 1.28 (t, *J* = 8.2 Hz, 2H), 1.15 (m, 6H), 1.04 (s, 3H), 0.93 (d, *J* = 6.5 Hz, 2H), δ 0.93 (d, *J* = 6.5 Hz, 3H), 0.89 (dd, *J* = 6.6, 1.7 Hz, 6H), 0.70 (s, 3H). ^{13}C NMR (151 MHz, $CDCl_3$) δ 170.40, 168.98, 167.85, 139.49, 122.82, 74.88, 56.66, 56.08, 49.95, 42.30, 39.70, 39.52, 37.94, 36.93, 36.57, 36.18, 35.82, 31.91, 31.78 – 31.68 (m), 29.04, 28.27, 28.05, 27.62, 26.34, 25.59, 24.30, 23.84, 22.88, 22.61, 21.02, 19.33, 18.73, 11.87. HRMS (ESI) calcd for $C_{35}H_{53}NO_6$ [M+Na]$^+$ = 606.3765; Found 606.3768.

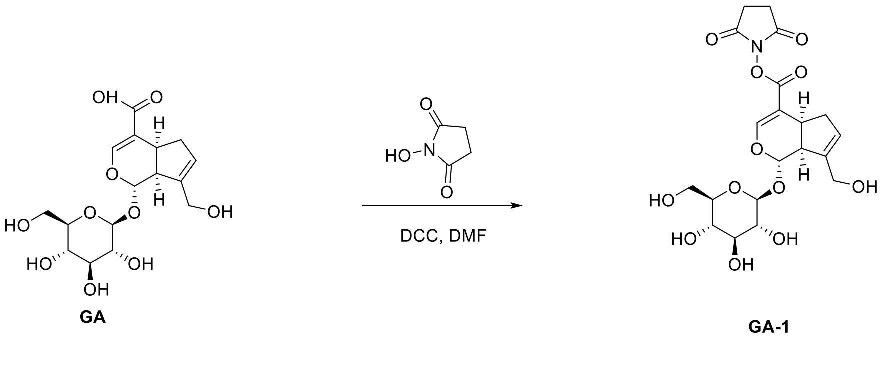

Scheme S20

(**GA-1**) This compound was synthesized according to General Procedure A, product **GA-1** was afforded as a white solid (437 mg, 80% yield). ^{1}H NMR (400 MHz, DMSO-d_6) δ 7.84 (s, 1H), 5.29 (d, *J* = 6.6 Hz, 1H), 5.14 (s, 1H), 5.06 – 4.92 (m, 2H), 4.78 (s, 1H), 4.57 (d, *J* = 7.5 Hz, 1H), 4.48 (s, 1H), 4.14 (t, *J* = 12.5 Hz, 2H), 4.02 (s, 1H), 3.65 (d, *J* = 5.9 Hz, 1H), 3.46 – 3.42 (m, 1H), 3.15 (d, *J* = 10.1 Hz, 4H), 3.08 – 2.98 (m, 2H), 2.82 (s, 4H), 2.78 – 2.64 (m, 1H), 2.19 (d, *J* = 15.9 Hz, 1H).^{13}C NMR (151 MHz, DMSO-d_6) δ 171.10, 162.71, 156.31, 144.32, 125.91, 106.84, 99.20, 96.72, 77.84, 77.05, 73.71, 70.34, 61.42, 59.72, 49.07, 46.14, 34.59, 25.89. HRMS (ESI) calcd for $C_{20}H_{25}NO_{12}$ [M+Na]$^+$ = 494.1269; Found 494.126.

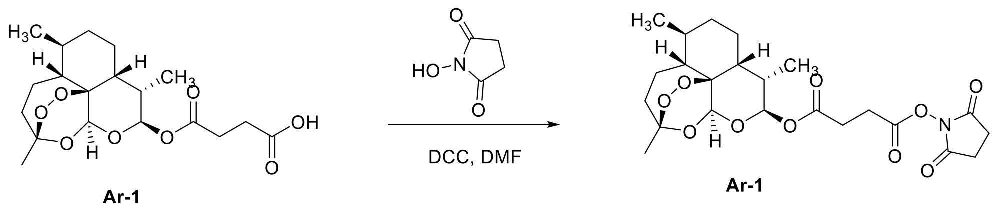

Scheme S21

(**Ar-1**) This compound was synthesized according to General Procedure A, product **Ar-1** was afforded as a white solid (445 mg, 80% yield). ^{1}H NMR (400 MHz, DMSO-d_6) δ 5.70 (d, *J* = 9.6 Hz, 1H), 5.59 (s, 1H), 2.98 (s, 2H), 2.82 (s, 5H), 2.32 (s, 1H), 2.20 (t, *J* = 13.6 Hz, 1H), 2.01 (d, *J* = 10.4 Hz, 2H), 1.81 (s, 1H), 1.68 – 1.39 (m, 5H), 1.30 (s, 4H), 1.19 (t, *J* = 6.2 Hz, 2H), 0.90 (d, *J* = 5.4 Hz, 3H), 0.78 (d, *J* = 6.5 Hz, 3H).^{13}C NMR (151 MHz, DMSO-d_6) δ 170.66, 170.56, 168.83, 104.05, 92.48, 91.08, 80.32, 60.25, 51.55, 45.01, 36.40, 36.32, 34.16, 32.06, 28.48, 25.96, 25.88, 20.53, 12.21. HRMS (ESI) calcd for $C_{23}H_{31}NO_{10}$ [M+Na]$^+$ = 504.1840; Found 504.1844.

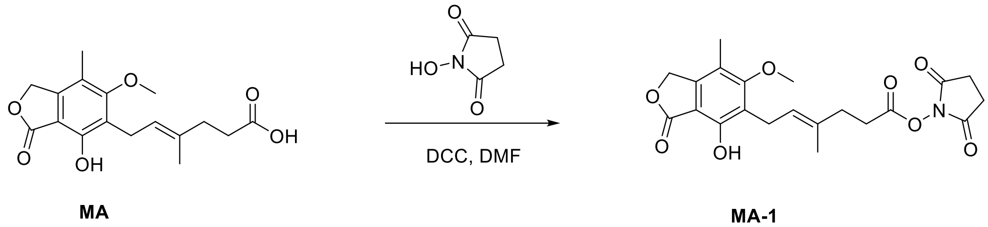

Scheme S22

(**MA-1**) This compound was synthesized according to General Procedure A, product **MA-1** was afforded as a white solid (394 mg, 80% yield). ^{1}H NMR (400 MHz, CDCl$_3$) δ 7.68 (s, 1H), 5.31 (s, 1H), 5.21 (s, 2H), 3.78 (s, 3H), 3.41 (d, *J* = 6.9 Hz, 2H), 2.83 (s, 4H), 2.70 (d, *J* = 8.3 Hz, 2H), 2.42 (t, *J* = 7.8 Hz, 2H), 2.16 (s, 3H), 1.84 (s, 3H).^{13}C NMR (151 MHz, CDCl$_3$) δ 172.91, 170.80, 163.62, 153.54, 145.88, 144.19, 133.56, 123.09, 121.87, 116.83, 106.42, 99.34, 70.10, 61.01, 37.37, 35.67, 34.11, 32.57, 22.60, 16.20, 11.58. HRMS (ESI) calcd for $C_{21}H_{23}NO_8$ [M+Na]$^+$ = 418.1496; Found 418.1497.

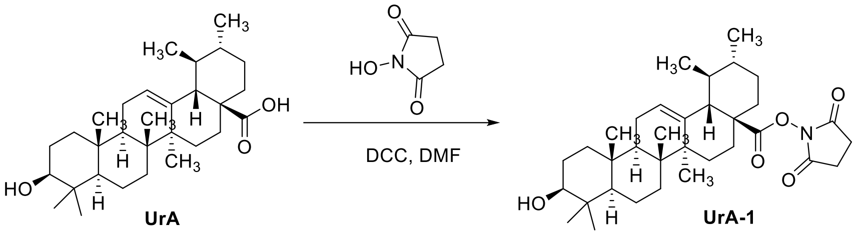

Scheme S23

(**UrA-1**) This compound was synthesized according to General Procedure A, product **UrA-1** was afforded as a white solid (408 mg, 65% yield). ^{1}H NMR (400 MHz, DMSO-d_6) δ 5.57 (d, *J* = 7.8 Hz, 1H), 5.20 (s, 1H), 4.29 (d, *J* = 5.1 Hz, 1H), 3.01 (dd, *J* = 10.3, 5.0 Hz, 1H), 2.76 (d, *J* = 4.4 Hz, 4H), 2.18 (t, *J* = 12.2 Hz, 2H), 1.94 – 1.82 (m, 3H), 1.72 (d, *J* = 13.8 Hz, 3H), 1.63 – 1.39 (m, 10H), 1.35 – 1.20 (m, 5H), 1.08 (s, 3H), 0.93 (d, *J* = 5.7 Hz, 3H), 0.90 (s, 3H), 0.87 (s, 3H), 0.83 (d, *J* = 6.4 Hz, 3H), 0.74 (s, 2H), 0.68 (s, 3H). ^{13}C NMR (151 MHz, DMSO-d_6) δ 172.38, 157.08, 137.11, 126.28, 77.28, 60.23, 55.26, 53.03, 48.42, 47.49, 42.35, 38.85, 38.83, 38.71, 38.63, 36.94, 33.81, 33.36, 30.39, 28.71, 28.22, 27.46, 25.88, 25.79, 24.93, HRMS (ESI) calcd.for $C_{34}H_{51}NO_5$ [M+Na]$^+$ = 576.3659; Found 576.3675.

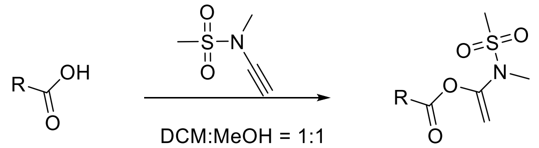

General Procedure B. Commercially available natural products were dissolved in DCM/MeOH (1:1), followed by the addition of MYTsA (1.5 mmol). The reaction mixture was purified by flash column chromatography with DCM/MeOH to give the desired product as a white solid.

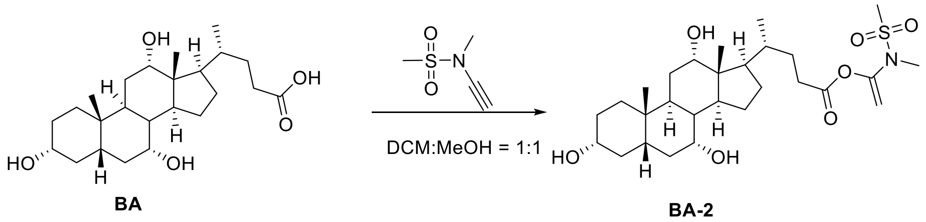

Scheme S24

(**BA-2**). This compound was synthesized according to General Procedure B, product **BA-2** was afforded as a white solid (455 mg, 75% yield). ^{1}H NMR (400 MHz, DMSO-d_6) δ 5.00 (s, 1H), 4.81 (s, 1H), 4.31 (s, 1H), 4.12 (s, 1H), 4.01 (s, 1H), 3.79 (s, 1H), 3.61 (s, 1H), 3.06 (d, *J* = 2.6 Hz, 3H), 3.01 (d, *J* = 2.6 Hz, 3H), 2.47 (d, *J* = 15.9 Hz, 1H), 2.41 – 2.27 (m, 1H), 2.26 – 2.09 (m, 2H), 1.99 (q, *J* = 11.2 Hz, 1H), 1.72 (m, 6H), 1.52 – 1.09 (m, 14H), 0.94 (d, *J* = 6.0 Hz, 3H), 0.88 – 0.78 (m, 3H), 0.59 (d, *J* = 2.6 Hz, 3H). ^{13}C NMR (151 MHz, DMSO-d_6) δ 171.75, 146.79, 99.49, 71.47, 70.92, 66.72, 46.45, 46.27, 42.01, 41.86, 37.26, 36.76, 35.34, 34.86, 30.89, 30.84, 30.76, 29.00, 27.70, 26.69, 23.26, 23.09, 17.37, 12.79. HRMS (ESI) calcd for $C_{28}H_{47}NO_7S$ $[M+H]^+$ = 542.3146; Found 542.3195.

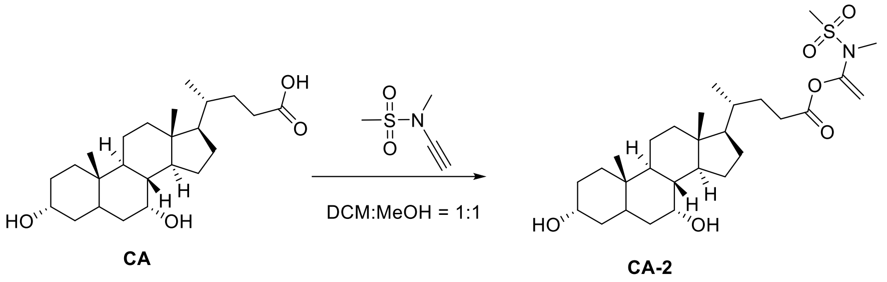

Scheme S25

(**CA-2**). This compound was synthesized according to General Procedure B, product C**A-2** was afforded as a white solid (443 mg, 75% yield). ^{1}H NMR (400 MHz, DMSO-d_6) δ 5.00 (t, *J* = 2.5 Hz, 1H), 4.81 (t, *J* = 2.4 Hz, 1H), 4.11 (s, 1H), 3.63 (s, 1H), 3.23 – 3.13 (m, 1H), 3.06 (d, *J* = 3.6 Hz, 3H), 3.01 (d, *J* = 3.3 Hz, 3H), 2.42 – 2.29 (m, 1H), 2.19 (d, *J* = 12.5 Hz, 1H), 1.97 – 1.59 (m, 8H), 1.54 – 1.04 (m, 17H), 0.94 – 0.87 (m, 3H), 0.84 (d, *J* = 3.3 Hz, 3H), 0.61 (d, *J* = 3.9 Hz, 3H). ^{13}C NMR (151 MHz, DMSO-d_6) δ 171.69, 146.80, 99.50, 70.82, 66.64, 55.90, 50.47, 42.43, 41.90, 37.25, 36.77, 35.78, 35.29, 35.24, 35.20, 32.76, 30.78, 30.68, 28.20, 23.62, 23.18, 20.73, 18.58, 12.11. HRMS (ESI) calcd for $C_{28}H_{47}NO_6S$ $[M+Na]^+$ = 548.3016; Found 548.3029.

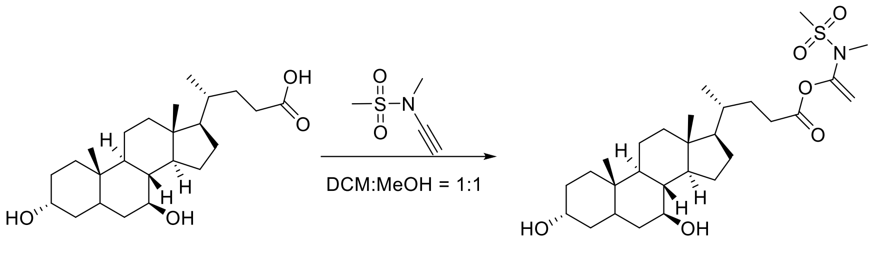

UA **UA-2**

Scheme S26

(**UA-2**). This compound was synthesized according to General Procedure B, product **UA-2** was afforded as a white solid, (443 mg, 75% yield). ^{1}H NMR (400 MHz, DMSO-d_6) δ 5.00 (d, *J* = 2.8 Hz, 1H), 4.81 (d, *J* = 2.8 Hz, 1H), 4.47 (s, 1H), 3.88 (d, *J* = 5.9 Hz, 1H), 3.30 (s, 1H), 3.05 (d, *J* = 3.7 Hz, 2H), 3.01 (d, *J* = 2.8 Hz, 3H), 2.37 (s, 3H), 2.00 – 1.60 (m, 6H), 1.54 – 0.98 (m, 18H), 0.90 (dd, *J* = 6.8, 2.8 Hz, 3H), 0.87 (d, *J* = 3.5 Hz, 3H), 0.62 (d, *J* = 3.3 Hz, 3H). ^{13}C NMR (151 MHz, DMSO-d_6) δ 171.69, 146.79, 99.52, 70.20, 69.93, 56.31, 55.05, 43.59, 43.48, 42.66, 39.20, 38.18, 37.73, 37.24, 36.77, 35.31, 35.15, 34.23, 30.81, 30.73, 30.70, 28.59, 27.16, 23.78, 21.33, 18.72, 12.50. HRMS (ESI) calcd for $C_{28}H_{47}NO_6S$ $[M+Na]^+$ = 548.3016; Found 548.3025.

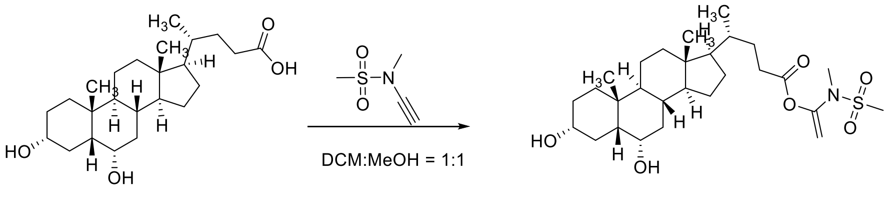

HA **HA-2**

Scheme S27

(**HA-2**). This compound was synthesized according to General Procedure B, product **HA-2** was afforded as a white solid (443 mg, 75% yield). ^{1}H NMR (400 MHz, DMSO-d_6) δ 5.00 (d, *J* = 2.5 Hz, 1H), 4.81 (d, *J* = 2.6 Hz, 1H), 4.41 (s, 1H), 4.23 (s, 1H), 3.83 (m, 1H), 3.32 (m, 1H), 3.06 (s, 3H), 3.01 (s, 3H), 2.36 (m, 2H), 2.01 – 1.59 (m, 6H), 1.57 – 0.94 (m, 18H), 0.90 (d, *J* = 6.5 Hz, 3H), 0.84 (s, 3H), 0.61 (s, 3H). ^{13}C NMR (151 MHz, DMSO-d_6) δ 171.67, 146.81, 99.52, 70.48, 66.36, 56.34, 55.86, 48.74, 42.86, 37.25, 36.79, 36.01, 35.87, 35.35, 35.12, 34.82, 30.84, 30.76, 30.62, 29.73, 28.10, 24.34, 24.02, 20.88, 18.55, 12.33. HRMS (ESI) calcd for $C_{28}H_{47}NO_6S$ $[M+Na]^+$ = 548.3016; Found 548.3019.

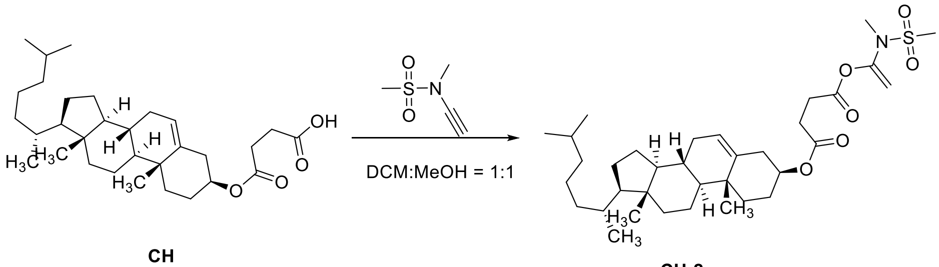

CH **CH-2**

Scheme S28

(**CH-2**). This compound was synthesized according to General Procedure B, product **CH-2** was afforded as a white solid (445 mg, 65% yield). ^{1}H NMR (400 MHz, $CDCl_3$) δ 5.38 (d, *J* = 4.2

Hz, 1H), 5.04 (d, J = 2.5 Hz, 1H), 4.85 (d, J = 2.5 Hz, 1H), 4.71 – 4.47 (m, 1H), 3.12 (s, 3H), 3.01 (s, 3H), 2.76 (t, J = 6.3 Hz, 2H), 2.69 – 2.64 (m, 2H), 2.33 (d, J = 7.6 Hz, 2H), 2.00 (t, J = 15.3 Hz, 2H), 1.93 – 1.78 (m, 3H), 1.68 – 1.41 (m, 8H), 1.40 – 1.33 (m, 3H), 1.14 (m, 6H), 1.03 (s, 4H), 0.93 (d, J = 6.5 Hz, 3H), 0.88 (dd, J = 6.6, 1.5 Hz, 6H), 0.69 (s, 3H). ^{13}C NMR (151 MHz, Chloroform-*d*) δ 171.43, 170.30, 145.68, 139.44, 122.87, 100.01, 74.68, 56.66, 56.08, 49.96, 42.30, 39.69, 39.52, 38.07, 37.58, 36.92, 36.58, 36.18, 35.84, 35.82, 31.90, 31.82, 29.07, 28.94, 28.26, 28.05, 27.72, 24.30, 23.83, 22.89, 22.62, 21.02, 19.34, 18.73, 11.88. HRMS (ESI) calcd for $C_{35}H_{57}NO_6S$ [M+Na]$^+$ = 642.3799; Found 642.3799.

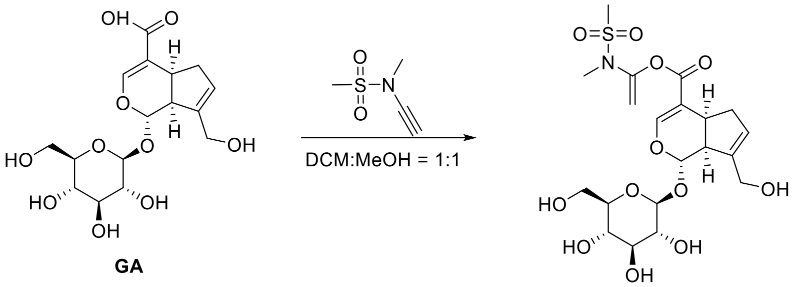

Scheme S29

(**GA-2**). This compound was synthesized according to General Procedure B, product **GA-2** was afforded as a white solid (487 mg, 85% yield). ^{1}H NMR (400 MHz, DMSO-d_6) δ 7.66 (s, 1H), 5.75 (d, J = 2.5 Hz, 1H), 5.70 (s, 1H), 5.19 (d, J = 6.9 Hz, 1H), 5.08 (t, J = 3.7 Hz, 1H), 5.03 (s, 1H), 5.00 (d, J = 3.6 Hz, 1H), 4.96 (dd, J = 5.1, 2.5 Hz, 1H), 4.87 (s, 1H), 4.74 (d, J = 6.0 Hz, 1H), 4.55 (d, J = 7.8 Hz, 1H), 4.47 (d, J = 6.3 Hz, 1H), 4.14 (d, J = 15.2 Hz, 1H), 3.99 (d, J = 15.5 Hz, 1H), 3.75 – 3.52 (m, 1H), 3.48 – 3.37 (m, 1H), 3.14 (m, 1H), 3.06 (d, J = 2.4 Hz, 3H), 3.02 (d, J = 2.4 Hz, 6H), 2.83 – 2.64 (m, 2H), 2.11 (d, J = 16.7 Hz, 1H). ^{13}C NMR (151 MHz, DMSO-d_6) δ 164.67 (s), 154.57 (s), 146.64 (s), 144.39 (s), 126.05 (s), 110.30 (s), 100.26 (s), 99.08 (s), 96.50 (s), 77.78 (s), 76.86 , 76.25 (m), 73.73 (s), 70.40 (s), 61.43 (s), 59.76 (s), 46.27 (s), 37.09 (s), 36.72 (s), 34.77 (s). HRMS (ESI) calcd for $C_{20}H_{29}NO_{12}S$ [M+Na]$^+$ = 530.1303; Found 530.1299.

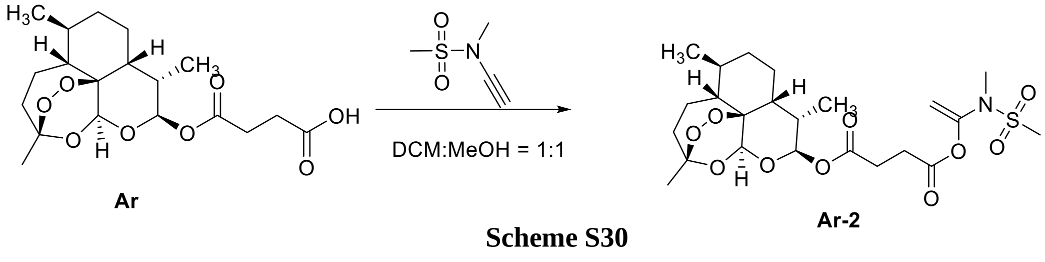

Scheme S30

(**Ar-2**). This compound was synthesized according to General Procedure B, product **Ar-2** was afforded as a white solid (495 mg, 85% yield). ^{1}H NMR (400 MHz, DMSO-d_6) δ 5.68 (d, J = 9.6 Hz, 1H), 5.57 (d, J = 4.3 Hz, 1H), 5.03 (d, J = 2.9 Hz, 1H), 4.84 (d, J = 3.0 Hz, 1H), 3.06 (d, J = 2.0 Hz, 3H), 3.00 (d, J = 2.1 Hz, 3H), 2.75 (p, J = 3.3 Hz, 4H), 2.35 – 2.25 (m, 1H), 2.19 (t, J = 13.5 Hz, 1H), 2.00 (d, J = 9.4 Hz, 2H), 1.89 – 1.75 (m, 1H), 1.60 (d, J = 14.4 Hz, 3H), 1.44 (t, J = 11.8 Hz, 2H), 1.29 (s, 3H), 1.24 (s, 2H), 0.89 (d, J = 6.5 Hz, 3H), 0.77 (d, J = 7.1 Hz, 3H). ^{13}C NMR (151 MHz, DMSO-d_6) δ 171.20, 170.40, 146.32, 104.06, 100.33, 92.44, 91.09, 80.33, 51.59, 45.03, 37.48,

36.46, 36.41, 36.36, 34.17, 32.09, 28.88, 25.97, 24.66, 21.46, 20.53, 12.21. HRMS (ESI) calcd for $C_{23}H_{35}NO_{10}S$ $[M+Na]^+$ = 540.1874; Found 540.1878.

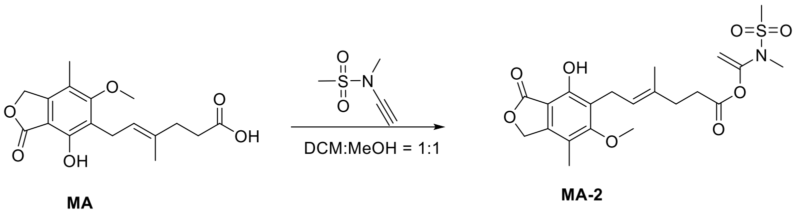

Scheme S31

(**MA-2**). This compound was synthesized according to General Procedure B, product **MA-2** was afforded as a white solid (415 mg, 80% yield). ^{1}H NMR (400 MHz, $CDCl_3$) δ 7.67 (s, 1H), 5.25 (t, J = 7.1 Hz, 1H), 5.19 (s, 2H), 4.95 (d, J = 2.5 Hz, 1H), 4.72 (d, J = 2.6 Hz, 1H), 3.75 (s, 3H), 3.38 (d, J = 7.0 Hz, 2H), 3.02 (s, 3H), 2.94 (s, 3H), 2.54 (t, J = 7.6 Hz, 2H), δ 2.32 (t, J = 7.6 Hz, 2H). 2.39 – 2.28 (m, 3H), 1.81 (s, 3H). ^{13}C NMR (151 MHz, $CDCl_3$) δ 172.91, 168.96, 163.65, 153.54, 144.13, 133.31, 123.28, 121.87, 116.81, 106.34, 70.08, 61.02, 41.28, 33.93, 33.74, 32.85, 24.69, 22.58, 16.13, 11.56. HRMS (ESI) calcd for $C_{21}H_{27}NO_8S$ $[M+H]^+$ = 454.1530; Found 454.1535.

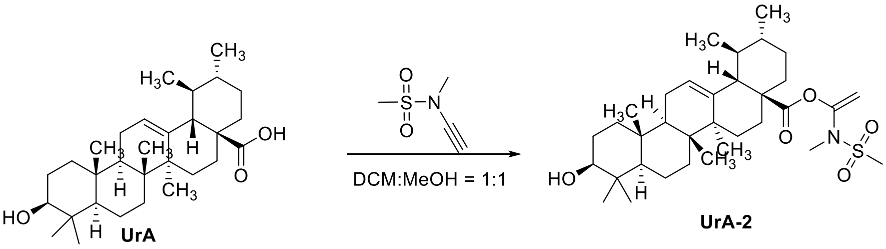

Scheme S32

(**UrA-2**). This compound was synthesized according to General Procedure B, product **UrA-2** was afforded as a white solid (557 mg, 85% yield). ^{1}H NMR (400 MHz, $CDCl_3$) δ 5.31 (s, 2H), 5.08 (s, 1H), 4.81 (s, 1H), 3.31 – 3.17 (m, 1H), 3.08 (s, 3H), 3.00 (s, 3H), 2.29 – 1.29 (m, 23H), 1.12 (s, 3H), 1.07 – 0.93 (m, 9H), 0.88 (d, J = 6.2 Hz, 3H), 0.84 (s, 3H), 0.80 (s, 3H). ^{13}C NMR (151 MHz, $CDCl_3$) δ 175.26, 146.37, 137.56, 126.25, 101.05, 79.00, 55.22, 53.46, 52.94, 48.78, 47.51, 42.25, 39.69, 39.13, 38.80, 38.66, 37.12, 36.98, 36.46, 35.77, 33.22, 30.56, 28.16, 27.99, 27.22, 24.31, 23.38, 23.31, 21.12, 18.30, 17.60, 16.96, 15.65, 15.52. HRMS (ESI) calcd for $C_{34}H_{55}NO_5S$ $[M+H]^+$ = 590.3874; Found 590.3885.

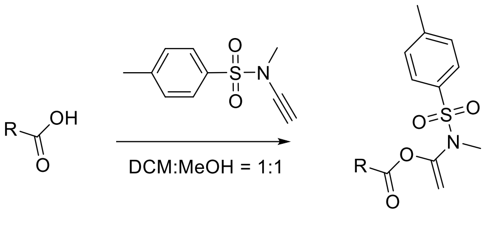

General Procedure C. Commercially available natural products were dissolved in DCM/MeOH (1:1), followed by the addition of MYTsA (1.5 mmol). The reaction mixture was purified by flash column chromatography with DCM/MeOH to give the desired product as a solid.

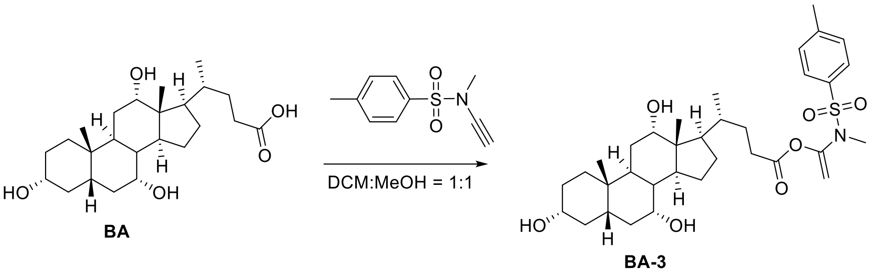

Scheme S33

(**BA-3**). This compound was synthesized according to General Procedure C, product **BA-3** was afforded as a white solid (613 mg, 85% yield). ^{1}H NMR (400 MHz, DMSO-d_6) δ 7.68 (d, *J* = 7.8 Hz, 2H), 7.45 (d, *J* = 7.9 Hz, 2H). δ 5.00 (s, 1H), 4.81 (s, 1H), 4.31 (s, 1H), 4.12 (s, 1H), 4.01 (s, 1H), 3.79 (s, 1H), 3.61 (s, 1H), 3.06 (d, *J* = 2.6 Hz, 3H), 3.01 (d, *J* = 2.6 Hz, 3H), 2.47 (d, *J* = 15.9 Hz, 1H), 2.41 – 2.27 (m, 1H), 2.26 – 2.09 (m, 2H), 1.99 (q, *J* = 11.2 Hz, 1H), 1.72 (m, 6H), 1.52 – 1.09 (m, 14H), 0.94 (d, *J* = 6.0 Hz, 3H), 0.88 – 0.78 (m, 3H), 0.59 (d, *J* = 2.6 Hz, 3H). ^{13}C NMR (151 MHz, DMSO-d_6) δ 171.45, 146.73, 144.59, 134.26, 130.24, 127.97, 100.08, 71.47, 70.93, 66.73, 55.36, 46.42, 46.25, 42.02, 41.85, 37.42, 35.79, 35.36, 35.29, 34.86, 30.89, 30.79, 30.66, 29.00, 27.69, 26.70, 23.27, 23.09, 21.55, 17.32, 12.77. HRMS (ESI) calcd for $C_{34}H_{51}NO_7S$ [M+H]$^+$ = 618.3459; Found 618.3486.

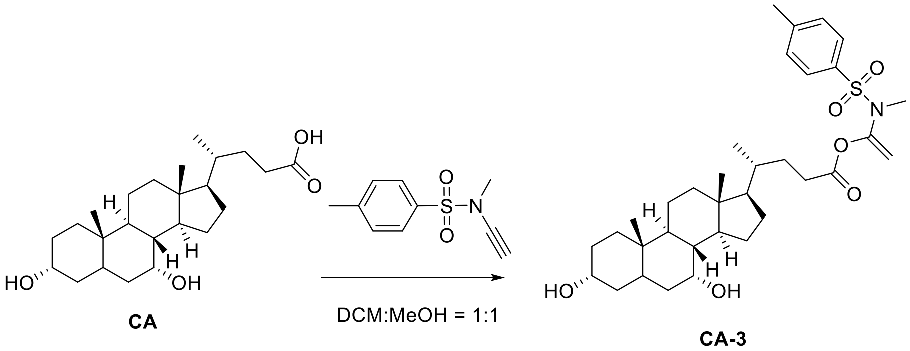

Scheme S34

(**CA-3**). This compound was synthesized according to General Procedure C, product **CA-3** was afforded as a white solid (599 mg, 85% yield). ^{1}H NMR (400 MHz, DMSO-d_6) δ 7.68 (dd, *J* = 8.2, 2.3 Hz, 2H), 7.55 – 7.34 (m, 2H), 4.74 (dt, *J* = 27.1, 2.8 Hz, 2H), 4.32 (s, 1H), 4.11 (t, *J* = 2.8 Hz, 1H), 3.63 (s, 1H), 3.18 (d, *J* = 11.4 Hz, 1H), 2.94 (d, *J* = 2.3 Hz, 1H), 2.41 (d, *J* = 2.2 Hz, 3H), 2.33 – 2.09 (m, 3H), 1.95 – 1.62 (m, 8H), 1.52 – 0.97 (m, 17H), 0.84 (t, *J* = 5.3 Hz, 6H), 0.60 (d, *J* = 2.4 Hz, 3H).^{13}C NMR (151 MHz, DMSO-d_6) δ 171.37, 146.71, 144.53, 13.28, 130.23, 127.98, 100.15, 70.82, 66.64, 55.86, 50.46, 42.41, 41.91, 37.41, 35.79, 35.30, 35.20, 35.18, 32.76, 31.03, 30.71, 30.57, 28.18, 23.61, 23.17, 21.55, 20.73, 18.52, 12.09. HRMS (ESI) calcd for $C_{34}H_{51}NO_6S$ [M+Na]$^+$ = 624.3329; Found 624.3339.

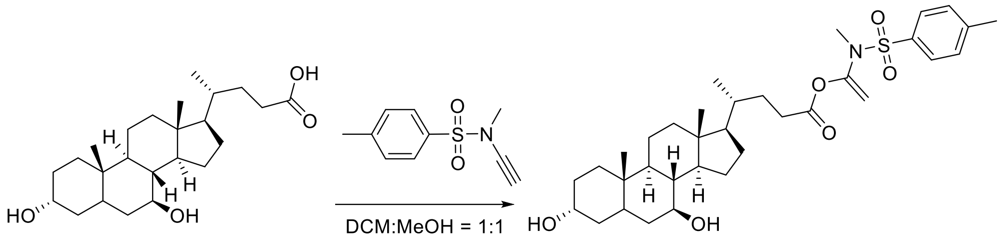

UA UA-3

Scheme S35

(**UA-3**). This compound was synthesized according to General Procedure C, product **UA-3** was afforded as a white solid (599 mg, 85% yield). ^{1}H NMR (400 MHz, DMSO-d_6) δ 7.68 (d, J = 7.9 Hz, 2H), 7.45 (d, J = 7.9 Hz, 2H), 4.88 – 4.76 (m, 1H), 4.74 – 4.68 (m, 1H), 4.44 (t, J = 3.5 Hz, 1H), 3.87 (d, J = 6.9 Hz, 1H), 3.29 – 3.22 (m, 2H), 2.94 (s, 3H), 2.41 (s, 3H), 2.29 – 2.13 (m, 2H), 1.98 – 1.57 (m, 6H), 1.54 – 1.05 (m, 18H), 0.87 (d, J = 5.9 Hz, 6H), 0.61 (s, 3H).^{13}C NMR (151 MHz, DMSO-d_6) δ 171.40, 146.69, 144.53, 134.30, 130.24, 127.98, 100.20, 70.20, 69.93, 56.30, 54.99, 43.57, 43.50, 42.66, 38.20, 37.75, 37.42, 35.30, 35.09, 34.23, 30.72, 30.61, 28.56, 27.17, 23.78, 21.55, 21.31, 18.67, 12.49. HRMS (ESI) calcd for $C_{34}H_{51}NO_6S$ $[M+Na]^+$ = 624.3329; Found 624.3325.

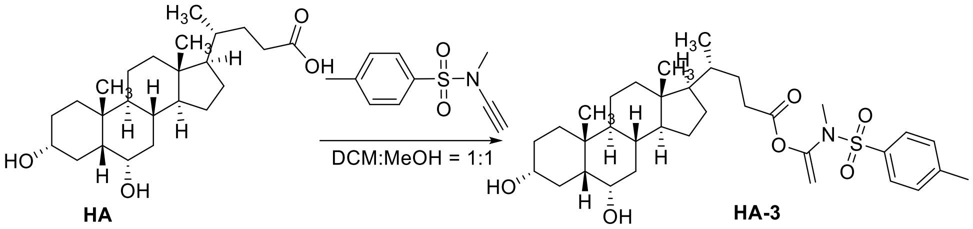

Scheme S36

(**HA-3**). This compound was synthesized according to General Procedure C, product **HA-3** was afforded as a white solid (599 mg, 85% yield). ^{1}H NMR (400 MHz, DMSO-d_6) δ 7.68 (d, J = 8.0 Hz, 2H), 7.45 (d, J = 7.9 Hz, 2H), 4.78 (d, J = 2.0 Hz, 1H), 4.71 (d, J = 1.9 Hz, 1H), 4.42 (s, 1H), 4.24 (s, 1H), 3.90 – 3.73 (m, 1H), 3.26 (s, 1H), 2.41 (s, 3H), 2.35 – 2.26 (m, 3H), 2.18 (dd, J = 15.7, 7.3 Hz, 2H), 1.95 – 1.42 (m, 6H), 1.41 – 0.92 (m, 18H), 0.84 (s, 6H), 0.60 (s, 3H). ^{13}C NMR (151 MHz, DMSO-d_6) δ 171.36, 146.72, 144.50, 134.28, 130.23, 127.99, 100.16, 70.49, 66.37, 56.33, 55.81, 48.74, 42.84, 37.42, 36.02, 35.87, 35.35, 35.06, 34.82, 30.84, 30.74 – 30.62 (m), 30.52, 29.73, 28.08, 24.34, 24.02, 21.54, 20.88, 18.48, 12.31. HRMS (ESI) calcd for $C_{34}H_{51}NO_6S$ $[M+Na]^+$ = 624.3329; Found 624.333.

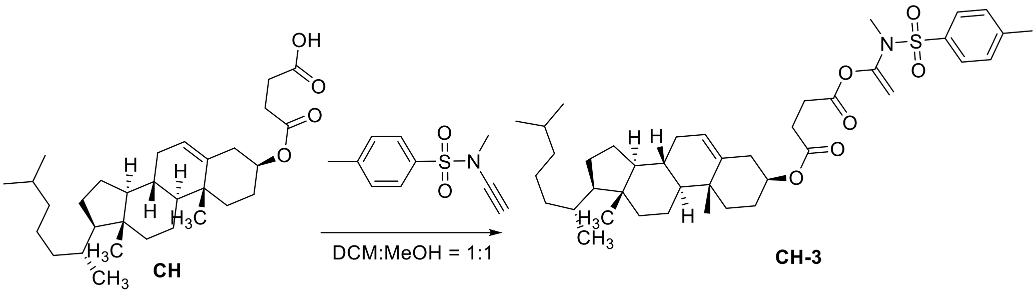

Scheme S37

(**CH-3**). This compound was synthesized according to General Procedure C, product **CH-3** was afforded as a white solid (519 mg, 65% yield). ^{1}H NMR (400 MHz, $CDCl_3$) δ 7.74 (d, *J* = 8.0 Hz, 2H), 7.34 (d, *J* = 8.1 Hz, 2H), 5.44 – 5.36 (m, 1H), 4.84 (d, *J* = 2.6 Hz, 1H), 4.65 (d, *J* = 2.6 Hz, 2H), 3.02 (s, 3H), 2.66 (dd, *J* = 7.5, 4.8 Hz, 2H), 2.59 (dd, *J* = 7.5, 4.8 Hz, 2H), 2.46 (d, *J* = 4.2 Hz, 3H), 2.33 (d, *J* = 8.2 Hz, 2H), 2.08 – 1.95 (m, 2H), 1.86 (m, 3H), 1.69 – 1.44 (m, 7H), 1.42 – 1.24 (m, 6H), 1.15 (dd, *J* = 9.3, 4.6 Hz, 6H), 1.03 (s, 3H), 1.01 – 0.96 (m, 2H), 0.93 (d, *J* = 6.4 Hz, 3H), 0.88 (dd, *J* = 6.7, 1.8 Hz, 6H), 0.69 (s, 3H).^{13}C NMR (151 MHz, $CDCl_3$) δ 171.31, 170.05, 146.81, 144.18, 139.52, 133.62, 129.57, 128.04, 122.78, 100.51, 74.53, 56.66, 56.08, 49.97, 42.30, 39.70, 39.52, 38.04, 37.25, 36.94, 36.57, 36.18, 35.82, 31.91, 31.82, 29.09, 29.03, 28.27, 28.05, 27.71, 24.30, 23.83, 22.89, 22.61, 21.67, 21.02, 19.35, 18.73, 11.88. HRMS (ESI) calcd for $C_{41}H_{61}NO_6S$ $[M+Na]^+$ = 718.4112; Found 718.411.

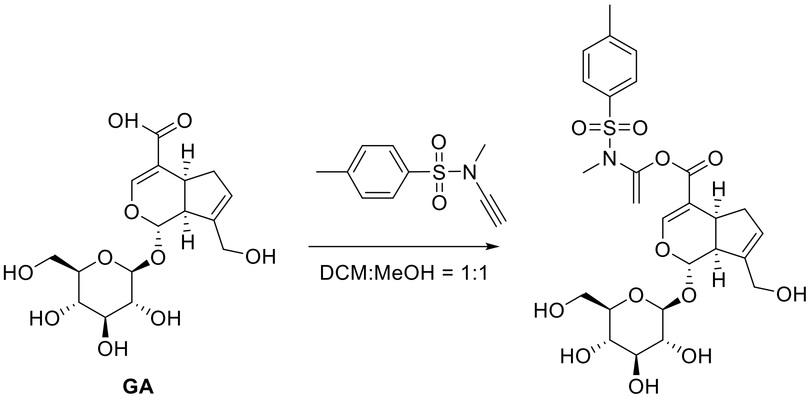

Scheme S38

(**GA-3**). This compound was synthesized according to General Procedure C, product **GA-3** was afforded as a white solid (515 mg, 75% yield). ^{1}H NMR (400 MHz, DMSO-d_6) δ 7.74 – 7.61 (m, 2H), 7.44 (d, *J* = 8.0 Hz, 2H), 5.76 (d, *J* = 2.4 Hz, 1H), 5.67 (s, 1H), 5.11 – 5.04 (m, 2H), 5.02 (d, *J* = 4.1 Hz, 2H), 4.99 – 4.95 (m, 1H), 4.87 (d, *J* = 3.3 Hz, 1H), 4.80 (s, 1H), 4.75 (d, *J* = 6.5 Hz, 1H), 4.58 – 4.52 (m, 1H), 4.47 (d, *J* = 6.5 Hz, 1H), 4.14 (d, *J* = 15.3 Hz, 1H), 3.98 (d, *J* = 15.1 Hz, 1H), 3.72 – 3.57 (m, 1H), 3.47 – 3.37 (m, 1H), 3.16 (t, *J* = 13.6 Hz, 2H), 3.04 (dt, *J* = 23.1, 7.2 Hz, 2H), 2.94 (d, *J* = 2.6 Hz, 3H), 2.90 (t, *J* = 8.2 Hz, 1H), 2.60 (d, *J* = 7.5 Hz, 1H), 2.40 (d, *J* = 2.5 Hz, 3H), 1.93 (d, *J* = 16.8 Hz, 1H). ^{13}C NMR (151 MHz, DMSO-d_6) δ 164.33, 154.41, 146.34, 144.65, 144.44, 134.04, 130.29, 128.04, 125.94, 109.96, 99.06, 96.69, 77.81, 77.04, 73.73, 70.33, 61.37,

59.83, 55.42, 46.01, 38.12, 37.27, 34.90, 21.55. HRMS (ESI) calcd for $C_{26}H_{33}NO_{12}S$ $[M+H]^+$ = 584.1796; Found 584.1799.

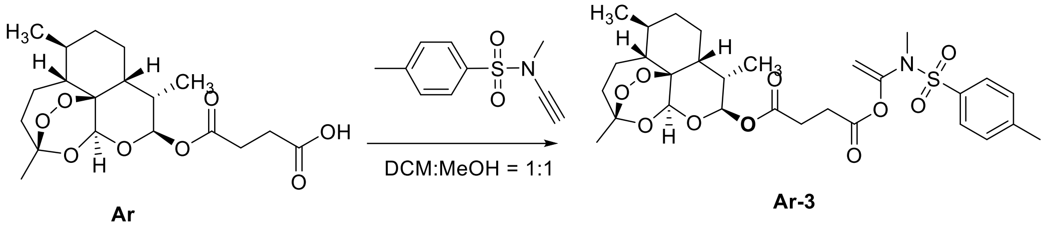

Scheme S39

(**Ar-3**). This compound was synthesized according to General Procedure C, product **Ar-3** was afforded as a white solid (592 mg, 85% yield). ^{1}H NMR (400 MHz, DMSO-d_6) δ 7.69 (d, *J* = 7.9 Hz, 2H), 7.47 (d, *J* = 7.9 Hz, 2H), 5.67 (d, *J* = 9.6 Hz, 1H), 5.57 (d, *J* = 2.7 Hz, 1H), 4.80 (s, 1H), 4.73 (t, *J* = 2.9 Hz, 1H), 2.93 (d, *J* = 2.6 Hz, 3H), 2.60 (s, 3H), 2.42 (d, *J* = 2.6 Hz, 3H), 2.31 (ddd, *J* = 10.9, 8.6, 5.3 Hz, 1H), 2.18 (dd, *J* = 19.5, 8.6 Hz, 1H), 1.99 (dd, *J* = 10.0, 7.4 Hz, 1H), 1.86 – 1.75 (m, 1H), 1.66 – 1.37 (m, 6H), 1.36 – 1.16 (m, 5H), 0.95 (d, *J* = 12.5 Hz, 1H), 0.89 (d, *J* = 6.2 Hz, 3H), 0.76 (d, *J* = 7.1 Hz, 3H). ^{13}C NMR (151 MHz, DMSO-d_6) δ 170.99, 170.03, 146.43, 144.67, 134.05, 130.30, 128.04, 104.06, 100.45, 92.41, 91.09, 80.32, 55.38, 51.58, 45.03, 37.20, 36.42, 36.36, 34.17, 32.08, 28.81, 28.77, 25.96, 24.66, 21.54, 21.46, 20.52, 12.20. HRMS (ESI) calcd for $C_{29}H_{39}NO_{10}S$ $[M+Na]^+$ = 616.2187; Found 616.2178.

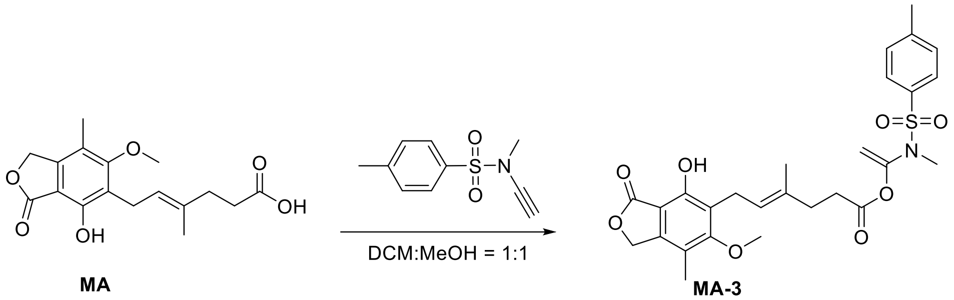

Scheme S40

(**MA-3**). This compound was synthesized according to General Procedure C, product **MA-3** was afforded as a white solid (538 mg, 85% yield). ^{1}H NMR (400 MHz, $CDCl_3$) δ 7.73 – 7.67 (m, 2H), 7.32 (d, *J* = 8.1 Hz, 2H), 5.31 (s, 1H), 5.20 (s, 2H), 4.74 (d, *J* = 2.5 Hz, 1H), 4.60 (d, *J* = 2.5 Hz, 3H), 3.77 (s, 2H), 3.39 (d, *J* = 7.0 Hz, 3H), 2.97 (s, 5H), 2.42 (d, *J* = 4.3 Hz, 2H), 2.26 (d, *J* = 7.9 Hz, 3H), 2.16 (s, 1H), 2.05 (s, 1H), 1.79 (d, *J* = 1.4 Hz, 3H). ^{13}C NMR (151 MHz, $CDCl_3$) δ 172.93, 170.71, 163.67, 153.57, 146.92, 144.10 (d, *J* = 2.5 Hz), 133.87, 133.64, 129.51, 122.99, 121.97, 116.81, 100.41, 70.08, 61.01, 37.17, 34.03, 32.66, 22.58, 21.55, 16.13, 11.58. HRMS (ESI) calcd for $C_{27}H_{31}NO_8S$ $[M+Na]^+$ = 552.1663; Found 552.1666.

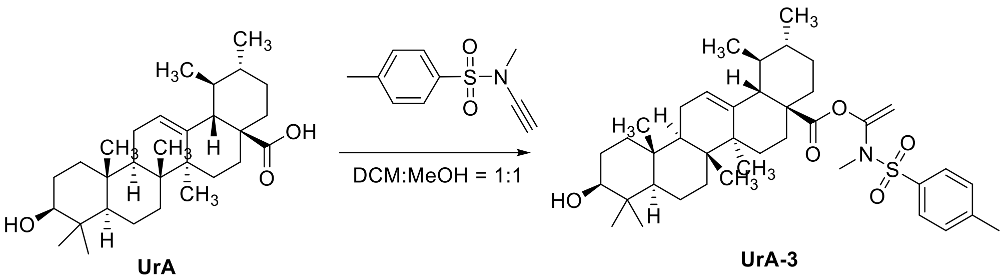

Scheme S41

(**UrA-3**). This compound was synthesized according to General Procedure C, product **UrA-3** was afforded as a white solid (500 mg, 65% yield). ^{1}H NMR (400 MHz, $CDCl_3$) δ 7.78 – 7.71 (m, 2H), 7.33 (t, J = 6.4 Hz, 2H), 5.21 (t, J = 3.7 Hz, 1H), 4.81 (d, J = 2.2 Hz, 1H), 4.75 (d, J = 2.2 Hz, 1H), 3.21 (dd, J = 10.9, 5.0 Hz, 1H), 2.94 (s, 3H), 2.43 (s, 3H), 1.94 – 1.85 (m, 3H), 1.63 – 1.52 (m, 9H), 1.49 – 1.38 (m, 4H), 1.33 (s, 2H), 1.30 – 1.27 (m, 2H), 1.26 (d, J = 1.6 Hz, 4H), 1.07 (s, 3H), 0.99 (s, 3H), 0.94 – 0.91 (m, 6H), 0.83 (t, J = 4.1 Hz, 3H), 0.78 (d, J = 2.0 Hz, 6H).^{13}C NMR (151 MHz, $CDCl_3$) δ 175.12, 146.88, 143.91, 137.61, 134.05, 130.01, 129.53, 128.17, 127.34, 126.08, 102.92, 79.04, 55.23, 52.72, 48.63, 47.53, 42.17, 39.65, 39.06, 38.76, 38.70, 38.66, 36.98, 36.82, 36.04, 33.15, 30.52, 28.16, 27.98, 27.23, 24.04, 23.39, 23.30, 21.60, 21.13, 18.31, 17.45, 16.91, 15.64 (d, J = 2.2 Hz), 15.51. HRMS (ESI) calcd for $C_{40}H_{59}NO_5S$ $[M+Na]^+$ = 688.4006; Found 688.4014.

2. Identification of conjugation product between A2 and Ethylamine

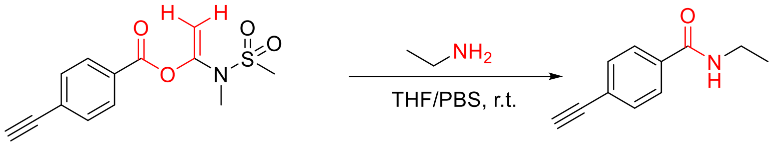

Scheme S42

A2 (279.3 mg, 1 mmol), ethylamine (45.0 mg, 1 mmol) and THF/PBS (1:1) 2 mL were added to a round bottom flask. After stirring at r.t. overnight. The mixture was extracted with ethyl acetate (3×5 mL). The organic layers were combined and dried with Na_2SO_4. Upon evaporation of the solvent under reduced pressure, the crude product was purified by flash chromatography eluting with ethyl acetate/petroleum ether (1:20) to produce **S19** as a white solid (144 mg, 83%).^{1}H NMR (400 MHz, DMSO-d_6) δ 8.54 (s, 1H), 7.84 (d, J = 8.0 Hz, 2H), 7.56 (d, J = 8.0 Hz, 2H), 4.36 (s, 1H), 3.31 – 3.24 (m, 2H), 1.12 (t, J = 7.2 Hz, 3H).

3. Stability analysis of A1

Briefly, HPLC analysis of the stability of **A1** under different conditions. **A1** in high probe concentration (8 mM) or low probe concentration (100 μM) was dissolved in PBS or RPMI 1640 medium with FBS (1 mL) and incubated at 37°C for 24 h. 50 μL of the mixture was added to MeOH (1 mL) and remove insoluble impurities with filter membrane. Finally, the mixture was analyzed by HPLC.

4. The reactivity analysis of A1/A2/A3/A4 with amino compounds

A1-**A4** (0.1 mmol), benzylamine (0.1 mmol) and THF/PBS (1:1) 1 mL were added to a round bottom flask. After stirring at r.t. for 5 h, the reactions were analyzed by HPLC (Aglient Technologies, 1260 Infinity, YMC-Triant C18, 250 × 4.6 mml.D. S-5 μm, 12 nm). Flow phase was 85% MeOH and 15% H_2O with flow rate of 1.0 mL/min.

5. HPLC analysis of the reactivity between A1 and different amino acids

To a solution of **A1** (35.5 mg, 0.1mmol) in THF (1.0 mL)/PBS (1.0 mL) was added methyl(tert-butoxycarbonyl)-L-lysinate (0.1mmol), methyl(tert-butoxycarbonyl)-L-cysteinate (0.1 mmol), 4-((tert-butoxycarbonyl)amino)-5-methoxy-5-oxopentanoic acid (0.1 mmol), methyl(tert-butoxycarbonyl)-L-tyrosinate (0.1 mmol), methyl(tert-butoxycarbonyl)-L-tryptophan (0.1 mmol), methyl(tert-butoxycarbonyl)-L-histidinate (0.1 mmol), methyl(tert-butoxycarbonyl)-L-threonine (0.1 mmol), methylacetyl-L-serinate (0.1 mmol) respectively. After incubation for 24 h, 10 μL of the mixture was subsequently analyzed by HPLC (Aglient Technologies, 1260 Infinity, YMC-Triant C18, 250 × 4.6 mml.D. S-5 μm, 12 nm). Flow phase was 80% MeOH and 20% H_2O with flow rate of 1.0 mL/min.

6. Database search

The raw data were processed by using MaxQuant software (1.5.8.3) and processed as per default workflow. MS tolerance is 4.5 ppm, and MS/MS tolerance is 20 ppm. Searches were performed against the UniProtKB human database (taxonomy 9606, version 20180929). Reversed database searches were used to evaluate false discovery rate (FDR) of site, peptide and protein identifications. Two missed cleavage sites of trypsin were allowed. Carbamidomethylation (C) was set as a fixed modification, and acetyl (Protein N-term), oxidation (M), deamidation (NQ), modified probes were set as variable modifications. The FDR of both peptide identification and protein identification is set to be 1%. The options of "Second peptides", "Match between runs" and "Dependent peptides" were used. Label-free quantification was used to quantify the difference between different samples.

As such there is no fixed cut-off score threshold but instead spectra were accepted until the 1% false discovery rate (FDR) detected. Only peptides with a minimum length of 7 amino acids were considered for identification and detected in at least one or more of the replicates. All probes modified peptide spectra were manually validated by applying stringent acceptance criteria: only modification event on K with PEP ≤ 0.01 were used for further analysis. Assignments were screened for peptides uniquely labeled on a single amino acid residue in two out of three biological replicates.

7. Annexin V-FITC/PI dual staining analysis

MDA-MB-231 cells were seeded in a six-well plate and treated with indicated concentrations of probe **A1/A2/A3/A4** for 4 h. Then the cells were harvested and washed with cold PBS and resuspended in 100 μL 1×BD binding buffer (BD PharmingenTM, #559763) with annexin V-PE and 7-AAD for 10 min in dark. 400 μL 1×BD binding buffer was added to stop dyeing before measurement on a Guava easyCyte flow cytometer (Merck, USA).

8. Cellular thermal shift assay (CETSA)

K562 or H3255 cells were grown to 80-90% confluence in 10 cm dishes under conditions described above. The medium was removed and cells were washed twice with PBS and then treated with 10 mL probe-containing FBS-free medium (final concentration of the probe was 10 µM). Control cells were incubated with an equal volume of DMSO. After 2 h of incubation, the medium was aspirated and cells were washed twice with PBS to remove excessive probes, harvested with trypsin and centrifuged at 800 rpm for 3 min at room temperature. The pellets were suspended in PBS and the cells were suspended equally (100 µL) into PCR tubes. The samples were then subjected for 3 minutes to a 8-step temperature gradient (40-61°C) using BIO-RAD S1000™ Thermal Cycler and lysed by cycling freezing in liquid nitrogen and melting at 37°C three times. The cell lysates were centrifuged at 14000 rpm for 30 min to remove aggregates at 4°C, supernatants were transferred to 1.5 mL tubes and dissolved in 5 × SDS loading buffer and heated for 10 min at 95°C. The CDK1 or EGFR L858R was identified by western blot. Immunoblotting band intensities were quantified using ImageJ software and thermal curves were analyzed by Boltzmann curve fitting using Graph Pad Prism software.

9. Pull-down experiment to validate the targets of natural ligands

MDA-MB-231 cells were lysed in pre-chilled NETN buffer containing 50 mM HEPES (pH 7.6), 150 mM NaCl, and 1% IGEPAL and was added 1×protease and phosphatase inhibitors (Thermo Scientific, A32961). 1.5 mg protein was then incubated with natural products and probes at different concentrations or DMSO for 2 hours at room temperature under rotation. After this, the mixture was incubated with NHS-biotin (Sigma, CAS No. 459426-22-3) at 100 µM probe concentration for 1 hour at room temperature. After that, 200 µL of streptavidin agarose beads (GE, Cat. No. 17-5113-01) were added and incubated at room temperature for 2 hours. The beads were initially washed three times with NETN buffer and then washed three times with PBS. Finally, each sample was supplemented with 100 µL of 1× loading buffer and boiled at 95°C for 20 minutes. The enriched bands were detected using the corresponding OAT antibodies (SAB, Cat. No. #38766) through Western blotting (WB).

Supplementary Figures

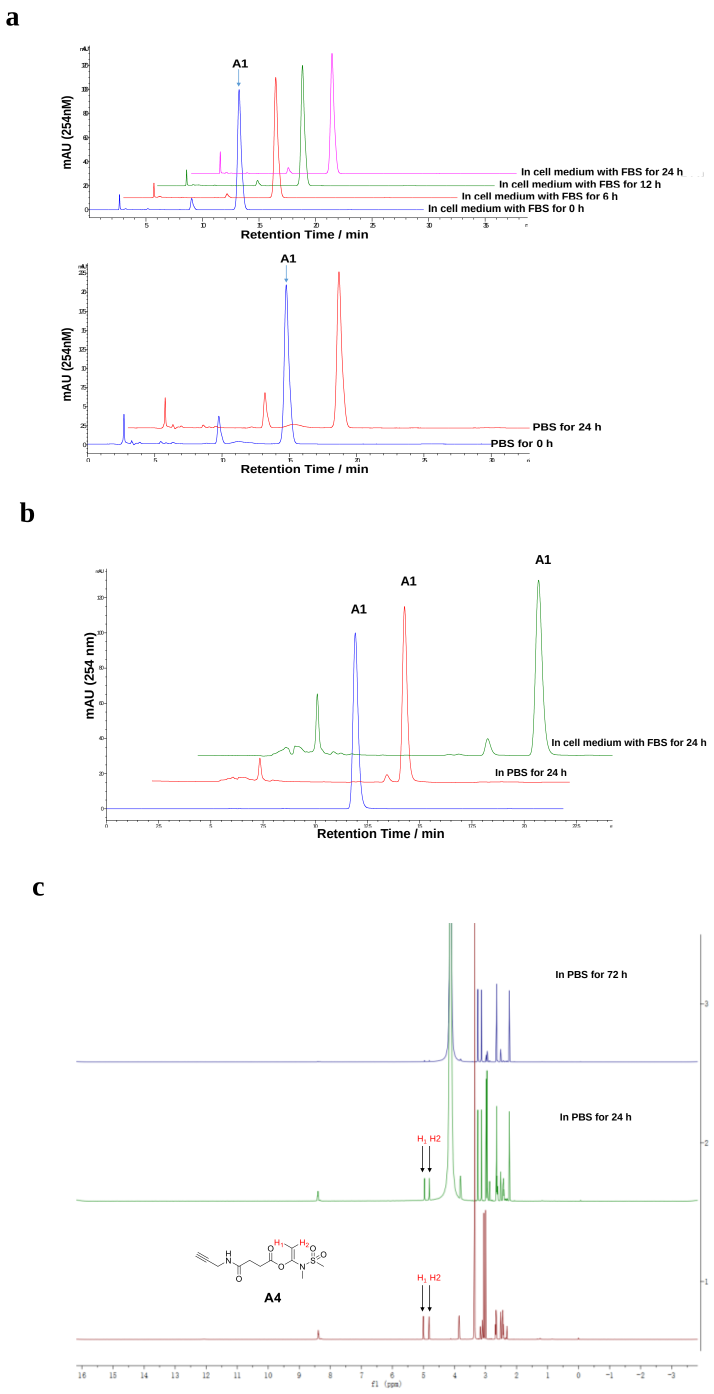

Supplementary Fig. 1 The stability of the probes. a A1 (8 mM) in PBS or cell medium with FBS (1640). **b** A1 (100 μM) in PBS or cell medium with FBS (1640). **c** ^{1}H NMR spectra analysis of A4 in DMSO-d6: PBS = 1:1 under different time conditions.

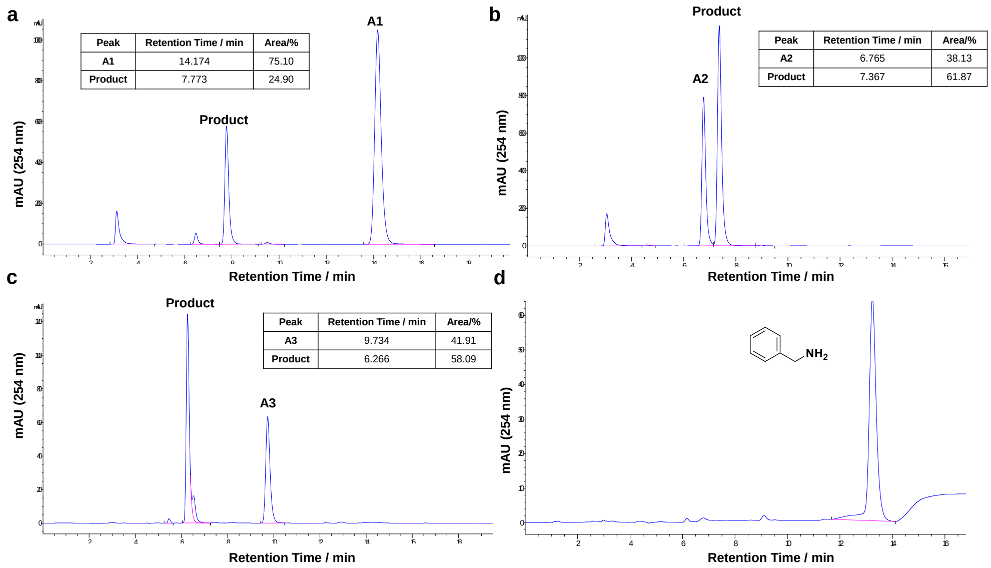

Peak	Retention Time / min	Area/%
A1	14.174	75.10
Product	7.773	24.90

Peak	Retention Time / min	Area/%
A2	6.765	38.13
Product	7.367	61.87

Peak	Retention Time / min	Area/%
A3	9.734	41.91
Product	6.266	58.09

Supplementary Fig. 2 The reactivity analysis of A1/A2/A3/A4 with amino compounds. a HPLC analysis of the reaction between A1 and benzylamine (0.1 mmol) in THF:PBS = 1:1 at 37℃ for 5 h, and the product yield is 24.9%. **b** HPLC analysis of the reaction between A2 and benzylamine (0.1 mmol) in THF:PBS = 1:1 at 37℃ for 5 h, and the product yield is 61.87%. **c** HPLC analysis of the reaction between A3 and benzylamine (0.1 mmol) in THF:PBS = 1:1 at 37℃ for 5 h, and the product yield is 58.09%. **d** HPLC analysis of the reaction between A4 and benzylamine (0.1 mmol) in THF:PBS = 1:1 at 37℃ for 5 h, and no products were detected.

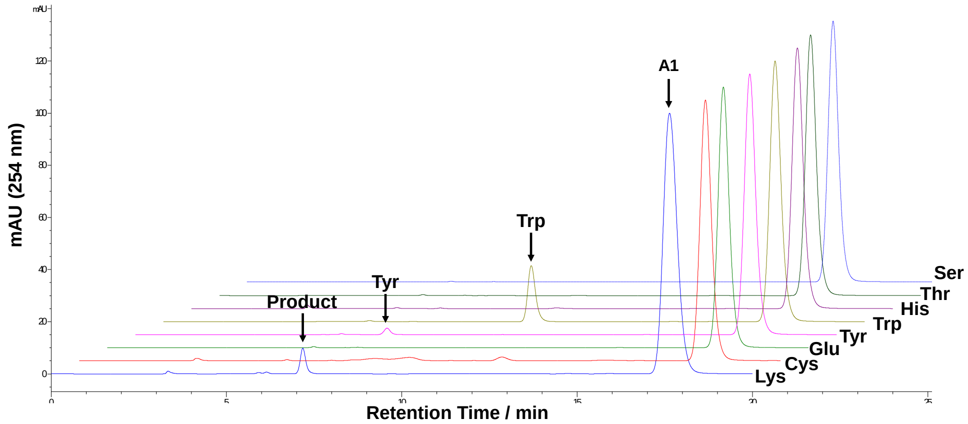

Supplementary Fig. 3. HPLC analysis of the reaction between A1 and different amino acids, no product was observed from the reaction with Ser, Thr, His, Trp, Tyr, Glu and Cys, and a portion of product was observed from the reaction with Lys.

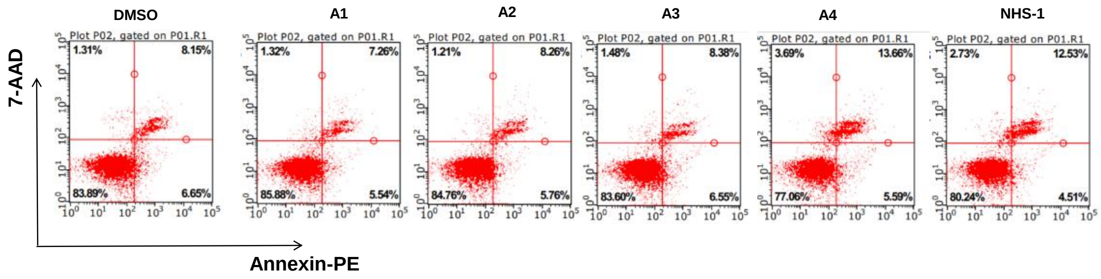

Supplementary Fig. 4 Apoptosis of MDA-MB-231 cells treated with A1/A2/A3/A4/NHS-1 (100 μM) for 4 h, which was analyzed by flow cytometry followed by cell death detection.

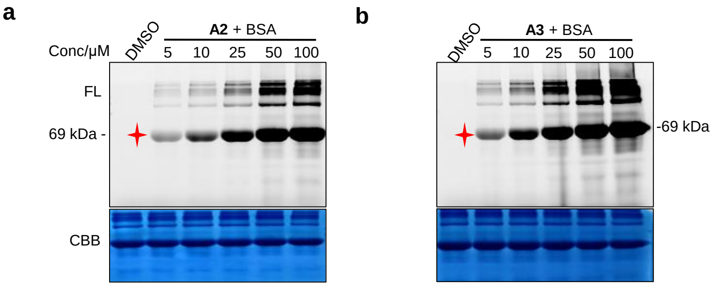

Supplementary Fig. 5 Concentration-dependent labeling profiles of BSA with A2/A3 (4 h incubation).

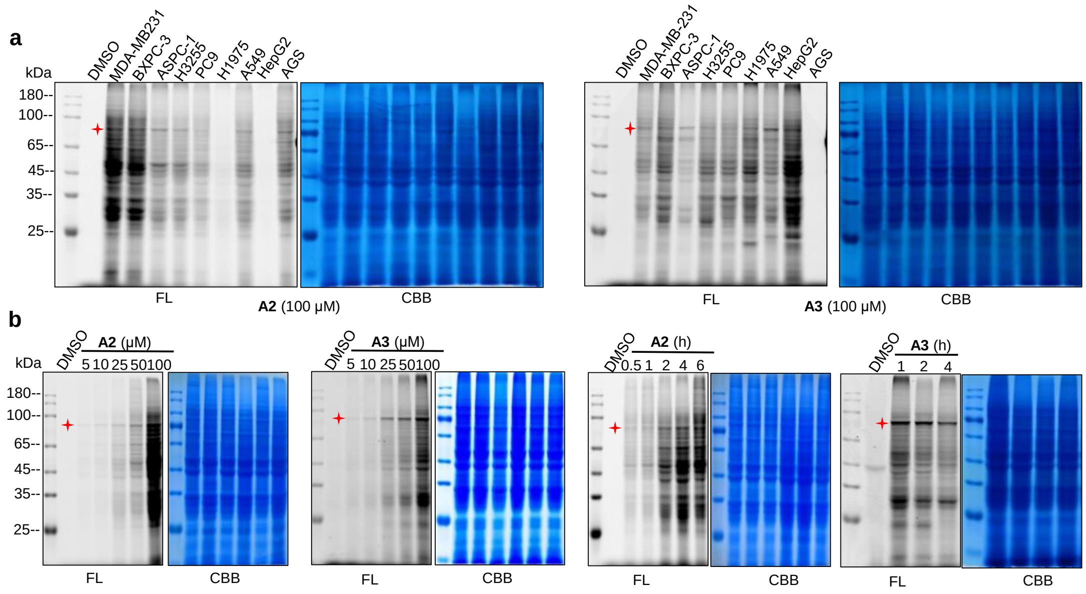

Supplementary Fig. 6 In situ proteome labeling. **a** Proteome reactivity profiles of different cancer cells with A2 and A3 (100 μM), FL = in-gel fluorescence scanning. CBB = Coomassie gel. **b** Concentration- and time-dependent labeling profiles of MDA-MB-231 live cells with A2/A3.

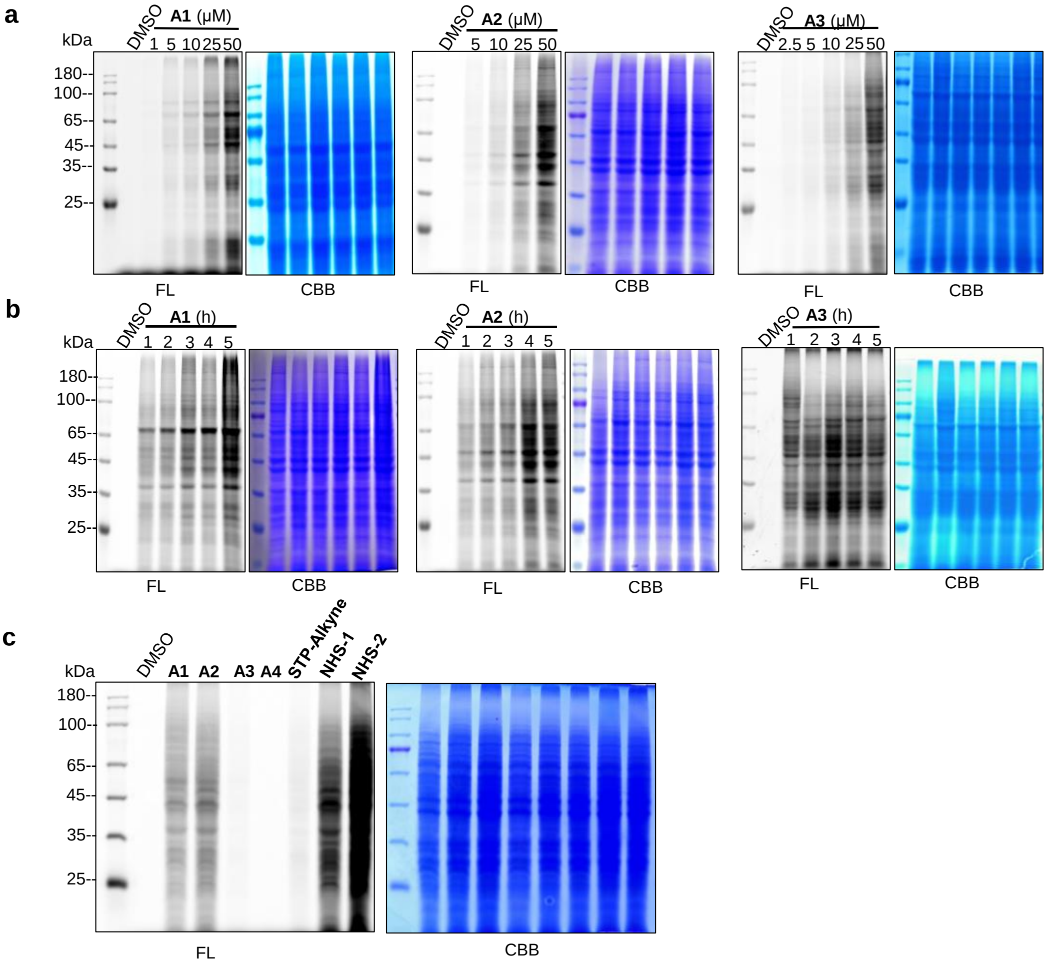

Supplementary Fig. 7 Concentration- and Time-dependent labeling of A1/A2/A3/A4. a Concentration-dependent (4 h incubation time) labeling profiles of MDA-MB-231 cell lysates with A1/A2/A3. **b** Time-dependent labeling profiles of MDA-MB-231 cell lysates with A1/A2/A3 (final probe concentration is 50 μM). **c** Proteome reactivity profiles of A1/2/3/4/STP-alkyne/NHS-1/NHS-2 (100 μM) with MDA-MB-231 cell lysates, FL = in-gel fluorescence scanning. CBB = Coomassie gel.

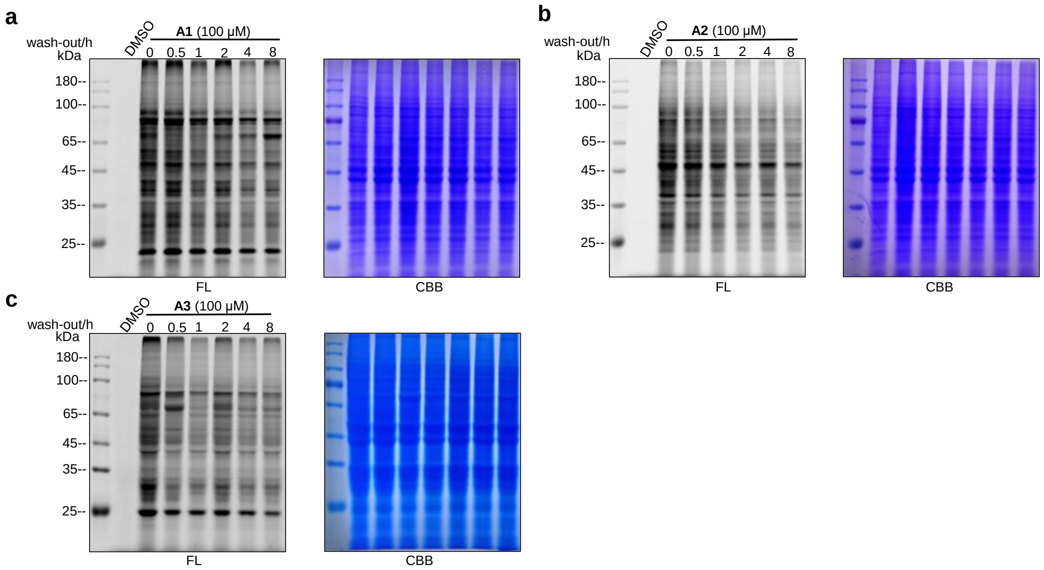

Supplementary Fig. 8 MDA-MB-231 cells were treated with A1/A2/A3, then washed with PBS for three times, followed by wash-out for the indicated times with fresh culture medium. Labeling profile experiments were carried out.

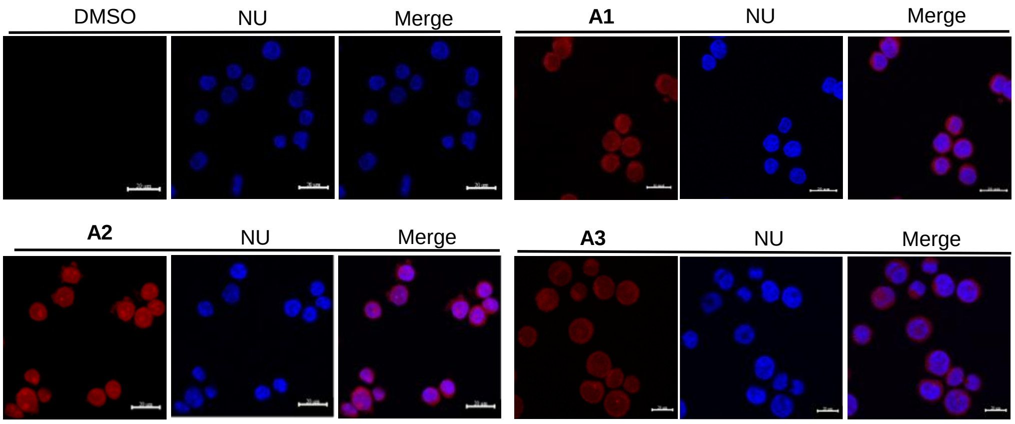

Supplementary Fig. 9 Cellular imaging of A1/A2/A3 (50 μM) in MDA-MB-231 cells, Nu = nucleus. Scale bar = 20 μm.

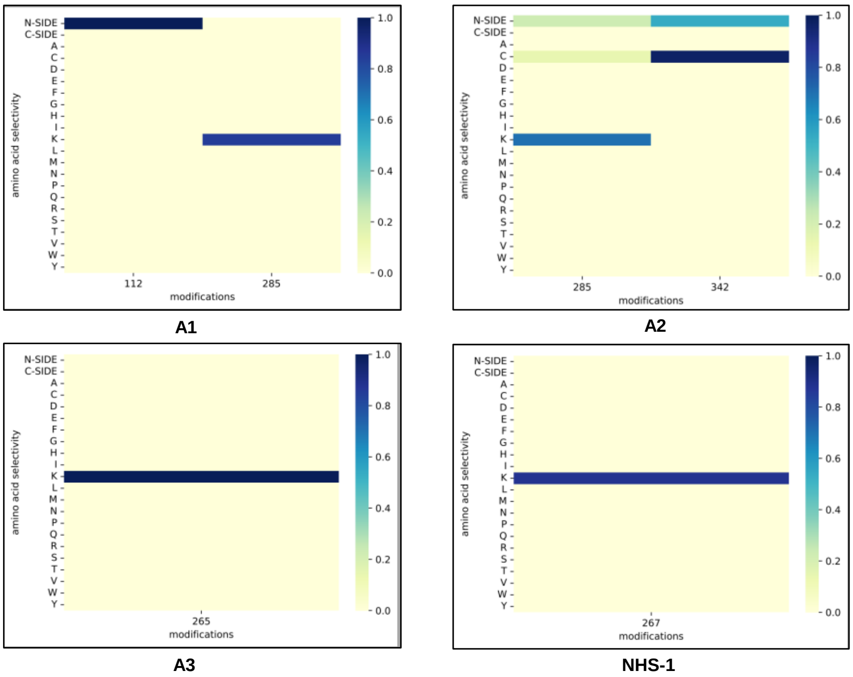

Supplementary Fig. 10 Amino acid selectivity assessment of A1/A2/A3/NHS-1 in MDA-MB-231 cell lysate by using quantitative proteomics approach (pChem search engine).

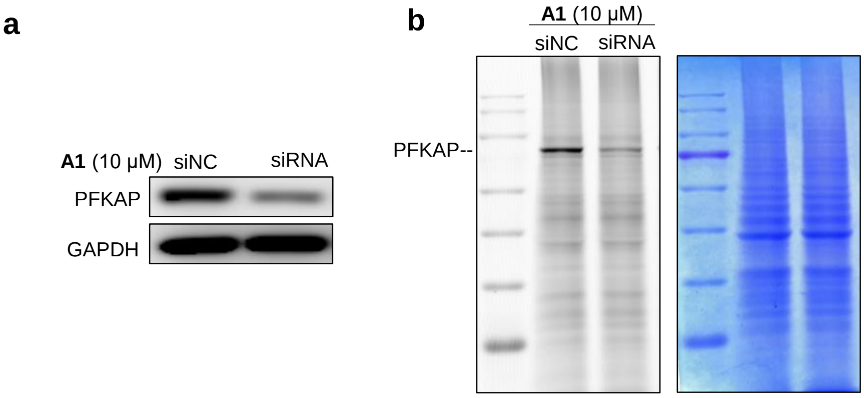

Supplementary Fig. 11 Validation of labeling profile of PFKAP with A1. a Western blotting and **b** Labeling of target protein PFKAP by probe A1 in the presence of siRNA or siNC.

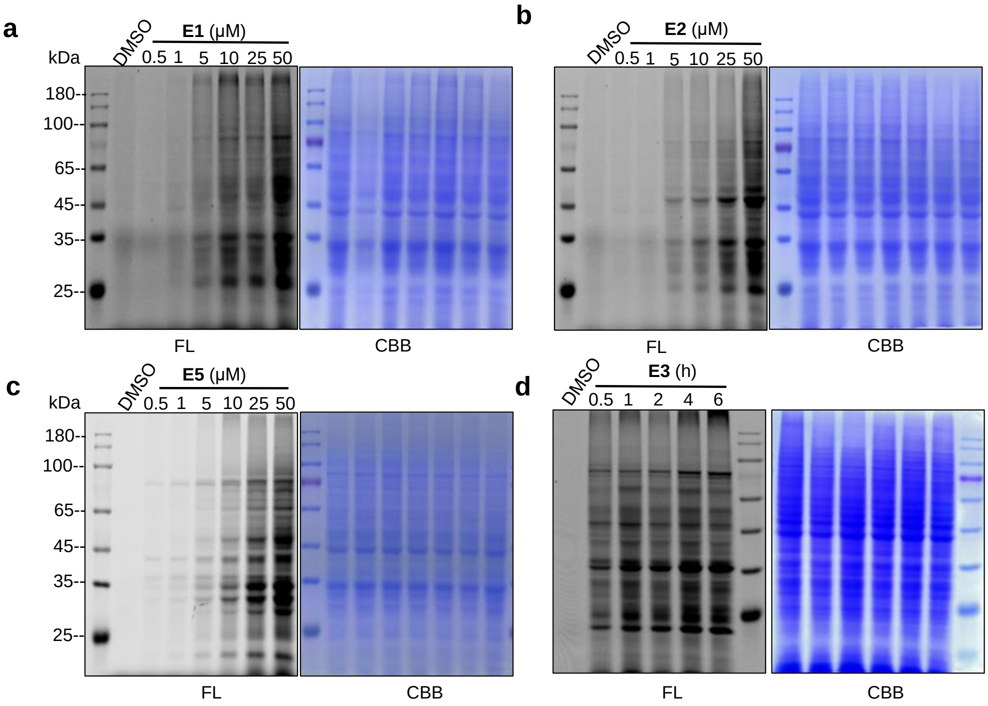

Supplementary Fig. 12 Concentration- and Time-dependent labeling of E1/E2/E3/E5. a b c Concentration-dependent labeling profiles of H3255 live cells with E1/E2/E5. **d** Time-dependent labeling profiles of H3255 live cells with E3.

Time	IC_{50} (nM) Against EGFR L858R
0 h	17.25
10 min	18.74
0.5 h	8.951
1 h	6.020
3 h	1.846
6 h	1.611
12 h	1.314

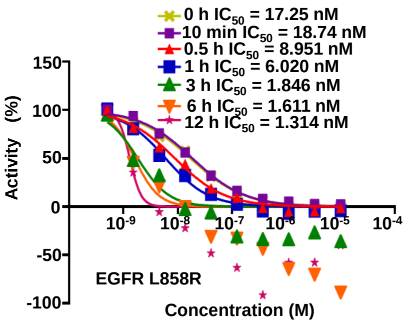

Supplementary Fig. 13 Time-dependent inhibitory potency of E3 against EGFR L858R after 12 h.

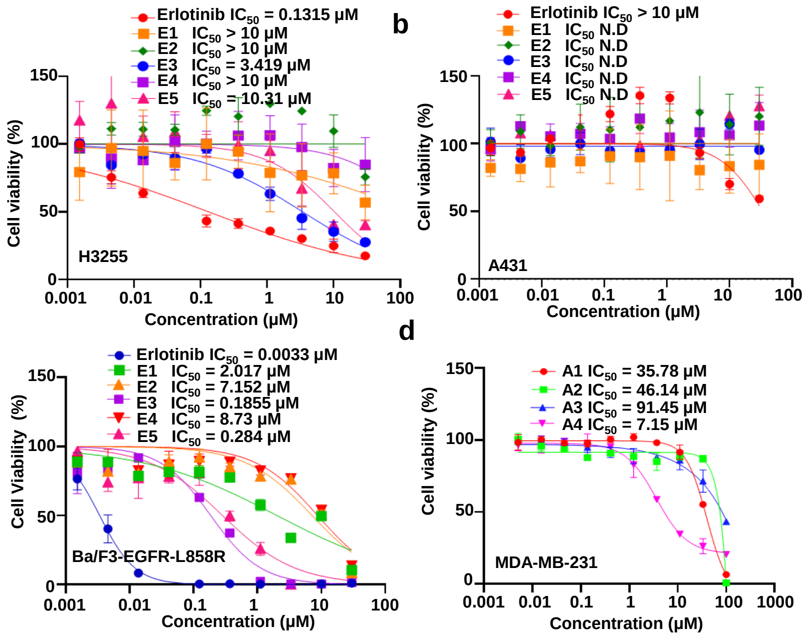

Supplementary Fig. 14 Cellular inhibition assay of E1/2/3/4/5 against different cancer cells. a H3255 cells. **b** A431 cells. **c** BaF3 cells. **d** Cellular inhibition assay of A1/2/3/4 against MDA-MB-231 cancer cells.

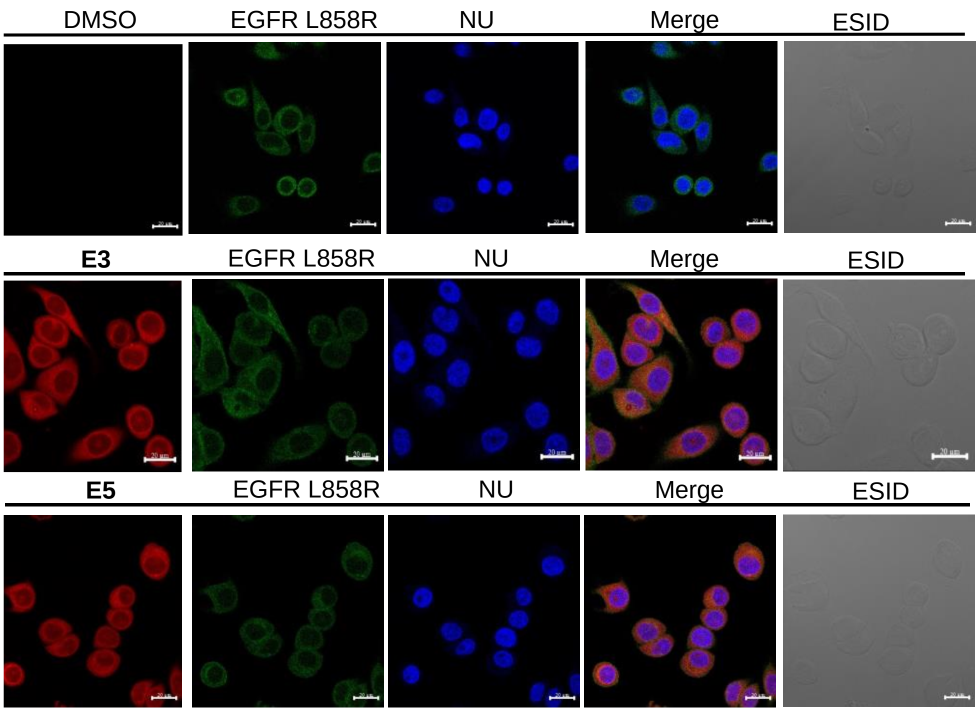

Supplementary Fig. 15 Cellular imaging of E3/E5 (10 μM) in H3255 cells harboring EGFR L858R and immunofluorescence (IF) against EGFR. Scale bar = 20 μm.

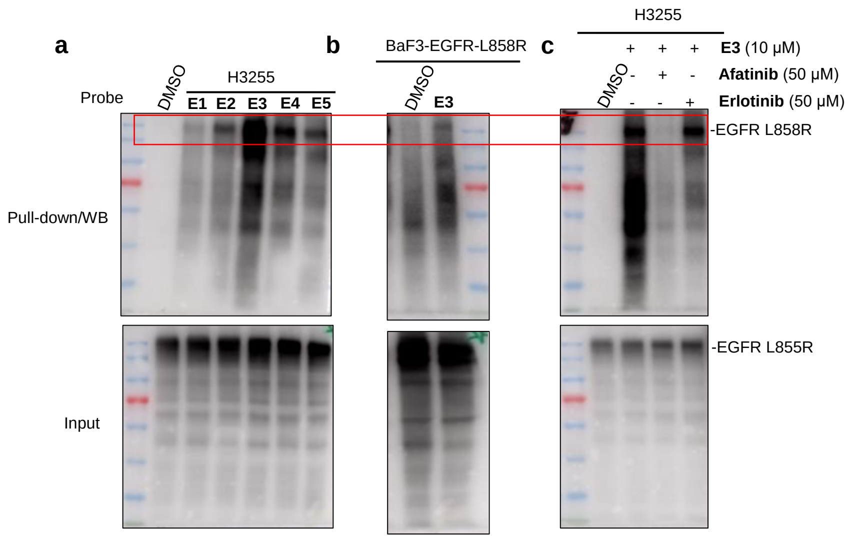

Supplementary Fig. 16 Pull-down and western blot experiments against EGFR L858R. a Pull-down/WB validation of EGFR L858R in H3255 cells with E1, E2, E3, E4 and E5 (10 μM); **b** Pull-down/WB validation of EGFR L858R in BaF3 cells with E3 (10 μM) (right). EGFR antibodies were used in western blots. **c** Competitive labeling of endogenous EGFR (L858R) with H3255 live cells in the presence of afatinib or erlotinib (5×) as a competitor.

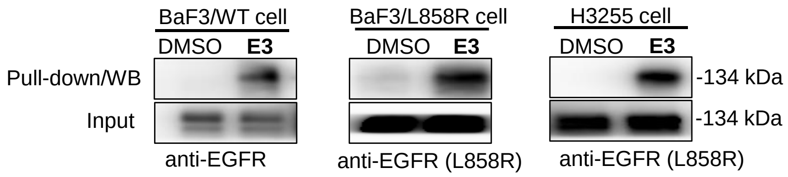

Supplementary Fig. 17 Pull-down/WB validation of EGFR WT in Ba/F3 cells (left), EGFR L858R in Ba/F3 cells (middle) and H3255 cells (right) with E3 (10 μM), the western blots were performed using EGFR antibodies.

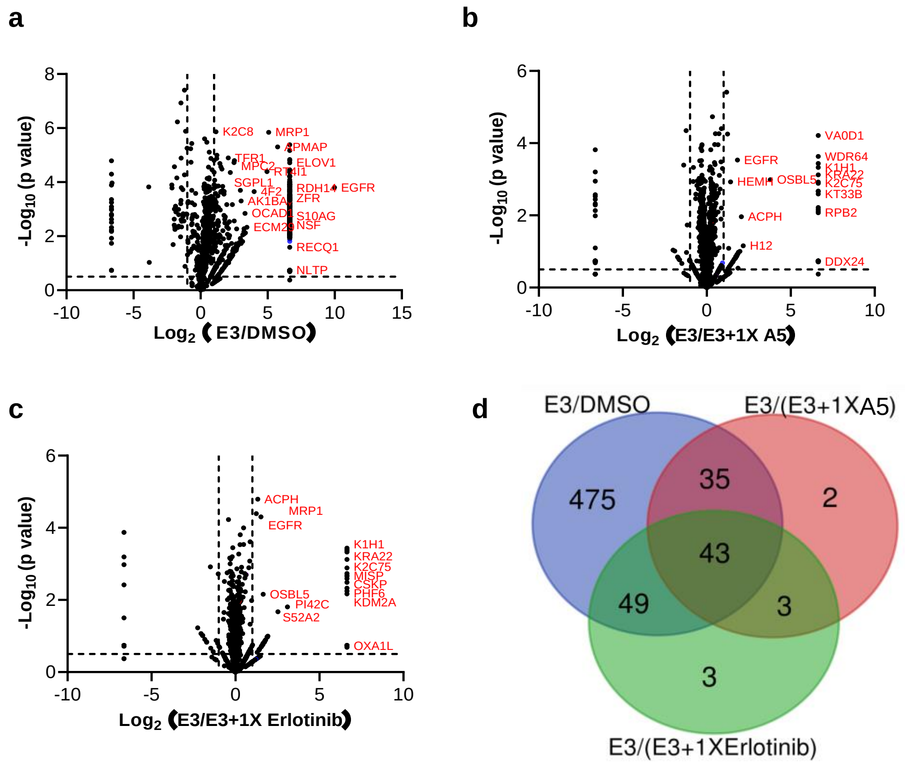

Supplementary Fig. 18 Mass spectrometry-based profiling cellular targets from H3255 cancer cells. a E3/DMSO, **b** E3/(E3+1×A5) and **c** E3/(E3+1×Erlotinib) binding proteins (10 μM probe concentration), DMSO-treated sample was used as a negative control. **d** Venn diagram explains the coincidence and difference of E3/DMSO, E3/(E3+1×A5) and E3/(E3+1×Erlotinib) targets.

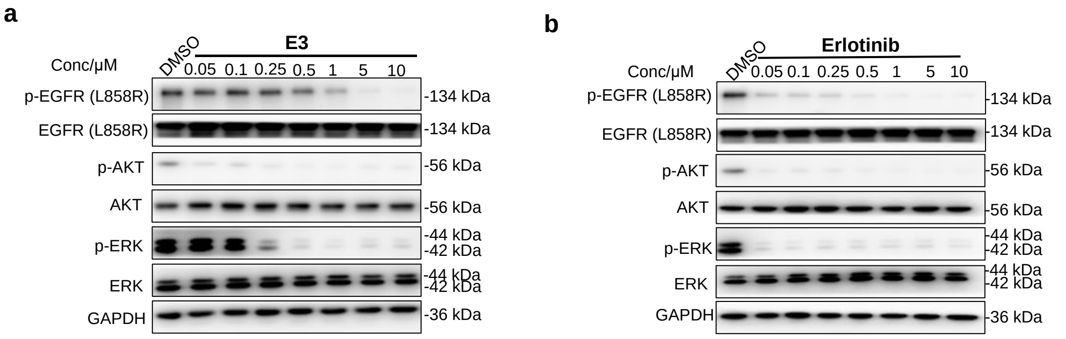

Supplementary Fig. 19 Effect on the phosphorylation level of EGFR and its downstream proteins, including AKT and ERK in H3255 cells treated with E3 or Erlotinib at different concentrations.

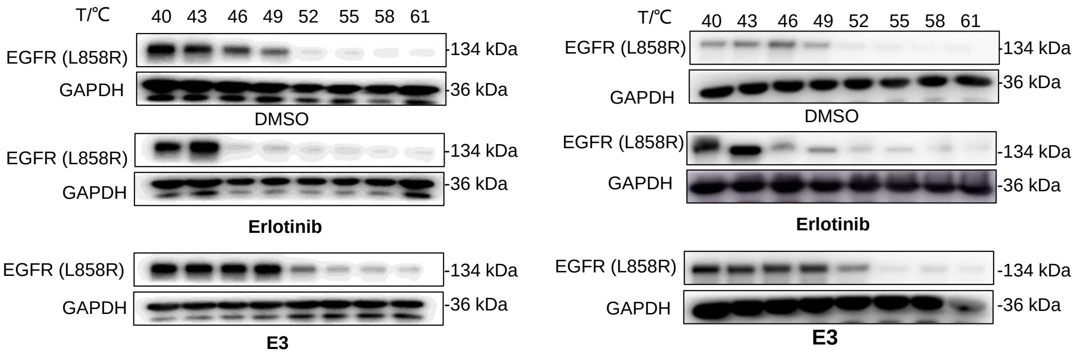

Supplementary Fig. 20 Cellular thermal shift assay evaluation of the binding between the compound E3/Erlotinib and EGFR (L858R).

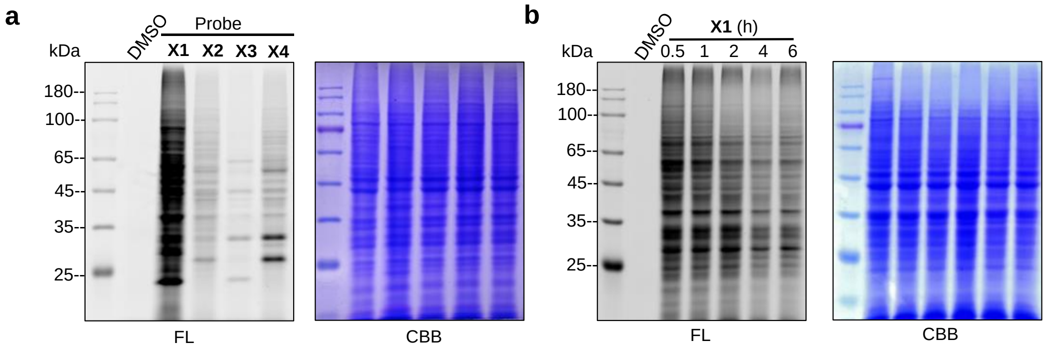

Supplementary Fig. 21 Labeling profiles of X1/X2/X3/X4. a Proteome reactivity profiles of K562 live cells with X1/X2/X3/X4 (10 μM). **b** Time-dependent labeling profiles of K562 live cells with X1.

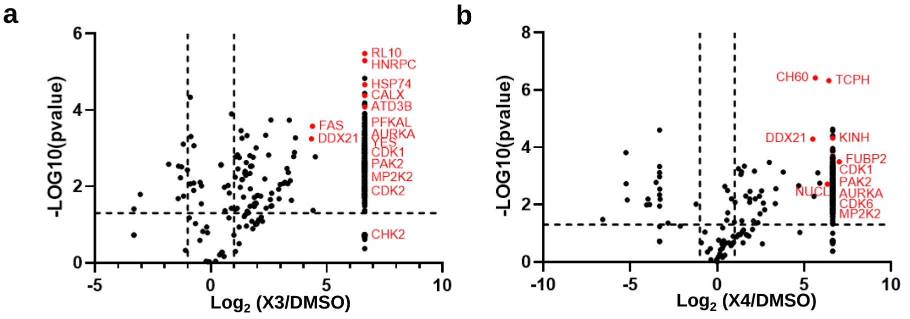

Supplementary Fig. 22 Mass spectrometry-based profiling of X3/X4 binding proteins (10 μM probe concentration), DMSO-treated sample was used as a negative control. The MS data of X1/X2 were presented in main text (Fig. 7b).

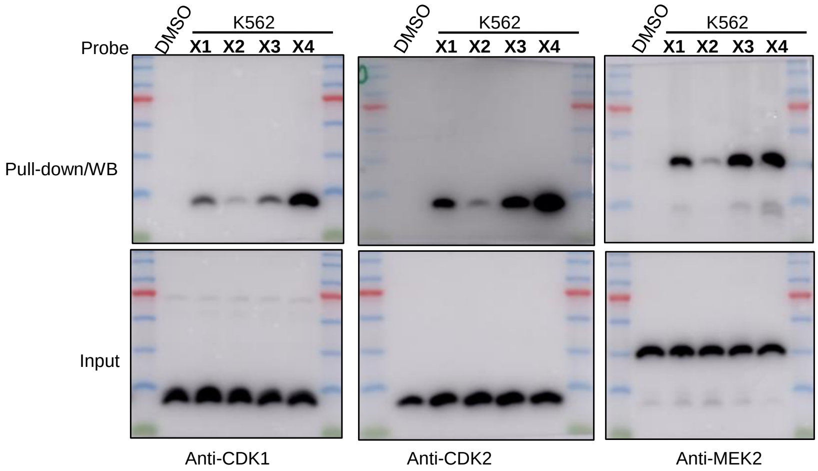

Supplementary Fig. 23 Pull-down/WB validation of CDK1/CDK2/MEK2 in K562 cells with X1/2/3/4 (10 µM) in full gel.

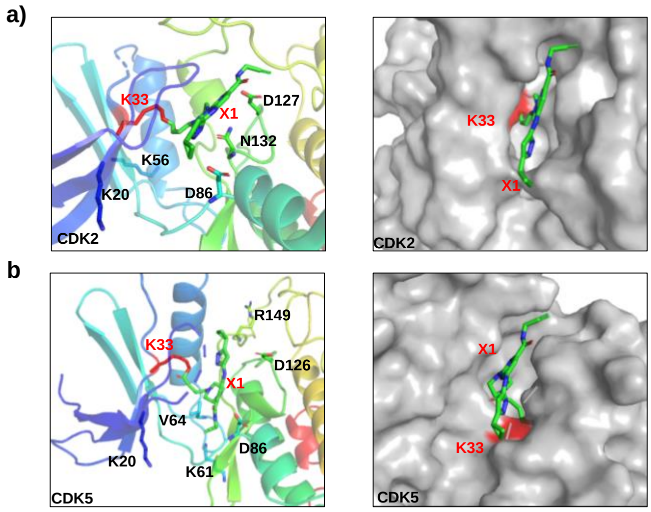

Supplementary Fig. 24 Docking experiment to predict the binding mode. a X1 with CDK2 (PDB code: 1B38). **b** X1 with CDK5 (PDB code: 1H4L). The data of CDK1 was presented in main text (Fig. 8).

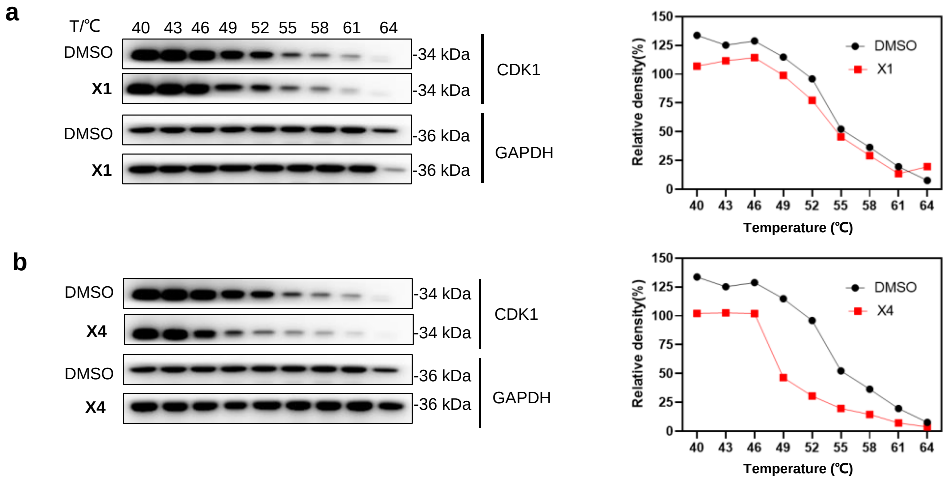

Supplementary Fig. 25 Cellular thermal shift assay to evaluate the binding between the compound X1/X4 and CDK1.

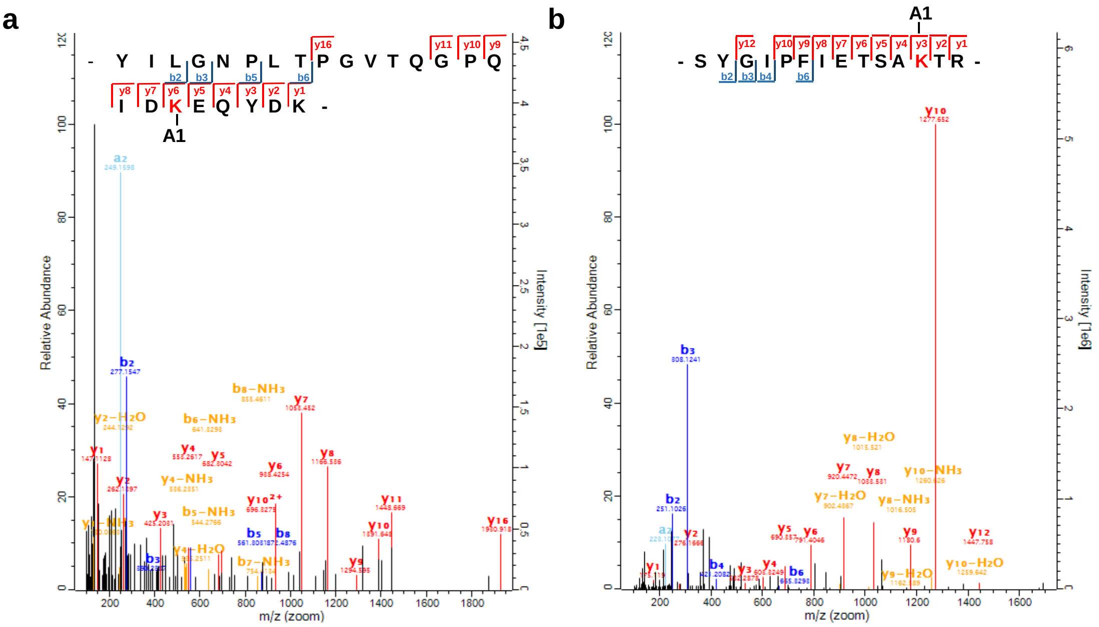

Supplementary Fig. 26 Representative spectra of peptides labeled by A1 (100 µM). a from ALDH1A1, modification of the Lys348 residue. **b** from KRAS G12D, modification of the Lys147 residue. The modification sites identified by MS2 spectra are highlighted (red).

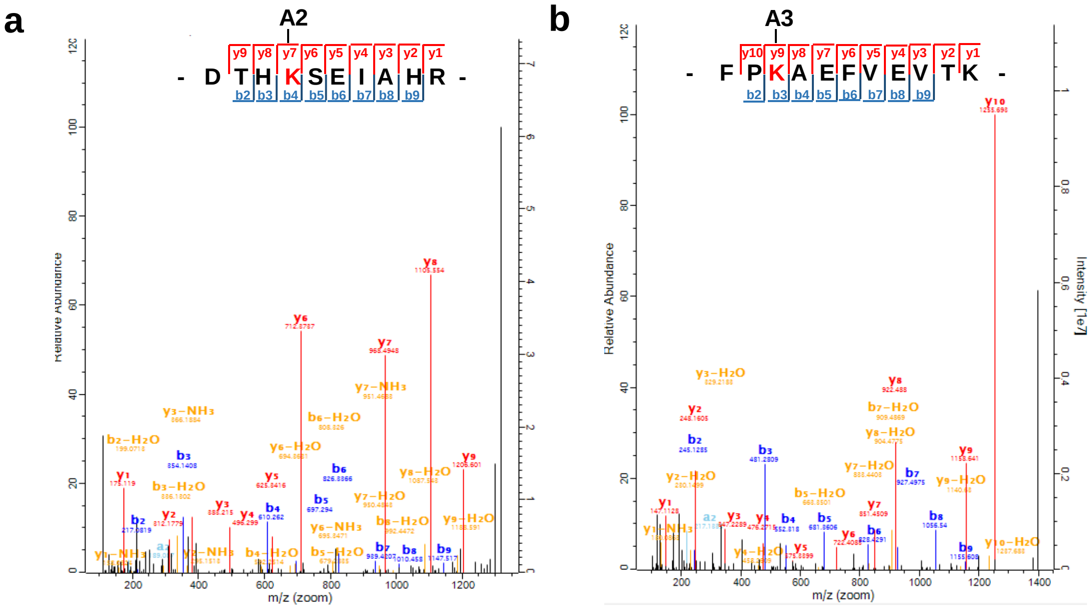

Supplementary Fig. 27 Representative spectra of peptides labeled by A2 and A3. a A2 (100 μM) from BSA, modification of the Lys28 residue. **b** A3 (100 μM) from BSA, modification of the Lys248 residue. The modification sites identified by MS2 spectra are highlighted (red).

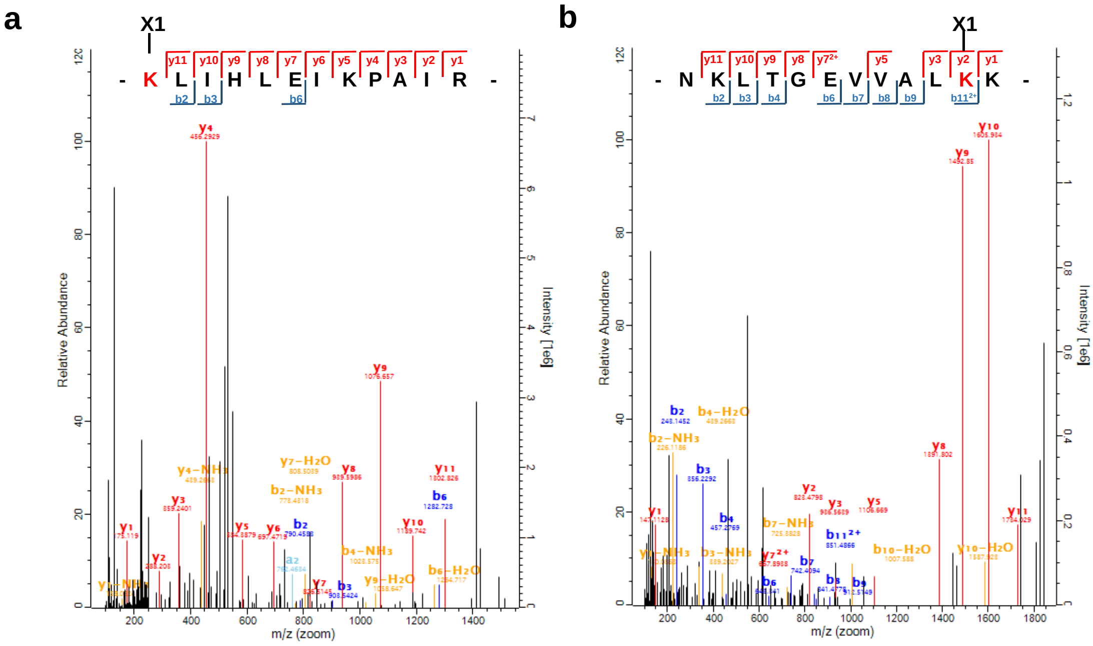

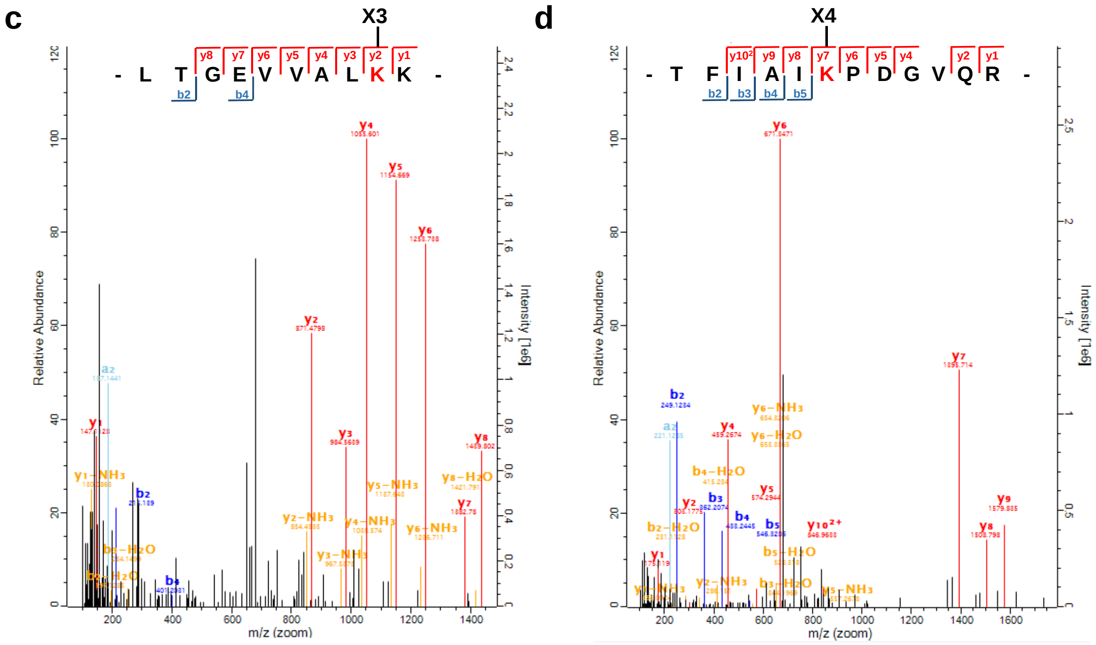

Supplementary Fig. 28 Representative spectra of peptides labeled by X1/X3/X4 from K562 cell lysates. a Representative spectra of modification by X1 from MEK2, modification of the Lys101 residue. **b** Representative spectra of peptides labeled by X1 from CDK2, modification of the Lys33 residue. **c** Representative spectra of peptides labeled by X3 from CDK2, modification of the Lys33 residue. **d** Representative spectra of peptides labeled by X4 from NDKB, modification of the Lys12 residue, the modification sites identified by MS2 spectra are highlighted (red).

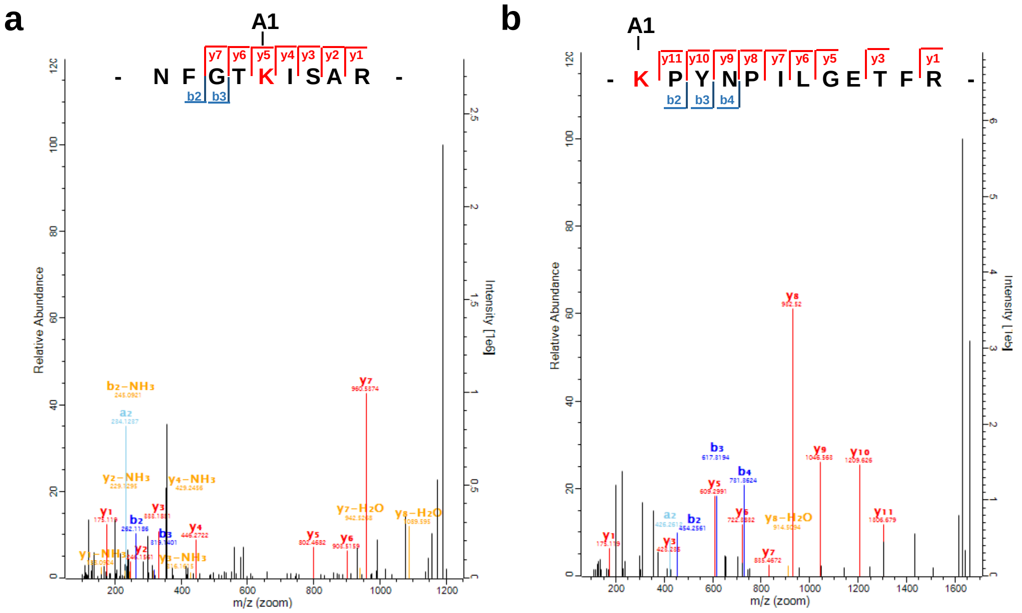

Supplementary Fig. 29 Representative spectra of peptides labeled by A1 from MDA-MB-231 live cells. a Representative spectra of peptides labeled by A1 from PFKAP, modification of the Lys688 residue. **b** Representative spectra of peptides labeled by A1 from OSBL5, modification of the Lys446 residue. The modification sites identified by MS2 spectra are highlighted (red).
