# Supplementary material for: Catalyst-free late-stage functionalization to assemble α-acyloxyenamide electrophiles for selectively profiling conserved lysine residues
Source: Commun Chem. 2024 Feb 14;7:31. doi: 10.1038/s42004-024-01107-4 (PMC10866925; doi:10.1038/s42004-024-01107-4)
Supplement: Supplementary file 2 — Description of Additional Supplementary Files [file 42004_2024_1107_MOESM2_ESM.pdf]

Description of Additional Supplementary Files

File name: Supplementary Data 1

Description: Source data underlying the graphs and charts presented in the main figures

File name: Supplementary Data 2

Description: NMR spectra
