# Supplementary material for: Catalyst-free late-stage functionalization to assemble α-acyloxyenamide electrophiles for selectively profiling conserved lysine residues
Source: Commun Chem. 2024 Feb 14;7:31. doi: 10.1038/s42004-024-01107-4 (PMC10866925; doi:10.1038/s42004-024-01107-4)
Supplement: Supplementary file 4 — Supplementary Data 2 [file 42004_2024_1107_MOESM4_ESM.pdf]

Supplementary Data 2

Catalyst-Free Late-Stage Functionalization to Assemble α-acyloxyenamide Electrophiles for Selectively Profiling Conserved Lysine Residues

Yuanyuan Zhao,[1,2,8] Kang Duan,[1,2,8] Youlong Fan,[1,2,8] Shengrong Li,[3,8] Liyan Huang,[1,2] Zhengchao Tu,[1,2] Hongyan Sun,[4] Gregory M. Cook,[5] Jing Yang,[6] Pinghua Sun,[1,2] Yi Tan,[*1,2] Ke Ding,[*1,2] Zhengqiu Li[*1,2,7]

[1]State Key Laboratory of Bioactive Molecules and Druggability Assessment, Jinan University, 601 Huangpu Avenue West, Guangzhou, 510632 China

[2]International Cooperative Laboratory of Traditional Chinese Medicine Modernization and Innovative Drug Development (MOE), School of Pharmacy, Jinan University, 601 Huangpu Avenue West, Guangzhou, 510632 China

[3]Guangdong Second Provincial General Hospital, Postdoctoral Station of Traditional Chinese Medicine, Jinan University, Guangzhou 510632, China

[4]Department of Chemistry and COSDAF (Centre of Super-Diamond and Advanced Films), City University of Hong Kong, 83 TatChee Avenue, Kowloon, Hong Kong, China 999077

[5]Department of Microbiology and Immunology, University of Otago, Dunedin 9054, New Zealand

[6] Guangzhou National Laboratory, Guangzhou International Bio Island, Guangzhou 510005 China

[7]MOE Key Laboratory of Tumor Molecular Biology, Jinan University, 601 Huangpu Avenue West, Guangzhou, 510632 China

[8]These authors contributed equally to this work.

Email: pharmlzq@jnu.edu.cn (Z. Li), dingke@jnu.edu.cn (K. Ding), tanyi@jnu.edu.cn (Y. Tan)

NMR Spectrum

^{1}H of A1

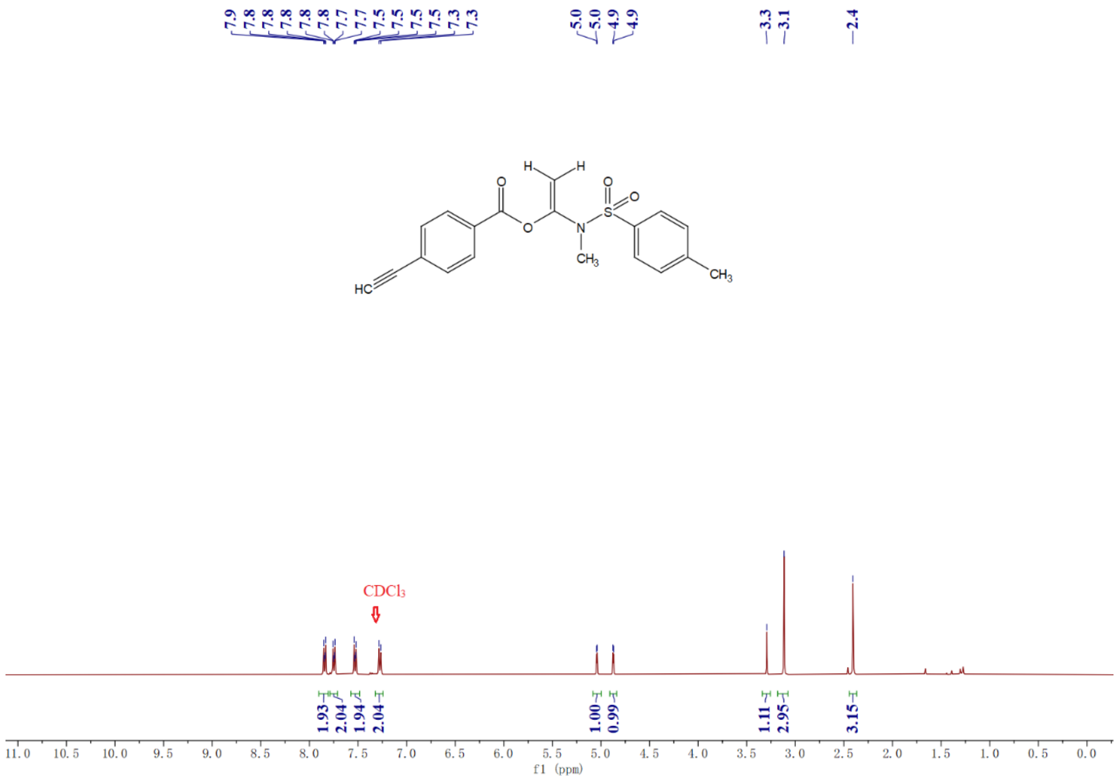

^{13}C of A1

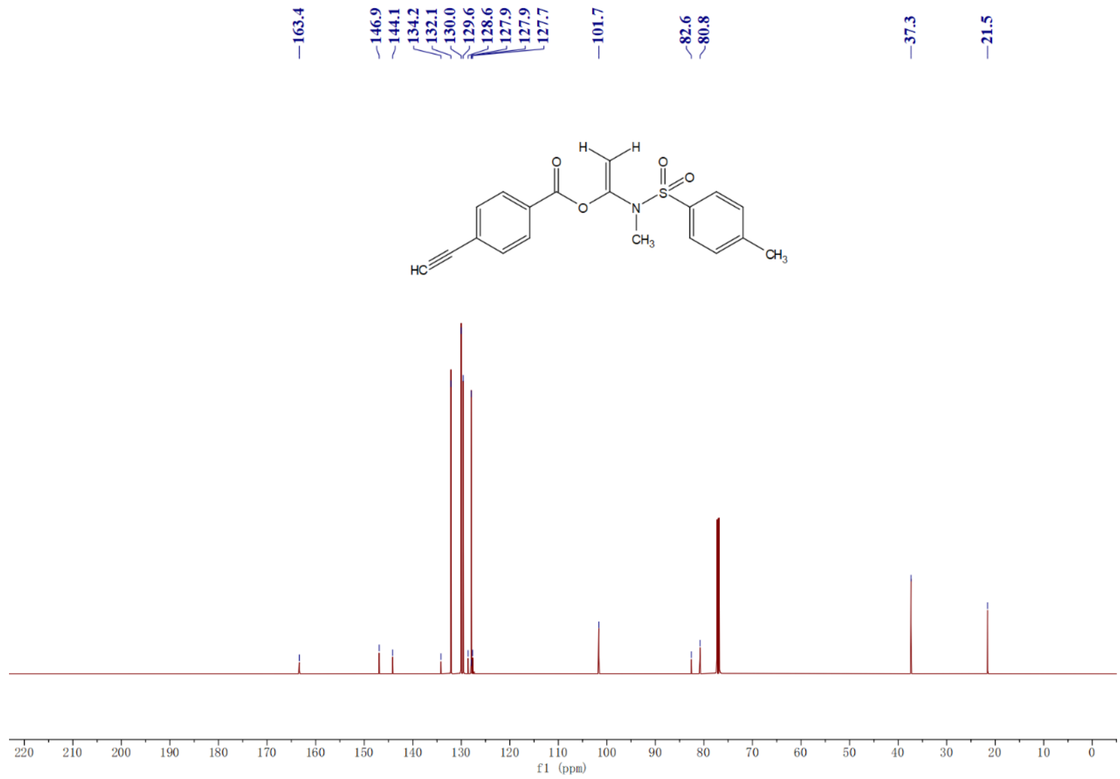

[1]H of A2

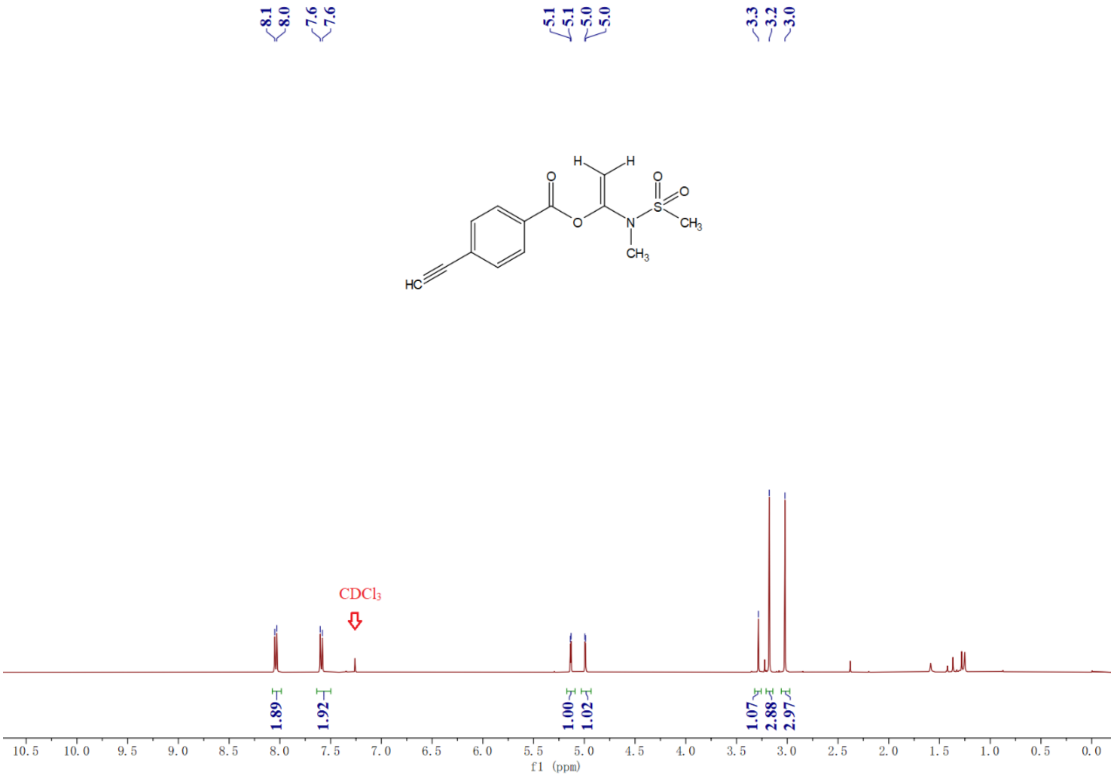

[13]C of A2

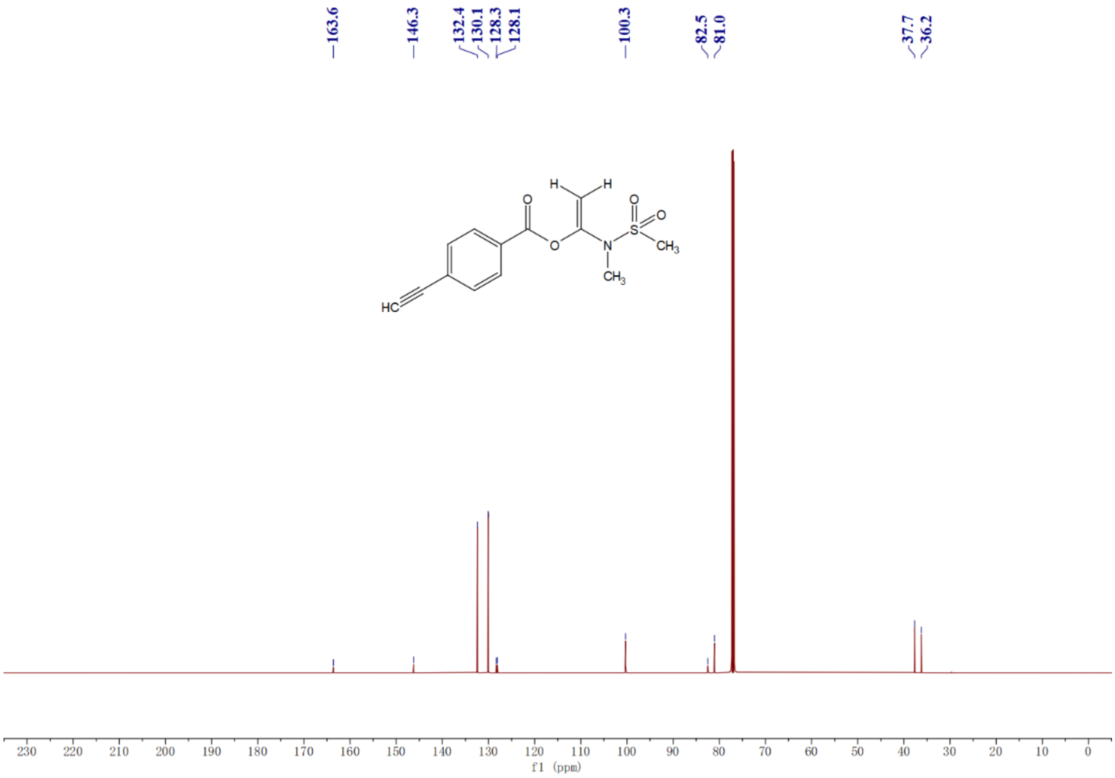

[1]H of A3

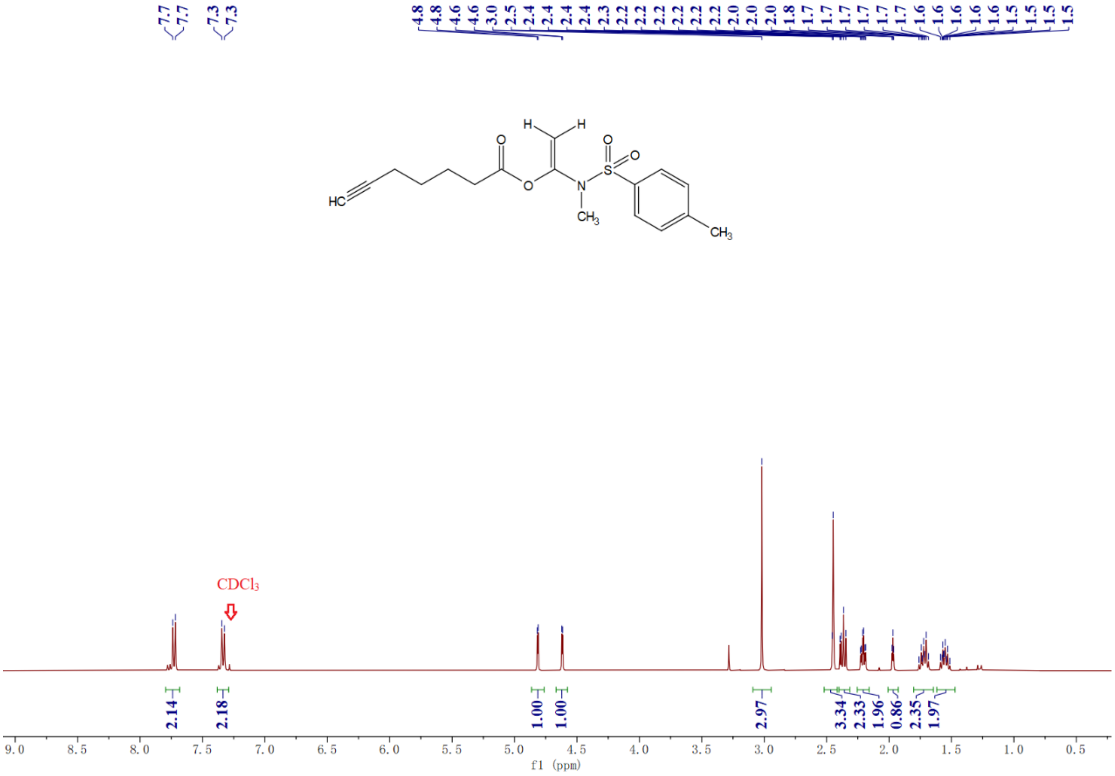

[13]C of A3

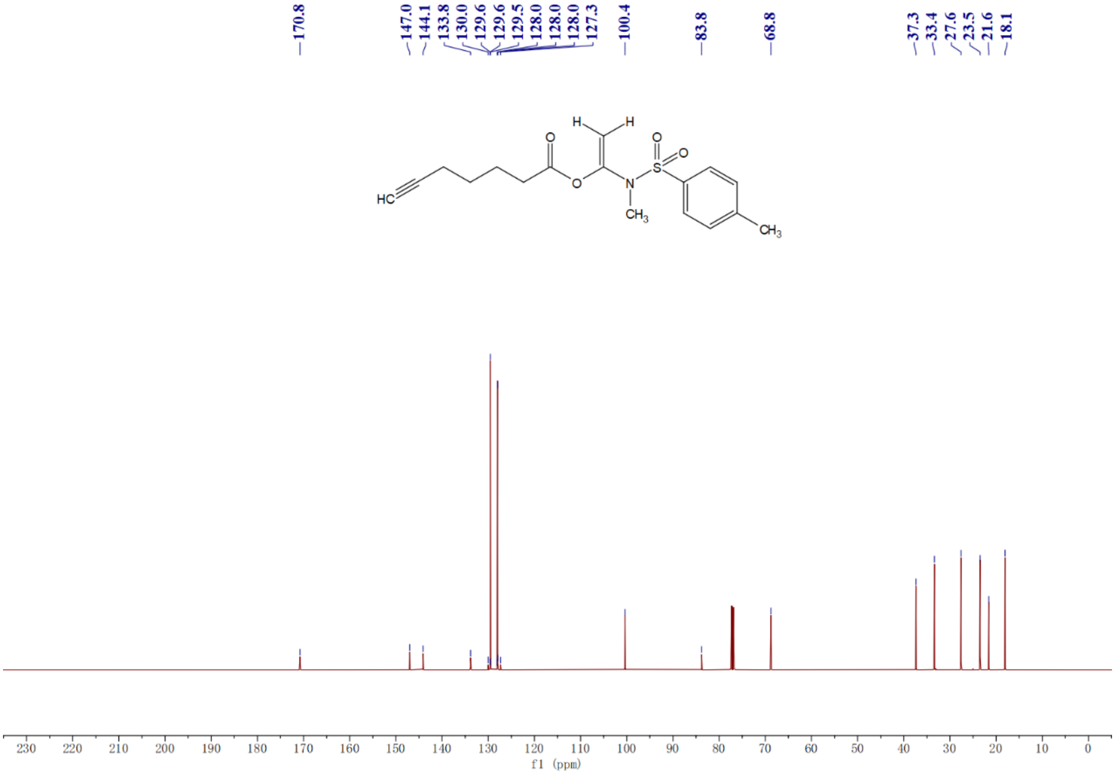

^{1}H of A4

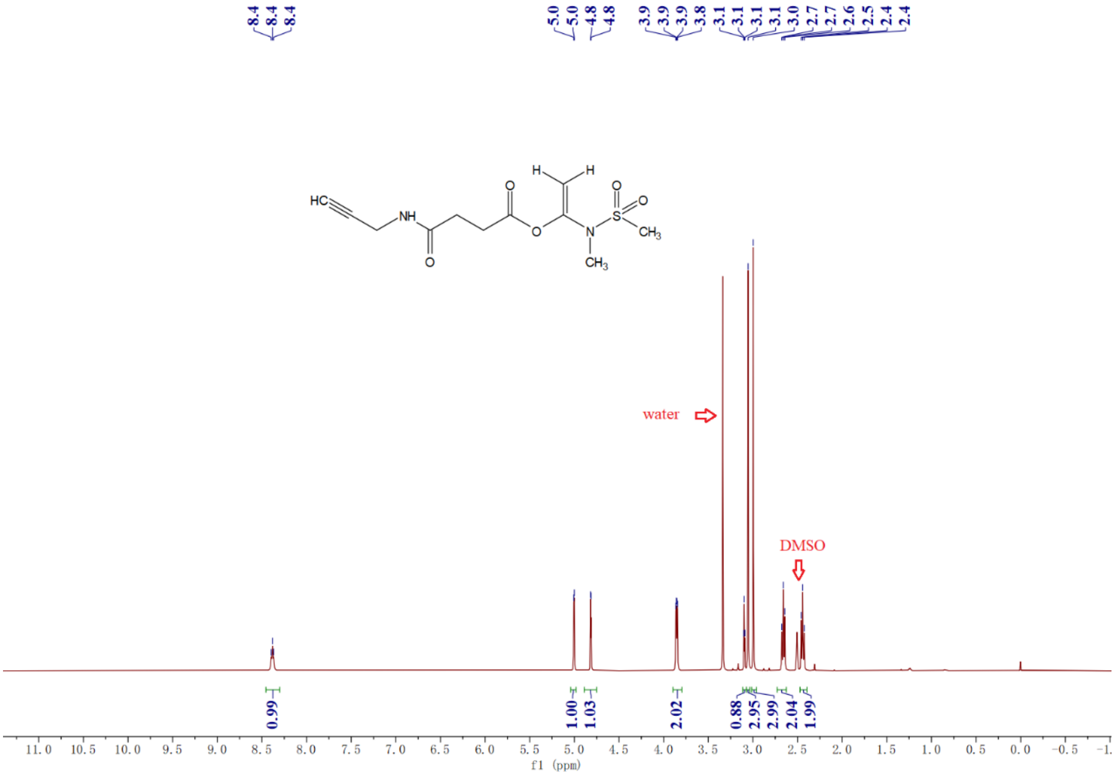

^{13}C of A4

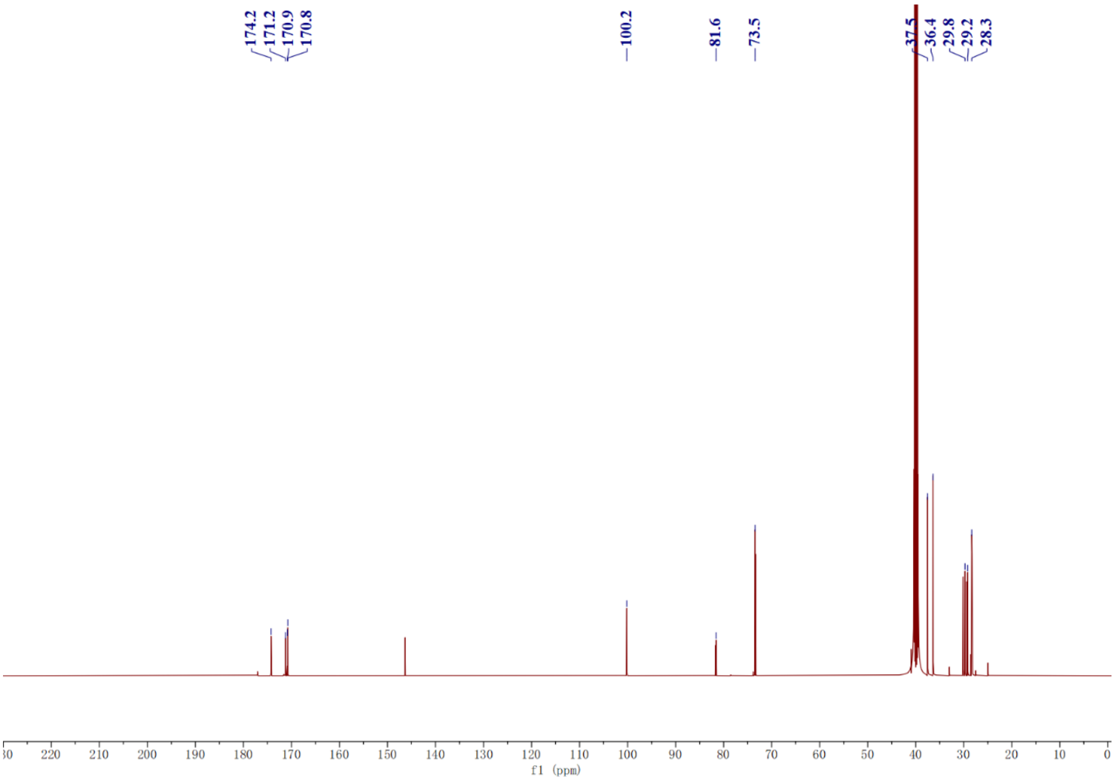

^{1}H of A5

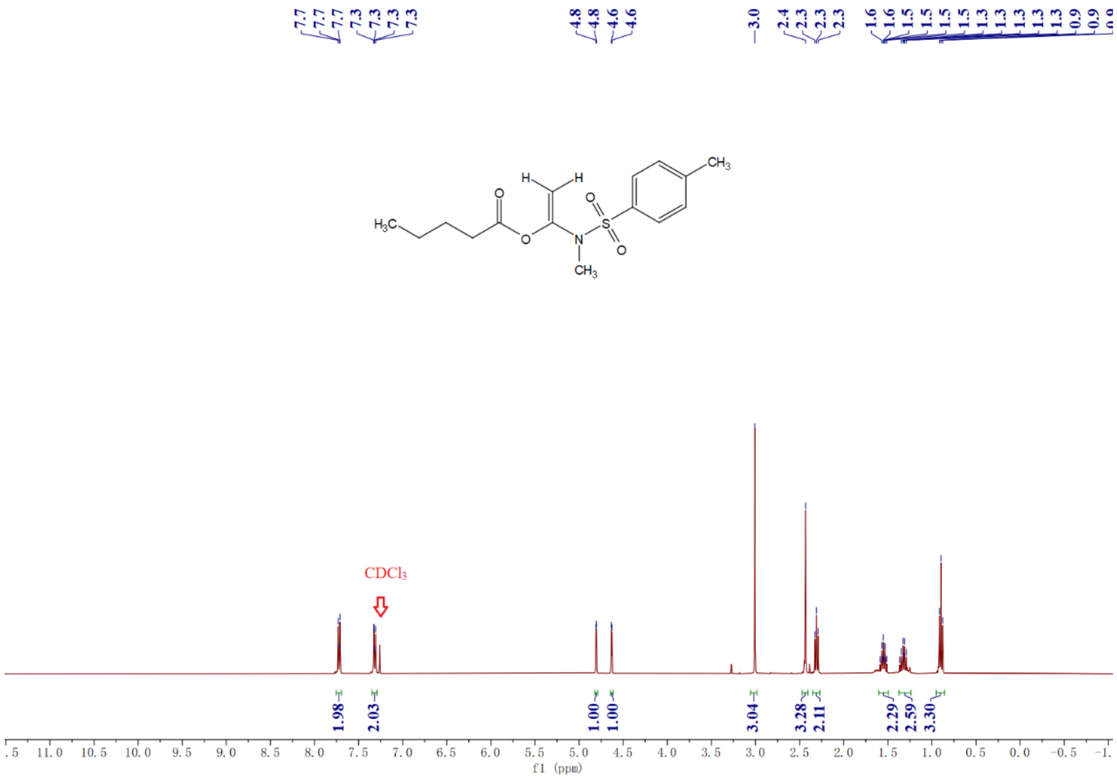

^{13}C of A5

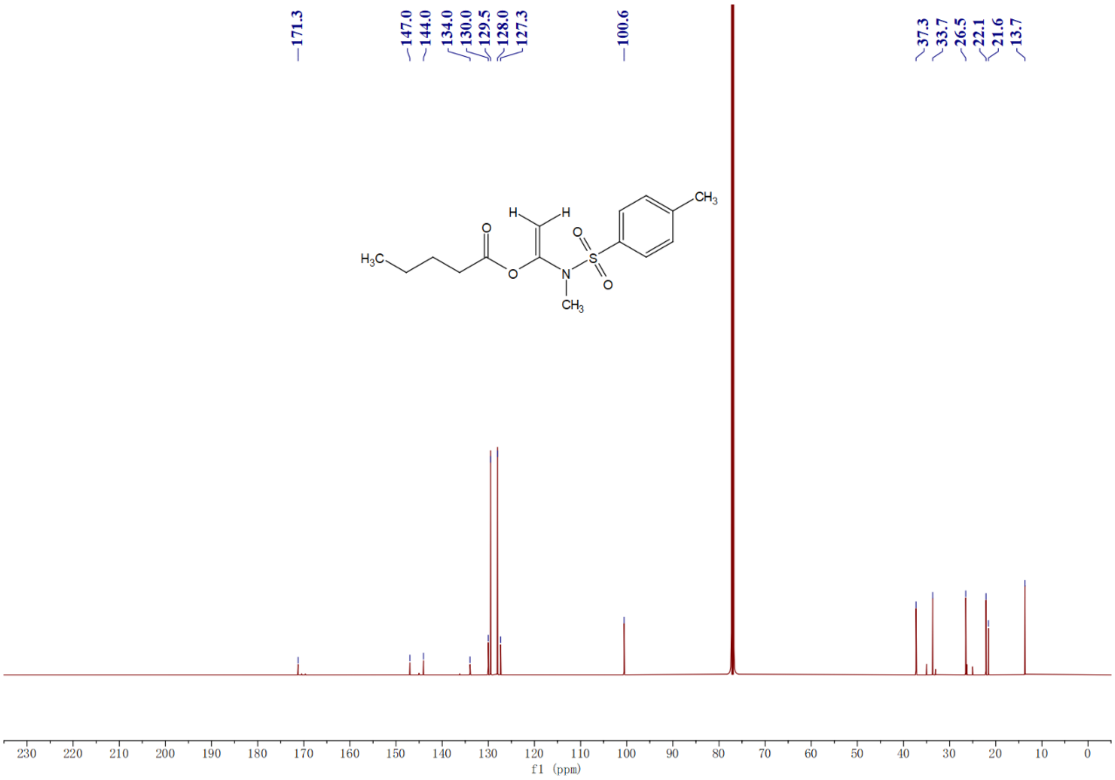

^{1}H of E1

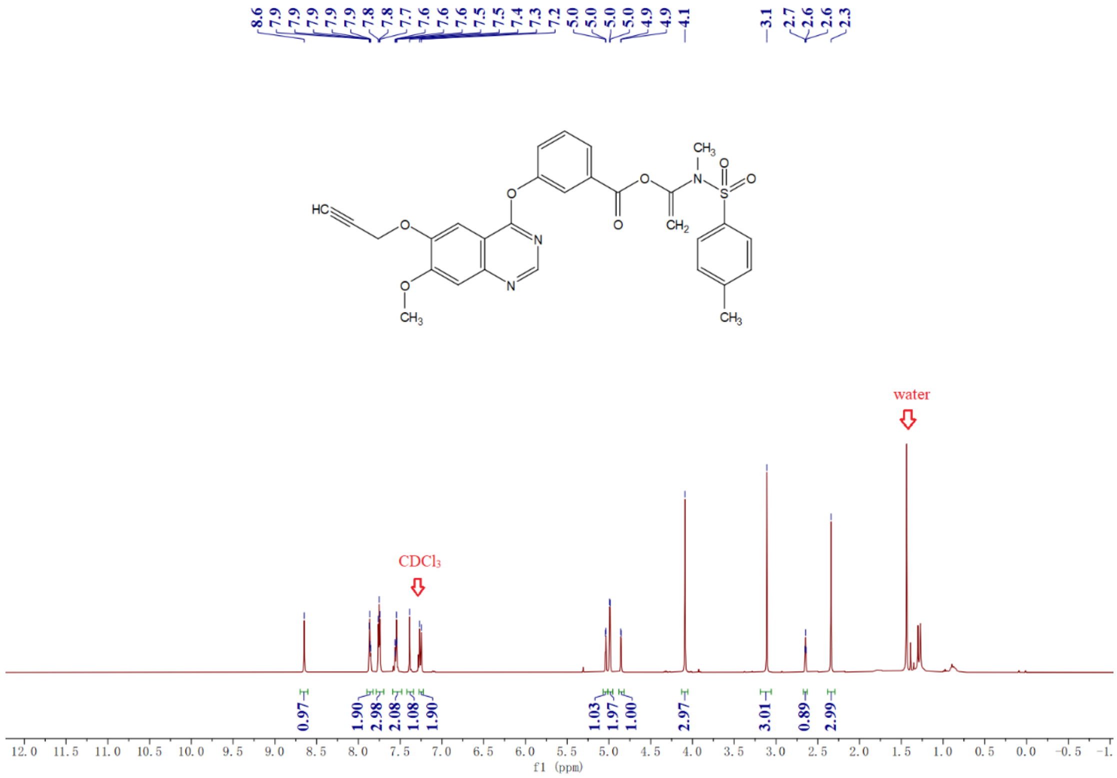

^{13}C of E1

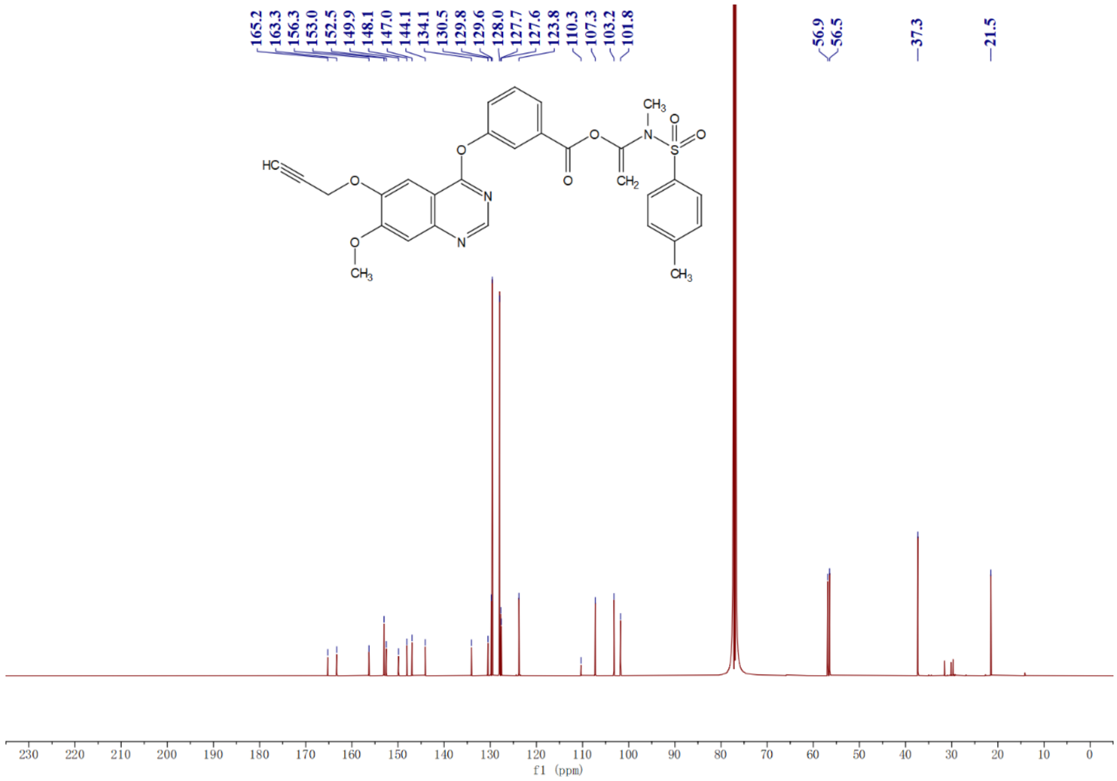

[1]H of E2

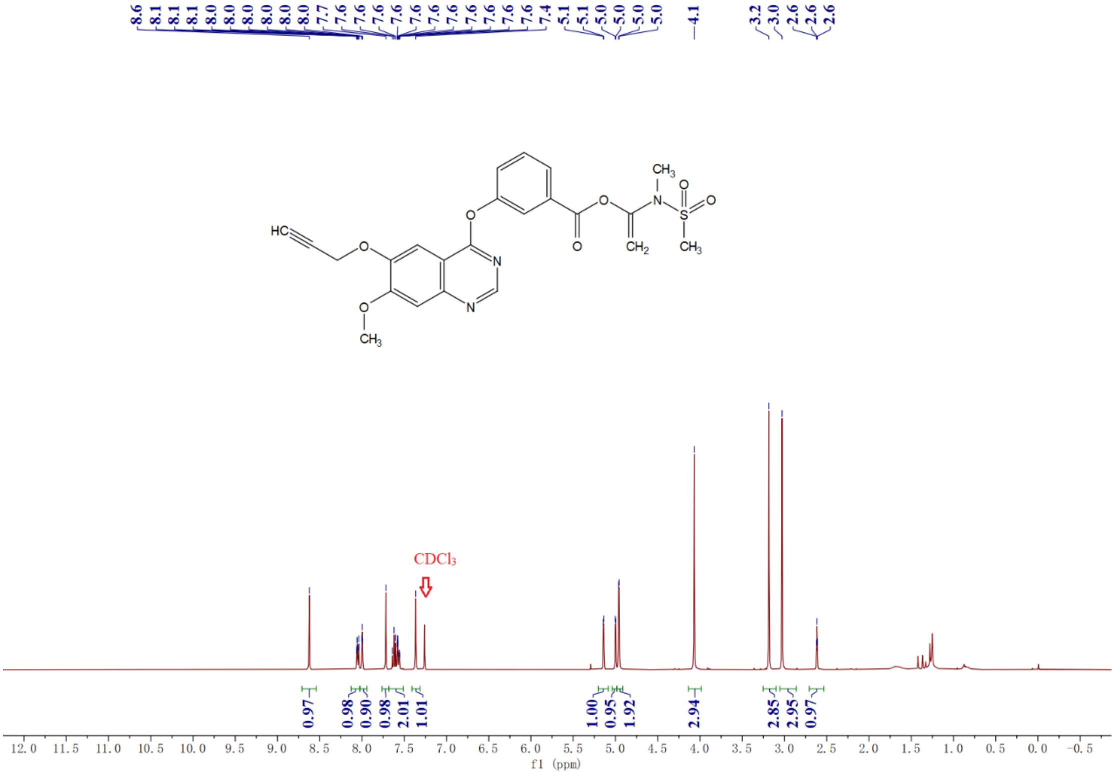

[13]C of E2

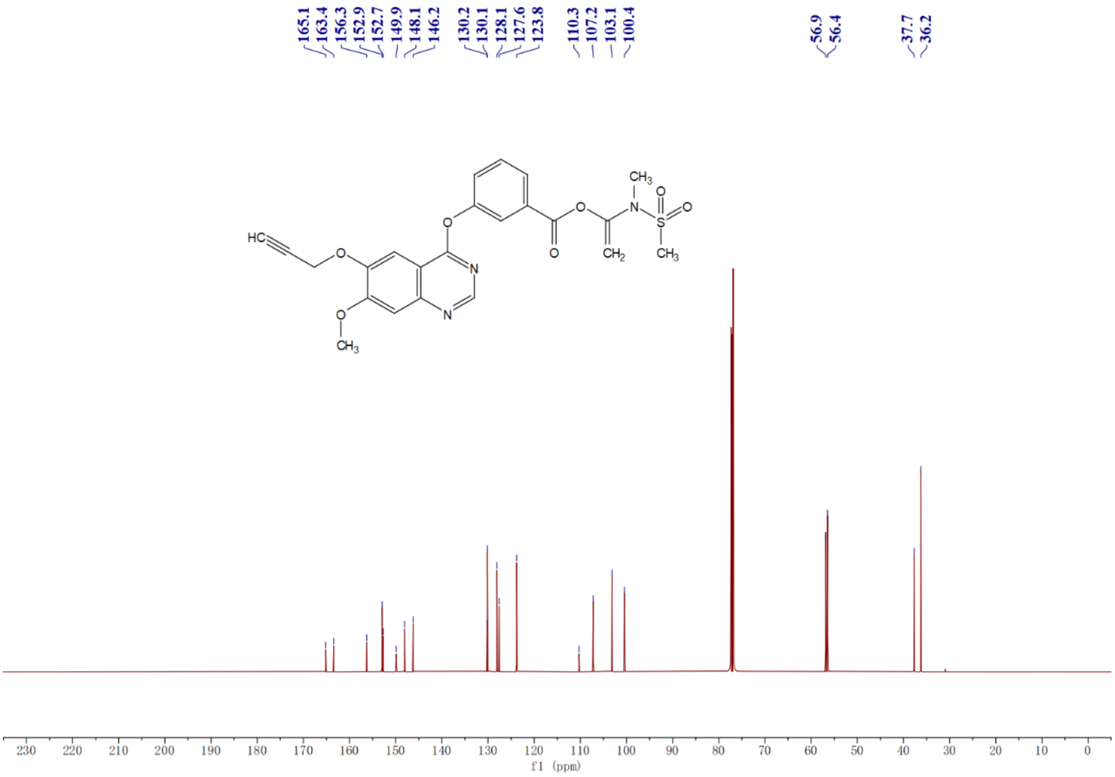

IR of E2

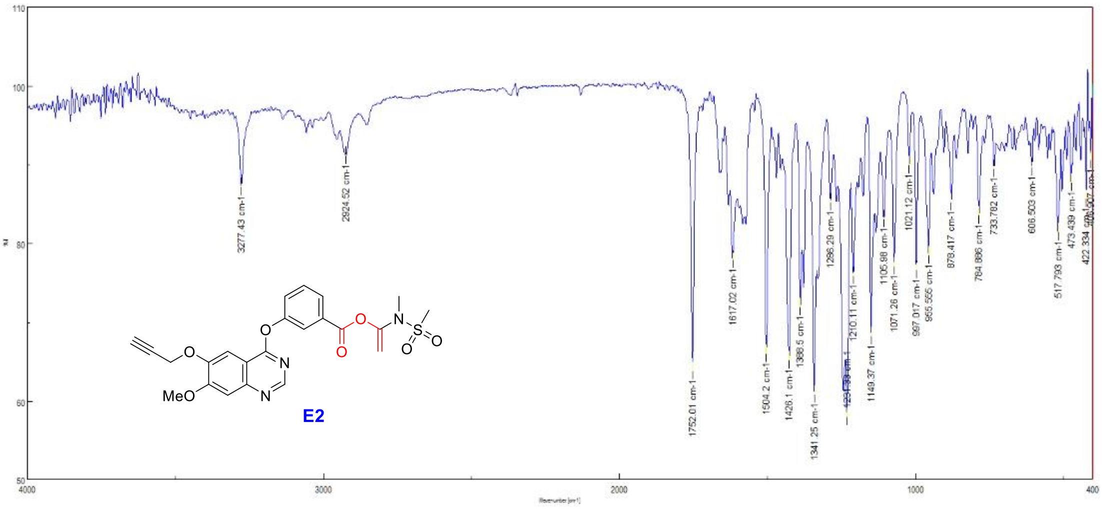

[1]H of E3

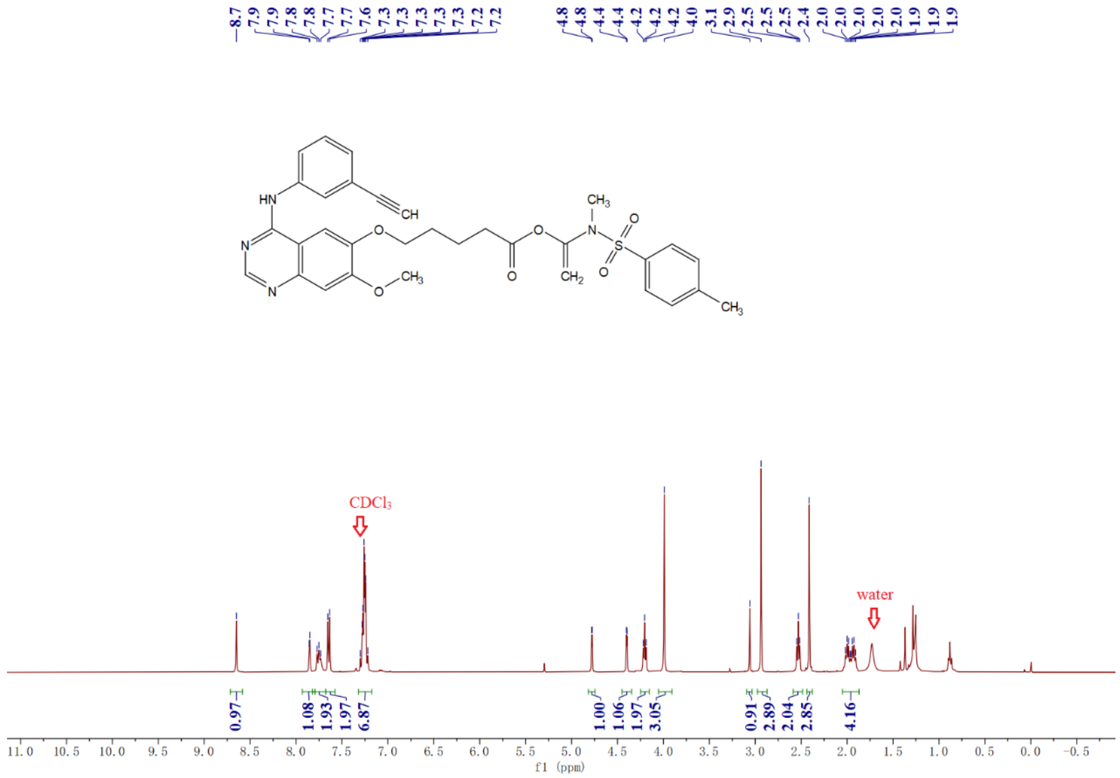

^{13}C of E3

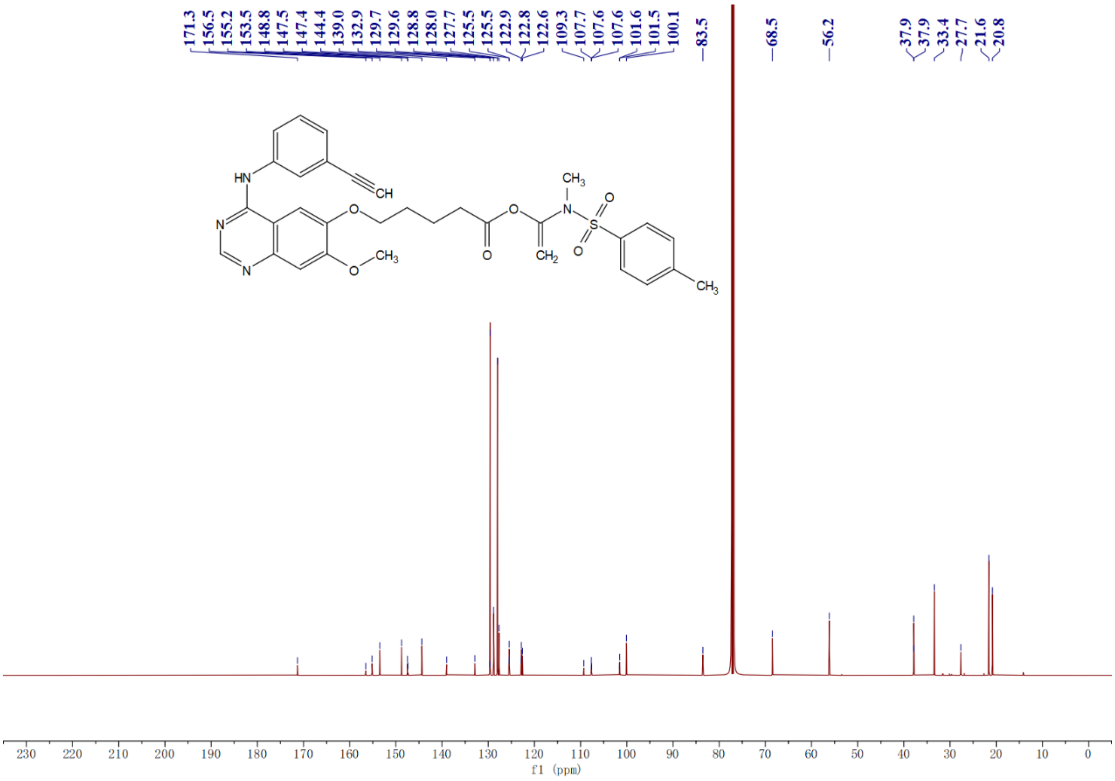

^{1}H of E4

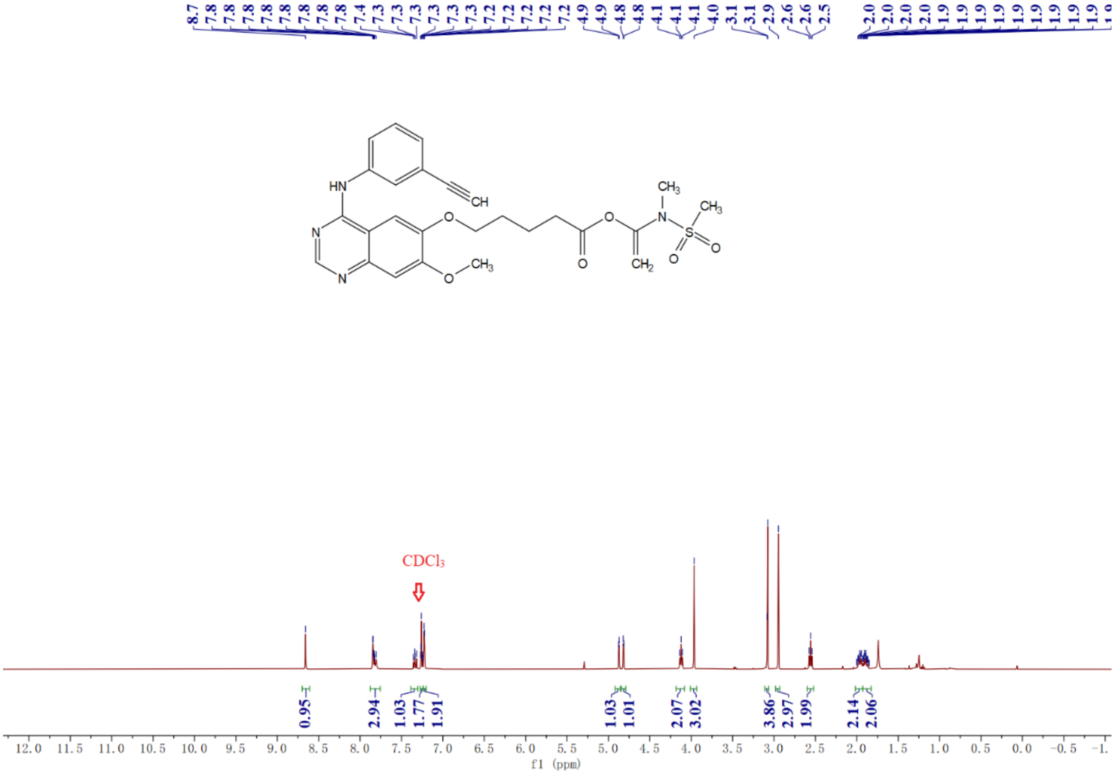

^{13}C of E4

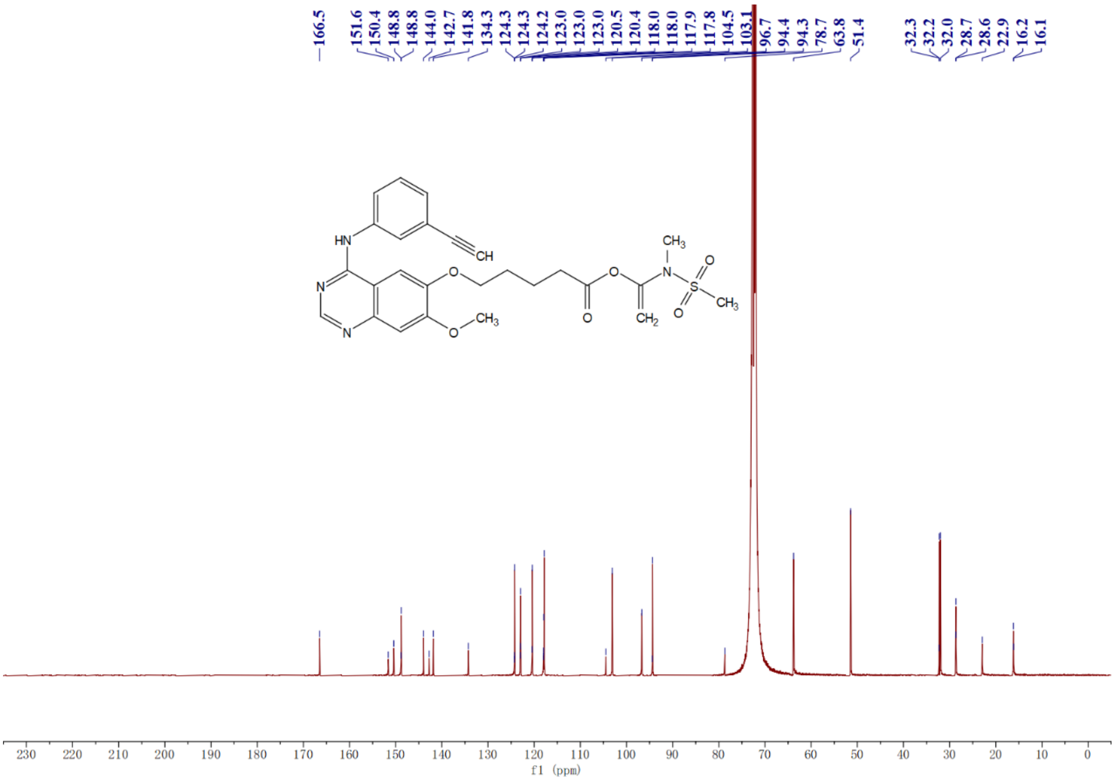

^{1}H of E5

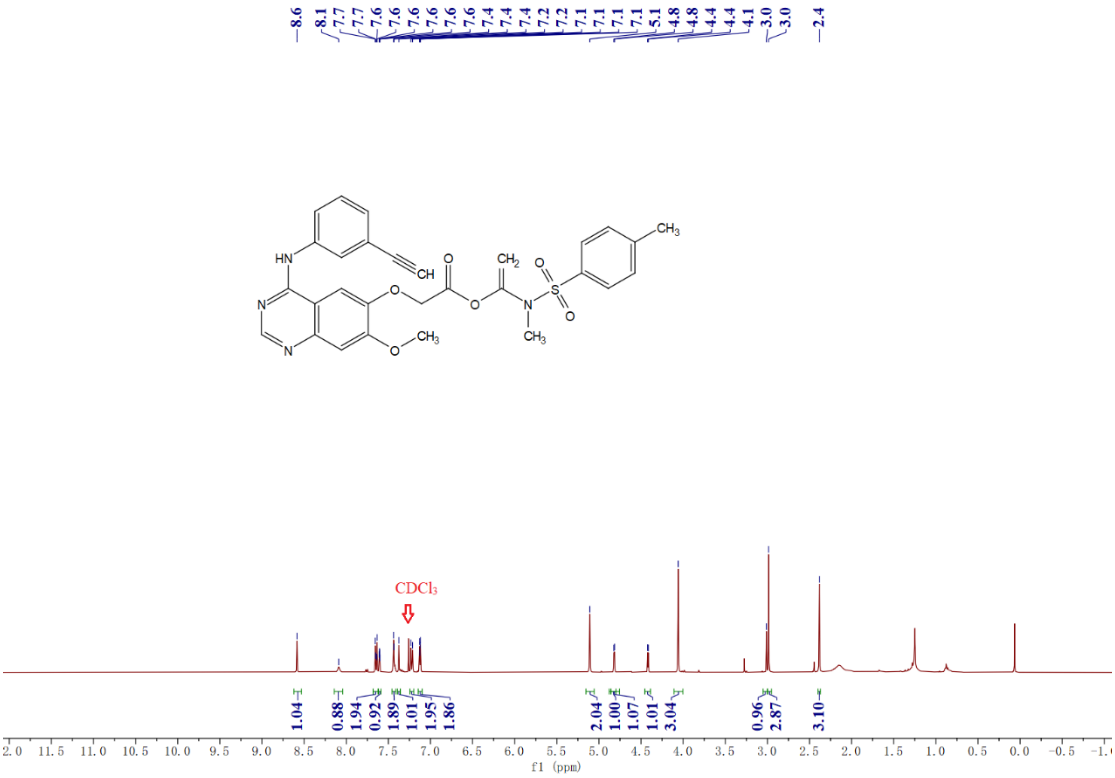

^{13}C of E5

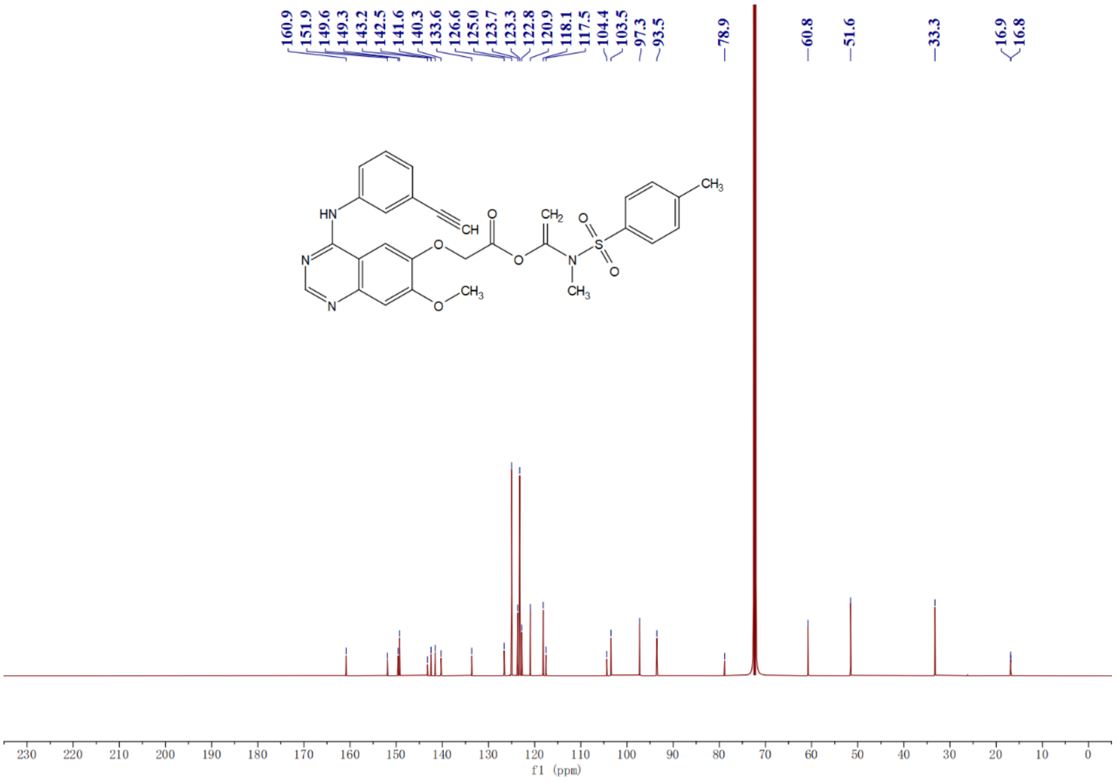

^{1}H of X1

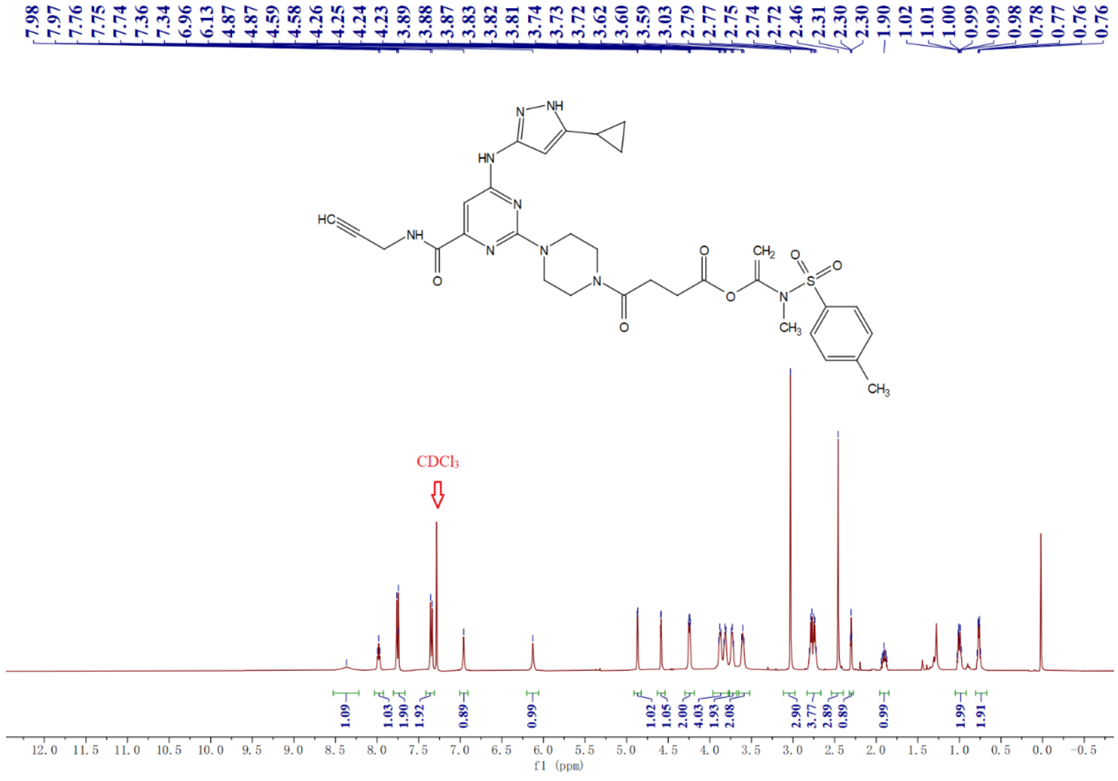

^{13}C of X1

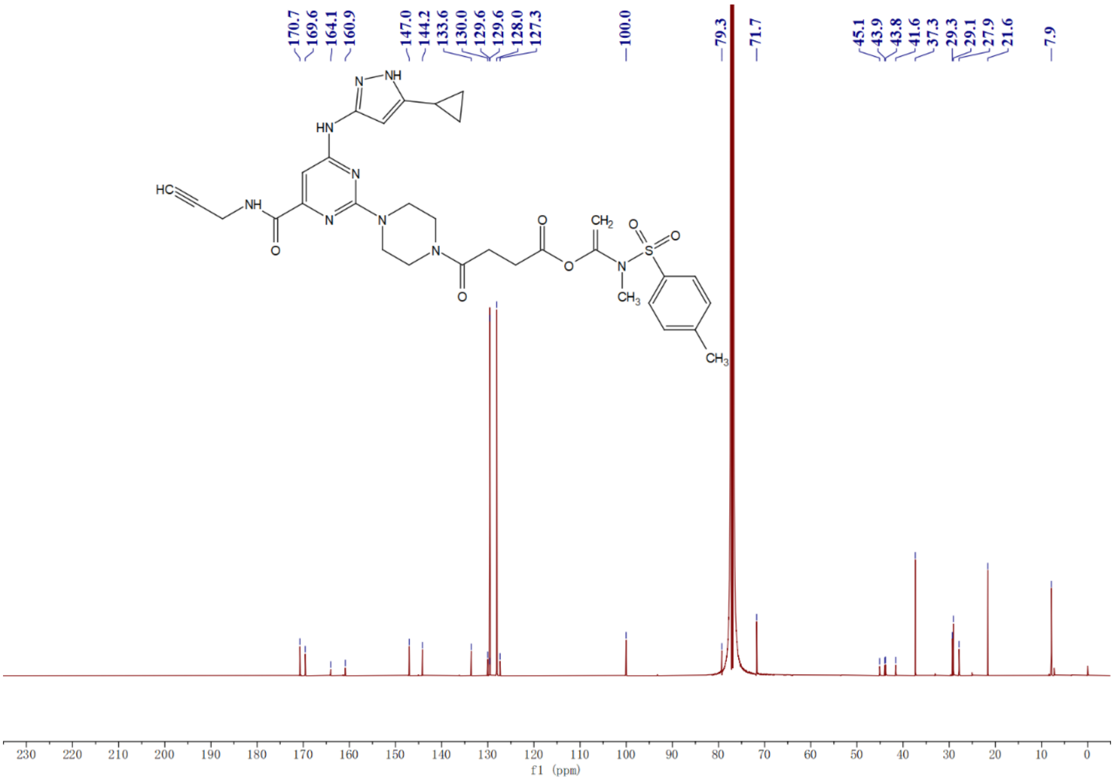

^{1}H of X2

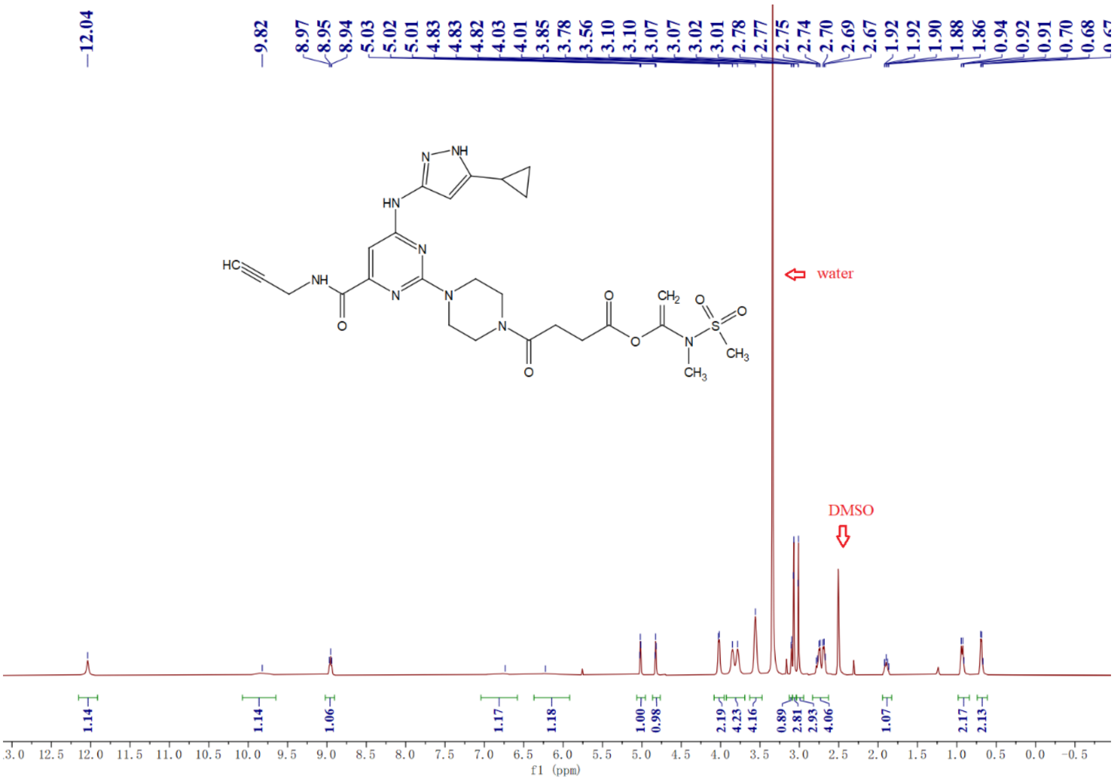

[13]C of X2

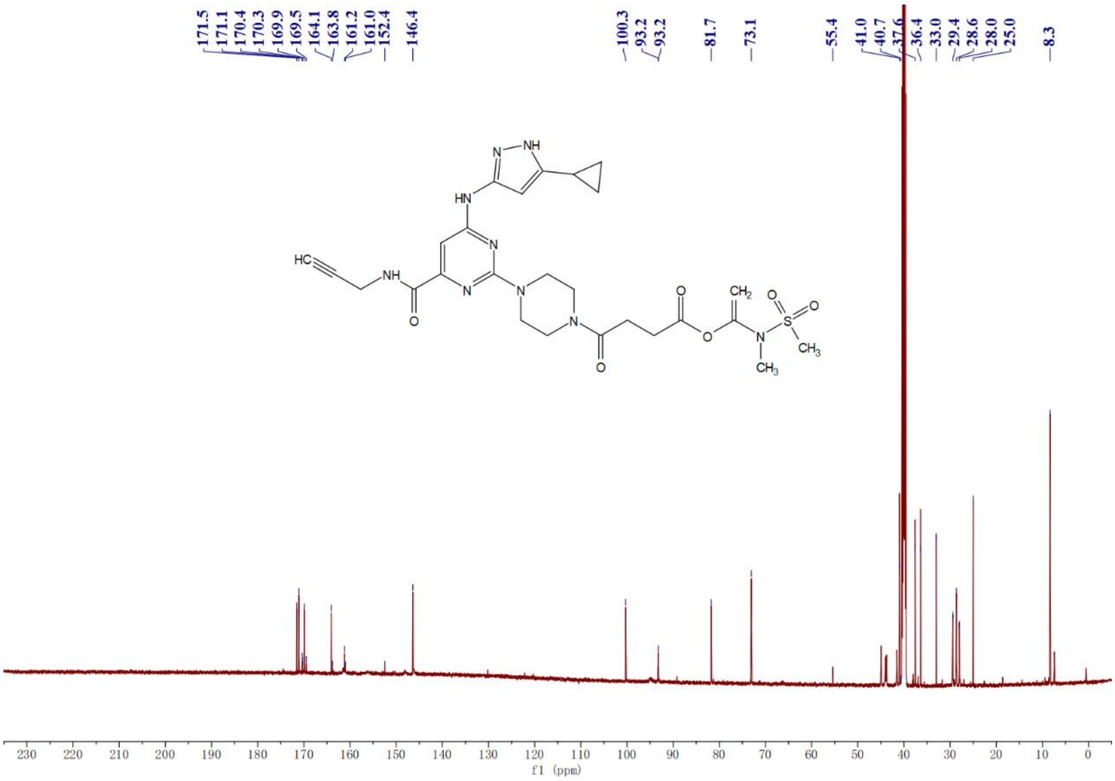

IR of X2

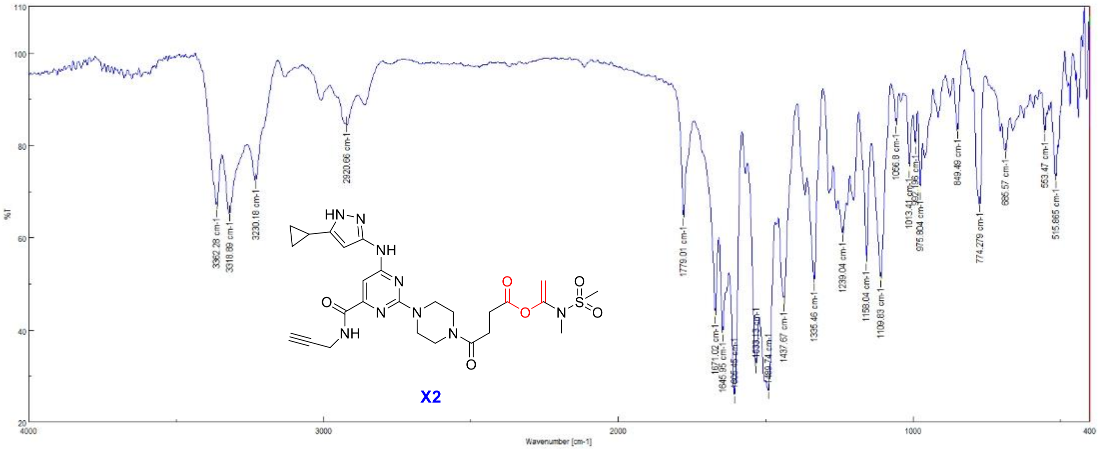

[1]H of X3

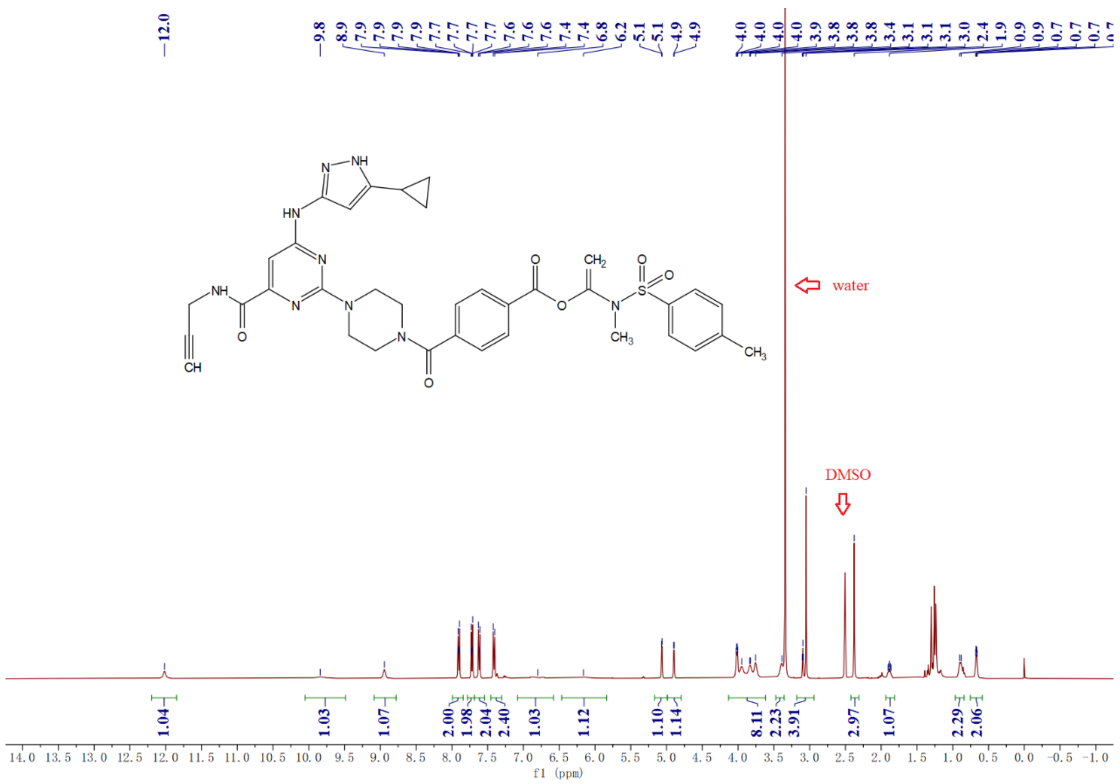

[13]C of X3

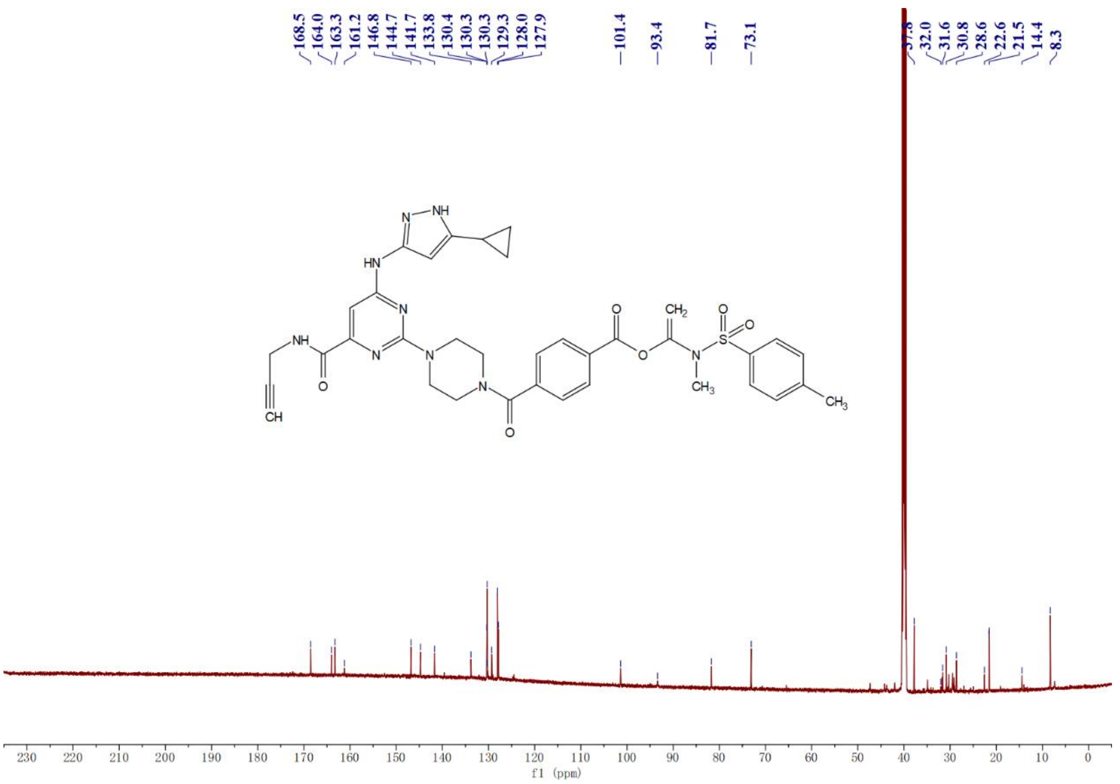

[1]H of X4

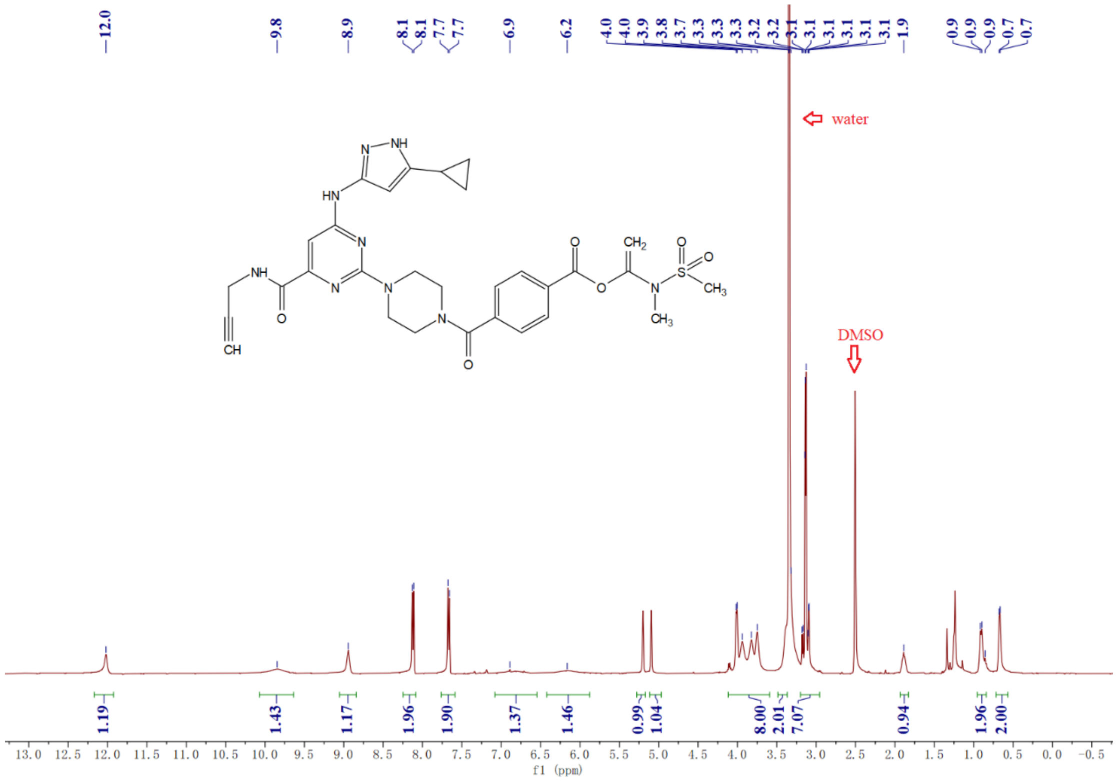

[13]C of X4

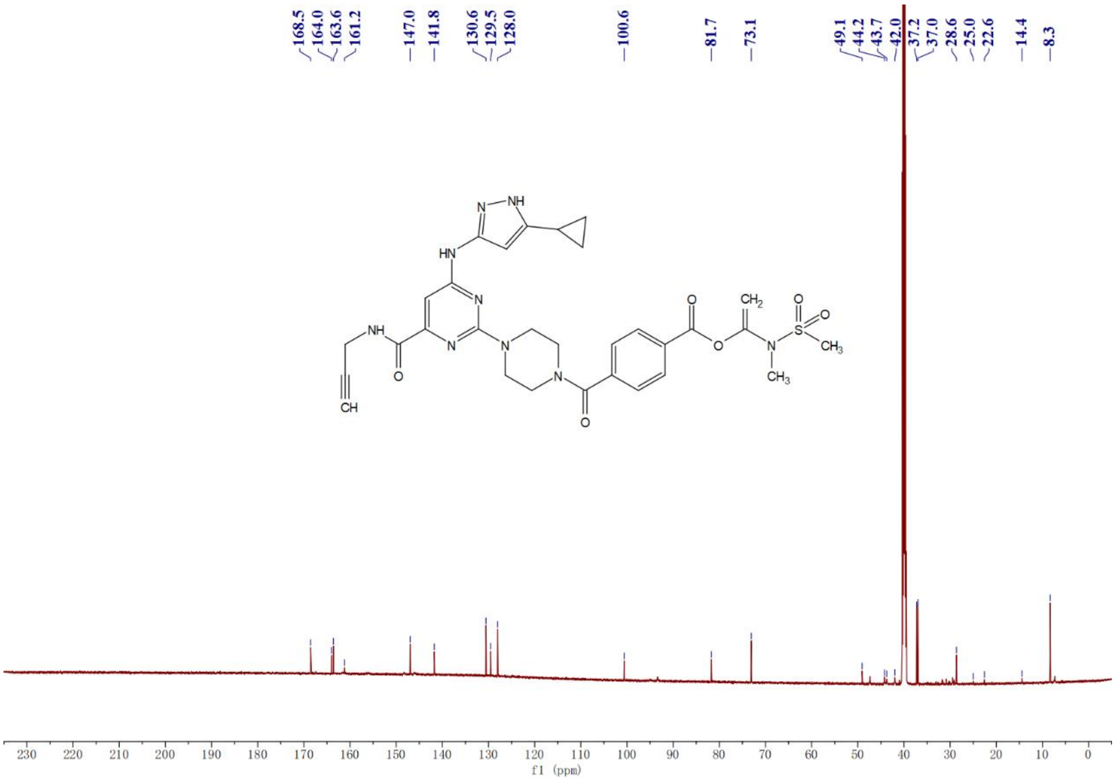

[1]H of BA-1

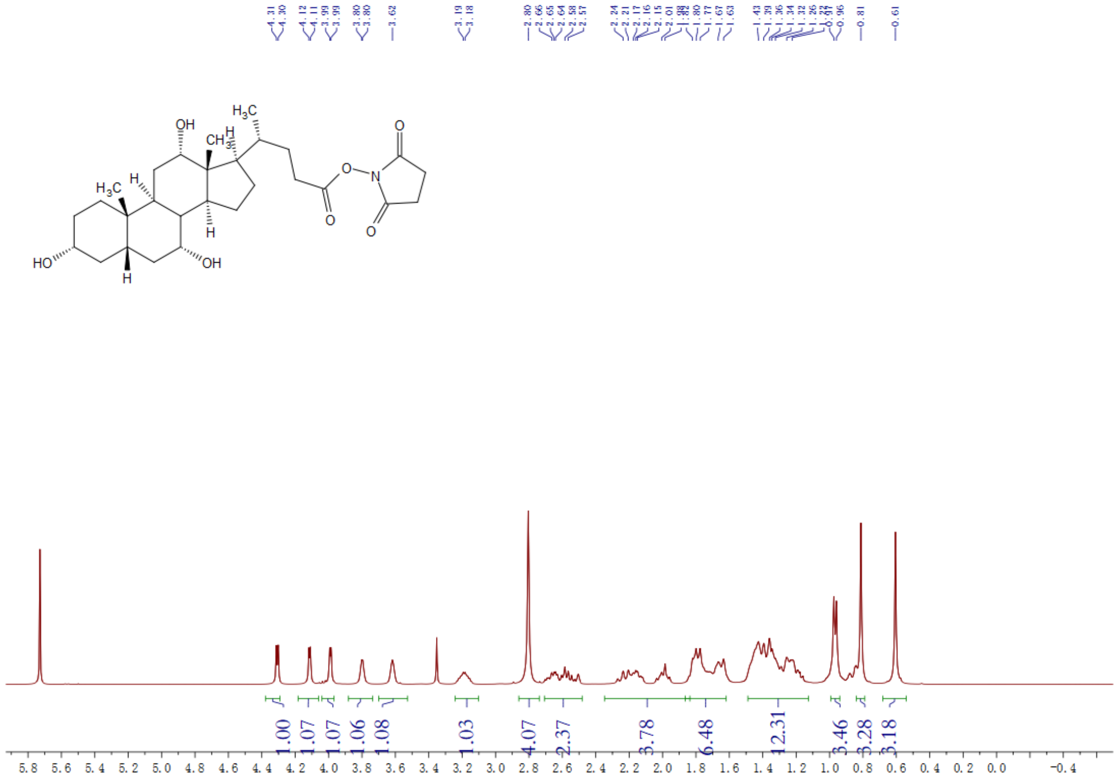

[13]C of BA-1

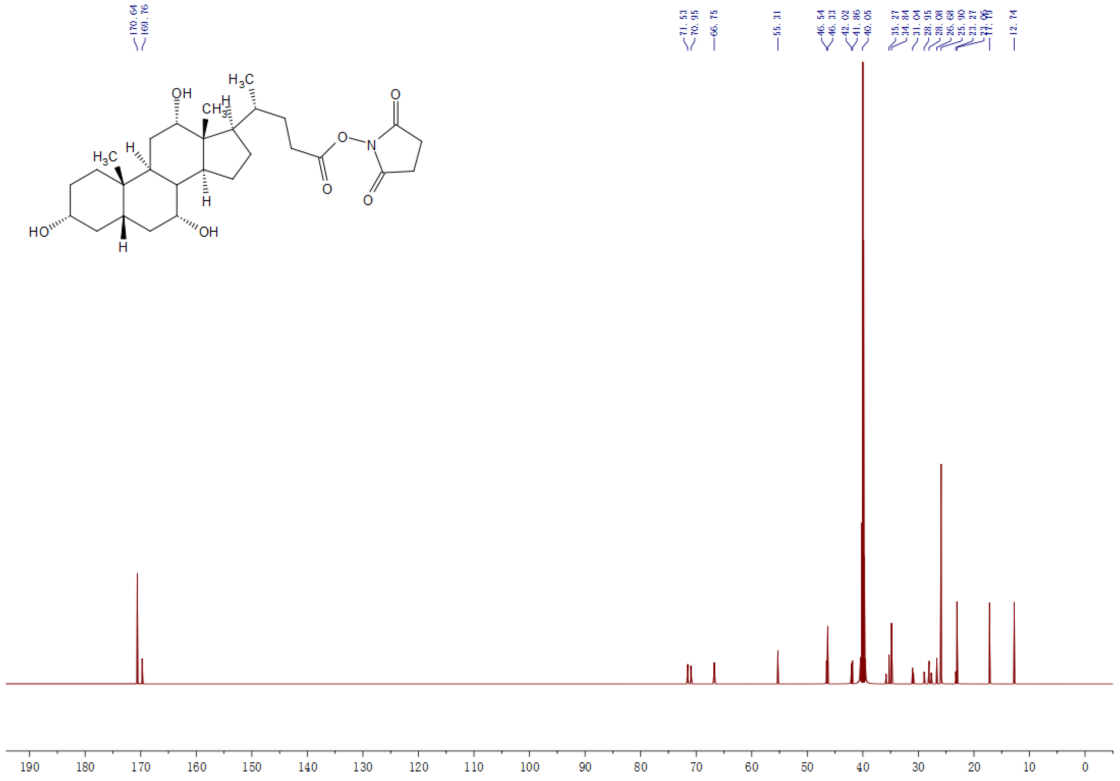

[1]H of CA-1

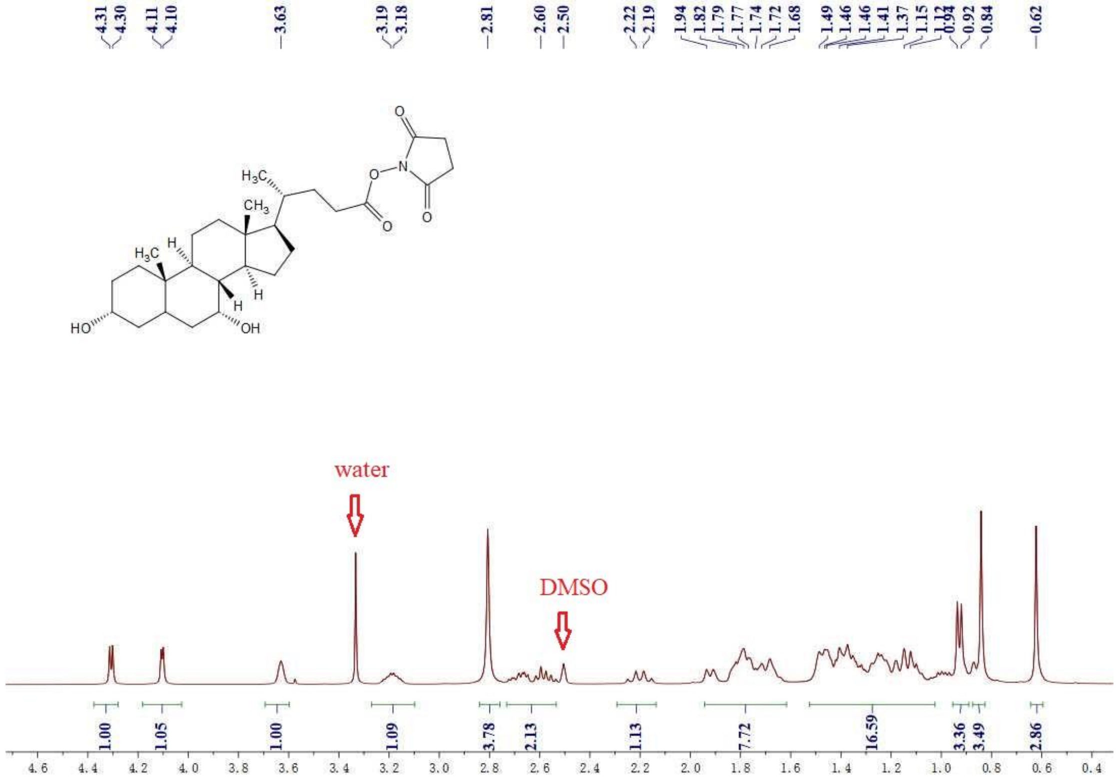

[13]C of CA-1

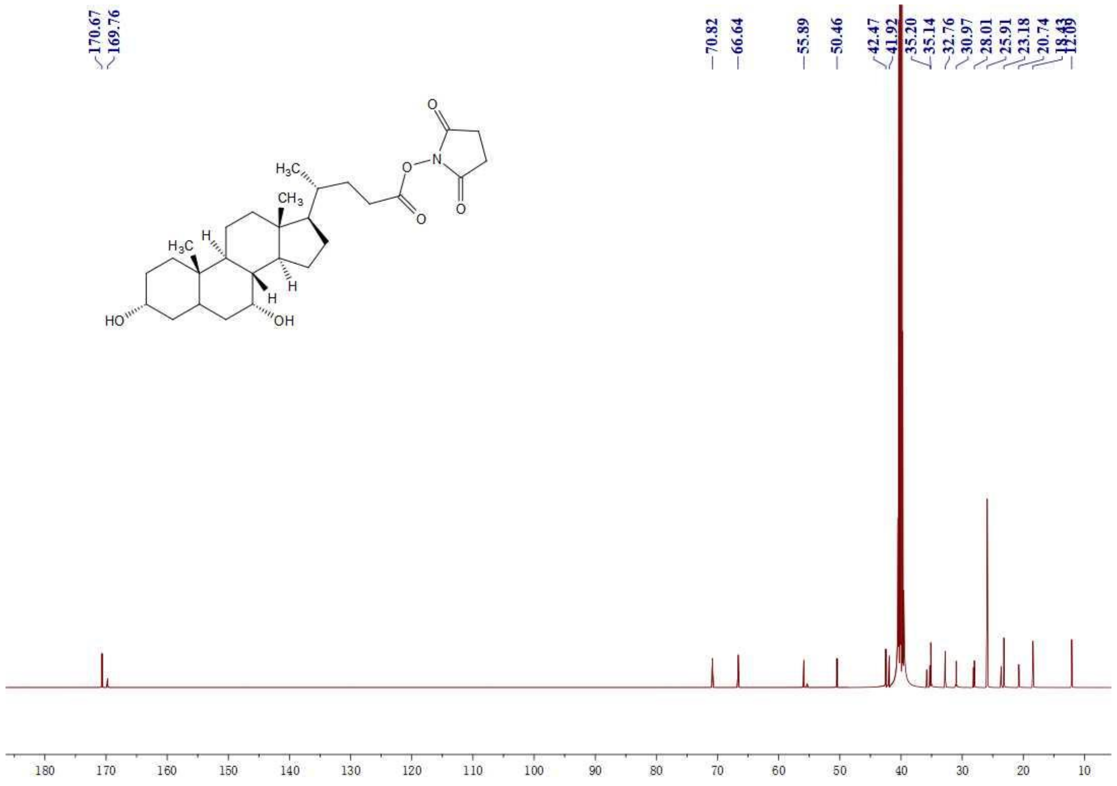

^{1}H of UA-1

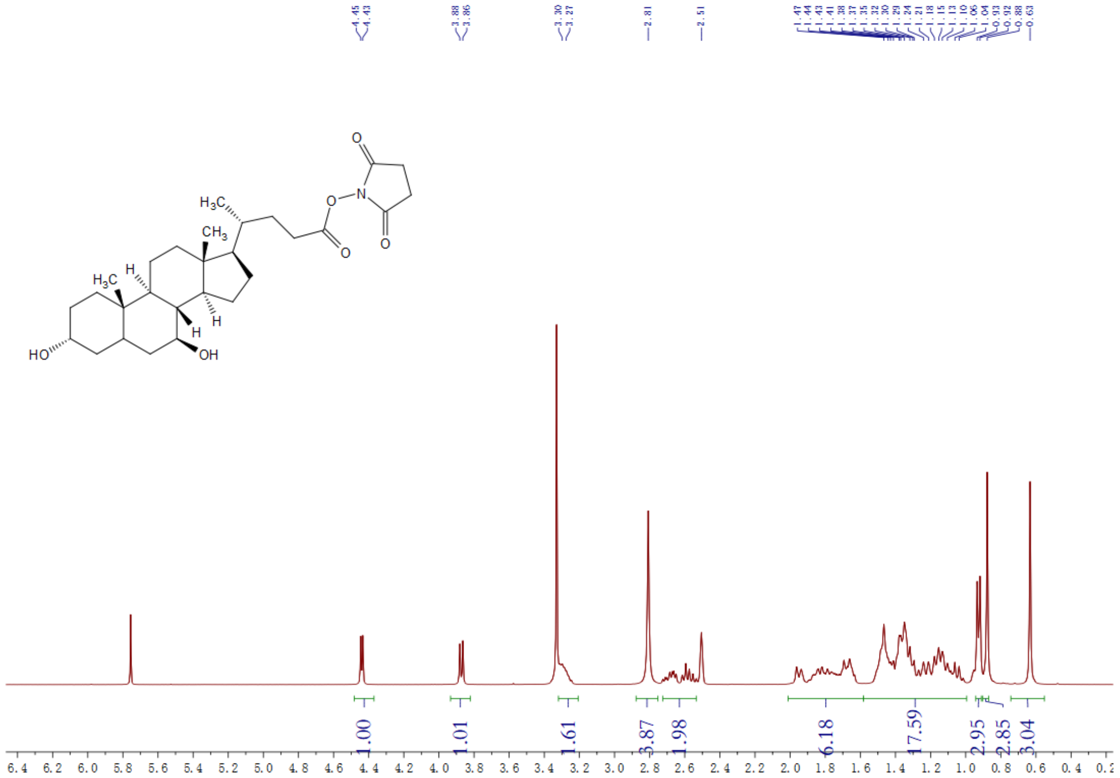

^{13}C of UA-1

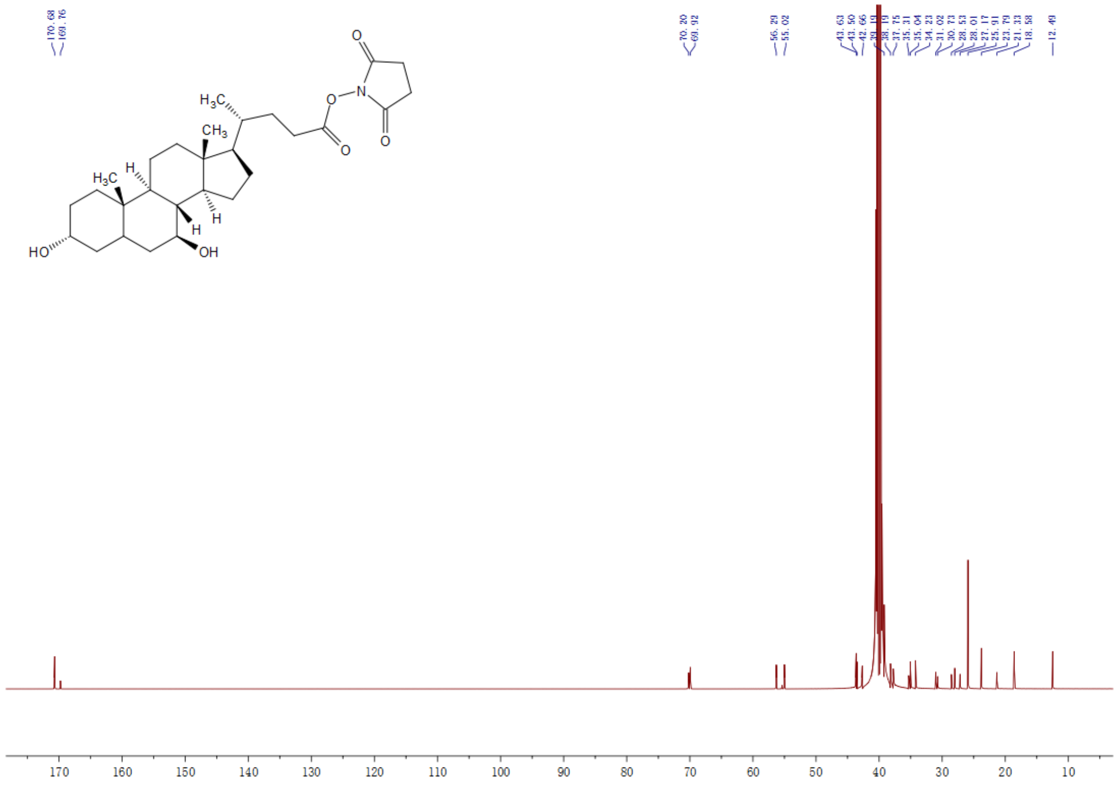

^{1}H of HA-1

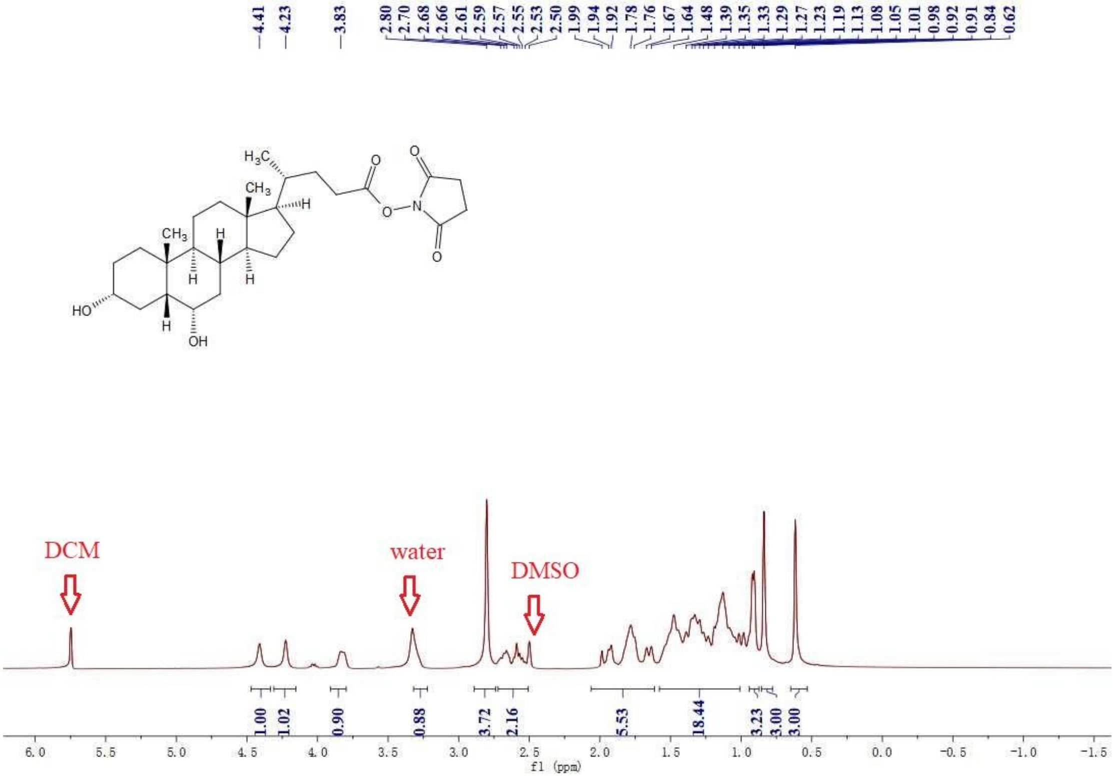

^{13}C of HA-1

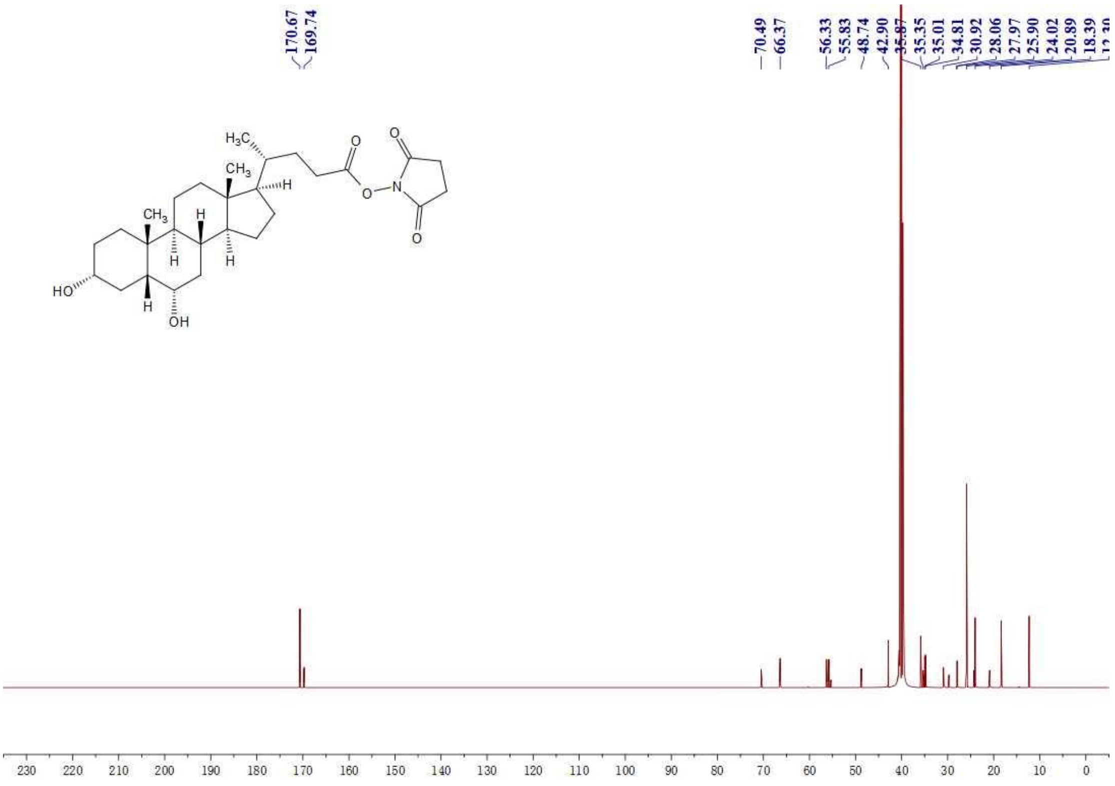

^{1}H of CH-1

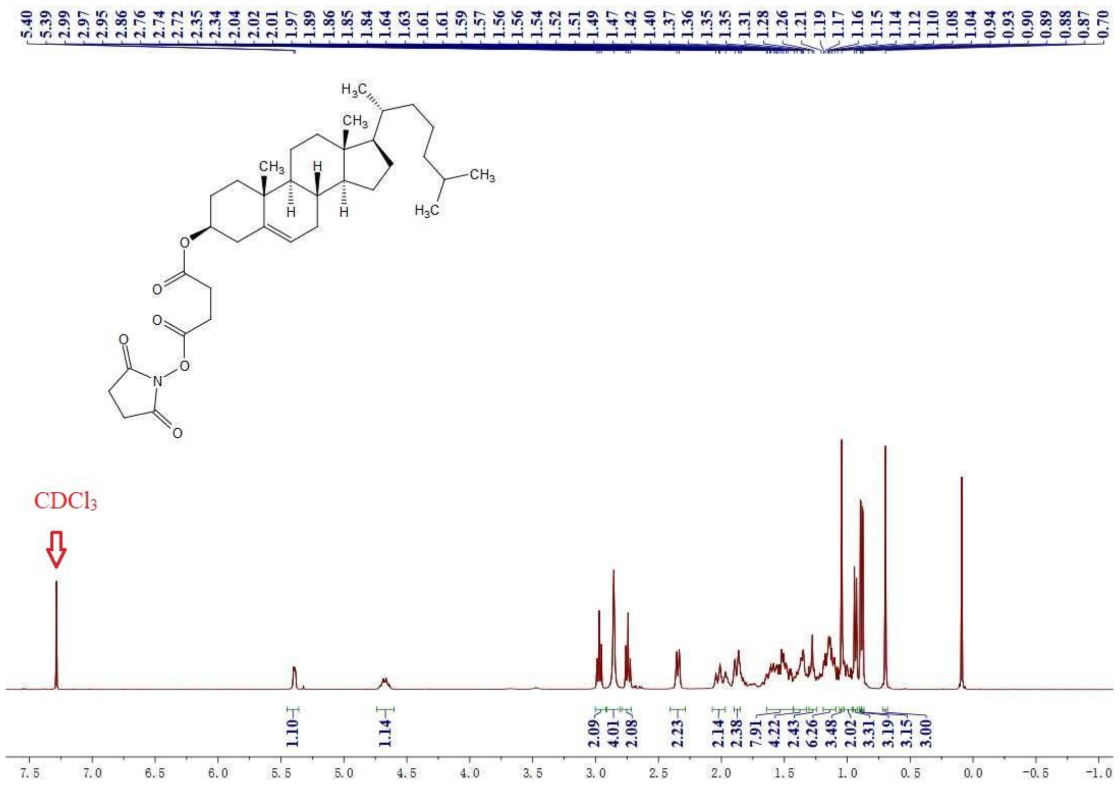

^{13}C of CH-1

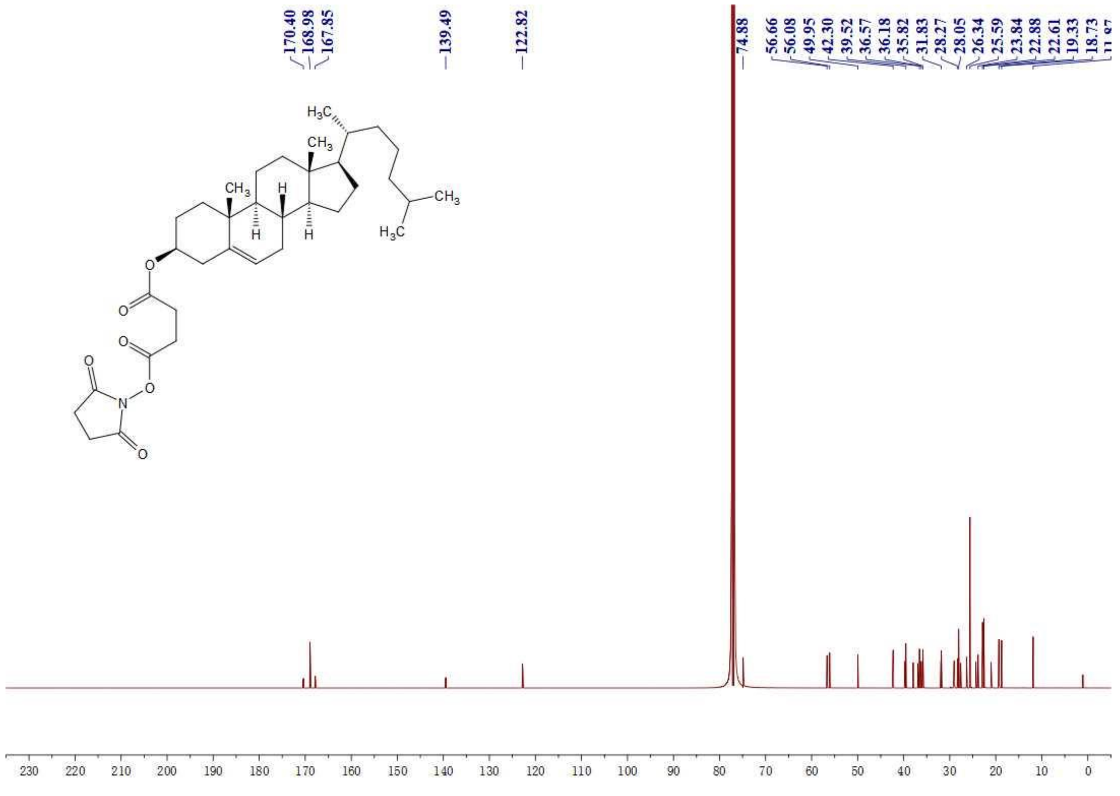

[1]H of GA-1

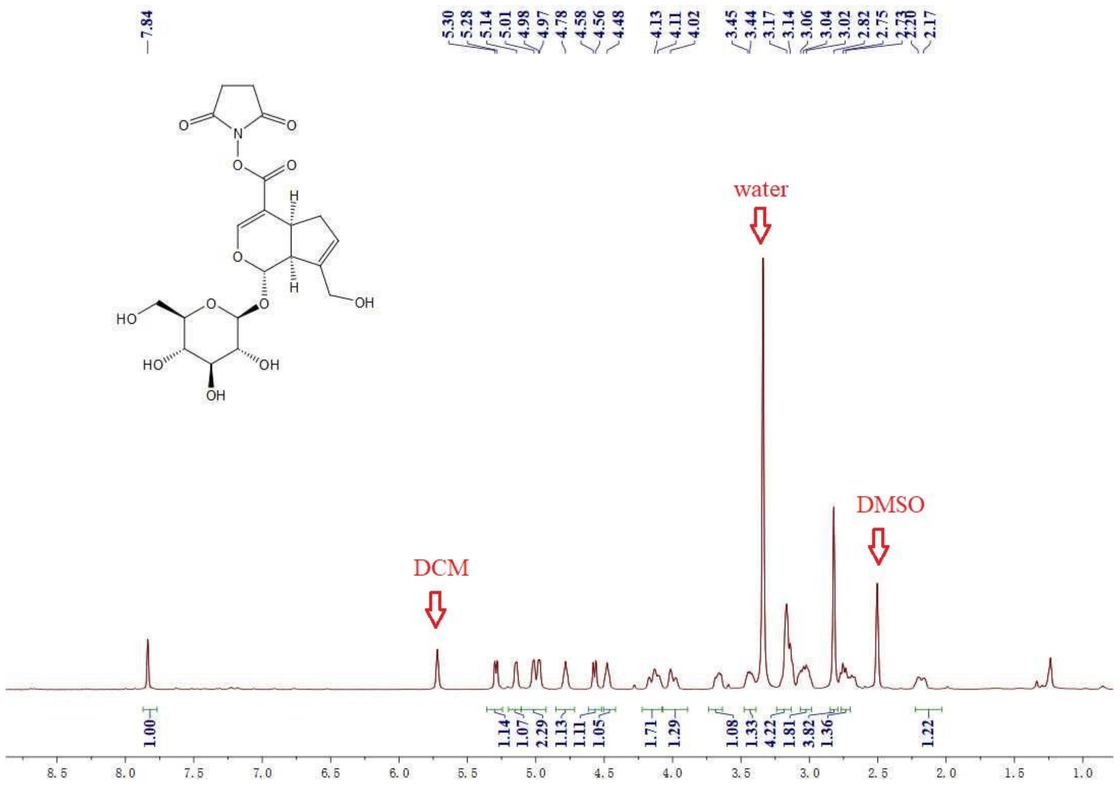

[13]C of GA-1

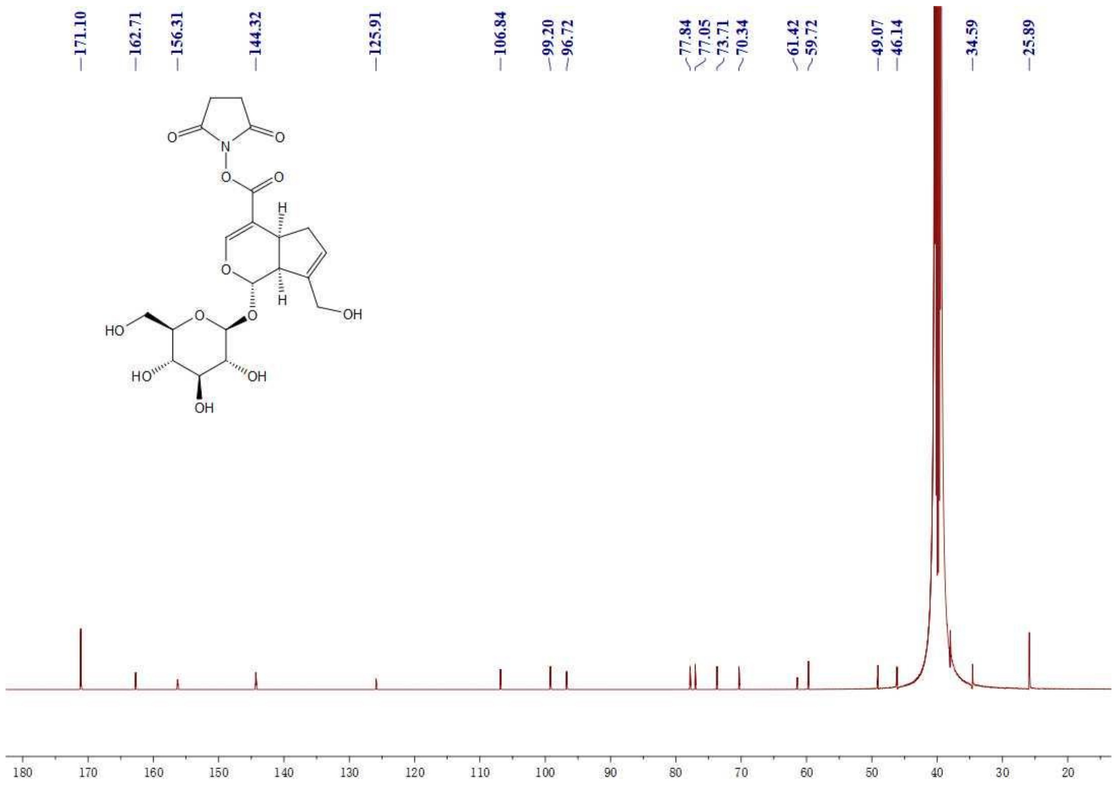

^{1}H of Ar-1

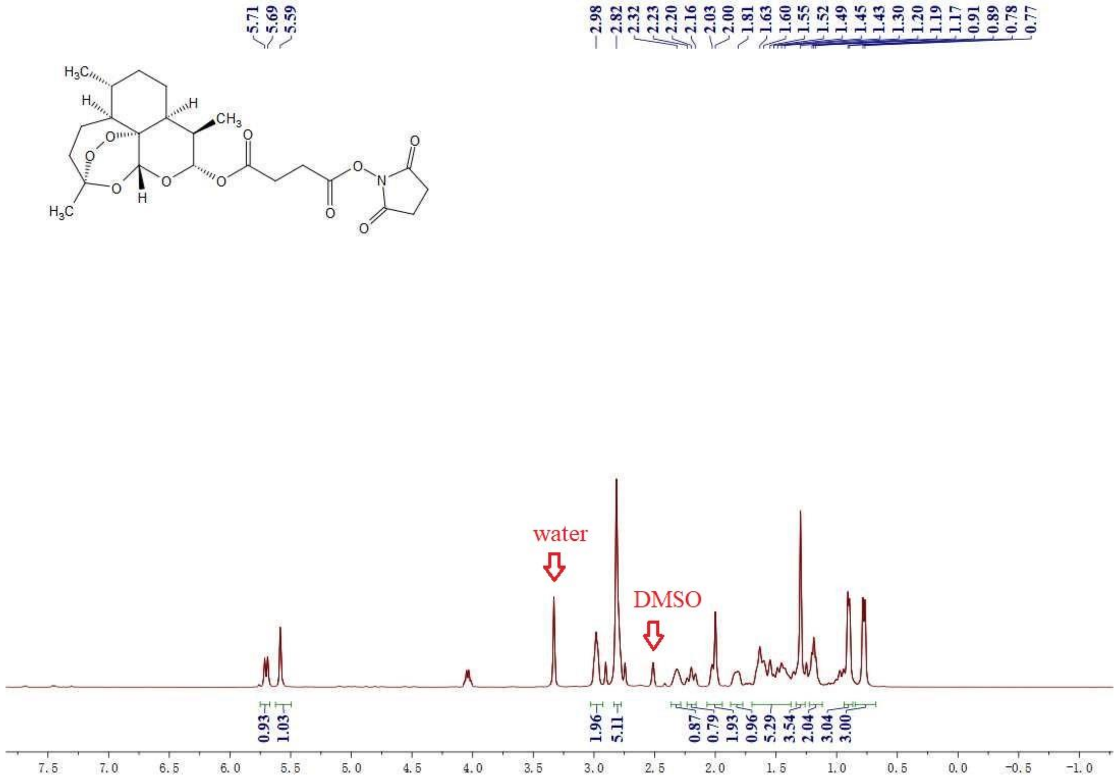

^{13}C of Ar-1

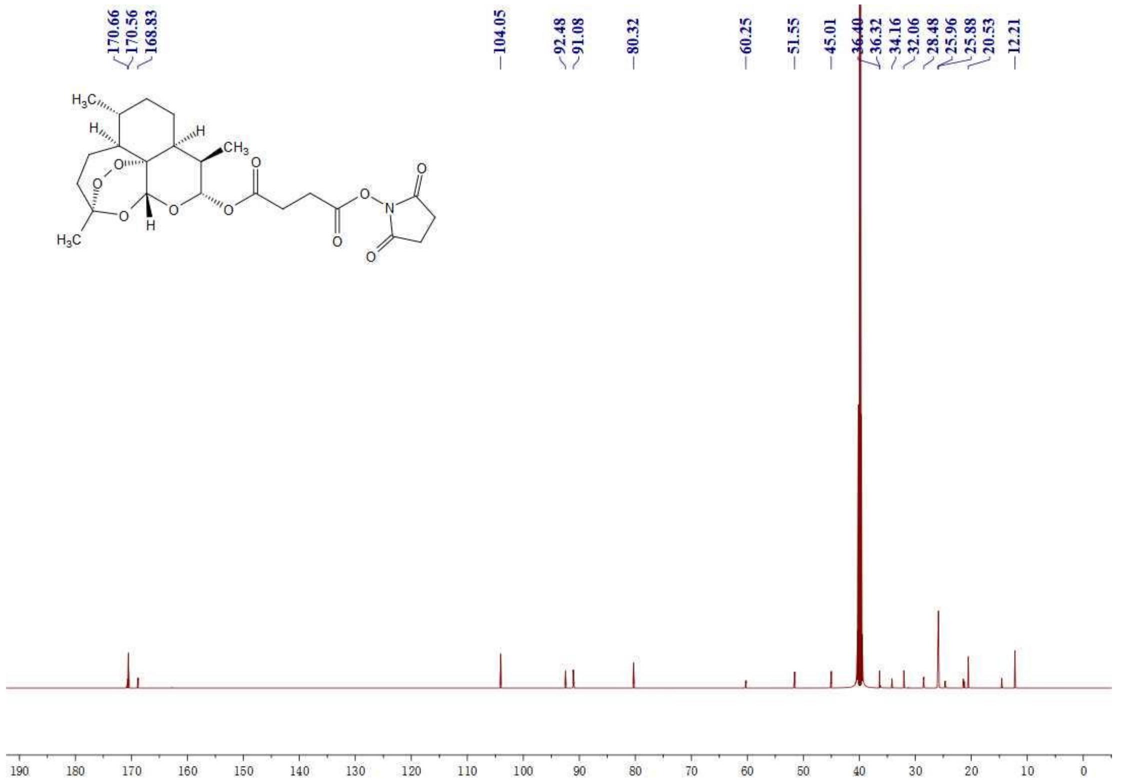

^{1}H of MA-1

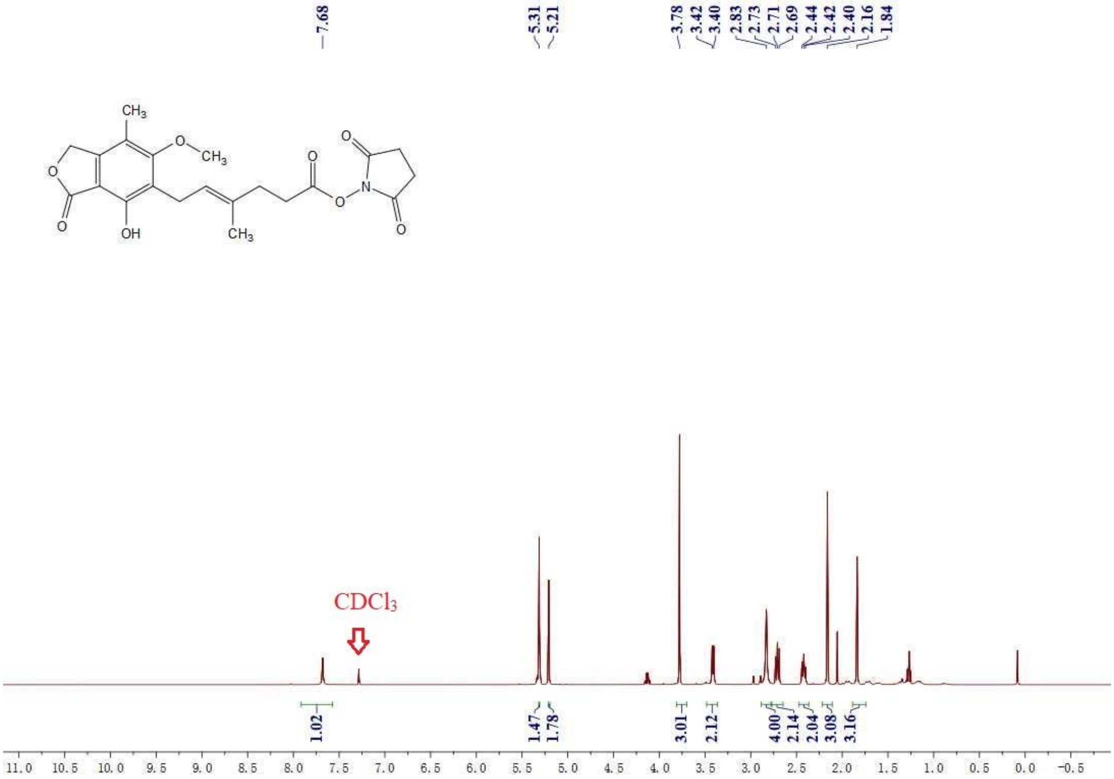

^{13}C of MA-1

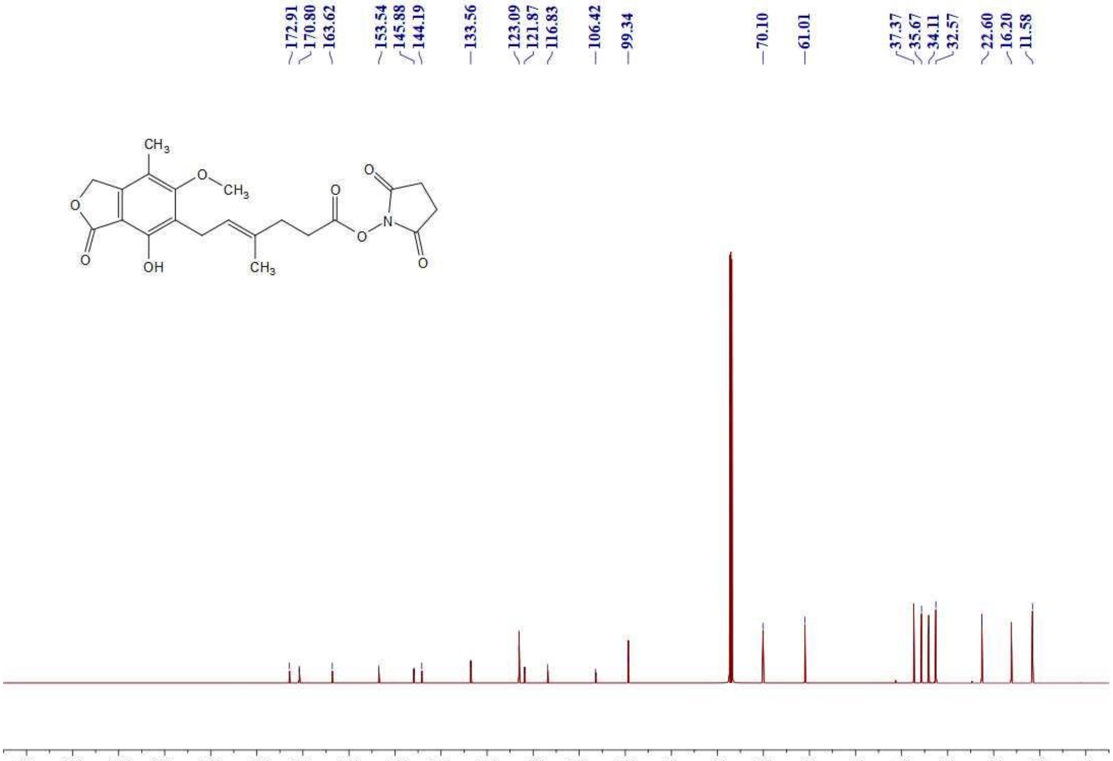

[1]H of UrA-1

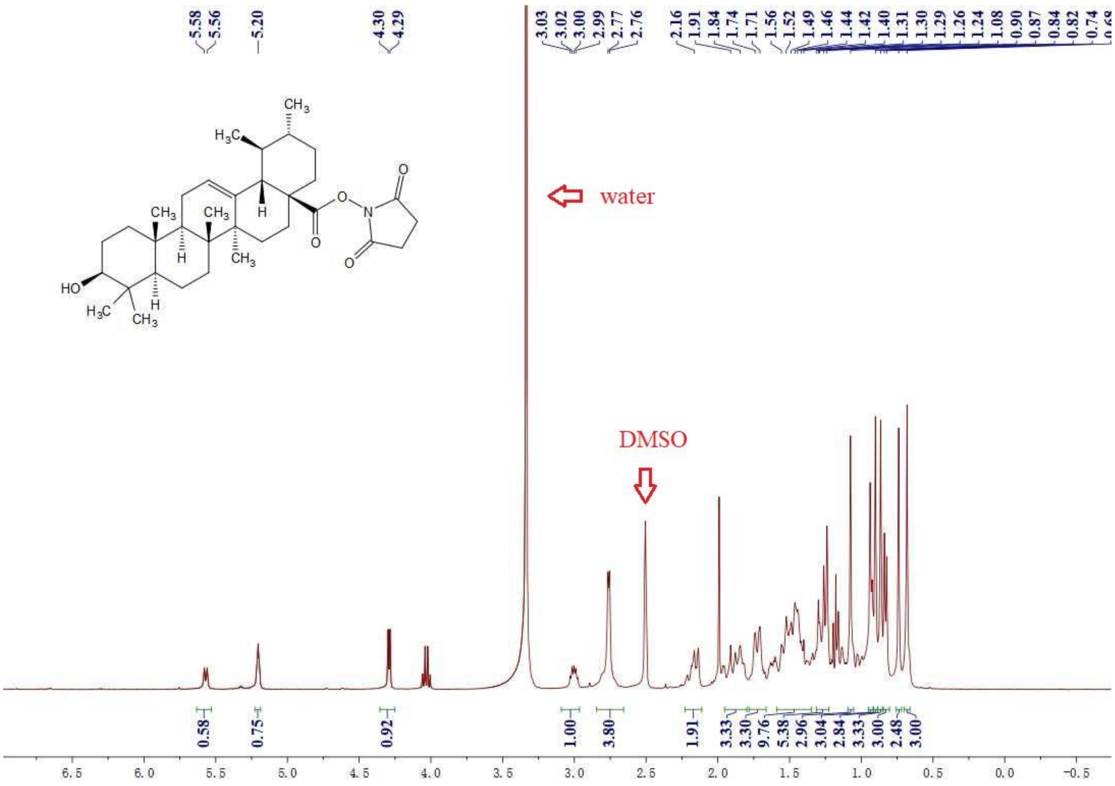

[13]C of UrA-1

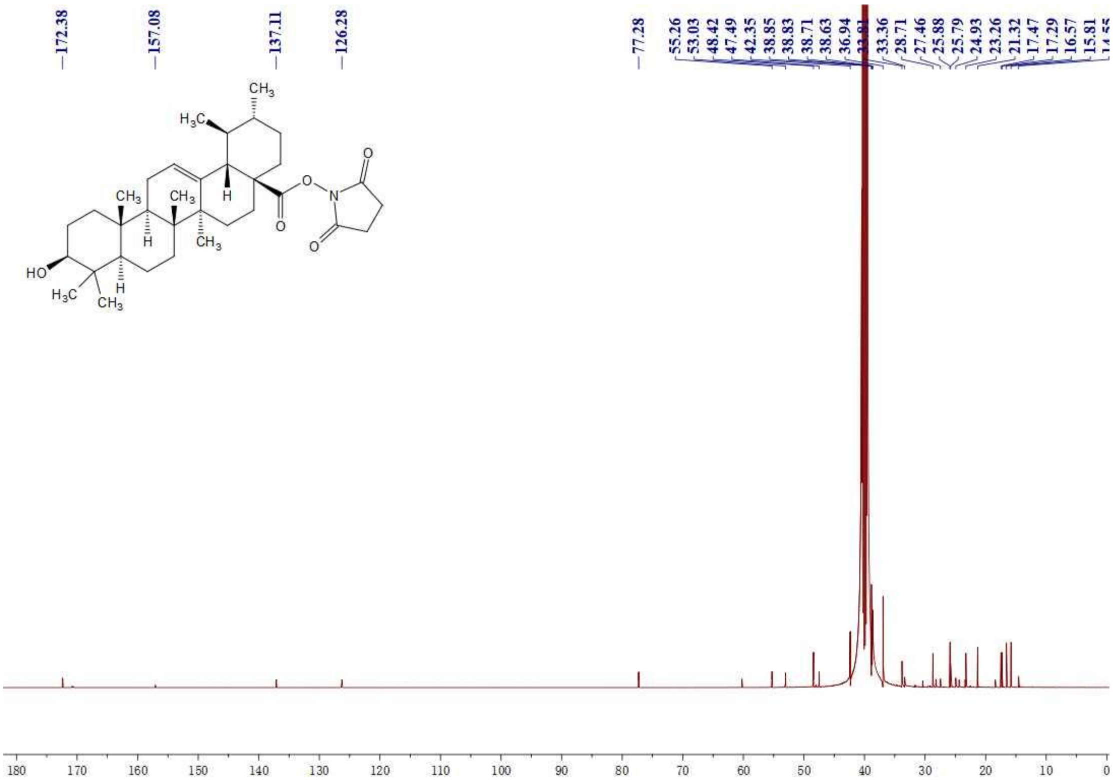

^{1}H of BA-2

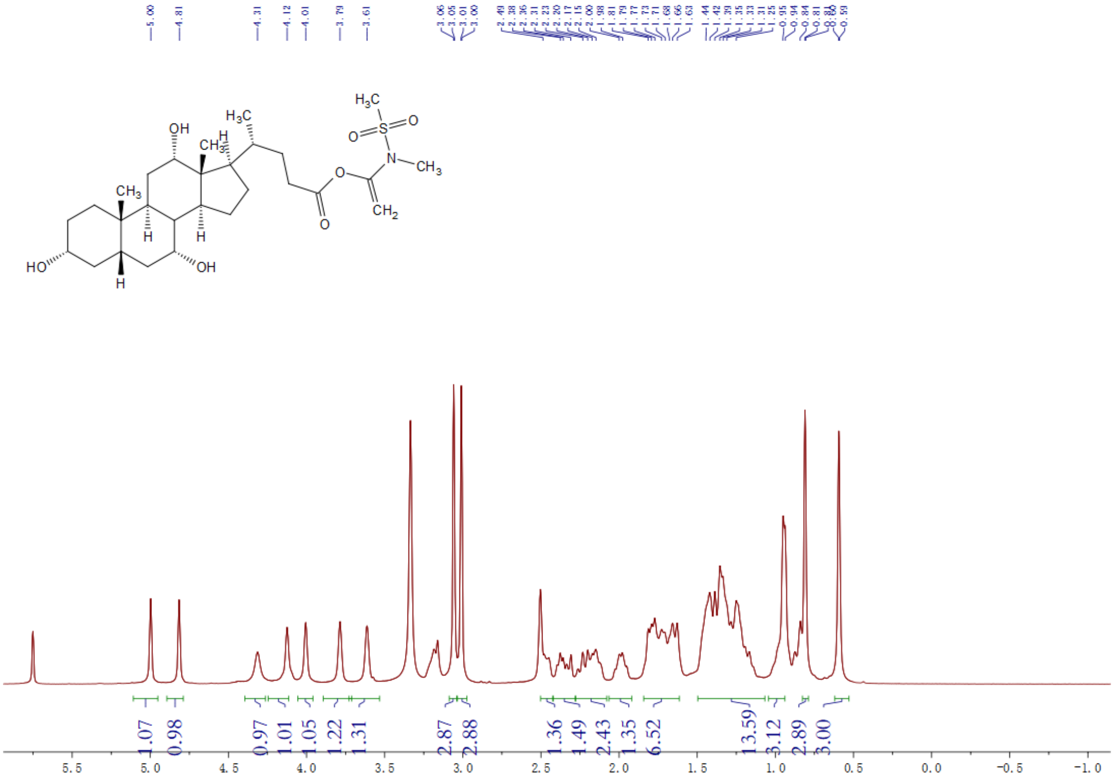

^{13}C of BA-2

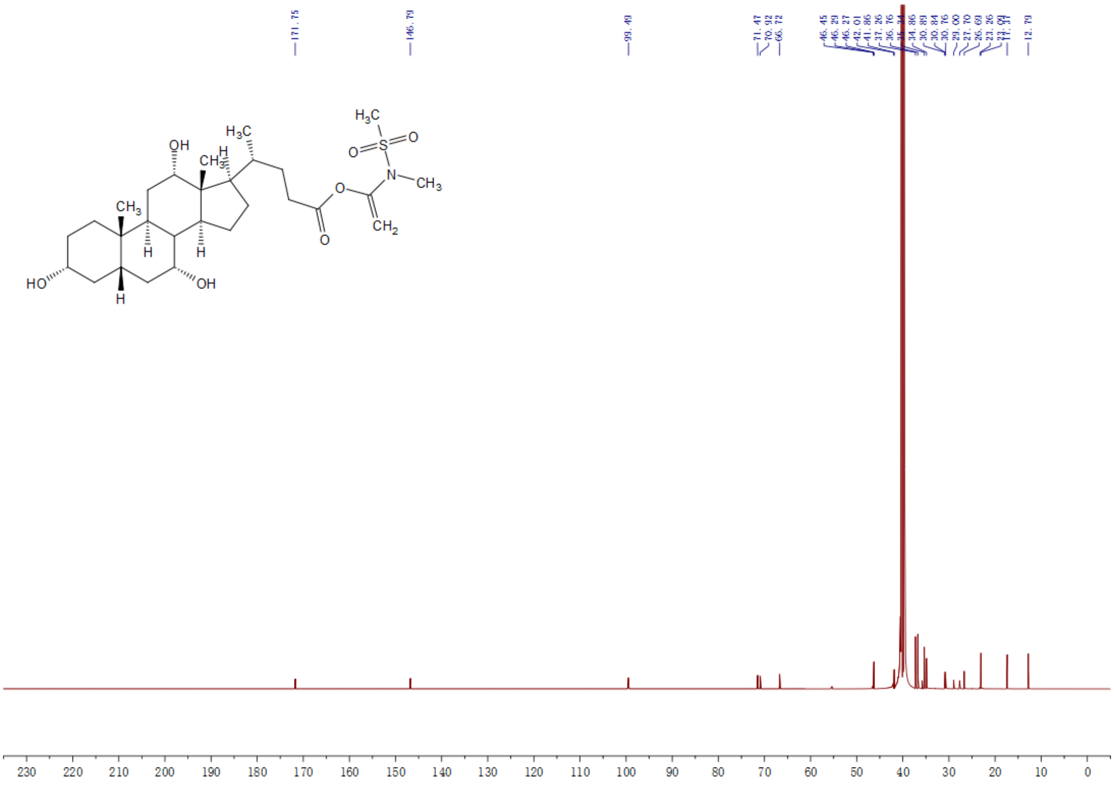

^{1}H of CA-2

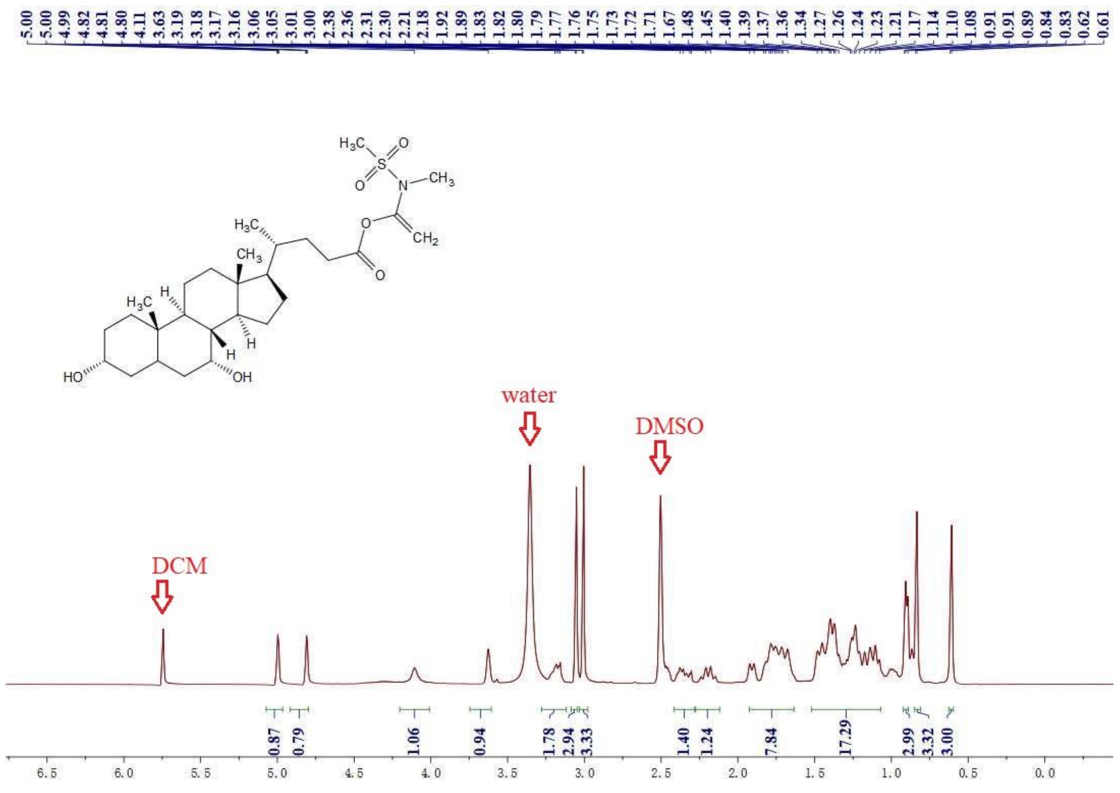

^{13}C of CA-2

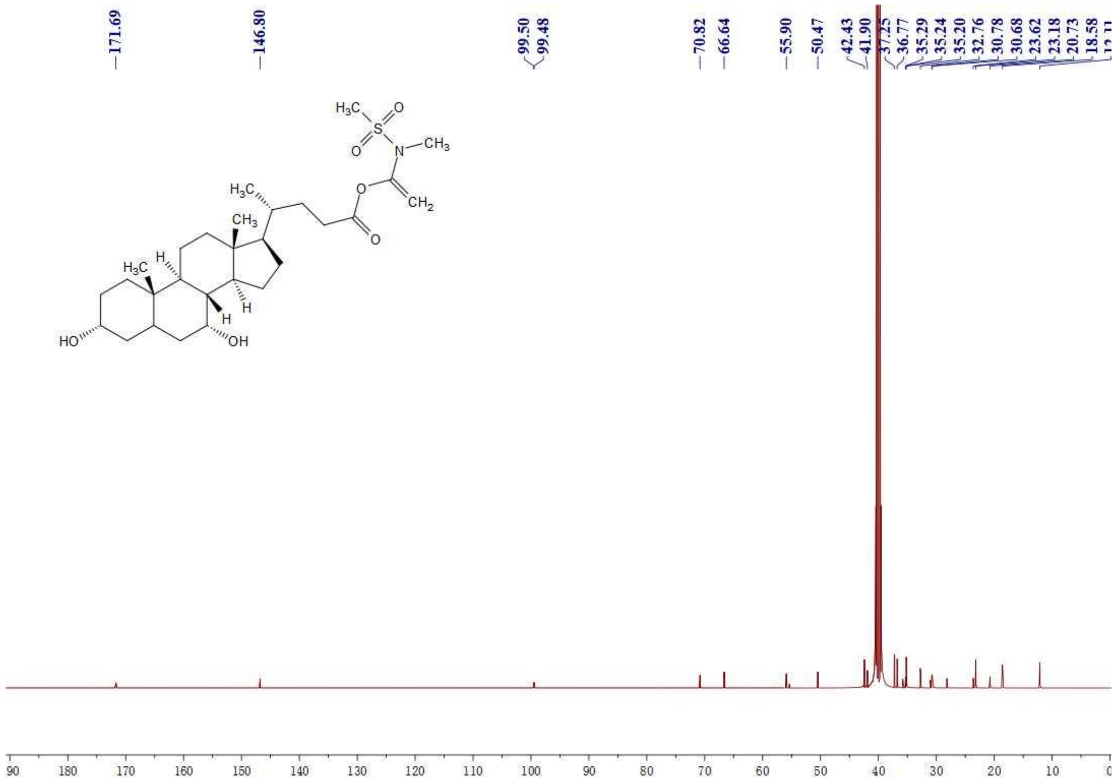

^{1}H of UA-2

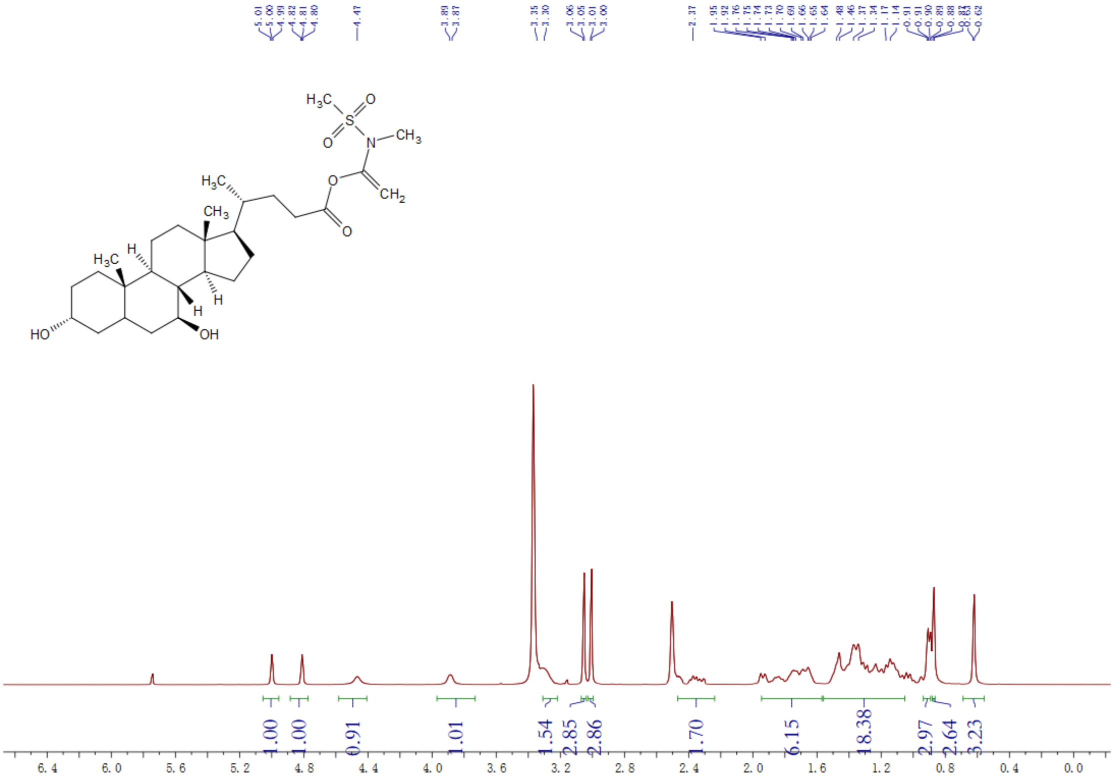

^{13}C of UA-2

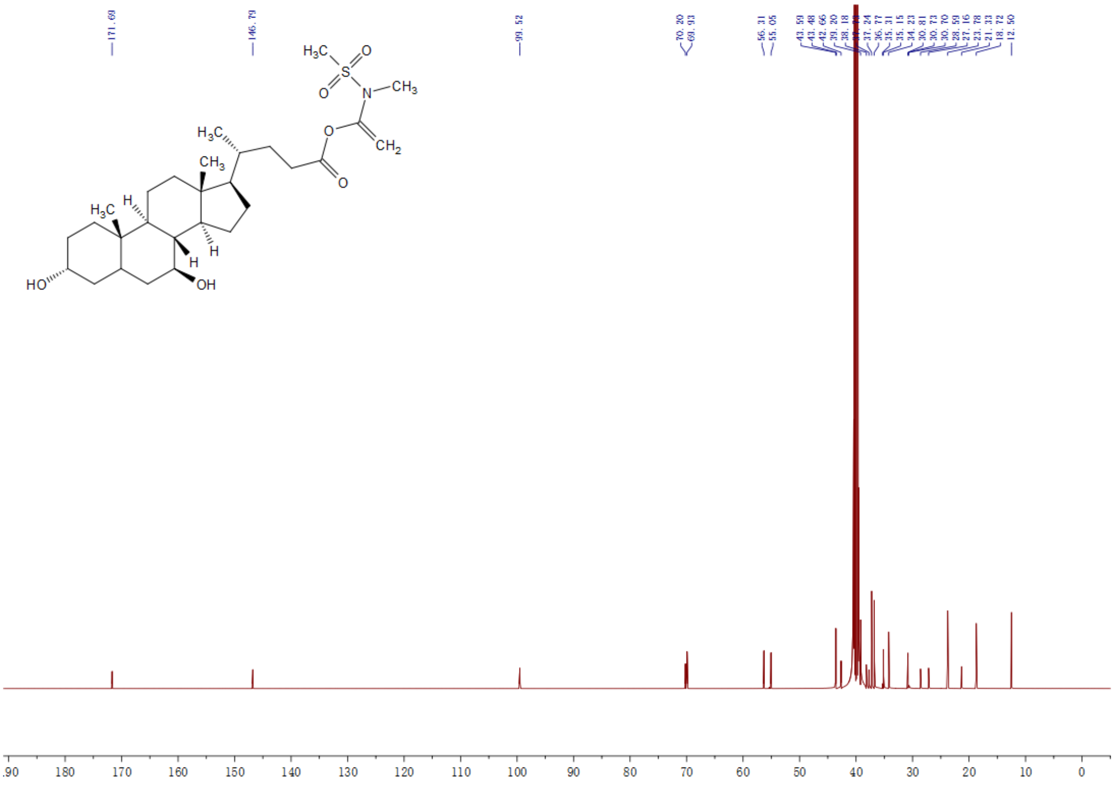

[1]H of HA-2

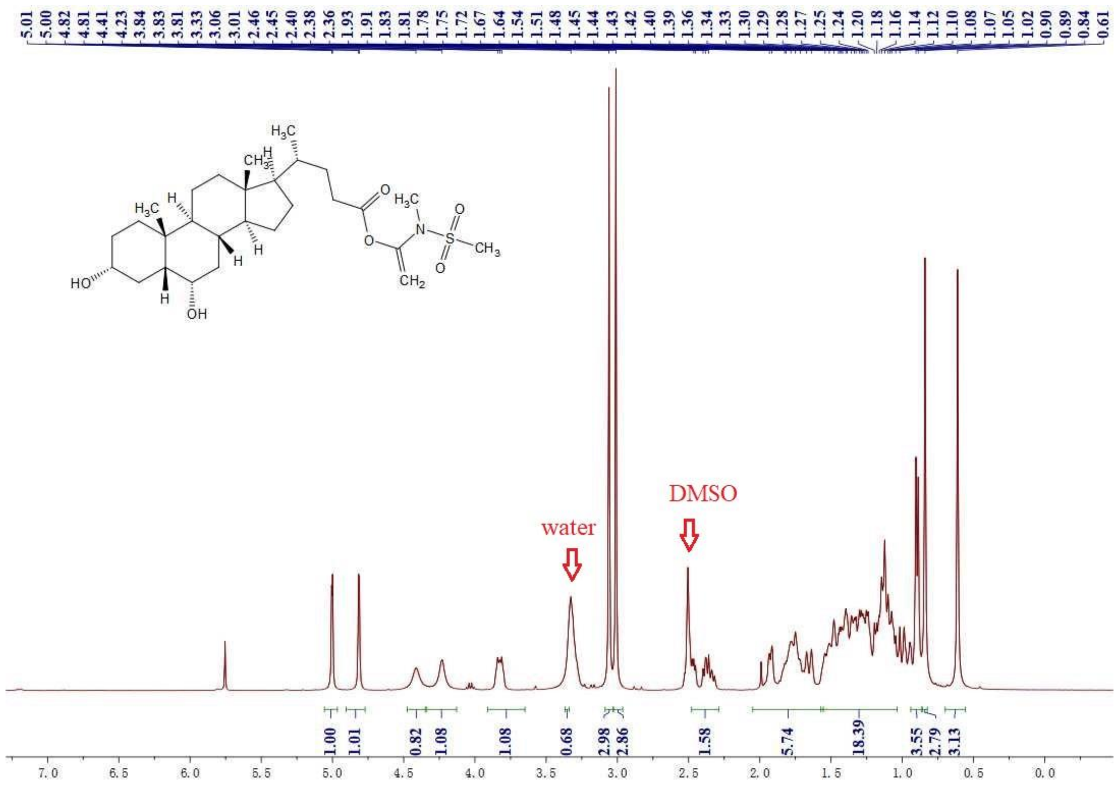

[13]C of HA-2

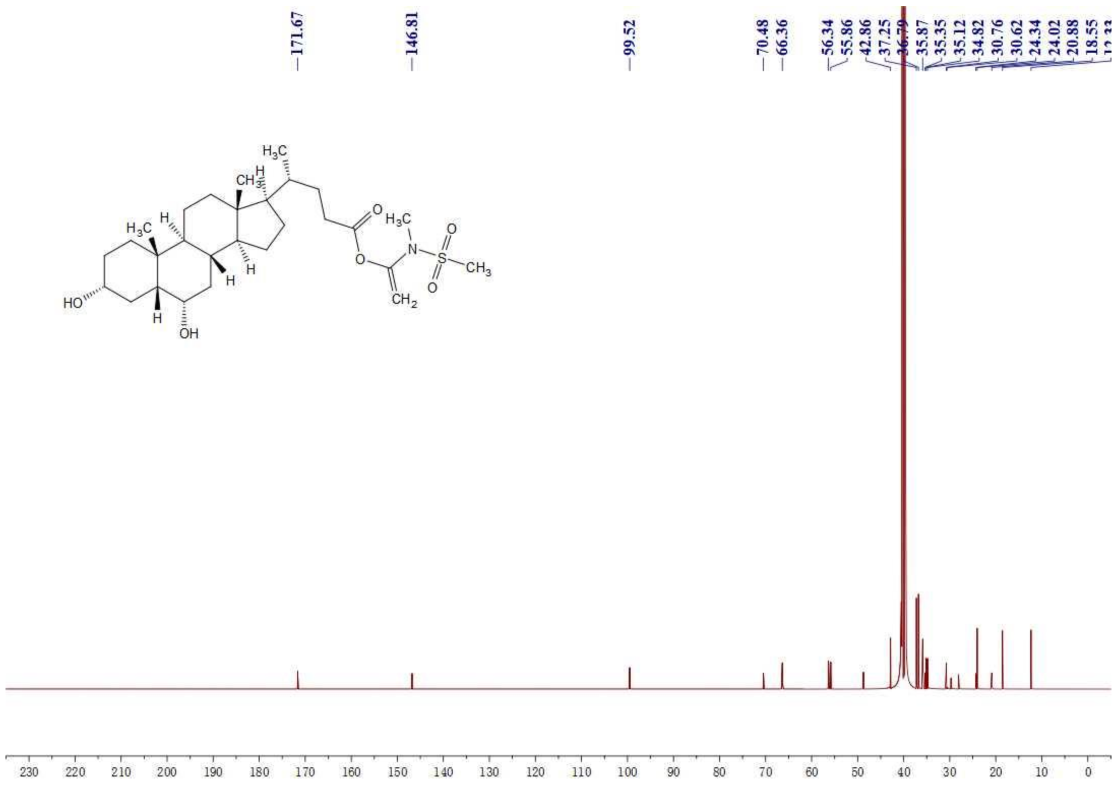

^{1}H of CH-2

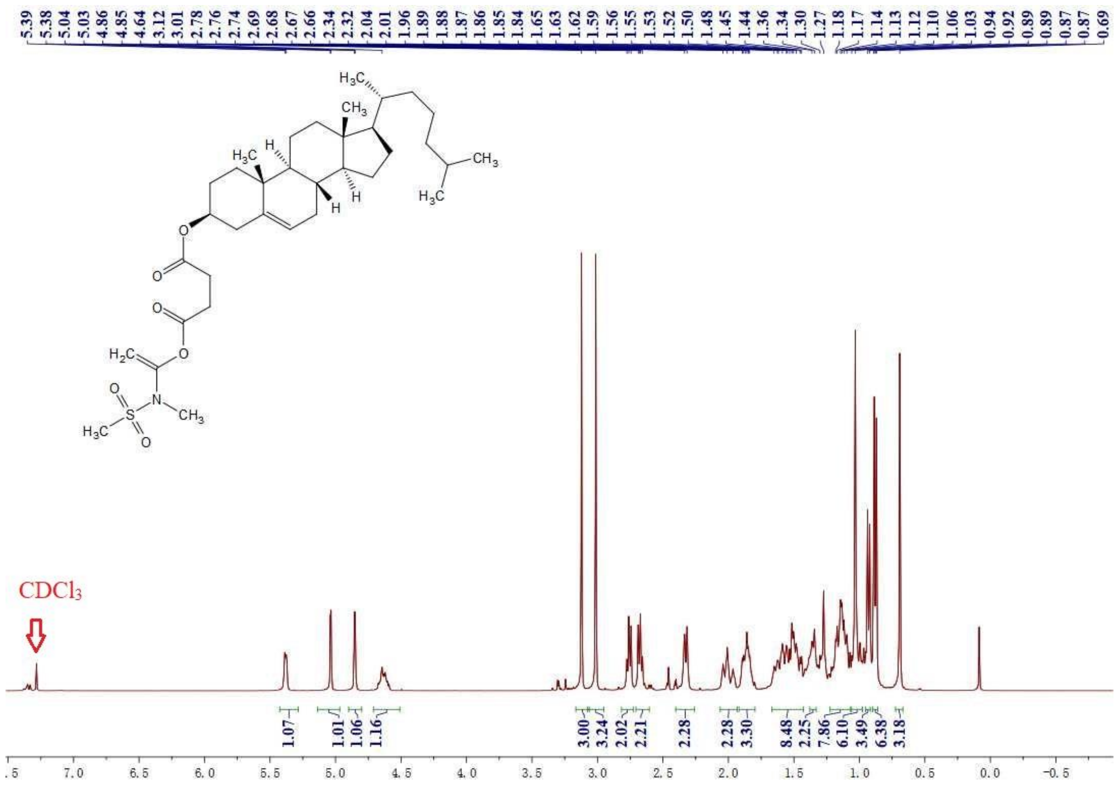

^{13}C of CH-2

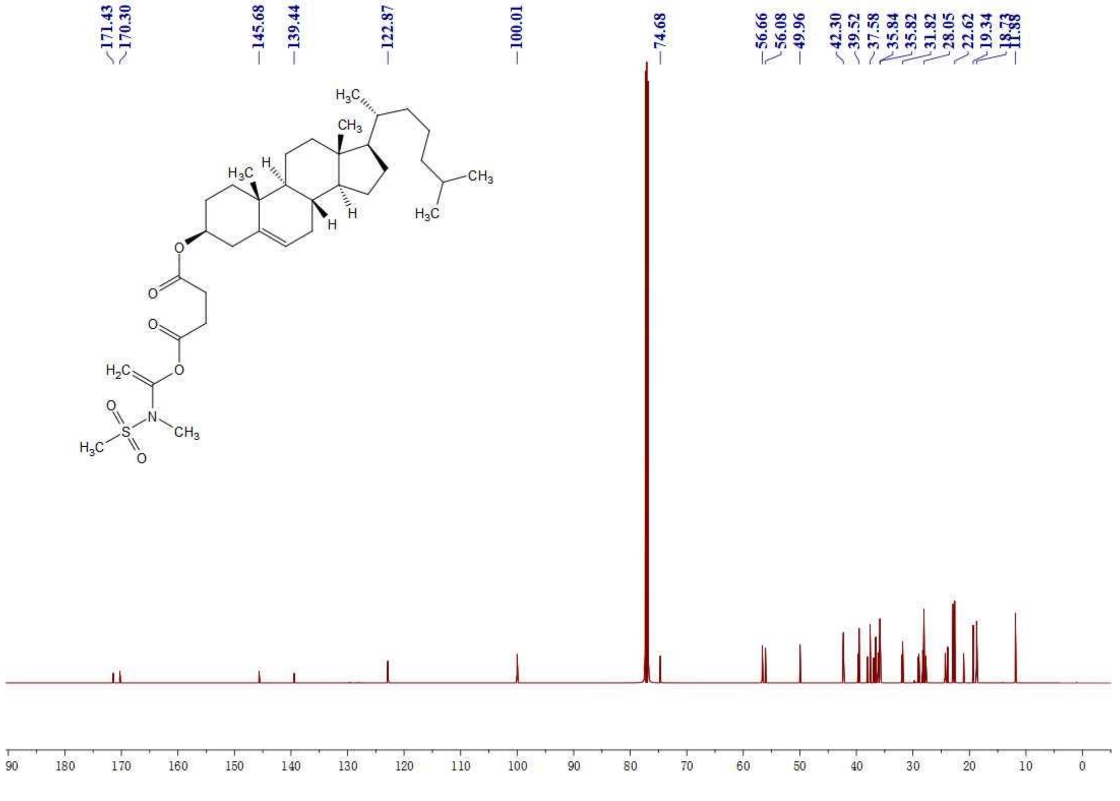

[1]H of GA-2

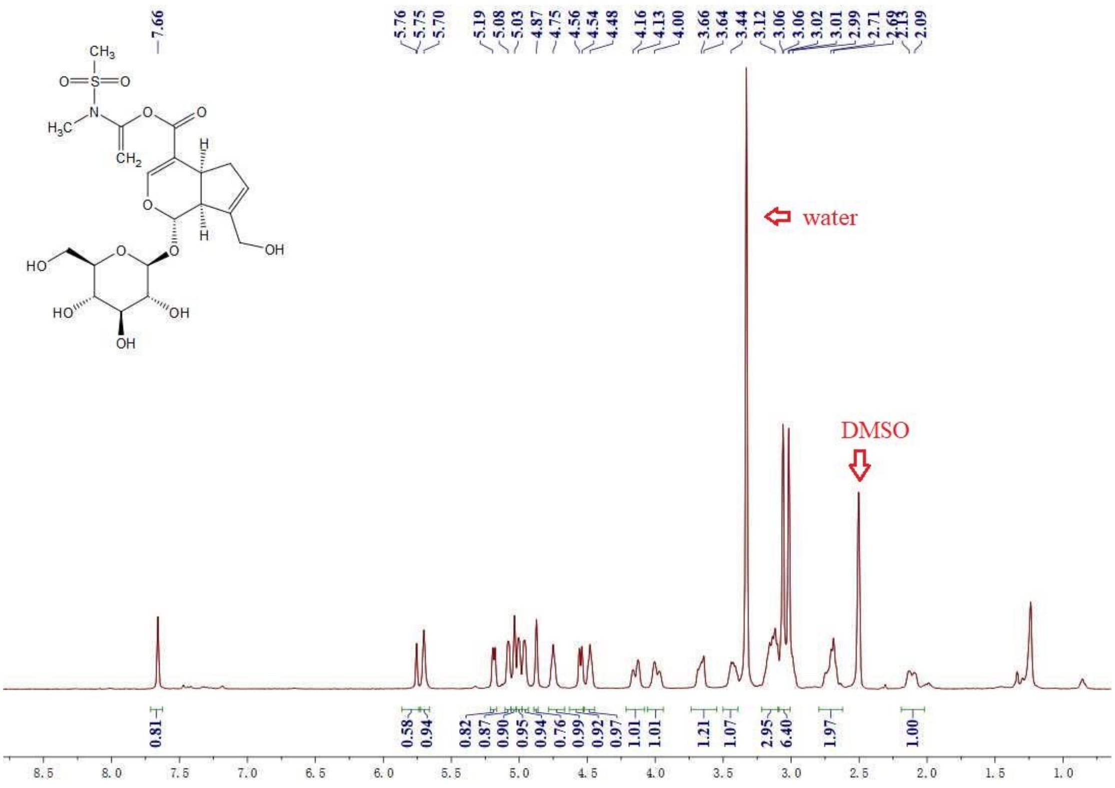

[13]C of GA-2

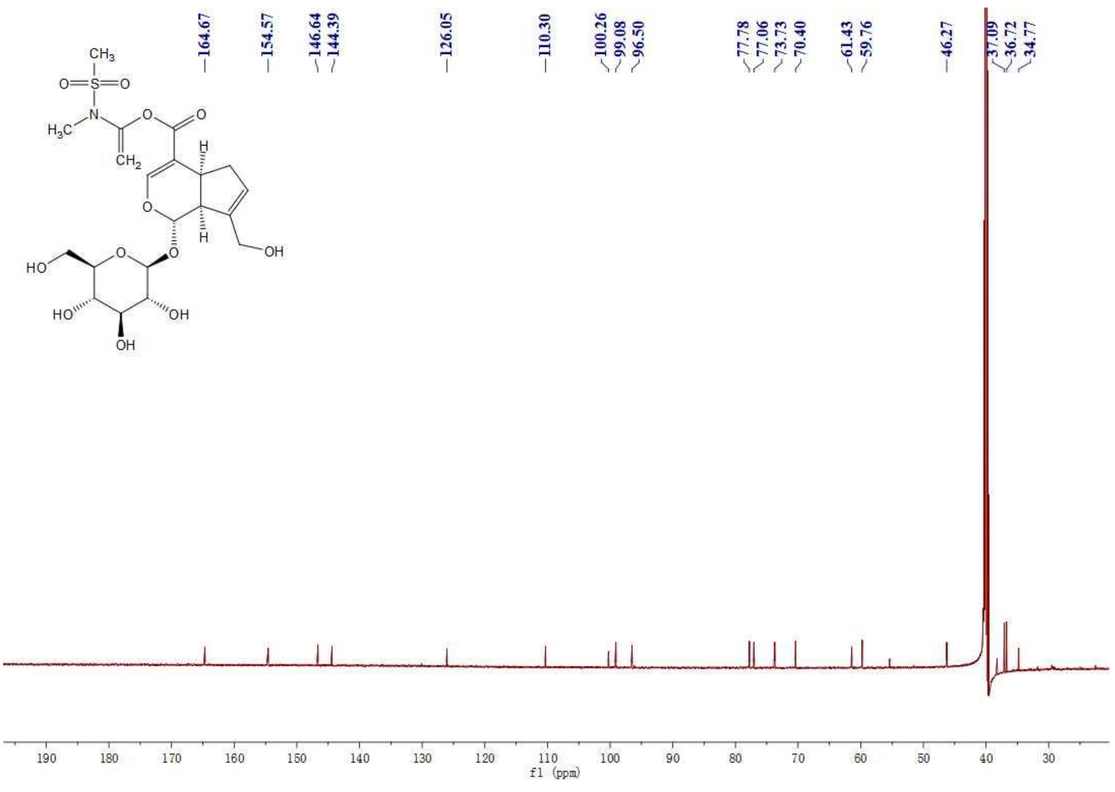

[1]H of Ar-2

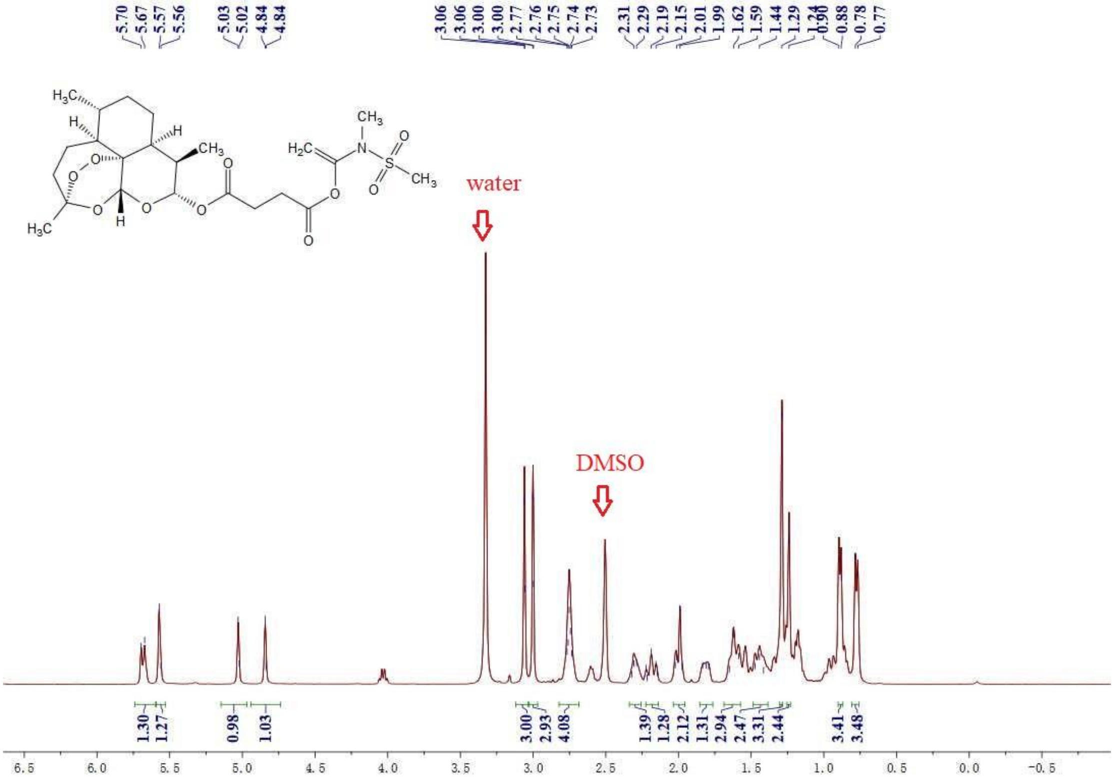

[13]C of Ar-2

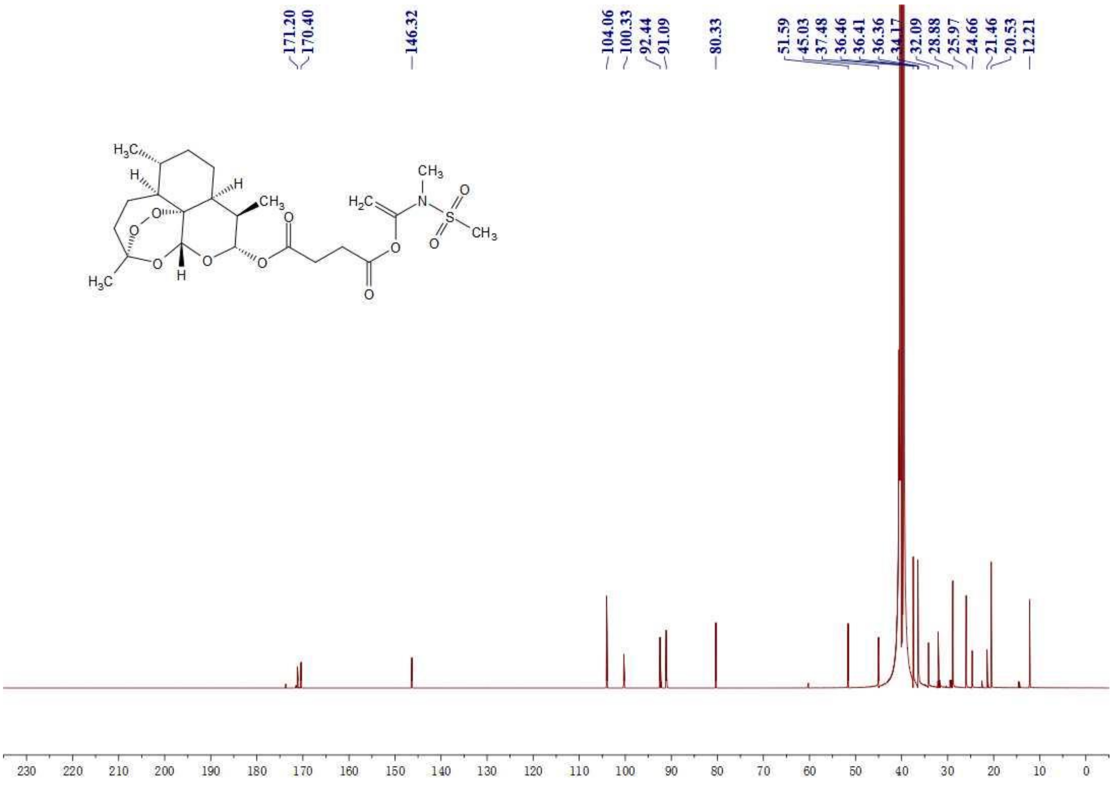

^{1}H of MA-2

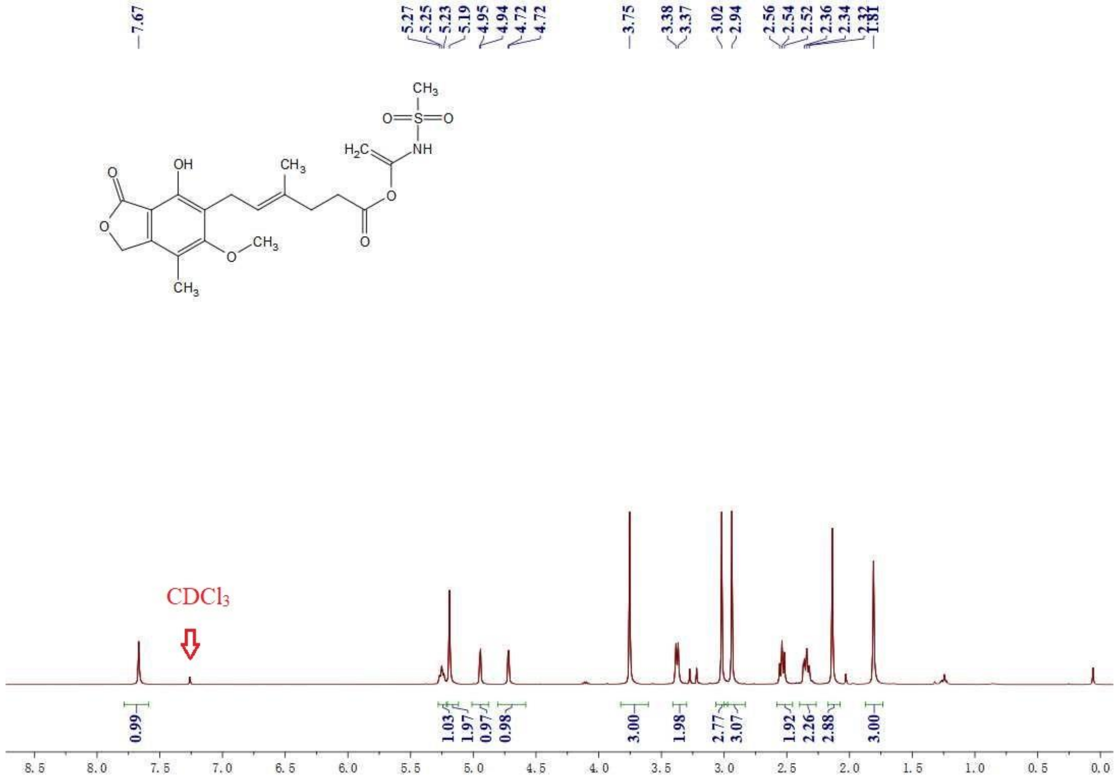

^{13}C of MA-2

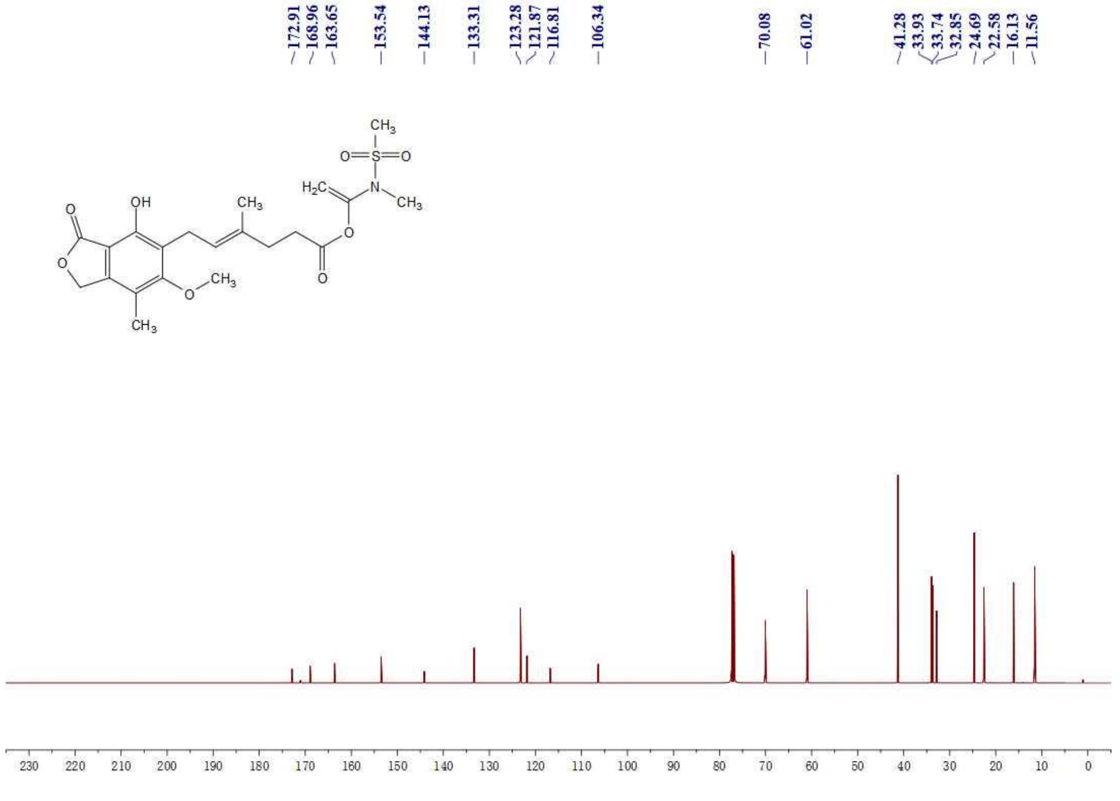

^{1}H of UrA-2

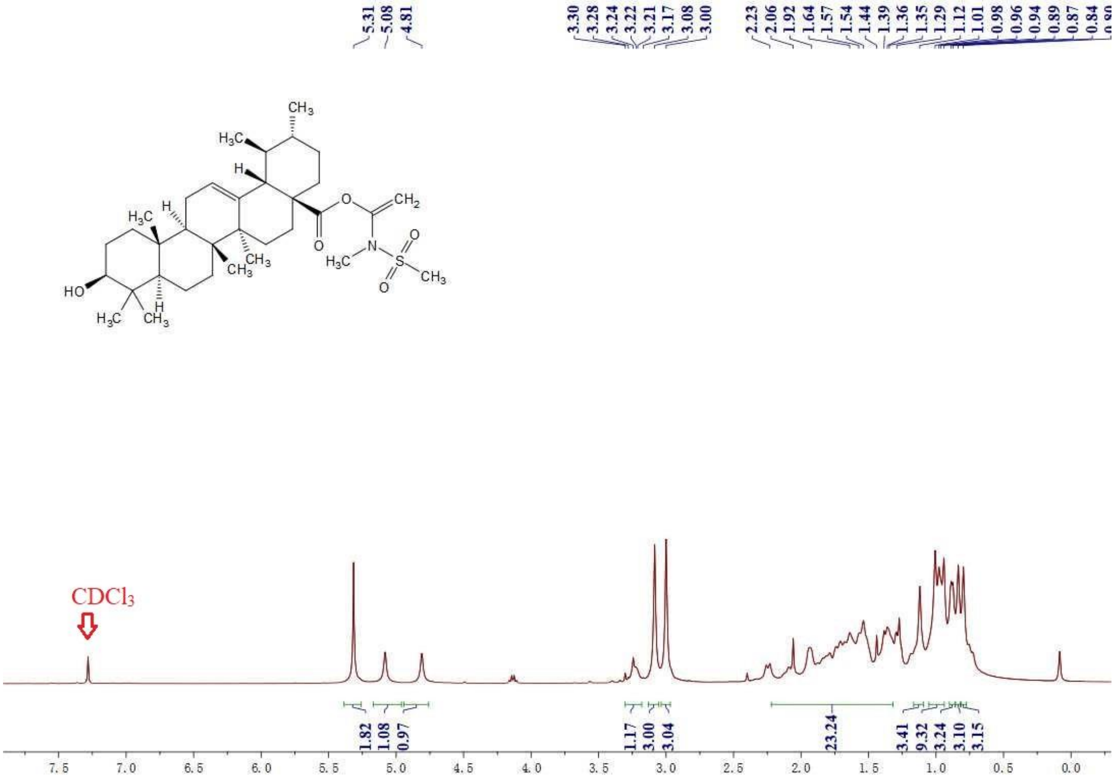

^{13}C of UrA-2

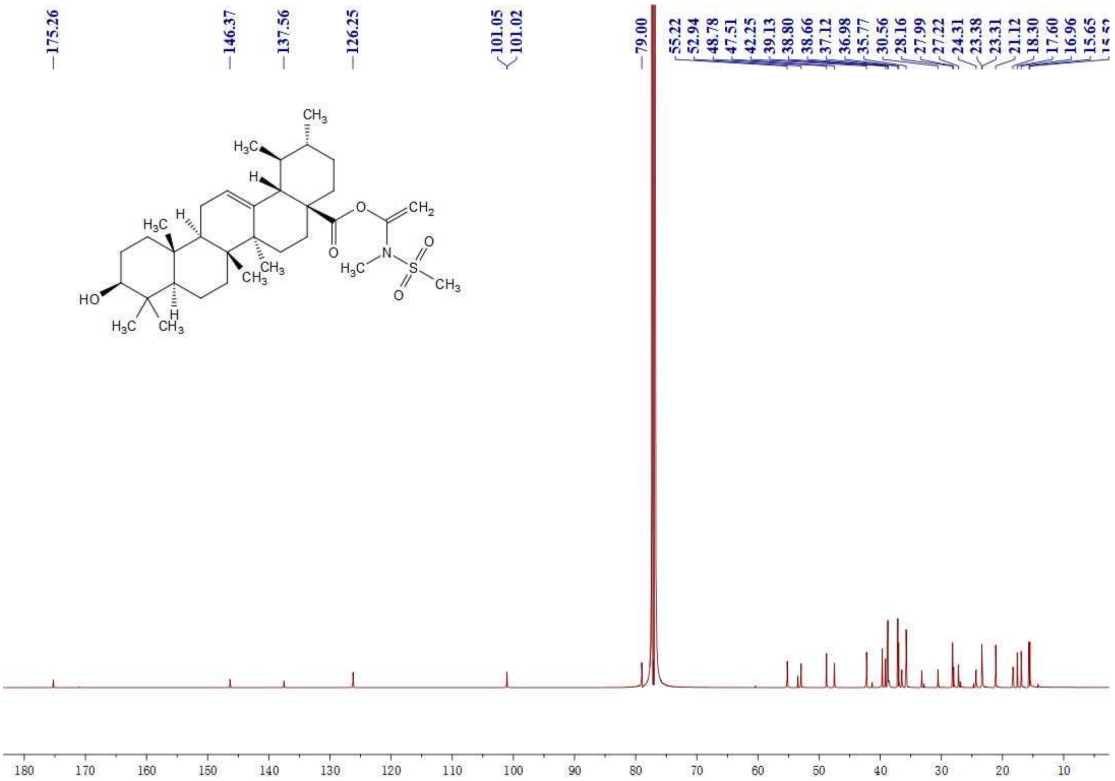

^{1}H of BA-3

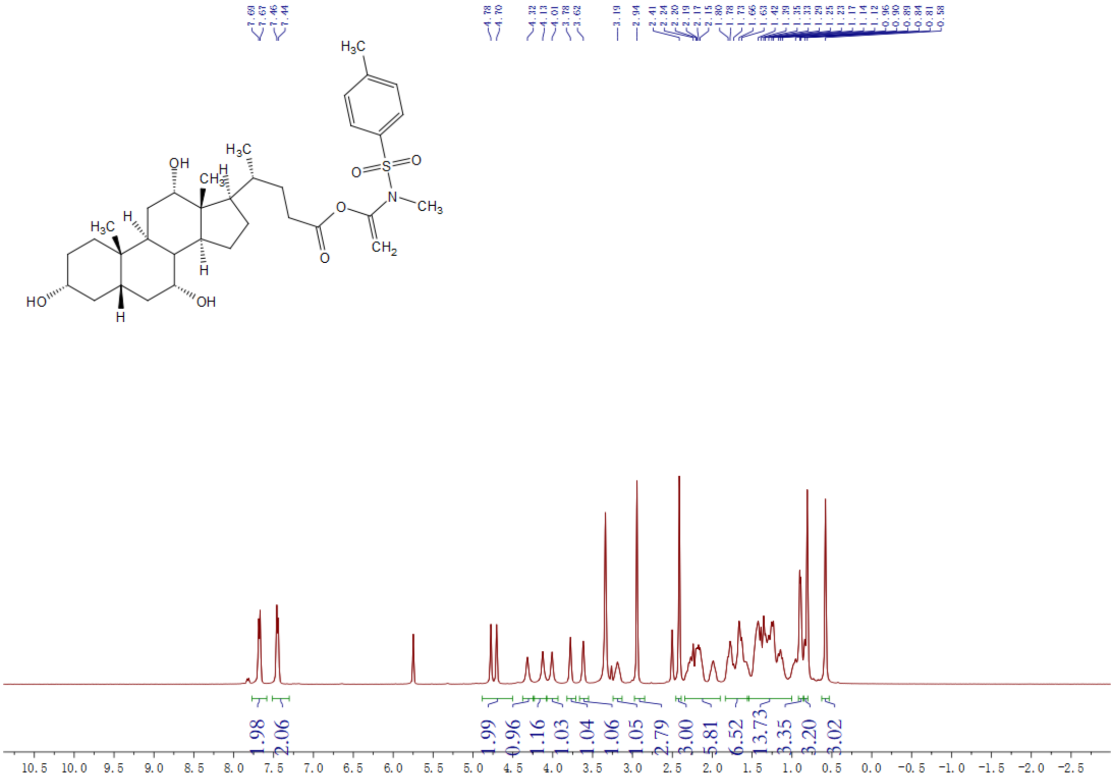

^{13}C of BA-3

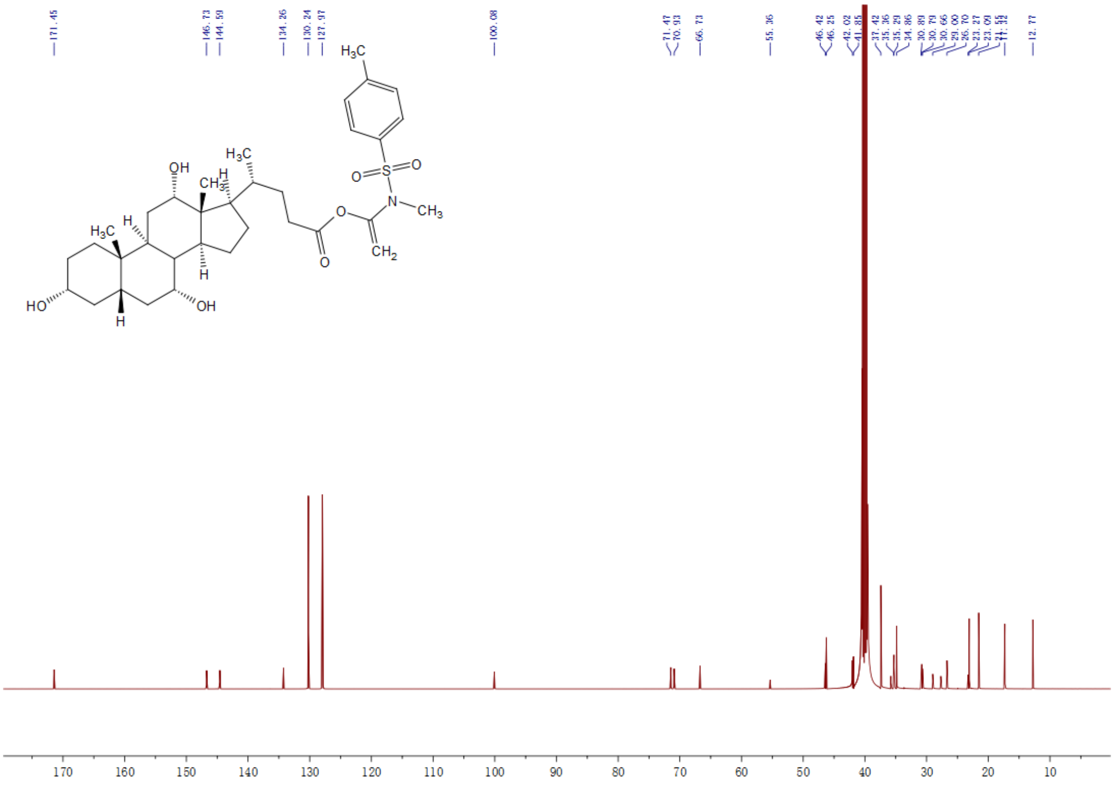

^{1}H of CA-3

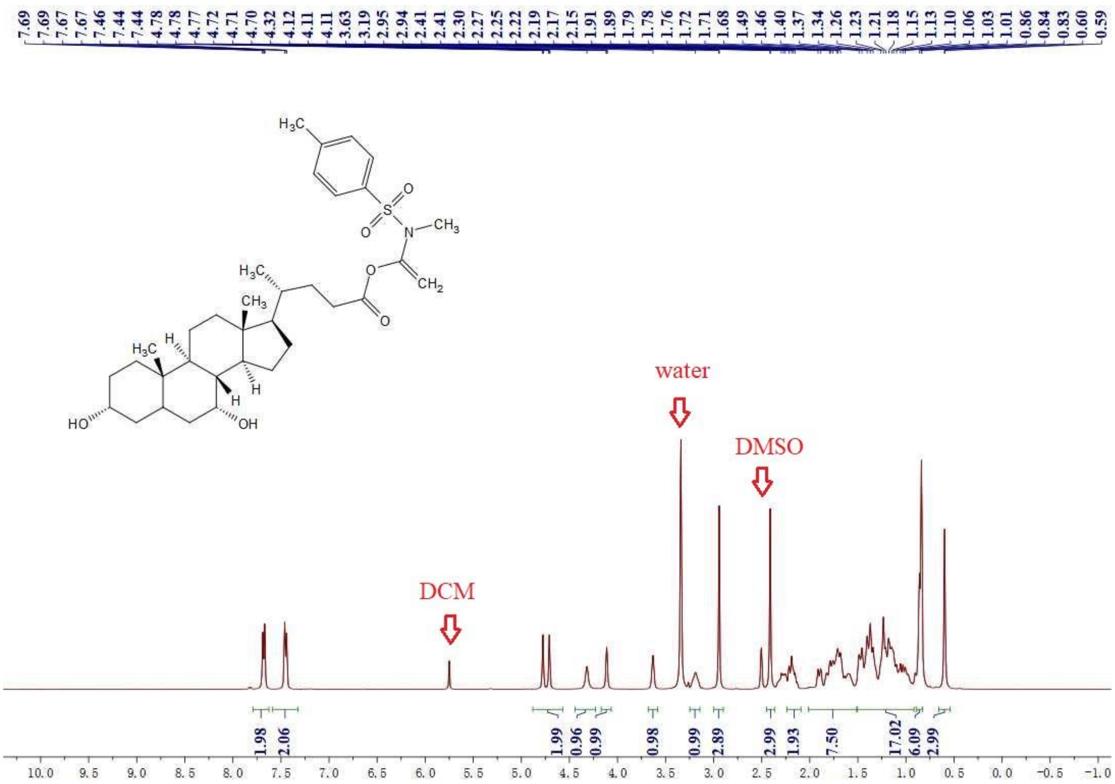

^{13}C of CA-3

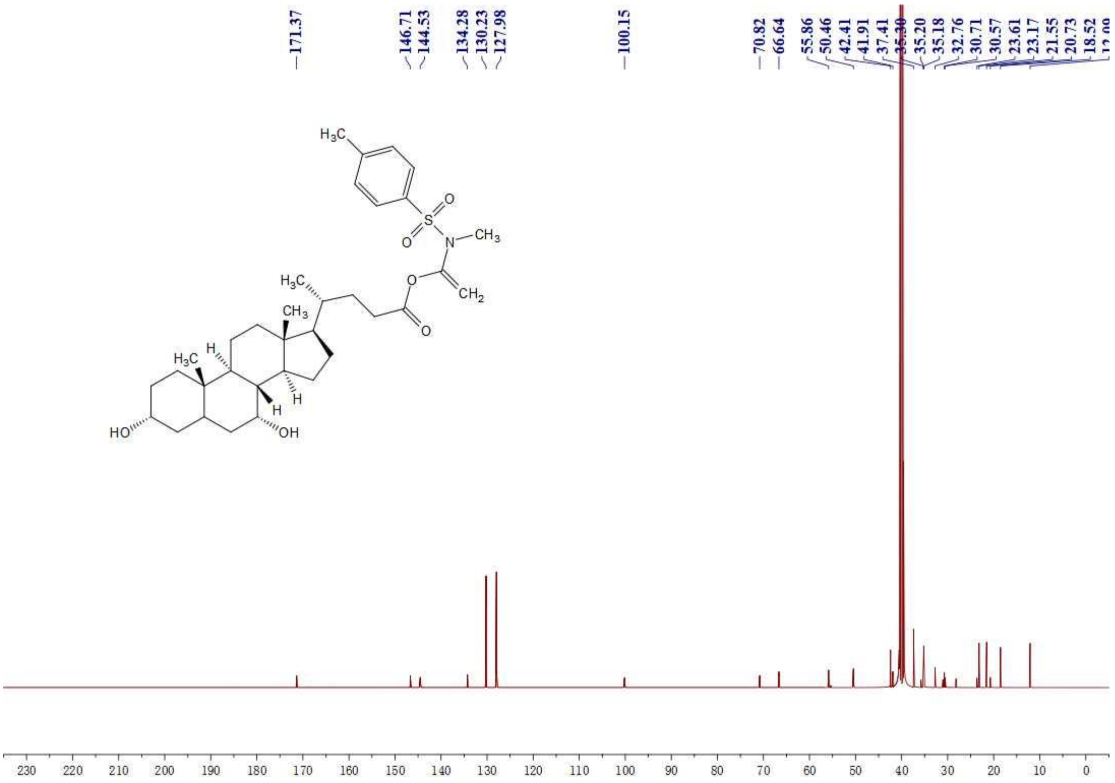

[1]H of UA-3

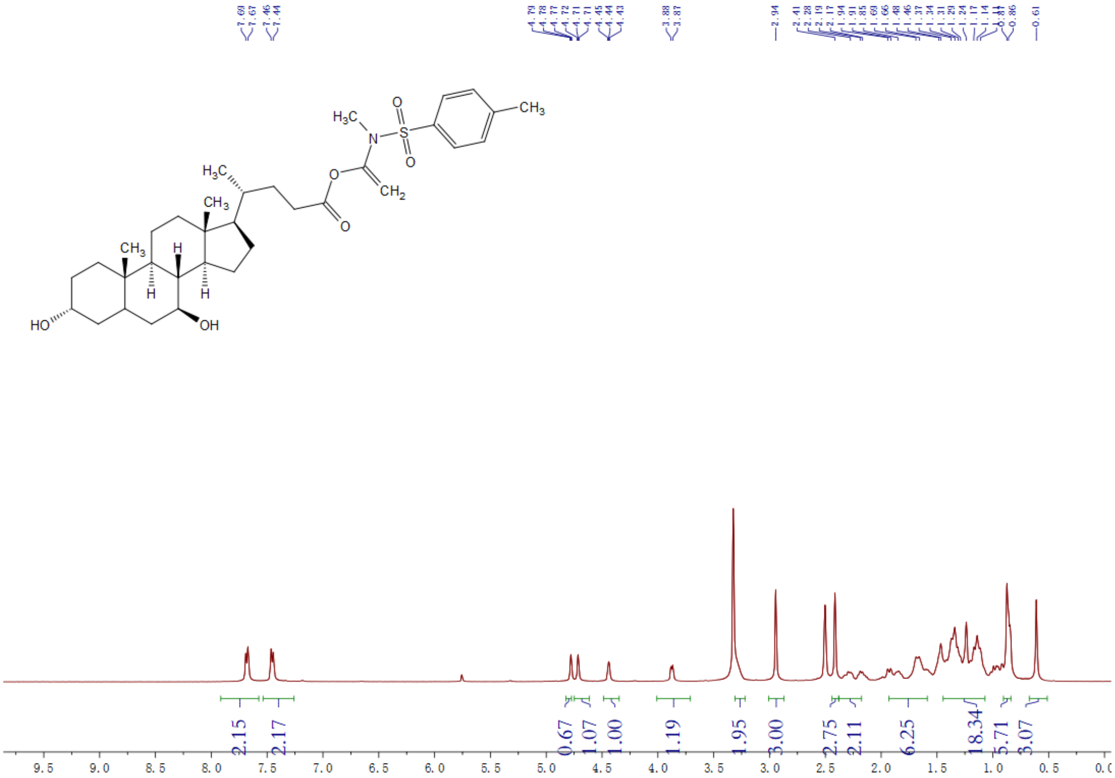

[13]C of UA-3

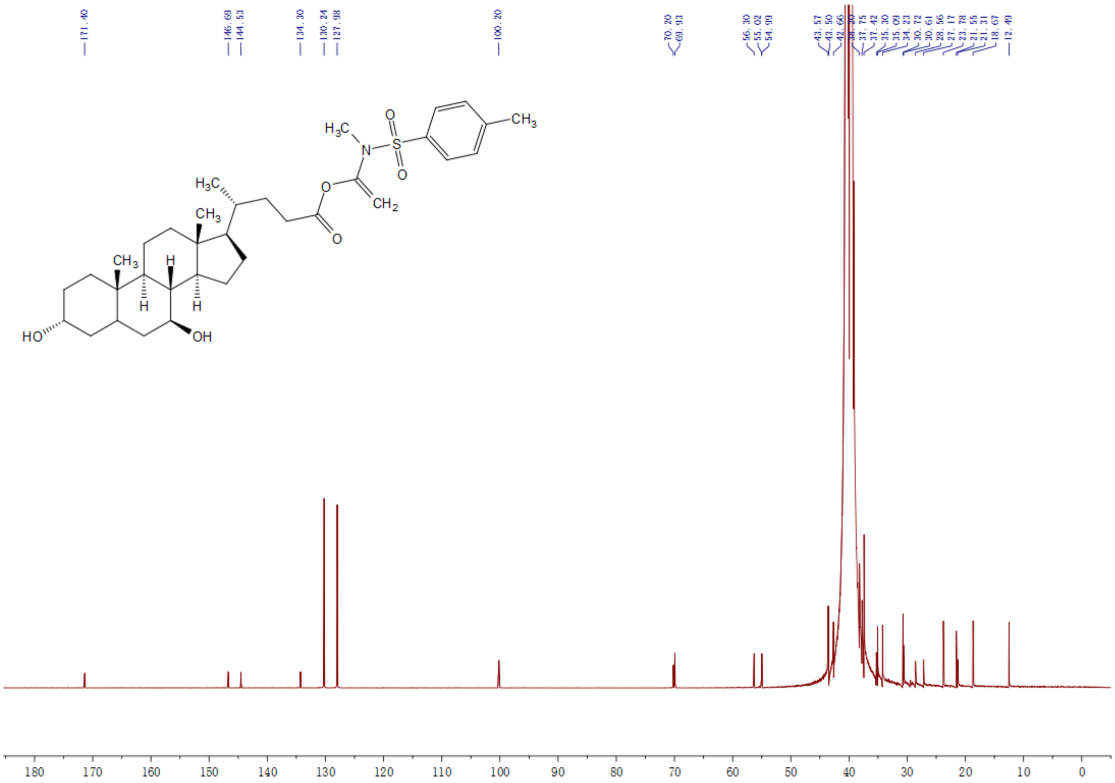

^{1}H of HA-3

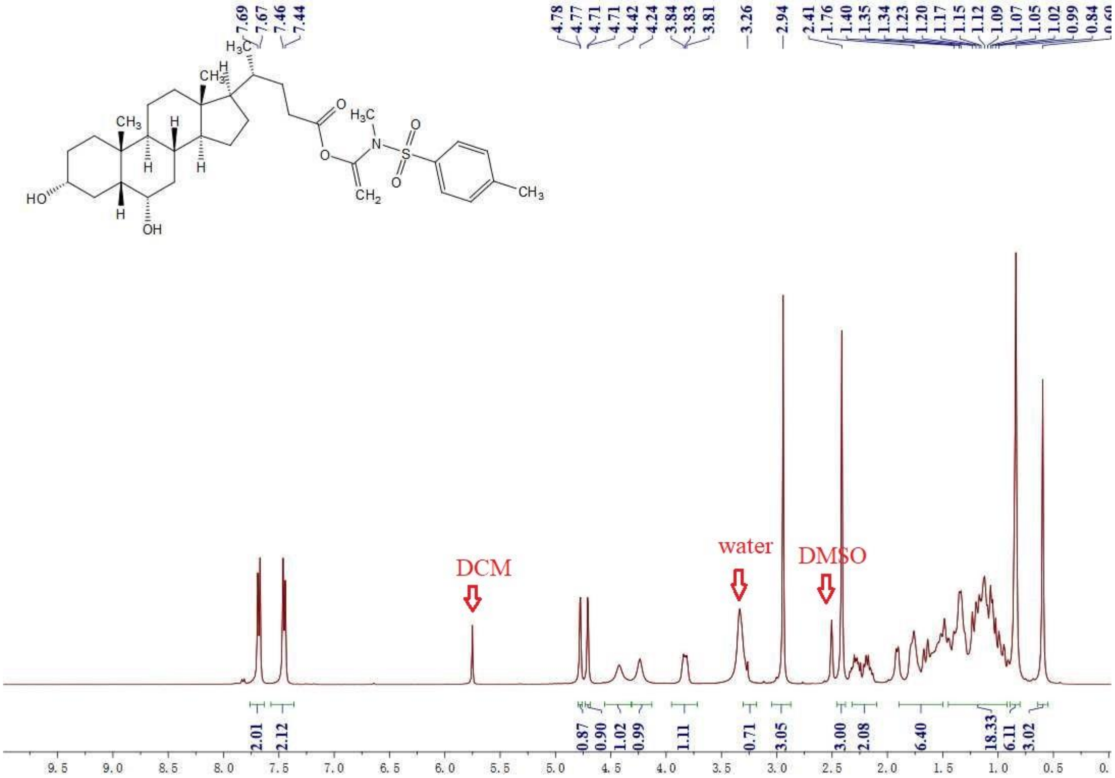

^{13}C of HA-3

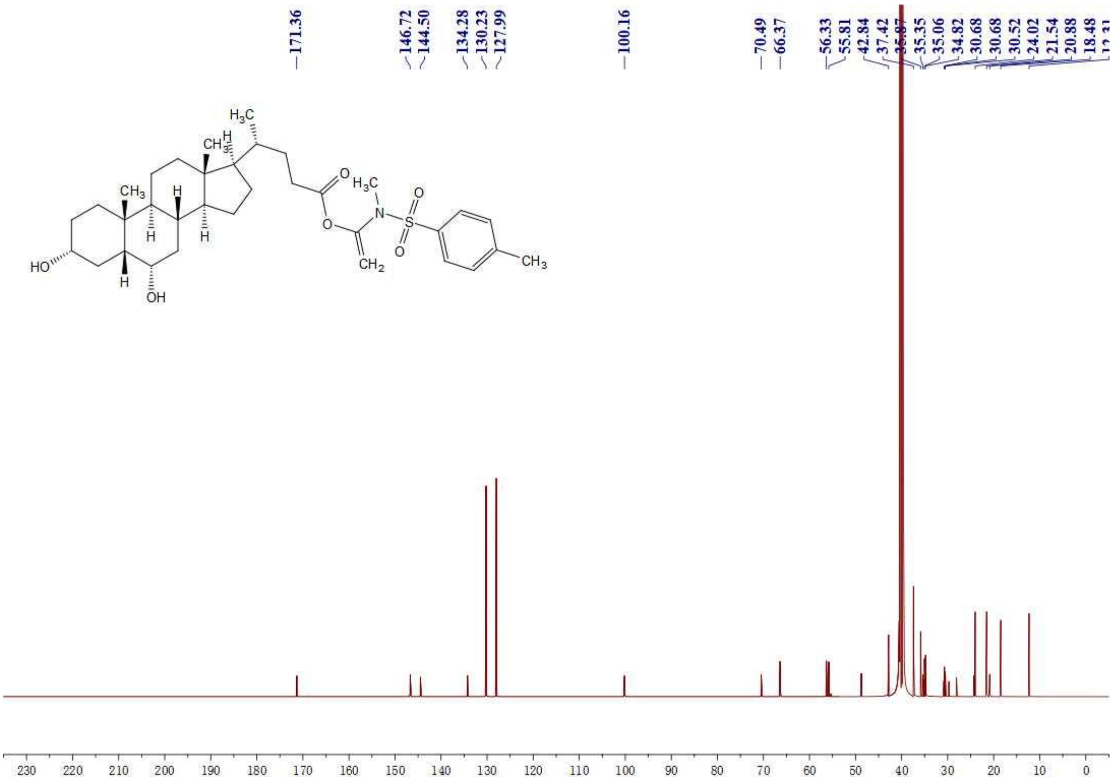

[1]H of CH-3

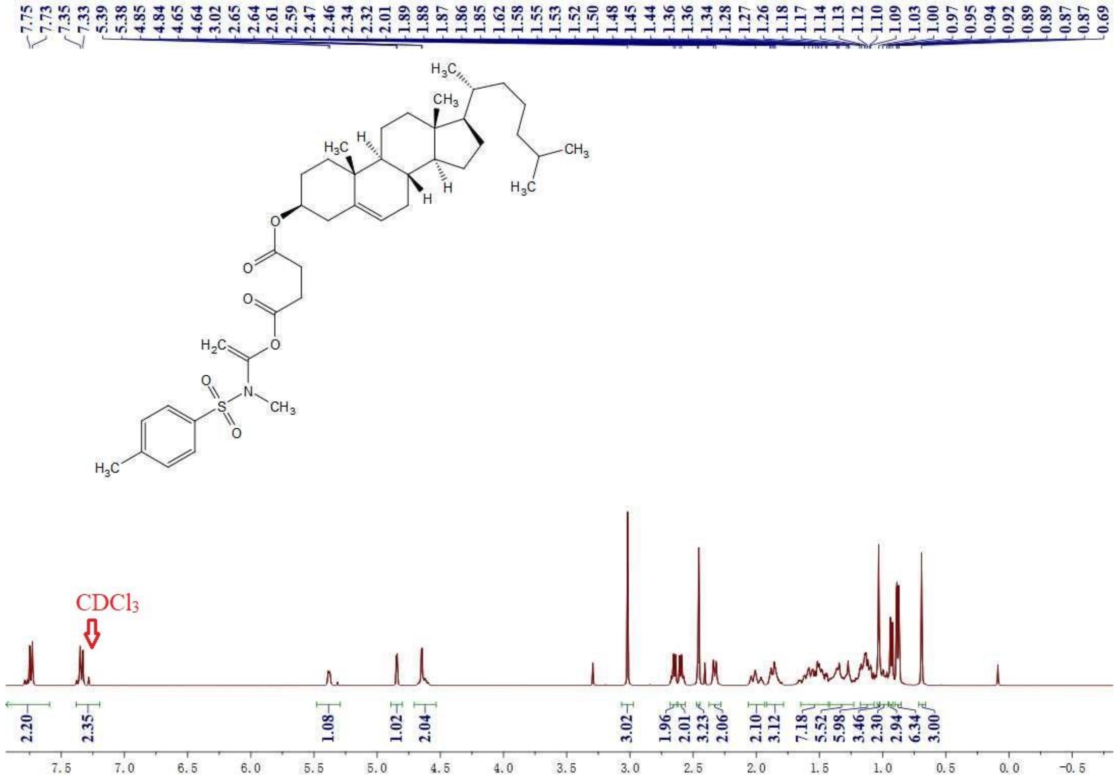

[13]C of CH-3

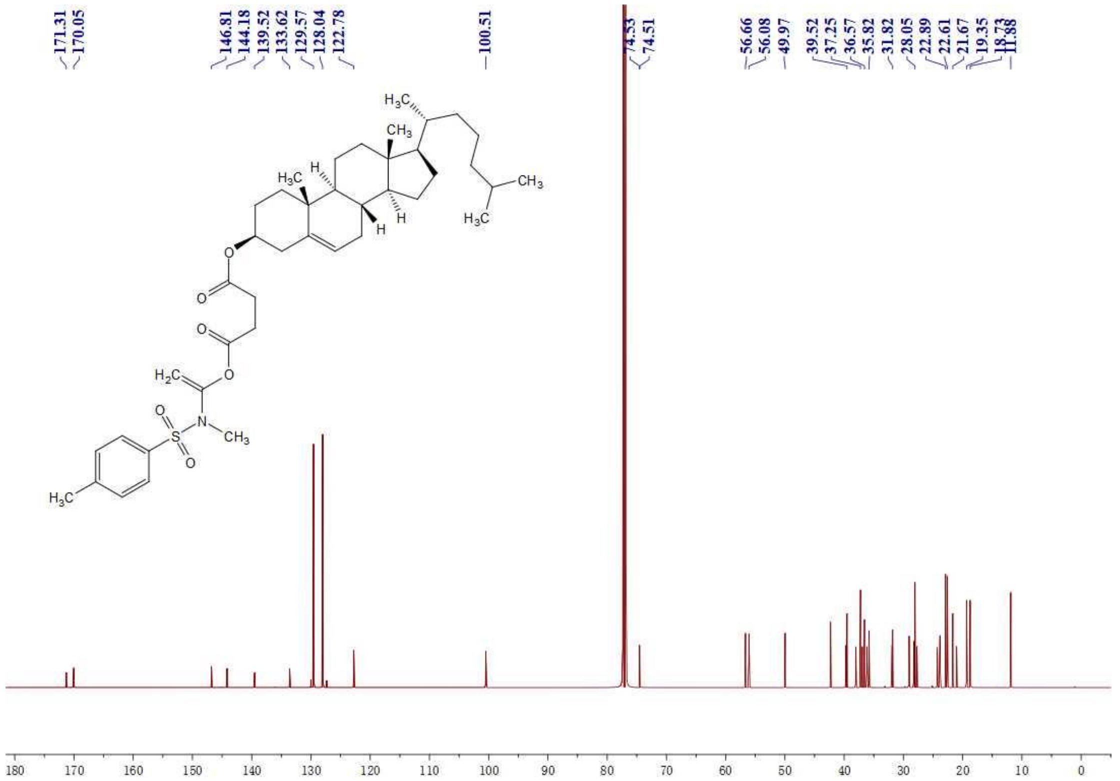

[1]H of GA-3

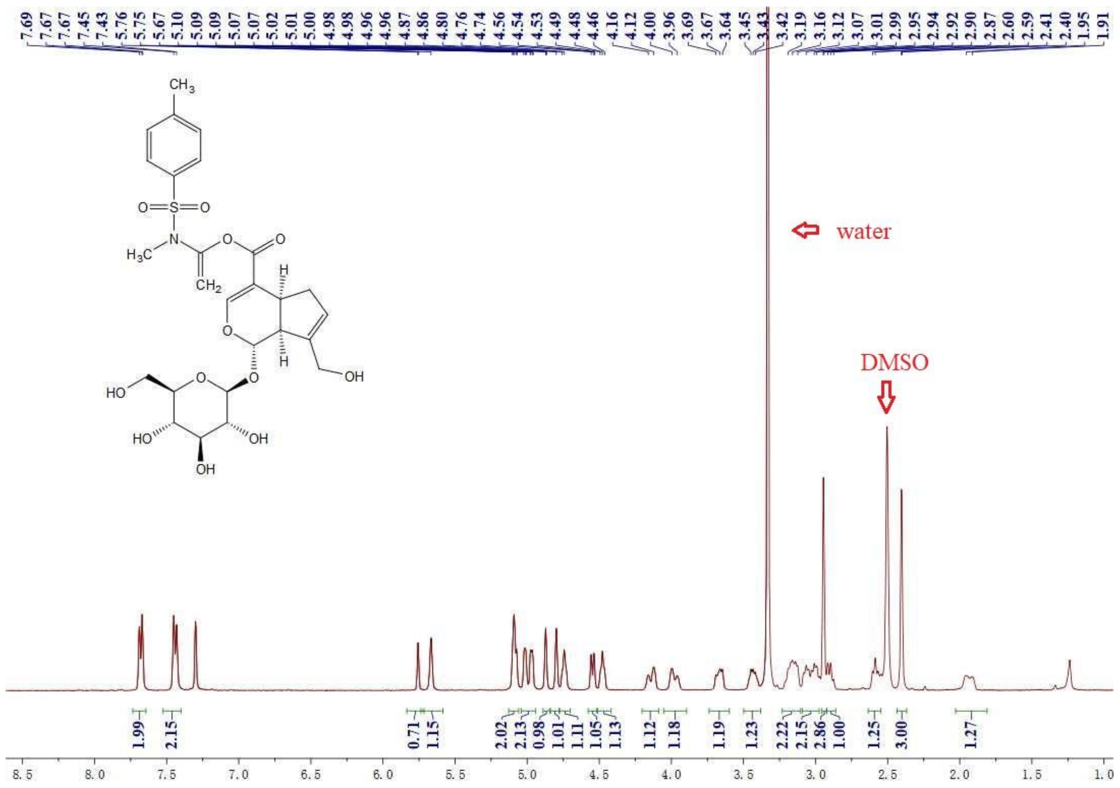

[13]C of GA-3

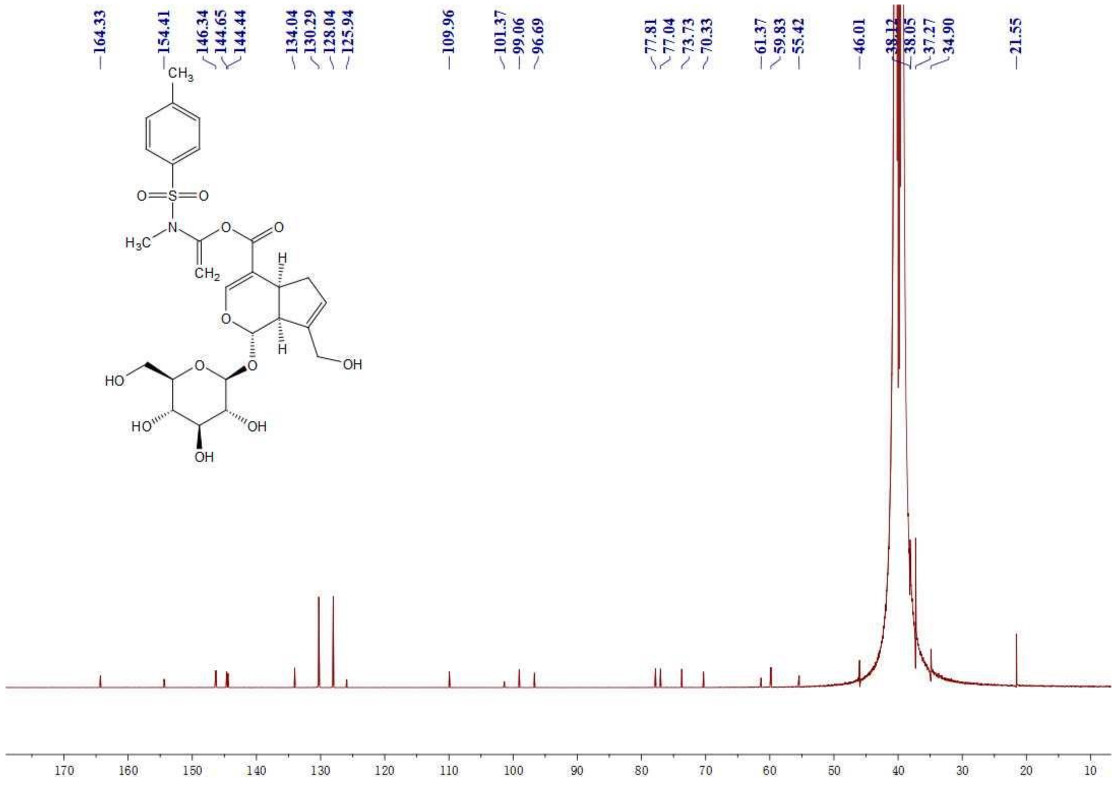

[1]H of Ar-3

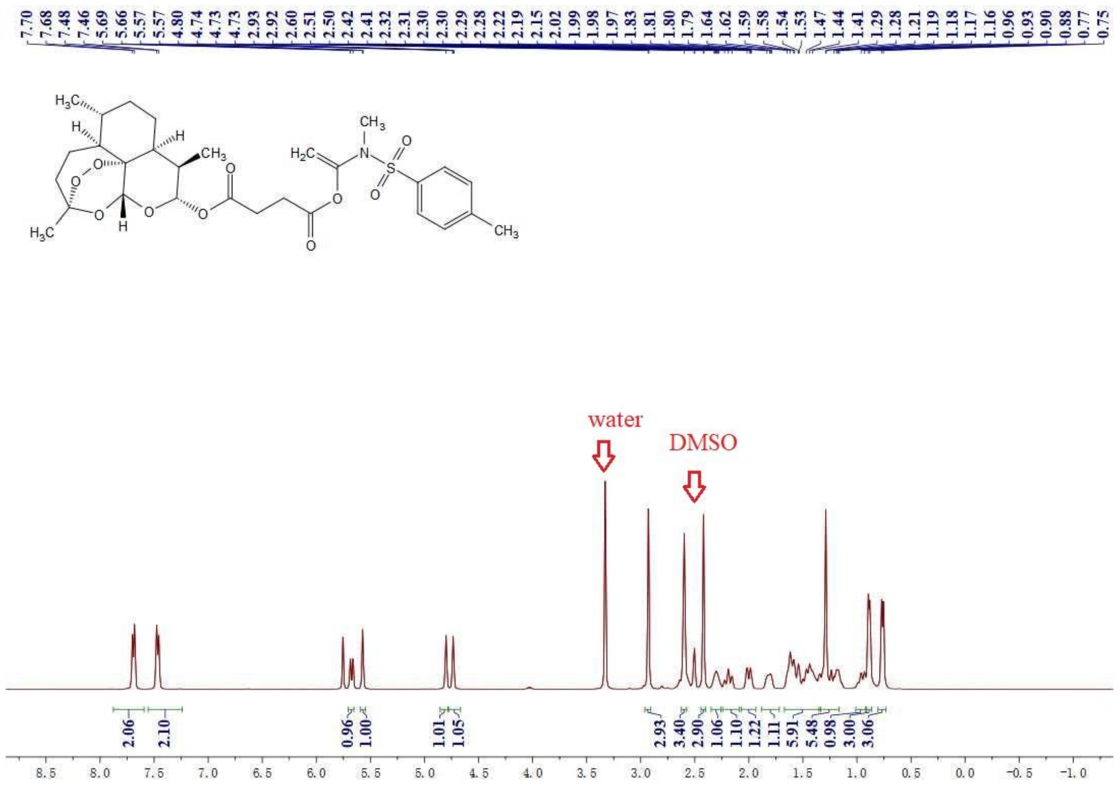

[13]C of Ar-3

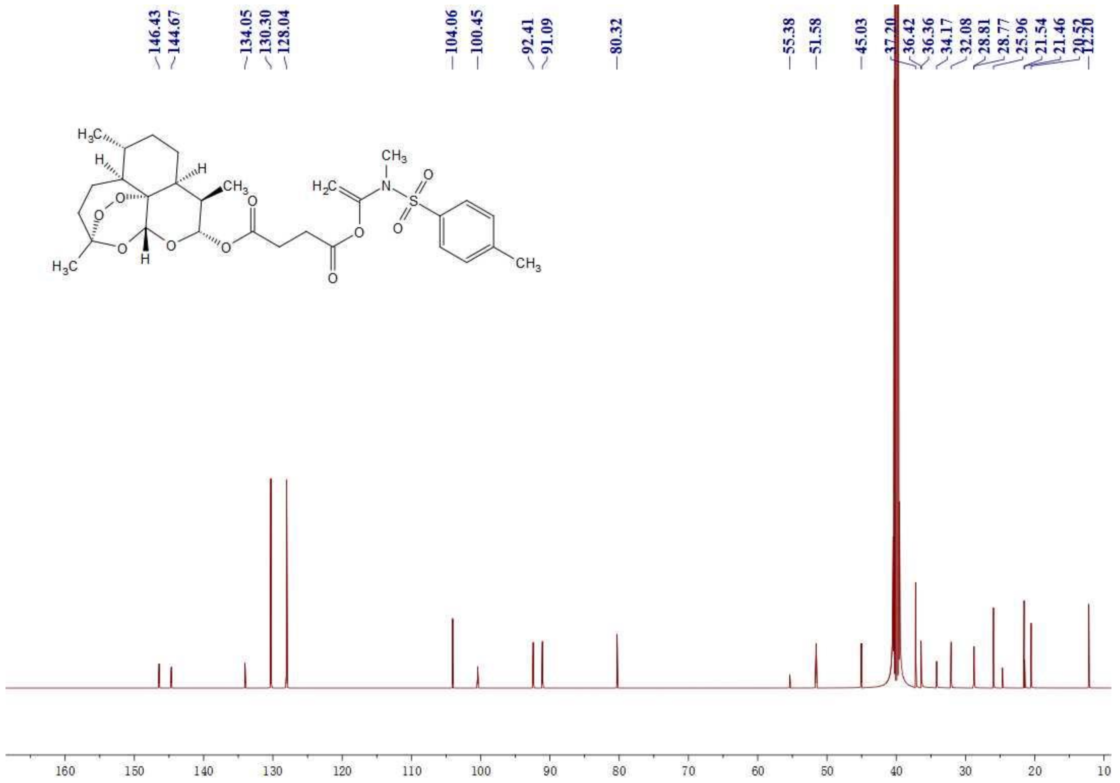

[1]H of MA-3

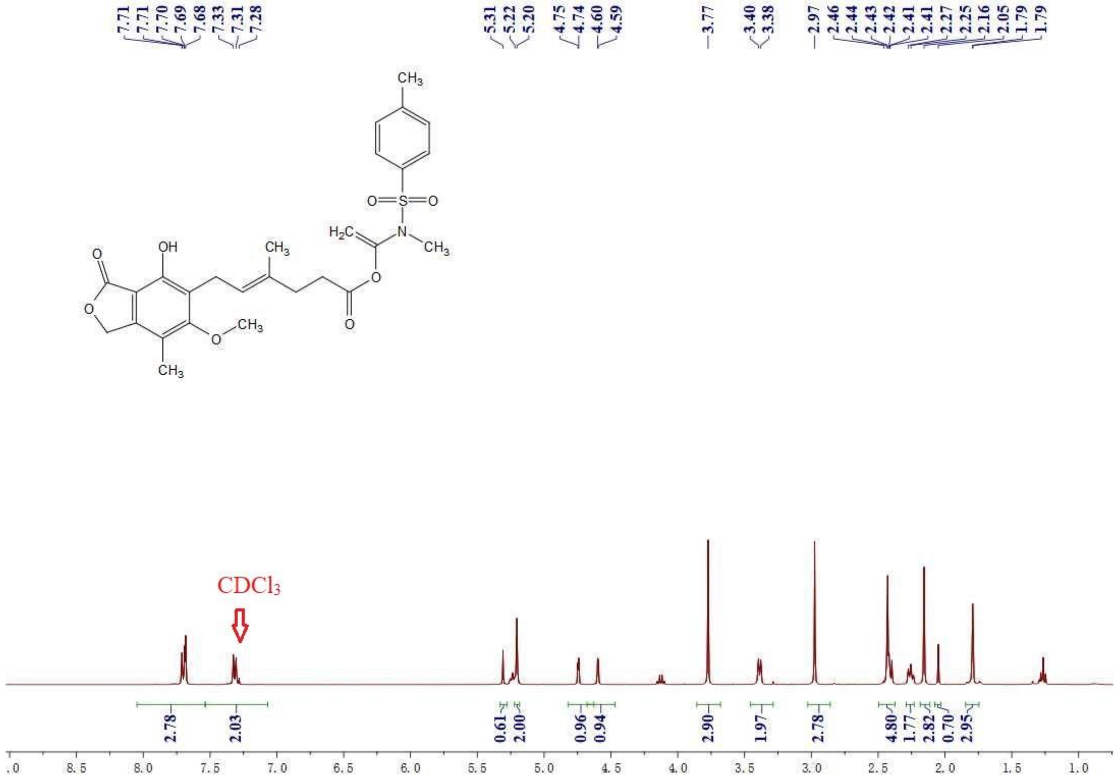

[13]C of MA-3

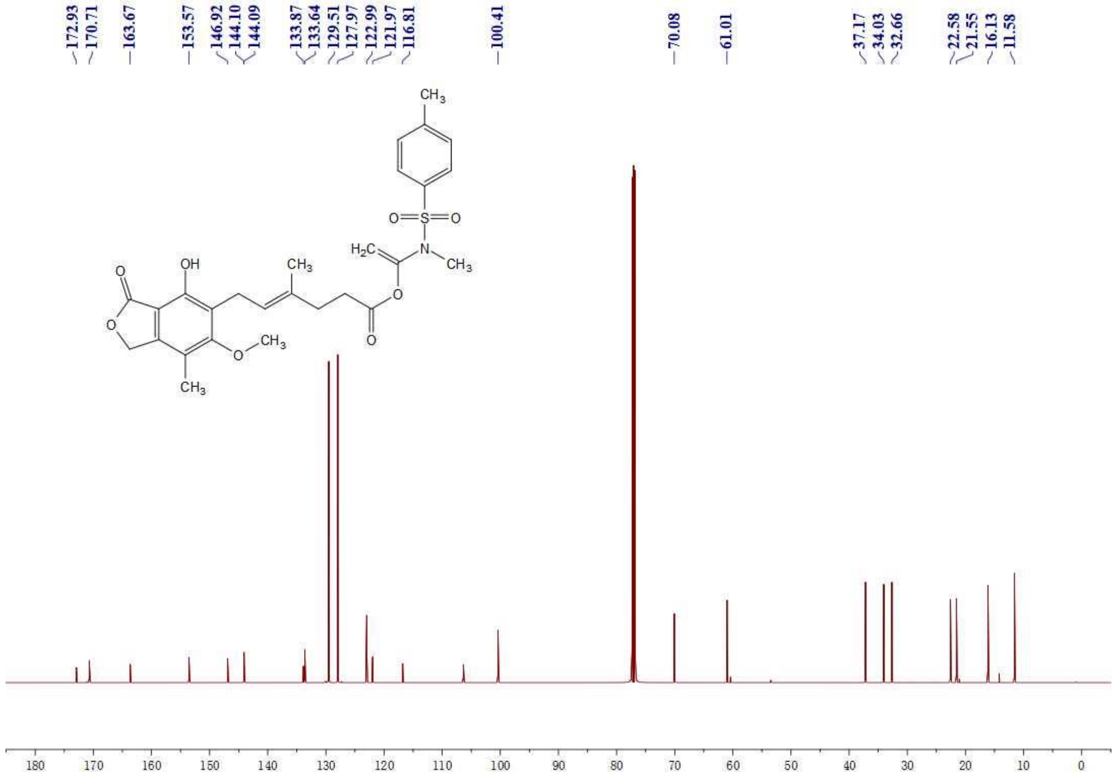

^{1}H of UrA-3

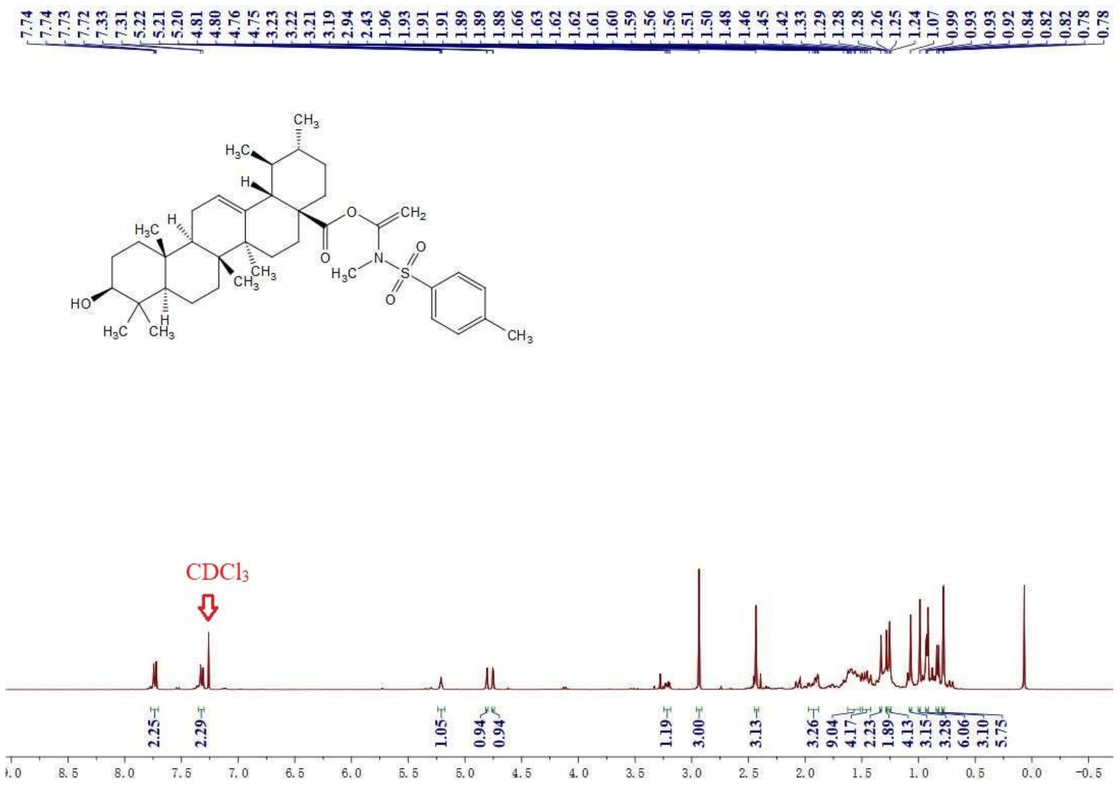

^{13}C of UrA-3

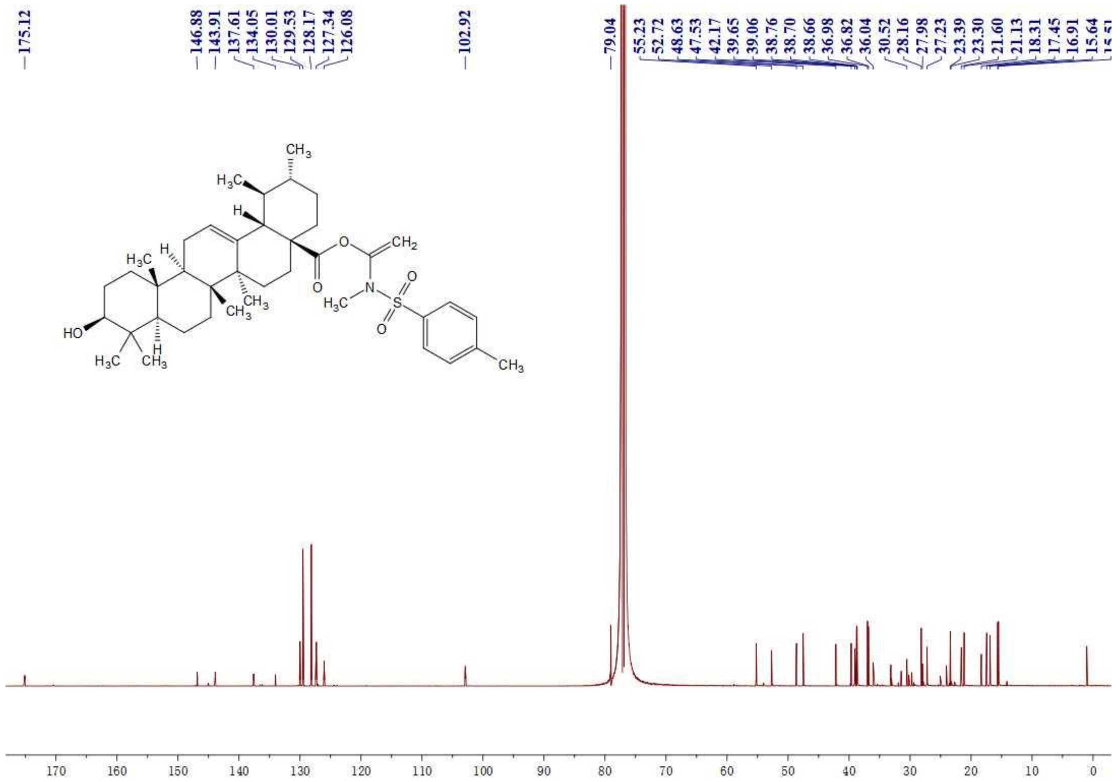

^{1}H of S19

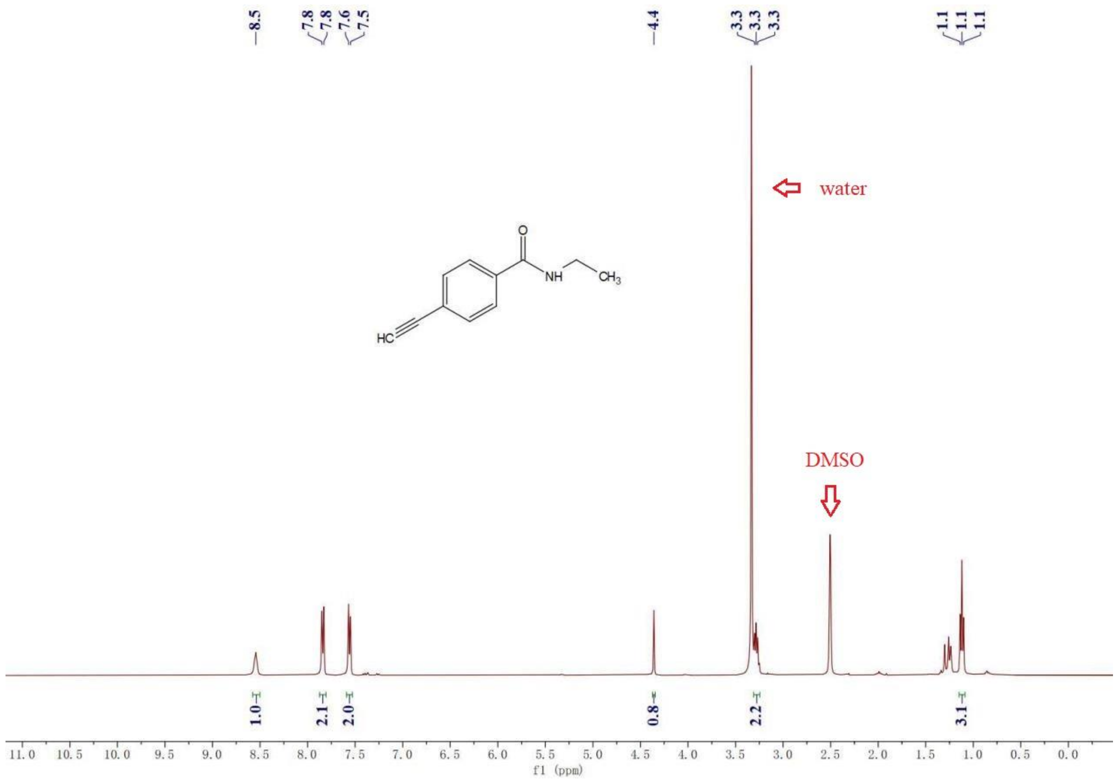
8.5
7.8
7.8
7.6
7.5
4.4
3.3
3.3
3.3
1.1
1.1
1.1
water
DMSO
NH
CH3
HC
O
1.0
2.1
2.0
0.8
2.2
3.1
11.0 10.5 10.0 9.5 9.0 8.5 8.0 7.5 7.0 6.5 6.0 5.5 5.0 4.5 4.0 3.5 3.0 2.5 2.0 1.5 1.0 0.5 0.0
f1 (ppm)
