# Supplementary material for: Catalyst-free late-stage functionalization to assemble α-acyloxyenamide electrophiles for selectively profiling conserved lysine residues
Source: Commun Chem. 2024 Feb 14;7:31. doi: 10.1038/s42004-024-01107-4 (PMC10866925; doi:10.1038/s42004-024-01107-4)
Supplement: Supplementary file 5 — Reporting Summary [file 42004_2024_1107_MOESM5_ESM.pdf]

Corresponding author(s): Zhengqiu Li

Last updated by author(s): Nov 20, 2023

Reporting Summary

Nature Portfolio wishes to improve the reproducibility of the work that we publish. This form provides structure for consistency and transparency in reporting. For further information on Nature Portfolio policies, see our Editorial Policies and the Editorial Policy Checklist.

Statistics

For all statistical analyses, confirm that the following items are present in the figure legend, table legend, main text, or Methods section.

n/a	Confirmed	
☐	☒	The exact sample size (*n*) for each experimental group/condition, given as a discrete number and unit of measurement
☐	☒	A statement on whether measurements were taken from distinct samples or whether the same sample was measured repeatedly
☒	☐	The statistical test(s) used AND whether they are one- or two-sided<br>*Only common tests should be described solely by name; describe more complex techniques in the Methods section.*
☒	☐	A description of all covariates tested
☒	☐	A description of any assumptions or corrections, such as tests of normality and adjustment for multiple comparisons
☒	☐	A full description of the statistical parameters including central tendency (e.g. means) or other basic estimates (e.g. regression coefficient) AND variation (e.g. standard deviation) or associated estimates of uncertainty (e.g. confidence intervals)
☒	☐	For null hypothesis testing, the test statistic (e.g. *F*, *t*, *r*) with confidence intervals, effect sizes, degrees of freedom and *P* value noted<br>*Give P values as exact values whenever suitable.*
☒	☐	For Bayesian analysis, information on the choice of priors and Markov chain Monte Carlo settings
☒	☐	For hierarchical and complex designs, identification of the appropriate level for tests and full reporting of outcomes
☒	☐	Estimates of effect sizes (e.g. Cohen's *d*, Pearson's *r*), indicating how they were calculated

Our web collection on statistics for biologists contains articles on many of the points above.

Software and code

Policy information about availability of computer code

Data collection	1. All biological mass spectrometry raw data in this article were collected using the instrument Thermo Fisher Q Exactive Plus<br>2. labeling gel data in this article were collected using the instrument Typhoon FLA 9500<br>3. celluer imaging and co-localization experiments were collected using the Zeiss LSM800 confocal microscope system equipped with Pln Apo 40 ×/1.3 Oil DICII, 405 nm diode laser, white laser (470–670 nm, with 1 nm increments, with eight channels AOTF for simultaneous control of eight laser lines, each excitation wavelength provides 1.5 mV), and a photomultipliertube (PMT) detector ranging from 410 to 700 nm for steady state fluorescence;<br>4. The cell growth inhibitory activity was detected by CCK8 method, and the absorbance at 450 nm and 650 nm wavelength was measured by a multifunctional microplate reader (Synergy HI, BioTek Instruments, Inc. Vermont, US).
Data analysis	1. The mass spectrometry raw data were processed by using MaxQuant software (1.5.8.3), pchem, pfinder and processed as per default workflow. Searches were performed against the UniProtKB human database (taxonomy 9606, version 20180929).;<br>2. the labeled gel data in this article were analyzed using the software ImageQuant TL<br>3. IC50 values were calculated using Graphpad Prism.<br>4. Docking experiment used custom reaction type in Maestro 11.5.

For manuscripts utilizing custom algorithms or software that are central to the research but not yet described in published literature, software must be made available to editors and reviewers. We strongly encourage code deposition in a community repository (e.g. GitHub). See the Nature Portfolio guidelines for submitting code & software for further information.

Data

Policy information about availability of data

All manuscripts must include a data availability statement. This statement should provide the following information, where applicable:

- Accession codes, unique identifiers, or web links for publicly available datasets
- A description of any restrictions on data availability
- For clinical datasets or third party data, please ensure that the statement adheres to our policy

https://www.ebi.ac.uk/pride/
Project Name: A General Approach to Ligand-directed Proximity Labeling and Target Profiling in Live Cells
Project accession: PXD041109
Project DOI: Not applicable
Reviewer account details:

Username: reviewer_pxd041109@ebi.ac.uk
Password: r4hvESqL

Human research participants

Policy information about studies involving human research participants and Sex and Gender in Research.

Reporting on sex and gender	Not involved in this study
Population characteristics	Not involved in this study
Recruitment	Not involved in this study
Ethics oversight	Not involved in this study

Note that full information on the approval of the study protocol must also be provided in the manuscript.

Field-specific reporting

Please select the one below that is the best fit for your research. If you are not sure, read the appropriate sections before making your selection.

☒ Life sciences ☐ Behavioural & social sciences ☐ Ecological, evolutionary & environmental sciences

For a reference copy of the document with all sections, see nature.com/documents/nr-reporting-summary-flat.pdf

Life sciences study design

All studies must disclose on these points even when the disclosure is negative.

Sample size	Cellular and enzymatic assays were completed using wild type or mutants of EGFR and CDK1/2/5. The sample size is sufficient to report effects of the probes.
Data exclusions	No data excluded
Replication	All the experiments, including protein labeling, enzymatic activity test, cell-based antiproliferation assay, pull-down/WB, pull-down/LC-MS experiments, cellular imaging, docking and CTSA , were performed at least twice with consistent results.
Randomization	The assays performed in this study require a rational approach for activity comparison. Therefore, randomization is not applicable to our experimental set up
Blinding	Blinding is not applicable to any biochemical or cellular assay performed in this study.

Reporting for specific materials, systems and methods

We require information from authors about some types of materials, experimental systems and methods used in many studies. Here, indicate whether each material, system or method listed is relevant to your study. If you are not sure if a list item applies to your research, read the appropriate section before selecting a response.

Materials & experimental systems

n/a	Involved in the study
☐	☒ Antibodies
☐	☒ Eukaryotic cell lines
☒	☐ Palaeontology and archaeology
☒	☐ Animals and other organisms
☒	☐ Clinical data
☒	☐ Dual use research of concern

Methods

n/a	Involved in the study
☒	☐ ChIP-seq
☒	☐ Flow cytometry
☒	☐ MRI-based neuroimaging

Antibodies

Antibodies used	PFKAP antibodies (ab119796), CDK1 antibodies (ab133327), CDK2 antibodies (ab32147), MEK2 antibodies (ab32517) were purchased from abcam company, GAPDH antibodies (AG019-1) was purchased from Beyotime, anti-flag (14793S) and EGFR antibodies (2232S) were purchased from Cell Signaling Technology.
Validation	The above antibodies have been verified using positive controls.

Eukaryotic cell lines

Policy information about cell lines and Sex and Gender in Research

Cell line source(s)	MDA-MB-231 cells are derived from human triple negative breast cancer cells,K562 cells are derived from human chronic leukemia cell line,H3255 cells are derived from human lung cancer,HEK 293T cells are derived fromhuman normal embryonic kidney, cell lines were obtained from the National Cancer Institute Developmental Therapeutics Program (NCI-60).
Authentication	Not involved in this study
Mycoplasma contamination	Not involved in this study
Commonly misidentified lines (See ICLAC register)	Not involved in this study
